# ABA signalling manipulation suppresses senescence of a leafy vegetable stored at room temperature

**DOI:** 10.1111/pbi.12793

**Published:** 2017-08-16

**Authors:** Javier A. Miret, Sergi Munné‐Bosch, Paul P. Dijkwel

**Affiliations:** ^1^ Department of Evolutionary Biology Ecology and Environmental Sciences Plant Physiology Section, Faculty of Biology Barcelona University Barcelona Spain; ^2^ Institute of Fundamental Sciences Massey University Palmerston North New Zealand

**Keywords:** age‐related changes, hormonal regulation, leaf senescence, pyrabactin, redox homeostasis, ubiquitin ligase–proteasome system

## Abstract

Postharvest senescence and associated stresses limit the shelf life and nutritional value of vegetables. Improved understanding of these processes creates options for better management. After harvest, controlled exposure to abiotic stresses and/or exogenous phytohormones can enhance nutraceutical, organoleptic and commercial longevity traits. With leaf senescence, abscisic acid (ABA) contents progressively rise, but the actual biological functions of this hormone through senescence still need to be clarified. Postharvest senescence of detached green cabbage leaves (*Brassica oleracea* var. *capitata*) was characterized under cold (4 °C) and room temperature (25 °C) storage conditions. Hormonal profiling of regions of the leaf blade (apical, medial, basal) revealed a decrease in cytokinins contents during the first days under both conditions, while ABA only increased at 25 °C. Treatments with ABA and a partial agonist of ABA (pyrabactin) for 8 days did not lead to significant effects on water and pigment contents, but increased cell integrity and altered 1‐aminocyclopropane‐1‐carboxylic acid (ACC) and cytokinins contents. Transcriptome analysis showed transcriptional regulation of ABA, cytokinin and ethylene metabolism and signalling; proteasome components; senescence regulation; protection of chloroplast functionality and cell homeostasis; and suppression of defence responses (including glucosinolates and phenylpropanoids metabolism). It is concluded that increasing the concentration of ABA (or its partial agonist pyrabactin) from the start of postharvest suppresses senescence of stored leaves, changes the transcriptional regulation of glucosinolates metabolism and down‐regulates biotic stress defence mechanisms. These results suggest a potential for manipulating ABA signalling for improving postharvest quality of leafy vegetables stored at ambient temperature.

## Introduction

Food loss represents up to a third of worldwide production, but 45% of fruits and vegetables deteriorate before reaching the consumer (FAO, 2016). In developing countries, the postharvest losses of vegetables may account for even a higher fraction of produce yield (FAO, 2016; Thongsavath *et al*., 2012). Global change and the forecasted increase in the frequency of extreme weather events will further strain the production and logistic chain from the field to the consumer, especially in the developing world (Gustavsson *et al*., 2011; Thongsavath *et al*., 2012).

Postharvest senescence and stresses limit the storage and shelf life of vegetables; with an improved understanding of the perception of this environment and the subsequent physiological responses, options for better management become possible (Toivonen, 2004; Toivonen and Hodges, 2011). From the time of harvest, quality declines in vegetables, including visual symptoms, such as loss of turgor and yellowing, as well as loss of nutraceutical and organoleptic metabolites (Cantwell and Suslow, 2001). Low‐temperature storage is the single most effective method for prolonging postharvest life, reducing respiration rate, ethylene production and sensitivity, moisture loss and growth of pathogens (Thompson *et al*., 2002). However, cold postharvest storage can have a negative impact on the organoleptic and nutraceutical traits of leafy vegetables (Cantwell and Suslow, 2001; Kramchote *et al*., 2012; Liu *et al*., 2015), and not in all markets the cold chain can be properly maintained (Thongsavath *et al*., 2012). Environmental factors can be leveraged to increase nutritional value, antioxidant content, organoleptic characteristics and eventually commercial longevity, but storage at ambient conditions can hinder produce quality and value (Adams *et al*., 2016; Toivonen and Hodges, 2011; Villarreal‐García *et al*., 2016). Therefore, methods to ensure the postharvest quality of vegetables at room temperature should be improved.

Plant hormones are known to affect leaf senescence, but they also regulate plant development and stress responses (Bartoli *et al*., 2013). For this reason, it has been difficult to unravel how different hormones regulate the onset and progression of leaf senescence; if acting directly on leaf senescence, or indirectly through altering the developmental programme or the response to stresses. Leaves are insensitive to senescence‐inducing environment signals till they have reached a certain developmental stage (Jibran *et al*., 2013; Jing *et al*., 2005). Depending on the previous occurrence of accumulated age‐related changes, the leaf can integrate environmental cues, which, if adverse, will induce the onset of senescence. The role of the rise of ABA contents and ABA signalling changes during leaf senescence is less clear (Sun *et al*., 2016; Zhao *et al*., 2016). ABA is involved in both developmental processes and stress responses (Finkelstein, 2013). In many processes, ABA promotes accumulation of antioxidants and protects chloroplast functionality (Ghassemian *et al*., 2008; Ivanov *et al*., 1995; Leng *et al*., 2014). Therefore, manipulation of its contents or signalling offers potential for biotechnological applications, including the regulation of dormancy and germination (Rademacher, 2015), manipulation of fruit maturation (Ji *et al*., 2012; Miret and Munné‐Bosch, 2016; Setha, 2012), tissue culture systems (Rai *et al*., 2011) and enhancing tolerance to abiotic stresses (Blum, 2015; Travaglia *et al*., 2010; Zhao *et al*., 2016). Further, ABA may control many of the developmental age‐related changes associated with sensibility to senescence‐inducing signals (Jibran *et al*., 2013), notably keeping redox and membrane homeostasis. These activities also play a role in protecting the cellular functions required for progression and completion of senescence (Jibran *et al*., 2013; Lim *et al*., 2007; Zhao *et al*., 2016). At the same time, ABA may promote senescence by promoting ethylene biosynthesis or through ethylene‐independent pathways (Zhao *et al*., 2016). Besides, the relationship between ABA and ethylene synthesis and signalling differs depending on organ type, age and physiological state (Ghassemian *et al*., 2000). Thus, the potential effect of ABA on leaf senescence strikes as being strongly dependent on the developmental state of the leaf.

The discovery of the PYR/PYL/RCAR ABA receptor family (Park *et al*., 2009) has accelerated the unravelling of ABA signal transduction and its interaction with ABA response modulators and effectors. In the presence of ABA, a complex is formed between ABA, PYR/PYL/RCAR receptors and PP2Cs, releasing SnRK2 from PP2Cs inhibition. SnRK2s then activate effectors and regulators of ABA response. Free PP2Cs can also interact with other effectors and regulators (Park *et al*., 2009; Umezawa *et al*., 2010). Further, phosphatases and ubiquitin ligases act as a layer of post‐translational regulation of most elements of this system whose biological function we are only starting to decipher (Mazzucotelli *et al*., 2008; Yang *et al*., 2017).

Green cabbage (*Brassica oleracea* L. var. *capitata*) is an economically important crop, widely consumed either fresh, steamed or cooked (Chiang *et al*., 1993). Cabbage is rich in health beneficial secondary metabolites that prevent chronic degenerative diseases, such as antioxidants, glucosinolates, phenylpropanoids and other bioactive compounds (Manchali *et al*., 2012; Podsędek, 2007). These compounds not only present nutraceutical and organoleptic interest, but also have been associated with increased abiotic and biotic stress tolerance in crops (Ishida *et al*., 2014). *Brassica oleracea* breeding has generated a wide diversity of varieties, with different morphology and edible parts (Liu *et al*., 2014). Research into postharvest senescence has focussed on broccoli florets, while leafy *B. oleracea*, such as pak choi, brussels sprouts, kale and cabbage, is less well studied. Broccoli postharvest senescence is marked by a decline in carbohydrates, proteins and antioxidants leading to pigment deterioration, lipid peroxidation and eventual necrosis (Coupe *et al*., 2003). These results may partially extend to leafy *Brassica*; however, while they belong to the same species, comparisons should be cautious as broccoli comprises immature inflorescences while cabbage represents leaves.

The postharvest senescence of detached green cabbage leaves was characterized in a first experiment, mimicking typical storage conditions. After purchase, leaves were stored either in a cold room (cold treatment; emulating a fridge) or in a laboratory growth chamber (room temperature; cupboard). Over 9 days of storage, a physiological and hormonal profile of different regions of the leaf (apical, medial and basal strips) was obtained, identifying ABA at the start of postharvest as a possible target for manipulation. Biochemical and transcriptomic tools were applied to study the effects of ABA/Pyr treatments to hormonal regulation, leaf function homeostasis, and the regulation of relevant nutraceutical and organoleptic compounds during postharvest under room temperature storage conditions.

## Results

### Characterization of green cabbage postharvest senescence under contrasting storage conditions

**Figure 1:**
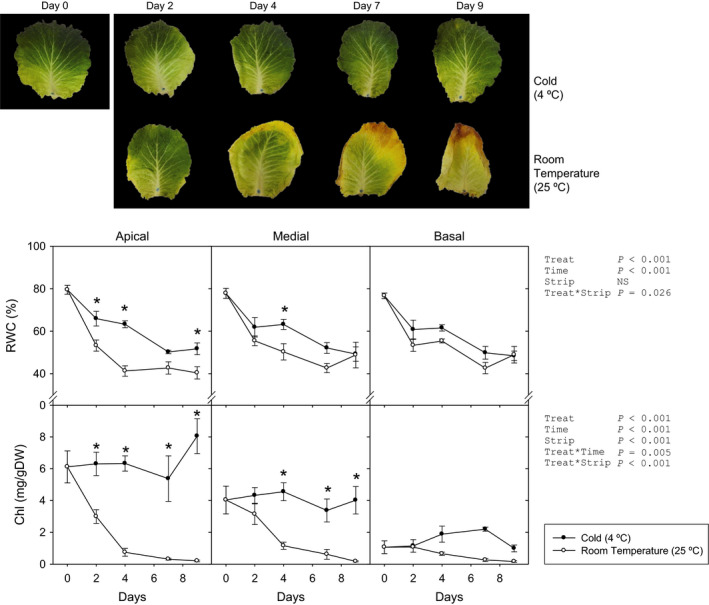
(Top) photographs of cold‐ and room‐temperature‐stored green cabbage leaves. (Bottom) Relative water content (RWC) and chlorophyll (Chl) of apical, medial and basal strips of green cabbage leaves under cold and room temperature storage conditions. Data points represent mean±SE of five replicates. Three‐way ANOVA *P*‐values, asterisks indicate differences between experimental conditions.

First, cabbage postharvest senescence under cold and room temperature conditions was studied. Whole leaf biomass decreased under room temperature conditions (RT, 25 °C), losing half its initial weight after 10 days (Figure S2) even under high relative humidity (75%). In contrast, after storage at 4 °C, leaves lost just 20%. All other physiological and biochemical analyses were performed independently for apical, medial and basal strips of the leaf, separated at sampling (Figures 1 and S1–S8). Apical strips from leaves kept at 4 °C showed less water loss than those kept at RT, while only slight differences appeared in the basal strip. After 4 days of storage, the maximum efficiency of the photosystem II (*F*_v_/*F*_m_) dramatically decreased in all strips at RT, with no changes under cold conditions. Likewise, decreases in chlorophyll levels depended on storage temperature, with carotenoids following similar patterns in the medial and basal strips, and no changes in the apical strip. These changes were manifested as a progressive yellowing and an appreciable water loss, starting in the apex and margins of the leaf blade (Figure 1). Leaves maintained at 4 °C remained green, with a certain water loss only appearing after more than 8 days.

**Figure 2:**
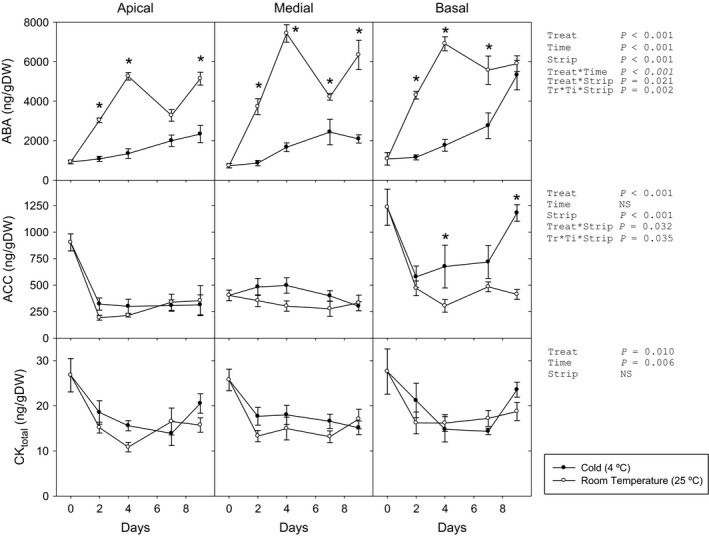
Abscisic acid (ABA), 1‐aminocyclopropane‐1‐carboxylic acid (ACC) and CK_total_ (sum of analysed cytokinins) of three strips of green cabbage leaves under cold and room temperature storage conditions. Data points represent mean±SE of five replicates. Three‐way ANOVA *P*‐values, asterisks indicate differences between experimental conditions.

The storage conditions caused major changes in the hormonal profile (Figures 2 and S5–S8). After an initial decrease in cytokinin contents (CKs), no differences appeared in CK_total_ contents between strips. Only in the basal strip of the room‐temperature‐stored (RT) leaves, *t*‐zeatin remained at the initial levels but decreased when kept at 4 °C. Dihydrozeatin, the most abundant cytokinin, decreased during the first 4 days across all strips without differences by storage condition. ABA contents increased considerably in leaves stored at RT, following a similar pattern in the three analysed strips—up to six‐ to ninefold of initial contents. Cold‐stored leaves showed a smaller rise in apical and medial strips (threefold), while the basal strip presented a later but major increase. At the same time, ACC contents (immediate precursor of ethylene) showed distinct patterns in the different strips under each storage condition. No differences between storage conditions were observed in the apical and medial strips, while in the basal strip ACC accumulated only under cold storage condition.

### Characterization of cabbage postharvest physiology after ABA signal manipulation by ABA/Pyr

**Figure 3:**
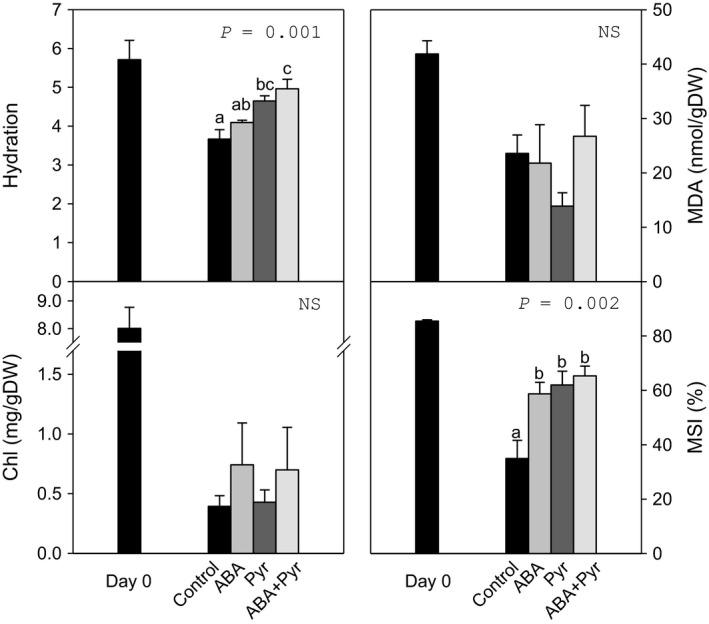
Hydration, chlorophyll (Chl), malondialdehyde (MDA) and membrane stability index (MSI) of the apical strip before and after 8 days of ABA/Pyr treatment under room temperature storage conditions. Data points represent mean±SE of five replicates. ANOVA *P*‐values, letters indicate differences between experimental conditions.

ABA contents increased over sixfold in tissue stored at RT. Therefore, in a subsequent experiment, ABA and/or pyrabactin (ABA/Pyr) were applied exogenously to leaves stored at RT every other day, for 8 days. Pyrabactin is a synthetic sulfonamide that is a partial agonist of ABA: (i) acting as an ABA agonist on certain PYR/PYL/RCAR receptors; (ii) binding other receptors without promoting signal transduction, thus antagonizing ABA productive binding; or (iii) with no interaction, such as with PYL9 or the plastidial ABA receptor CHLH. Pyrabactin application is of potential agronomic interest due to this selectivity, possibly presenting more limited effects than ABA application and thus also limiting potential undesired effects of ABA signalling manipulation on quality and yield. In addition, pyrabactin does not suffer from the same photosensitivity that ABA is subject to. ABA/Pyr treatments did not significantly affect pigments levels (Chl, carotenoids; Figures 3 and S9), but increased apical strip hydration, as compared to untreated leaves. The application of ABA, Pyr or the combined ABA+Pyr treatment increased membrane stability (MSI). Meanwhile, *F*_v_/*F*_m_, dependent on membrane integrity, showed no significant difference between treatments. Malondialdehyde (MDA) contents, a product of lipid peroxidation, also did not present significant differences. Neither hydrosoluble (ascorbic acid, ascorbate redox state) nor liposoluble (total carotenoids, α‐tocopherol) antioxidant levels were different between treatments. Another measure of membrane and cell wall integrity, and cell homeostasis is the main soluble cation content. Pyrabactin treatment increased soluble cations, especially K^+^, but no further differences as compared to untreated control leaves were found (Figure S10).

**Figure 4:**
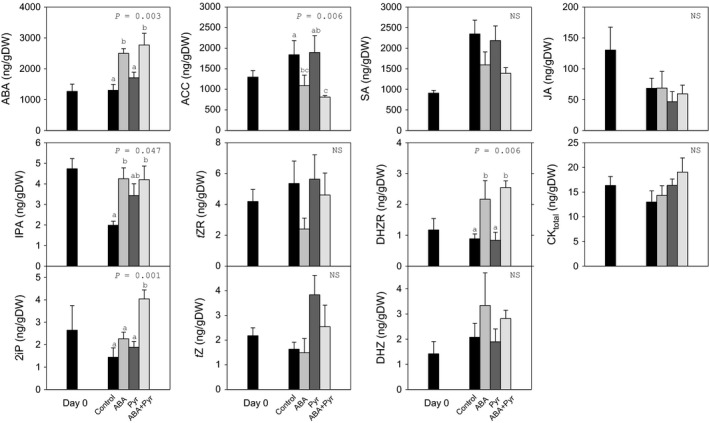
Effects of ABA application on endogenous hormonal balance. Abscisic acid (ABA), 1‐aminocyclopropane‐1‐carboxylic acid (ACC), salicylic acid (SA), jasmonic acid (JA), total cytokinins (CK_total_), *trans*‐zeatin ( *t*Z), dihydrozeatin (DHZ), 2‐isopentenyladenine (2iP), *trans*‐zeatin riboside ( *t*ZR), dihydrozeatin riboside (DHZR) and isopentenyladenosine (IPA) of the apical strip of green cabbage leaves before and after 8 days of ABA/Pyr treatment under RT storage conditions. Data points represent mean±SE of five replicates. ANOVA *P*‐values, letters indicate differences between experimental conditions.

The manipulation of ABA signalling through storage with the repeated application of ABA/Pyr represented great changes to the hormonal profile (Figure 4). Application of ABA every other day caused a more than doubling of endogenous ABA contents, when ABA was applied either alone or combined with pyrabactin. No treatment showed significant changes in total cytokinin contents, but several analysed cytokinins showed different responses to ABA/Pyr. The contents of cytokinin nucleosides IPA and DHZR rose with ABA treatments (alone or combined with Pyr), while *t*ZR levels were not affected. Meanwhile, no significant differences were found in the levels of the cytokinin free bases *t*Z and DHZ. Only the combined ABA+Pyr treatment increased 2iP contents, while Pyr alone had no significant effect on cytokinins profile. After 8 days, ACC contents in Pyr‐treated leaves remained at the same level of the control, but were halved after ABA and the ABA+Pyr combined treatment. Neither jasmonic acid (JA) nor salicylic acid (SA) contents were affected after ABA/Pyr treatments for 8 days.

Thus, ABA and ABA+Pyr treatments raised ABA contents, impacting water content and membrane integrity, but also representing notable effects on ACC contents and individual CKs nucleosides and free bases.

### Transcriptomic analysis of ABA/Pyr treatment effects

To better understand the effects of storage and ABA signalling manipulation through ABA/Pyr treatment, we performed a transcriptomic analysis of its effects on the apical strip. After 8 days at RT conditions, total RNA was extracted from the apical strip of three biological replicates for each treatment (control, ABA, Pyr, ABA+Pyr). The HiSeq 2000 sequencing platform generated 21.8–30.2 million 100‐base paired‐end reads for each of the twelve libraries (4 conditions × 3 biological replicates; Table S1) for a total 306 200 398 raw reads. Quality and rRNA content were assessed with FastQC (Andrews, 2010) and riboPicker (Schmieder *et al*., 2012). Low‐quality reads and reads mapped to rRNA were removed for downstream analyses, resulting in 21.5–29.9 million high‐quality reads per biological sample (Table S1).

As no comprehensive transcriptome was available for *Brassica oleracea*, we first aligned the high‐quality reads to *Brassica oleracea* v1.0 reference genome (Liu *et al*., 2014). For this study, the sequencing depth was at least 40.5× per biological replicate (for a 630 Mb genome size; Liu *et al*., 2014), which is sufficient coverage for differential expression analyses (Sims *et al*., 2014). 44%–47% reads mapped to the reference genome (36%–38% concordant read pairs, 3%–5% multiple alignment), generating 37 963 unique assembled transcripts in the merged reconstructed transcriptome (of 45 758 predicted protein‐coding genes in the reference genome). Next, we checked the completeness of our assembled *B. oleracea* transcriptome by comparing their sequences to a core set of embryophyta genes using BUSCO (Simão *et al*., 2015). The results revealed that 22.5% of BUSCO genes were “complete”, 17.3% “fragmented”, and the remaining 60.2% “missing” out of 1440 BUSCO genes. By this assessment, the quality and representability of our assembled transcriptome in relation with an expected comprehensive transcriptome were limited, probably due to the intrinsic limitations of this benchmark with species with complex polyploidy (Simão *et al*., 2015) and the senescent nature of the material, representing very specific tissue and conditions. TRAPID (Van Bel *et al*., 2013) provided an assessment of transcripts structure and completeness, by comparing transcript length to the lengths of its associated gene family. With an average sequence length of 1126 bases, 59.9% presented starting codons and 78.4% stop codons. 89% were ascribed to a gene family, with 37.1% of the transcripts classified by TRAPID as full‐length or quasi‐full‐length, and 50.3% as partial. 79.4% and 79.8% presented GO (Genome Ontology) functional annotation and identified protein domains, inferring function of most reconstructed transcripts. This indicates that the transcriptome is of sufficient quality to be used to quantify gene expression data from the four treatments.

**Figure 5:**
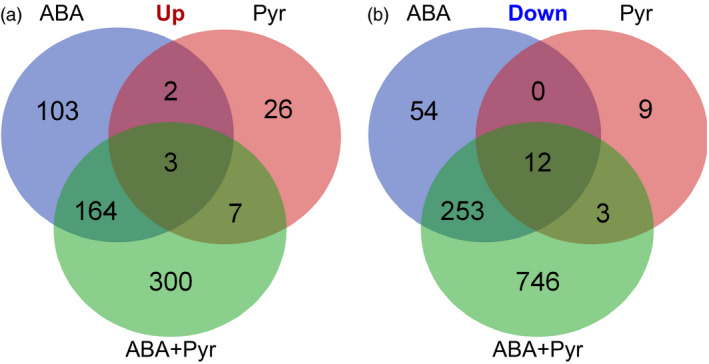
Number of differentially expressed transcripts between every two treatments and the number of joint differentially expressed transcripts. Up, up‐regulated transcripts; Down, down‐regulated transcripts.

Therefore, the aligned reads count and the reconstructed common transcriptome were used for differential expression analysis. There were 592, 62 and 1488 differentially expressed transcripts (ABA, Pyr and ABA+Pyr, respectively). Containing 1676 unique differentially expressed transcripts between ABA/Pyr and control conditions with a false discovery rate (FDR) <0.05 (Figure 5, Table S2). After blastx versus the Viridiplantae nr database, and GO mapping, GO enrichment analyses of differently up‐regulated and down‐regulated genes were performed to evaluate the impacts of ABA/Pyr treatment (Table S3).

### ABA metabolism and signalling responses to ABA/Pyr treatment

**Figure 6:**
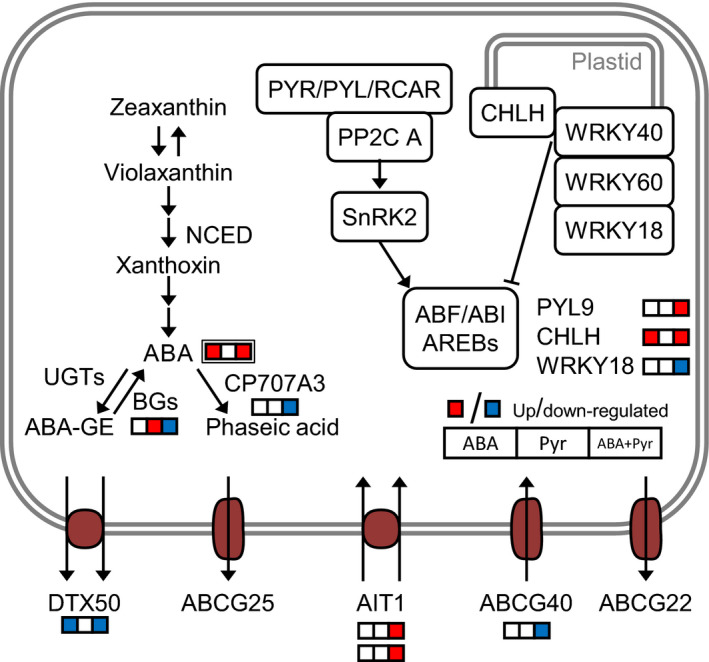
ABA metabolism and signalling. DE transcripts, single border; metabolites, double border. Proteins: ABF, ABA‐responsive elements‐binding factor; ABI, ABA‐insensitive; BG, beta‐1,3‐glucanase; CHLH, magnesium chelatase subunit H; NCED, 9‐*cis*‐epoxycarotenoid dioxygenase; PYL, PYR1‐*like*; PYR, pyrabactin resistance; RCAR, regulatory component of ABA receptor; SnRK, SNF1‐related protein kinase; UGTs, UDP‐glucosyltransferase; WRKY, WRKY DNA‐binding protein. Metabolites: ABA‐GE, ABA‐glucose ester conjugates.

Application of ABA every other day more than doubled endogenous ABA contents, either when ABA was applied alone or combined with pyrabactin (ABA+Pyr). We hypothesized that the increased ABA contents may affect ABA metabolism, transport and signalling. ABA and/or Pyr (ABA/Pyr) treatments represented no marked changes in the transcriptional regulation of ABA biosynthesis. However, ABA catabolism and ABA release from reversible conjugation were down‐regulated by ABA+Pyr (Figure 6). ABA transporters are a regulation point for the modulation of development and physiological ABA responses. Genes encoding those located in the cytosolic membrane were expressed (*ABCG22, ABCG25, ABCG40, DTX50, AIT1*), but not those in the vacuole (*ABCC1/MRP1, ABCC2/MRP2*). The continuous rise of ABA contents, caused a transcriptional down‐regulation of transporters associated with ABA sensitivity (DTX50, ABCG40), while the vascular ABA importer AIT1/NRT1.2 was up‐regulated.

Two of the three putative ABA receptor families presented transcriptional changes after ABA/Pyr treatments. *PYR1* and *PYL1,5,6,7,8,9* transcripts were detected while *PYL9* was up‐regulated by ABA+Pyr. Downstream the core ABA signalling pathway, targets of SnRK2 kinases activated by ABA perception were also up‐regulated by ABA+Pyr, notably genes encoding bZIP transcription factors: *AREB1/ABF2* (ABA+Pyr up‐regulated promotor of ABA signal); and *AFP3* and *AFP4/TMAC2* (ABA+Pyr down‐regulated inhibitor of ABA signal). The potassium channel KAT1 (regulated by SnRK2) was transcriptionally down‐regulated by ABA+Pyr, like SYNTAXIN121 (SYR1/PEN1) that positions KAT1 in different compartments, thus regulating fast responses to ABA. The plastidic ABA signal pathway was also transcriptionally responsive to ABA/Pyr. While *CHLH* was up‐regulated after ABA and ABA+Pyr, *WRKY18*—modulating plastidic ABA signal transduction—was down‐regulated by ABA+Pyr. Meanwhile, many ABA‐responsive transcription factors involved in abiotic and biotic stress crosstalk were transcriptionally down‐regulated, but only by ABA+Pyr (*CBF4/DREB1D/ERF028, DREB19/ERF046, DREB2C, ABR1/ERF111, ERF1, MYB102, MYB108, AIB, CAF1b, ZFHD1*).

**Figure 7:**
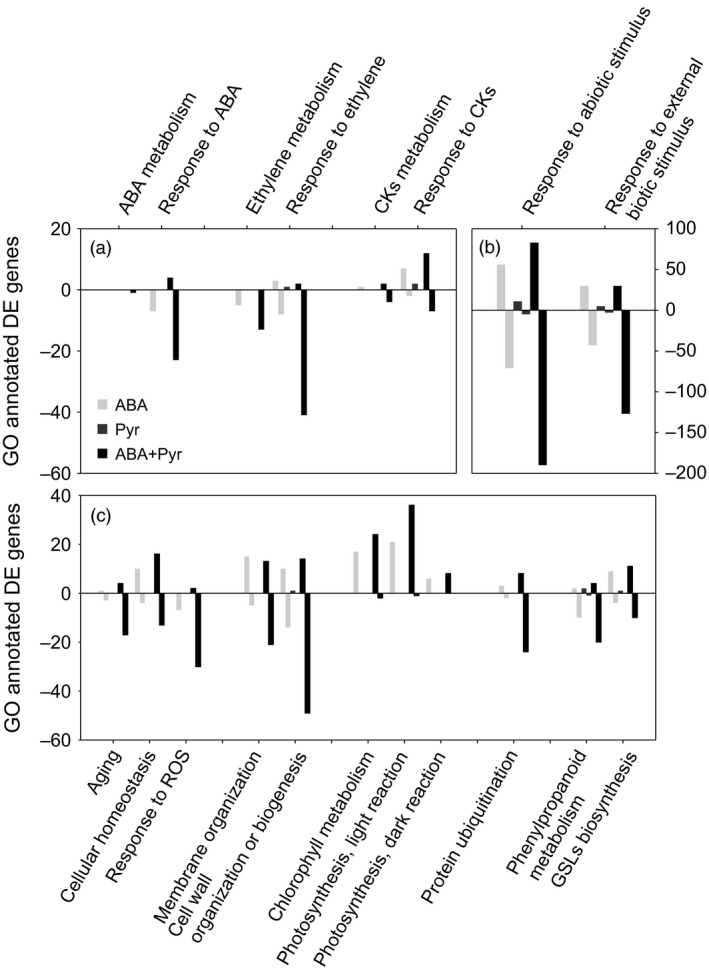
Differentially expressed transcripts annotated by certain GO terms. From left to right: (a) GO:0009687 abscisic acid metabolic process; GO:0071215 cellular response to abscisic acid stimulus; GO:0009692 ethylene metabolic process; GO:0009723 response to ethylene; GO:0009690 cytokinin metabolic process; GO:0009735 response to cytokinin. (b) GO:0009628 response to abiotic stimulus; GO:0043207 response to external biotic stimulus. (c) GO:0007568 aging; GO:0019725 cellular homeostasis; GO:0000302 response to reactive oxygen species; GO:0061024 membrane organization; GO:0071554 cell wall organization or biogenesis; GO:0015994 chlorophyll metabolic process; GO:0019684 photosynthesis, light reaction; GO:0019685 photosynthesis, dark reaction; GO:0016567 protein ubiquitination; GO:0009698 phenylpropanoid metabolic process; GO:0019761 glucosinolate biosynthetic process.

ABA/Pyr did not affect the transcriptional regulation of ABA synthesis, while down‐regulating its catabolism. Thus, the continuous rise of ABA contents coincided with a down‐regulation of ABA transporters. ABA receptors *PYL9* and *CHLH* were up‐regulated by ABA or ABA+Pyr, while a number of ABA‐responsive transcription factors and effectors were deregulated only by ABA+Pyr (Figure 7a).

### Ethylene metabolism and response

**Figure 8:**
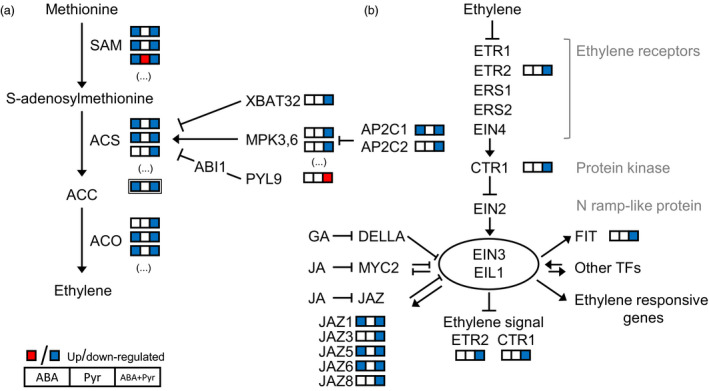
(a) Ethylene metabolism and (b) signalling. Proteins: ABI1, ABSCISIC ACID‐INSENSITIVE1; ACO, ACC oxidase; ACS, ACC synthase; AP2C, probable protein phosphatase 2C; CTR, constitutive triple response; EIL, EIN3‐*like*; EIN, ethylene‐insensitive; ETR/ERS, ethylene response; FIT, FER‐LIKE IRON DEFICIENCY‐INDUCED TRANSCRIPTION FACTOR; JAZ, jasmonate ZIM domain‐containing protein; MPK, mitogen‐activated protein kinase; PYL9, PYR1‐*like* 9; SAM,* S*‐adenosylmethionine synthetase; XBAT32, E3 ubiquitin‐protein ligase XBAT32.

The treatment with ABA and ABA+Pyr, and the consequent rise in ABA contents resulted in major changes to ethylene metabolism and its regulation. After 8 days, ACC (immediate precursor of ethylene) contents in Pyr‐treated leaves remained at the same level of the control, but were halved after ABA or ABA+Pyr treatment. In plants, ethylene is synthesized from methionine through the intermediates S‐adenosylmethionine (SAM) and 1‐aminocyclopropane‐1‐carboxylate (ACC) by the successive action of SAM synthetases, ACC synthases and ACC oxidases (Wang *et al*., 2002). After 8 days, genes encoding SAM synthetases (*MTO3, MAT3*) were down‐regulated by ABA and ABA+Pyr, but up‐regulated by Pyr (Figure 8a). In the next step of ethylene synthesis, putative ACC synthase transcripts (*ACS2,6*,*7*) were also down‐regulated by ABA and ABA+Pyr. As a key regulator of ACC and ethylene levels, ACS activity is strictly regulated. Different activation and inhibition elements were transcriptionally affected by ABA/Pyr treatment (Figure 8a). Finally, homologous to *Arabidopsis ACO1,2* were down‐regulated by ABA and ABA+Pyr.

The manipulation of ABA contents and signalling also represented changes in ethylene response pathways (Figure 8b). The ethylene receptor ETR2 and the protein kinase CTR1—negative regulators of ethylene signal transduction—were transcriptionally down‐regulated by ABA+Pyr. Downstream, transcription factors EIN3 and EIL1 promote the expression of ethylene‐responsive genes and are modulated by other regulators and transcription factors. First, EIN3/EIL1 modulate ethylene signalling by targeting CTR1 and ETR2 in a negative feedback loop and we found that both were transcriptionally down‐regulated by ABA+Pyr. Second, EIN3/EIL1 interact with other hormonal pathways as a point of coregulation, as revealed by a recent ChiP‐seq analysis (Chang *et al*., 2013). This is the case for the jasmonate‐responsive repressors JAZ and many *JAZ* homologues were transcriptionally down‐regulated by ABA+Pyr, or by both ABA and ABA+Pyr. Targets of EIN3/EIL1, like many ethylene‐responsive transcription factors (ERFs), were transcriptionally down‐regulated by ABA+Pyr treatment. Notably, between the transcriptionally regulated *ERFs* were genes encoding transcription factors involved in the regulation of stress signal pathways and redox homeostasis (*ERF1, ERF2, ERF072/RAP2.3, ERF113/RAP2.6L*), but also regulators of hormonal biosynthesis and responses, such as ABA (*ERF028/CBF4/DREB1D, ERF111/ABR1, ERF113/RAP2.6L*), CKs (*ERF066/CRF4*), gibberellins (*ERF032/DDF1*), and jasmonates and auxins (*ERF109*).

In summary, the manipulation of ABA contents and signalling represented changes in the regulation of ethylene biosynthesis, perception, signalling and responses (Figure 7a). Ethylene biosynthesis genes and elements required for its perception and response were generally down‐regulated by ABA and ABA+Pyr, while limited effects of Pyr alone were observed.

### Cytokinins metabolism and response

**Figure 9:**
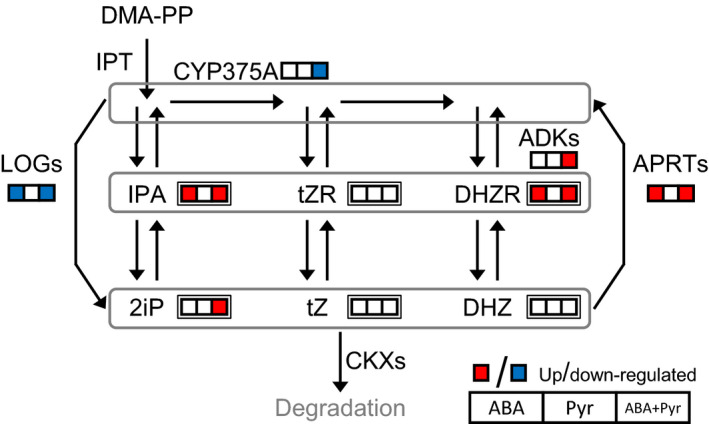
Cytokinins metabolism. Transcripts, single border; metabolites, double border. Proteins: ADKs, adenylate kinase; APRT, adenine phosphoribosyltransferase; IPT, isopentenyltransferase; LOG, LONELY GUY; CKX, cytokinin oxidase/dehydrogenase. Metabolites: DHZR, dihydrozeatin riboside; DHZ, dihydrozeatin; DMA‐PP, dimethylallyl pyrophosphate; 2iP, isopentenyladenine; IPA, isopentenyladenosine; *t*ZR, *trans*‐zeatin riboside; *t*Z,* trans*‐zeatin.

ABA/Pyr application caused changes in CKs profile; however, transcriptional regulation of genes encoding CKs biosynthesis or catabolism was not observed (Figure 9). Nevertheless, the effects of ABA/Pyr on cytokinin nucleosides IPA and DHZR, and the free base 2iP correlated with regulation of CKs metabolism. Genes encoding adenine phosphoribosyl transferases (APRTs) and adenosine kinases (ADKs)—generating CKs nucleotides from different CK forms—were up‐regulated by ABA and ABA+Pyr, while genes encoding antagonistic LOG7 isoforms, producing 2iP, Z and DHZ, were down‐regulated by ABA and ABA+Pyr. The cytokinin hydroxylase‐encoding gene *CYP735A2* which transforms iP‐type to Z‐type CKs was down‐regulated by ABA+Pyr. Meanwhile, the positive regulator of cytokinin contents *SOFL1* was up‐regulated after ABA+Pyr treatment. No transcriptional regulation of CKs signal pathway was observed, while CKs‐responsive elements were generally up‐regulated (Figure 7a). Yet, the negative regulator of CKs signalling ubiquitin ligase *KISS ME DEADLY 3* (*KMD3*), targeting type B ARRs, was up‐regulated by Pyr and ABA+Pyr.

Thus, ABA/Pyr treatment transcriptionally modified CK interconversion and the associated individual CKs contents. Moreover, it should be noted that genes encoding many enzymatic activities responsible of CKs interconversion have not been identified yet (Zhang *et al*., 2013), limiting the predictive value of the gene expression data.

### ABA signalling manipulation affects hallmarks of senescence

ABA/Pyr treatments altered key senescence hormones levels (ABA, CKs, ACC/ethylene) and the transcriptional regulation of the underlying signal and response pathways, suggesting a delay in leaf senescence. Hence, we examined several traits associated with the onset of senescence and its progression (Figure 7c).

Regulators of senescence—in the crosstalk between redox homeostasis and senescence progression—were transcriptionally down‐regulated by ABA+Pyr, notably the negative regulators of senescence *VNI2/ANAC083* and *JUNGBRUNNEN1* (*JUB1/ANAC042*) and the positive regulator *ATAF2/ANAC081*. Meanwhile*, GOLDEN2‐LIKE2* (*GLK2*), a major negative regulator of senescence, was transcriptionally up‐regulated by ABA and ABA+Pyr.

ABA and ABA+Pyr, but not Pyr, transcriptionally up‐regulated photosynthesis positive transcriptional regulators (GLK2), but also regulators of the photosynthesis machinery and metabolic flux [RUBISCO ACTIVASE (RCA), FRUCTOSE‐BISPHOSPHATE ALDOLASE1,2 (FBA1,2), SEDOHEPTULOSE‐1,7‐BISPHOSPHATASE (SBPase)], thus maintaining chloroplast function and allowing its adaptation to environmental and redox changes. In addition to *GLK2* transcriptional up‐regulation, we observed the subsequent up‐regulation of genes involved in chloroplast function maintenance. Other positive plastid regulators were also transcriptionally up‐regulated, including PLASTID TRANSCRIPTIONALLY ACTIVE6,16 (PTAC6,16) and CHLOROPLAST SENSOR KINASE (CSK). Furthermore, numerous components of the plastidic translation machinery were up‐regulated.

Genes involved in the maintenance of photosynthesis machinery were also up‐regulated (Figure 7c): from chlorophyll biosynthesis to many elements of the photosynthetic membrane (including subunits of photosystems I and II, light‐harvesting complexes, ATP synthase, plastocyanin and NAD(P)H dehydrogenases). Calvin cycle enzymes were also transcriptionally up‐regulated, notably FBA1,2, PHOSPHORIBULOKINASE (PRK), GLYCERALDEHYDE 3‐PHOSPHATE DEHYDROGENASE A (GAPA), FRUCTOSE‐1,6‐BISPHOSPHATASE (FBP) and SBPase. These included elements required for redox regulation, but other genes associated with redox homeostasis and response to redox stress were also affected (Figure 7c), including the transcriptional regulation of a number of reactive oxygen species detoxification enzymes, notably ascorbate and glutathione peroxidases (Table S4). *ROTAMASE CYP4* (*ROC4*), required for thiol accumulation for the build‐up of cellular reduction potential, was transcriptionally up‐regulated by ABA and ABA+Pyr.

Cell wall and membrane biogenesis and remodelling are essential for cell homeostasis. The biosynthesis of precursors of primary and secondary cell wall polymers and its assembly and remodelling pathways were transcriptionally regulated by ABA and ABA+Pyr (Figure 7c, Table S3). General phenylpropanoids biosynthesis, transcriptionally down‐regulated by ABA and ABA+Pyr, provides precursors for lignin, lignans, cutin and suberin. Downstream, the biosynthesis of lignin and lignans precursors was also transcriptionally down‐regulated by ABA and ABA+Pyr. On the other hand, many genes involved in membrane and cell wall remodelling were up‐regulated by ABA/Pyr, notably glycosyl hydrolases, xyloglucan transglycosylases and cell wall modification regulators such as expansins, peroxidases, subtilisins and *WALLS ARE THIN1*.

ABA/Pyr treatments promoted changes in the contents of key senescence hormones (ABA, CKs, ACC/ethylene) and in the regulation of underlying signal and response pathways, suggesting a delay in leaf senescence. We found transcriptional changes of genes involved in chloroplast function maintenance, redox homeostasis, membrane and cell wall homeostasis; as well as major regulators of senescence. Hence, we observed physiological and transcriptional changes consistent with a delayed onset of senescence.

### Post‐translational regulation through ubiquitin ligases

RNA‐seq data provide high‐throughput and systematic information about transcriptional regulation, but in addition can give insights into another major regulatory point: the post‐translational regulation by ubiquitin ligases‐targeted proteasomal degradation. For example, many proteins involved in ABA signalling and response are subject to regulation by ubiquitination (Yang *et al*., 2017). The ubiquitin ligases AIP2 and RGLG1 were transcriptionally down‐regulated by ABA+Pyr; AIP2 is a negative regulator of ABA response, while RGLG1 is a positive regulator of ABA signalling, targeting ABA‐dependent degradation of PP2CA (Wu *et al*., 2016). RDUF1 and 2, positive regulators of ABA responses with not yet identified targets, were transcriptionally down‐regulated by ABA or ABA+Pyr. Contrarily, RGLG2, transcriptionally down‐regulated by ABA+Pyr, is a crosstalk point between growth and stress responses, while modulating hormonal metabolism and response (Cheng *et al*., 2012). Both the ubiquitin ligase PUB23 and a proteasome regulatory subunit (RPN1A)—required for stress responses and innate immunity in a reportedly ABA‐independent way (Seo *et al*., 2012; Yao *et al*., 2012)—were transcriptionally down‐regulated by ABA+Pyr. Meanwhile, transcriptionally up‐regulated by Pyr and ABA+Pyr, KISS ME DEADLY3 (KMD3) targets phenylpropanoids biosynthetic enzymes (Zhang *et al*., 2015) and type B ARR cytokinins signal elements (Kim *et al*., 2013). The results therefore suggest that ABA/Pyr treatments cause changes in protein contents through altering ubiquitination.

### Biotic resistance trade‐offs

Neither jasmonic acid (JA) nor salicylic acid (SA) contents were affected after ABA/Pyr treatments for 8 days. But JA and SA metabolism and response pathways presented transcriptional regulation in response to ABA/Pyr. *l9S‐* and *13S‐LIPOXYGENASE* isoforms (*LOX3,4,5*) involved in oxylipins metabolism (including jasmonates) were down‐regulated by all ABA/Pyr applications. Other steps of JA metabolism were also transcriptionally down‐regulated by ABA and/or ABA+Pyr, but not by Pyr alone. Genes encoding JA signal and response elements were down‐regulated by ABA and ABA+Pyr, particularly several JAZ isoforms. Salicylates biosynthesis was also transcriptionally down‐regulated with two isoforms of *PHENYLALANINE AMMONIA‐LYASE1* (*PAL1*) 10‐times less expressed after ABA and ABA+Pyr treatment. At the same time, *KMD3*, targeting PAL for proteasomal degradation, was transcriptionally up‐regulated by Pyr and ABA+Pyr. Genes encoding salicylate *O*‐methyltransferases responsible of methyl‐jasmonate formation were down‐regulated by ABA and ABA+Pyr, while acetone‐cyanohydrin lyases involved in salicylic hydrolysis were transcriptionally up‐regulated. Genes encoding regulators of salicylic acid‐related defence responses were down‐regulated by ABA and/or ABA+Pyr, but not by Pyr. The biosynthesis of defence phenylpropanoids (phytoalexins) was also transcriptionally down‐regulated by ABA and ABA+Pyr. Generally, many effectors and regulators required for defence responses were transcriptionally down‐regulated by ABA and ABA+Pyr (Figure 7b, Table S4).

### Impact on glucosinolates and defence phenylpropanoids signatures

**Figure 10:**
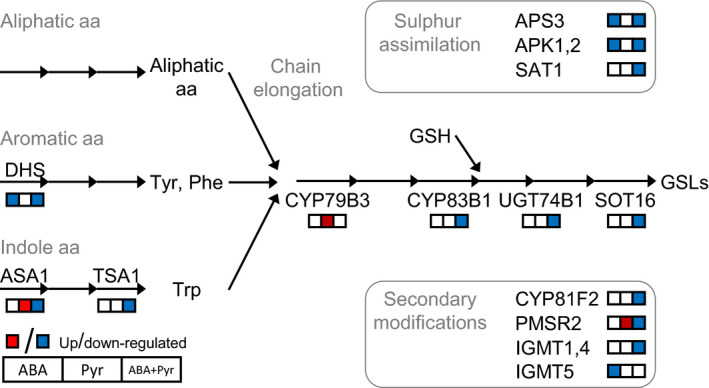
Glucosinolates (GSLs) metabolism. Metabolites: aa, amino acid; GSH, glutathione; Phe, phenylalanine; Trp, tryptophan; Tyr, tyrosine. Proteins: APK1,2, APS‐kinase; APS3, ATP‐sulfurylase3; ASA1, anthranilate synthase1; DHS, DAHP synthase1; IGMT, Indole glucosinolate *O*‐methyltransferase; PMSR2, peptidemethionine sulfoxide reductase2; SAT1, serine *O*‐acetyltransferase; SOT16, sulfotransferase16; TSA1, tryptophan synthase1; UGT, UDP‐glucosyltransferase.

Glucosinolates (GSLs) are sulphur‐rich secondary metabolites divided according to their amino acid precursors: aliphatic GSLs (derived from methionine, alanine, valine, leucine or isoleucine), aromatic GSLs (from phenylalanine or tyrosine) and indole GSLs (from tryptophan). With these starting substrates, GSLs biosynthesis comprises three independent steps: chain elongation, formation of the core GSL structure, and secondary modifications (Figure 10).

First, sulphur assimilation key enzymes (ATP‐SULFURYLASE3, ADENYLYL‐SULFATE KINASE1,2, SERINE ACETYLTRANSFERASE2‐1) were transcriptionally down‐regulated by ABA and ABA+Pyr; while the sulphur metabolism activator *ROC4* was up‐regulated by the same treatments. The biosynthesis of multiple amino acid precursors was also deregulated. Aromatic amino acids biosynthesis—precursors of aromatic GSLs and phenylpropanoids—was transcriptionally down‐regulated by ABA and ABA+Pyr, including the first committed step *(2‐DEOXY‐D‐ARABINO‐HEPTULOSONATE 7‐PHOSPHATE SYNTHASE, DHS)*. The limiting step for tryptophan synthesis, precursor of auxins, melatonin and indole GSLs, ANTHRANILATE SYNTHASE1 (ASA1) was transcriptionally up‐regulated by Pyr but down‐regulated by ABA+Pyr. Downstream, tryptophan synthase was transcriptionally down‐regulated by ABA.

Genes encoding all described steps of the GSL core formation were down‐regulated by ABA+Pyr: *CYP79B3*,* CYP83B*1, *UDP‐GLUCOSYLTRANSFERASE 74B1*, and several *SULFOTRANSFERASE16* homologues. *CYP79B3* was uniquely up‐regulated by Pyr. Further, genes involved in GSLs modification pathways were down‐regulated by ABA and ABA+Pyr, encoding a number of indole glucosinolate O‐methyltransferases, CYP81F2 and PEPTIDEMETHIONINE SULFOXIDE REDUCTASE2. Meanwhile, Pyr up‐regulated and ABA+Pyr down‐regulated *CYP71A12*, involved in the degradation of indole GSLs but also in the synthesis of the defence phenylpropanoid camalexin.

Phenylpropanoids are a family of metabolites with the aromatic amino acids phenylalanine and tyrosine as precursors, contributing to responses to abiotic stress and defence responses. ABA, and further ABA+Pyr, transcriptionally down‐regulated the biosynthesis of these precursor amino acids (discussed above), but relevant downstream enzymes were also deregulated. ABA and ABA+Pyr down‐regulated the expression of isoforms of all the steps of the general phenylpropanoids pathway (*PHENYLALANINE AMMONIA‐LYASE, CINNAMATE‐4‐HYDROXYLASE, 4‐CUMARATE‐COA‐LIGASE*), generating *P*‐coumaroyl‐CoA. This is a shared precursor of flavonoids, anthocyanins and other soluble phenylpropanoids, but also of cell wall polymers (lignin, lignans. cutin and suberin). Genes encoding enzymes involved in the biosynthesis of lignin, lignans, and anthocyanins were also down‐regulated by ABA and ABA+Pyr. Meanwhile, a number of benzoyltransferases, aminotransferases and γ‐glutamyl peptidases required for defence phenylpropanoids (phytoalexins) biosynthesis were also transcriptionally down‐regulated by ABA and ABA+Pyr. Finally, transcriptionally up‐regulated by Pyr and ABA+Pyr, the ubiquitin ligase KMD3 targets multiple phenylpropanoids biosynthetic enzymes, negatively regulating these pathways.

ABA/Pyr treatment caused transcriptional deregulation of genes involved in the metabolism of GSLs, phenylpropanoids and precursor amino acids, likely changing its metabolic signatures. ABA and further ABA+Pyr transcriptionally down‐regulated sulphur assimilation, aromatic and indole amino acids biosynthesis, the GSLs core formation, several enzymes involved in GSLs secondary modifications and phenylpropanoids biosynthesis. Meanwhile, Pyr uniquely up‐regulated steps of the biosynthesis of indole amino acids, and steps of the GSLs core formation and side‐chain conversion of aliphatic GSLs. Thus, ABA and/or Pyr treatment generated unique expression signatures of genes involved biosynthesis of GSLs and phenylpropanoids.

## Discussion

### ABA signal modification suppresses senescence

We observed how the application of ABA, but not Pyr, impacted ABA signalling, up‐regulating *PYL9* expression, reducing water loss and keeping membrane integrity after 8 days at room temperature storage conditions (summarized in a proposed model of ABA/PYL application senescence suppression in Figure S11). Further, we observed effects on the metabolism, signalling and actual contents of cytokinins and ACC/ethylene, two sets of phytohormones involved in senescence regulation. At the same time, ABA/Pyr treatments manipulated the expression of major senescence regulators and did not promote senescence, but stimulated redox homeostasis, chloroplast function and cell wall remodelling. PYL9/RCAR1 has recently been related to leaf senescence regulation. ABA acts through PYL9 to limit transpirational water loss in young leaves, while promoting leaf senescence in an ethylene‐independent manner through SnRK2s (Zhao *et al*., 2016). Further, an *Arabidopsis PYL9* overexpressor showed reduced electrolyte leakage (Zhao *et al*., 2016).

Two major negative regulators of senescence were transcriptionally down‐regulated by ABA+Pyr, *VNI2/ANAC083* and *JUNGBRUNNEN1* (*JUB1/ANAC042*). These two genes reportedly integrate ABA signalling and stress responses to leaf senescence progression (Wu *et al*., 2012; Yang *et al*., 2011). Otherwise, positive regulators of senescence were ABA+Pyr down‐regulated, like *ATAF2/ANAC081,* a stress‐responsive transcription factor involved in ABA‐inducible leaf senescence but also in the regulation of basal defence responses (Delessert *et al*., 2005; Takasaki *et al*., 2015). At the same time, the negative regulator of senescence *GOLDEN2‐LIKE2* (*GLK2*) was transcriptionally up‐regulated by both ABA and ABA+Pyr. *GLK2* is a key nuclear transcription factor that regulates chloroplast development (Waters *et al*., 2008) and is a positive regulator of the photosynthetic apparatus—including chlorophyll biosynthesis and light and dark photosynthesis reactions (Waters *et al*., 2009)—integrating responses to the environment and endogenous cues to keep redox homeostasis while preventing senescence (Rauf *et al*., 2013). Indeed, ABA and ABA+Pyr promoted genes involved in chloroplast function, up‐regulating plastid translation machinery, chlorophyll biosynthesis, and components of the photosynthetic membrane and Calvin cycle.

Enzymes controlling metabolic fluxes, and thus chloroplast function, were also induced by ABA and ABA+Pyr. A good example is chloroplastic fructose‐bisphosphate aldolases (FBAs), regulating fluxes in many central metabolism pathways. Changes in FBA activity are required for photosynthesis acclimation in response to abiotic stressors (Lu *et al*., 2012). Further, FBAs are phosphorylated in response to ABA, diminishing its abundance (Ghelis *et al*., 2008).

The discussed positive regulation of photosynthetic machinery may provide enhanced redox homeostasis, which may impact leaf senescence as a result of an increased capability to cope with redox changes. Further, *ROC4* was also transcriptionally up‐regulated and is required for thiol accumulation for the regulation of stress‐responsive redox homeostasis and build‐up of cellular reduction potential (Park *et al*., 2013). Genes encoding ABA‐responsive effectors relevant for cellular homeostasis—for example *GLUTATHIONE PEROXIDASE 7* (Chang *et al*., 2009)*, TOUCH 2* (Delk, 2005) and *CPK32* (Choi *et al*., 2005)—were also up‐regulated by ABA+Pyr. Redox signalling is essential for the adaptation of metabolism in response to environmental and endogenous cues to maintain cellular function. Further, ABA and ABA+Pyr up‐regulated the expression of *CHLOROPLAST SENSOR KINASE* (*CSK*), encoding a regulator of chloroplast transcription responsive to plastoquinone redox state (Puthiyaveetil *et al*., 2008).

ABA and ABA+Pyr up‐regulated the expression of *MAGNESIUM CHELATASE SUBUNIT H* (*CHLH*) and *GENOMES UNCOUPLED 4* (*GUN4*), necessary for coupling the expression of nuclear genes to the functional state of the chloroplast (Pfannschmidt, 2010). However, other retrograde signalling elements were transcriptionally down‐regulated by ABA+Pyr, like *ROC4* and the chloroplastic oxygen singlet‐responsive AAA‐ATPase (Simkova *et al*., 2012). Outside the chloroplast, the mitochondrial alternative oxidase, involved in redox homeostasis and retrograde signalling (Konert *et al*., 2015), was transcriptionally down‐regulated by ABA+Pyr. Nevertheless, the results are consistent with observations that nuclear transcription is coordinated with the functional state of the chloroplast through retrograde signalling mechanisms to maintain redox homeostasis in a changing environment.

ABA deregulated major senescence and cell homeostasis regulators, suppressing senescence and maintaining chloroplast functionality in challenging storage conditions. In contrast, Pyrabactin application caused limited effects on hormonal levels and transcriptional regulation of genes involved in hormone metabolism and cell homeostasis regulators. A similar limited impact was found on physiological parameters after 8 days of room temperature storage. The joint treatment with both ABA and Pyr at the same concentration generally further promoted the effects shown by ABA only treatment.

### ABA/Pyr decreases biotic stress resistance

The metabolism of salicylates and jasmonates, with its main role in defence against biotrophs and necrotrophs, is influenced by both biotic and abiotic stress factors (Xia *et al*., 2015) and can be affected by temperature (Miura and Tada, 2014). ABA has positive or negative effects on defence responses depending on the nature (necrotrophic or biotrophic) of the infection (Robert‐Seilaniantz *et al*., 2011) through enhanced redox buffering capacity and ABA signalling that affects signalling and defence mechanisms (Bartoli *et al*., 2013; Ton *et al*., 2009). ABA/Pyr treatment represented a significant transcriptional down‐regulation of JA and SA metabolism, and of effectors and modulators of responses to external biotic stimulus. This therefore suggests a reduction of the capacity of the leaf to resist biotic stress.

### ABA/Pyr may affect glucosinolates and phenylpropanoids profile

Glucosinolates accumulation is a desired trait associated with organoleptic and nutraceutical characteristics. Li *et al*. (2014) systematically identified transcription factors regulating glucosinolates profile. ABA signalling modification by treatments with ABA and/or Pyr affected the transcription of several of these, including transcription factors that also regulate hormonal balance and growth such as *DWARF AND DELAYED FLOWERING1* (*DDF1*; ABA down‐regulated, ABA+Pyr up‐regulated [Magome *et al*., 2004]), or associated with abiotic stress responses and down‐regulated by ABA+Pyr—*RELATED TO AP2.6L* (*RAP2.6L/ERF113;* Krishnaswamy *et al*., 2011; Liu *et al*., 2012) and *CBF4/DREB1D* (Haake *et al*., 2002).

In addition, many genes involved in GSLs biosynthesis *per se* where transcriptionally regulated. ABA/Pyr treatment down‐regulated sulphur assimilation, aromatic amino acids and defence compounds synthesis. Treatments containing only pyrabactin enhanced the expression of key tryptophan and indole glucosinolates biosynthetic genes, presenting a unique glucosinolates metabolism transcriptional profile, compared to ABA and ABA+Pyr treatments. GSLs contents are usually maximum at harvest but dependent on preharvest biotic and abiotic stressors (Velasco *et al*., 2007). GSLs content not only depends on preharvest contents and enzymatic activity, but *de novo* biosynthesis of GSLs is more likely to occur during postharvest storage (Villarreal‐García *et al*., 2016), and the manipulation of the environment including abiotic stressors and exogenous hormones has proven to modify GSLs content in fresh produce (Cisneros‐Zevallos, 2003; Jones *et al*., 2006). There are previous reports of effects of ABA treatment on young leafy *Brassica*, including increased GSLs content in 5‐day‐old cabbage sprouts (Wang *et al*., 2015), and in pak choi shoots (Hu and Zhu, 2013). Thus, in our study ABA/Pyr treatment potentially changed the cabbage glucosinolates profile and resulting organoleptic and nutraceutical traits.

## Conclusions

ABA plays a major role in stress responses. Leaf harvest, or detachment, followed by RT storage induces severe drought stress and senescence. Therefore, a role for ABA in regulating postharvest senescence is perhaps not surprising. Nevertheless, ABA's role in the regulation of senescence seems to depend on the age of the affected leaf (Jibran *et al*., 2013). While ABA stimulates senescence in old leaves, it may suppress senescence in young tissue (Lee *et al*., 2011; Zhang *et al*., 2012), suggesting that the ABA's role depends on leaves to acquire the competence to senescence. This is perhaps best illustrated by recent work done by Zhao *et al*. (2016), who showed that increased ABA signalling under severe drought stress accelerated senescence in older leaves, but induced drought resistance, and thus survival, in the younger leaves. We observed that ABA treatment through leaf postharvest storage at room temperature affected major senescence hallmarks, including maintenance of chloroplast function, increased turgor and membrane integrity. This then suggests that the green cabbage leaves did not yet acquire the competence to senesce at the start of the experiment and that the induced early rise on ABA contents promoted homeostasis responses resulting in the suppression of senescence. Perhaps as a trade‐off, ABA and pyrabactin application also down‐regulated biotic stress regulators, likely making the leaf more susceptible to disease. At the same time, the application caused major changes to phenylpropanoids and glucosinolates metabolism transcriptional regulation, probably changing phenylpropanoids and glucosinolates signatures. These results pave the way for manipulating ABA signalling for improving the postharvest quality of leafy vegetables stored at ambient temperature.

## Experimental procedures

### Plant material and experimental treatments

Green cabbage (*Brassica oleracea* L. var. *capitata* cv. ‘Clarissa’ belonging to the Savoy cabbage group) from the same origin (Orihuela, SE of Spain), selected for uniform size and colour, were purchased from a local supplier and transported to our laboratory at 4 °C, 75% relative humidity (RH). Exterior leaves (with a dark green apex) were detached with a sharp scalpel, washed with running water and gently dried with absorbent paper. Only healthy heads were selected, with no signs of damage by manipulation or stress.

In experiment I, the postharvest senescence of green cabbage leaves was studied under two storage conditions: cold (4 °C) and room temperature (RT, 25 °C). The leaves were stored in a cold room (4–5 °C, 75% ± 2% RH, darkness) for the cold treatment, or in a plant growth chamber (Sanyo MLR 350H, Japan) at 25 °C (RT), under the same conditions of humidity and darkness. In experiment II, the leaves were treated with ABA and/or pyrabactin, under the RT condition of experiment I. In experiment II, leaves were treated every other day with a uniform spray of different treatments over both sides of the leaf blade. Treatments with ABA 10^−5^ M, Pyr 10^−5^ M and ABA 10^−5^ M + Pyr 10^−5^ M were compared with a control treatment with the application solution, Milli‐Q water with 0.5% dimethyl sulphoxide 0.1% Tween‐20. Due to ABA light sensitivity, all manipulations were performed under minimal light. Unless stated otherwise, all chemicals were of HPLC grade or higher and purchased from Sigma‐Aldrich (Madrid, Spain).

As part of sampling, the leaf was transversally cut with a sharp scalpel with the help of a metallic rule in three strips by equal lengths of the baso‐apical axis: apical, medial and basal (Figure S1). A longitudinal cut was performed through the three strips for the cutting of leaf discs for turgor and ion leakage determinations. The different strips were immediately frozen in liquid nitrogen and stored at −80 °C until analysis. The strong midrib and major veins were discarded. All physiological and biochemical measures represent the average ± SE of five biological replicates.

### Physiological parameters

Relative water content (RWC) was determined as (FW−DW) × (TW−DW)^‐1^ and hydration as (FW−DW) × DW^−1^; where FW is fresh weight; TW, turgid weight after imbibition with distilled water for 24 h at 4 °C in darkness; and DW, dry weight after oven‐drying at 70 °C until constant weight was reached. Whole leaf biomass loss was measured as 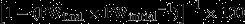, where FW_initial_ is the fresh weight at the start of the experiment and FW_final_ at sampling. Measurements of the maximum efficiency of photosystem II (*F*_v_/*F*_m_) were performed with a pulse‐modulated fluorimeter mini‐PAM (Walz, Germany). To assess membrane integrity, three discs of each sample were imbibed in Milli‐Q water‐sealed tubes at 25 °C for 2 hours before a first measure of electric conductivity (EC_1_). A second measure (EC_2_)—to determine maximum leakage—was performed after 10 min at 100 °C. Membrane stability index (MSI, %) was calculated as 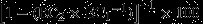 Lipid peroxidation was estimated by the thiobarbituric acid‐reactive substances (TBARS) assay, measuring the amount of malondialdehyde (MDA) after Hodges *et al*. (1999), taking into account the influence of interfering compounds.

### Hormonal profiling

The extraction and profiling of phytohormones by UPLC‐MSMS was performed as previously described (Müller and Munné‐Bosch, 2011). Quantification considered recovery rates for each sample using deuterated internal standards and authentic standards.

### Pigments, vitamin C and vitamin E determination

For the determination of pigments and vitamin E, *ca*. 50 mg of frozen tissue was ground and repeatedly extracted with ice‐cold methanol and ultrasonication. Chlorophyll and carotenoid contents were measured spectrophotometrically (Lichtenthaler and Wellburn, 1983). Vitamin E determination was carried out by HPLC‐DAD as described by Munné‐Bosch and Alegre (2003).

For vitamin C analyses*, ca*. 60 mg of tissue was ground and extracted with ice‐cold 6% (w/v) meta‐phosphoric acid 0.2 mm diethylene triamine pentaacetic acid using ultrasonication. Ascorbic acid and dehydroascorbate were analysed by the enzymatic assay as described by Queval and Noctor (2007).

### Soluble ions by ICP‐OES

Soluble cations (Ca^2+^, Mg^2+^, K^+^, Na^+^) were extracted from ground tissue with Milli‐Q water in an orbital shaker (150 rpm) for 1 h and determined by inductively coupled plasma optical emission spectrometry (ICP‐OES; Optima 3200RL, Perkin‐Elmer, CT).

### Statistical analyses of physiological and biochemical parameters

Analysis of variance ANOVA was performed to examine statistical differences across strips, treatments and time points (when applicable), using the Tukey‐*b* post hoc test. Differences were considered significant at a probability level of *P *≤* *0.05. All analyses were conducted using SPSS 20 (IBM, Armonk, NY, USA).

### Sequencing and differential expression analysis

RNA was extracted with the hot borate method (Wan and Wilkins, 1994) from three independent biological replicates of apical strip after 8 days of treatment with control solution, ABA and/or Pyr. RNA was quantified using a Nanodrop spectrophotometer (ND1000; Thermo Fisher Scientific, Waltham, MA) and RNA quality examined using a 2100 Bioanalyzer (Agilent Technologies, CA). High‐quality RNA samples for library construction were selected based on the 260/280 nm ratio and RNA integrity number above 2.1 and 6.5, respectively. New Zealand Genomics Limited (NZGL) RNA‐sequencing services (Dunedin, New Zealand) prepared Illumina libraries and sequenced on an Illumina HiSeq 2000 platform (100 bases paired‐end reads; Illumina, San Diego, CA). Quality control and rRNA content were assessed, and nondesired reads masked for further analyses with FastQC (Andrews, 2010) and riboPicker (rrnadb_2012‐01‐17 release; Schmieder *et al*. (2012)). No comprehensive reference transcriptome was available for *Brassica oleracea*. Recently published *B. oleracea* genome v1.0 (Liu *et al*., 2014) was used as reference for differential expression analysis with the Tuxedo suite (Trapnell *et al*., 2012). First, reads of each biological replicate were independently mapped to the reference genome with the splice‐aware aligner TopHat 2.0.9 (–b2‐very‐sensitive parameters set) and individually assembled transcripts were merged (Cufflinks 2.1.1 cuffmerge tool) to generate a unified transcriptome reconstruction for further analyses. Building a common transcriptome assembly as the basis for reads count and its normalization is a pertinent approach to accurately quantify expression levels reducing biases between conditions and samples (Trapnell *et al*., 2012). The completeness and quality of the assembled transcriptome was assessed with BUSCO and TRAPID (Simão *et al*., 2015; Van Bel *et al*., 2013). Differentially expressed transcripts between treatments were identified with cuffdiff 2.1.1 (default parameters plus—multi‐read‐correct and—frag‐bias‐correct, applying multiple biases corrections and thresholds to improve the accuracy of transcript abundance estimates; see Trapnell *et al*., 2012) at a false discovery rate (FDR) of 0.05.

### Annotation and GO enrichment analysis

The nonredundant (nr) Viridiplantae NCBI protein database was interrogated to blastx the assembled transcripts with blast+ (Camacho *et al*., 2009) with a cut‐off *E*‐value of 10^−5^. BLAST results imported to Blast2GO 4.0 (BioBam Bioinformatics, Valencia, Spain) were used for Gene Ontology (GO) mapping and annotation, enriched by interPro scan and Annex annotation augmentation (Conesa and Gotz, 2008; Finn *et al*., 2017). To identify overrepresented GO annotations in the differentially expressed gene sets, compared with the broader own reference assembly, GO enrichment analysis was performed with Ontologizer 2.0 implementation of the Parent‐Child‐Union method (Bauer *et al*., 2008)—taking into account the annotation of terms to the current term's parents, reducing false positives due to the inheritance problem—with Benjamini–Hochberg multiple test correction (Benjamini and Hochberg, 1995) at a FDR of 0.05.

## Supporting information


**Figure S1** Division of the leaf blade in three strips at sampling.
**Figures S2–S4** Physiological and biochemical parameters through experiment I.
**Figures S5–S8** Endogenous hormonal contents through experiment I.
**Figure S9** Antioxidant contents after application of ABA/Pyr (experiment II).
**Figure S10** Soluble ion contents after application of ABA/Pyr (experiment II).
**Figure S11** Proposed model of ABA/PYL application senescence suppression.

**Table S1** Quality control of RNA‐seq reads and assembly.

**Table S2** DE transcripts up‐regulated or down‐regulated per each treatment.

**Table S3** GO enrichment within the gene set up‐regulated or down‐regulated per each treatment.

**Table S4** List of DE transcripts annotated with the GO terms ‘GO:0043207 Response to external biotic stimulus’; ‘GO:0000302 ‘Response to reactive oxygen species’; and ‘GO:0016209 Antioxidant activity’.
