# Supplementary material for: ABA signalling manipulation suppresses senescence of a leafy vegetable stored at room temperature
Source: Plant Biotechnol J. 2017 Aug 16;16(2):530–44. doi: 10.1111/pbi.12793 (PMC5787841; doi:10.1111/pbi.12793)
Supplement: Supplementary file 1 — Figure S1 Division of the leaf blade in three strips at sampling. Figures S2–S4 Physiological and biochemical parameters through experiment I. Figures S5–S8 Endogenous hormonal contents through experiment I. Figure S9 Antioxidant contents after application of ABA/Pyr (experiment II). Figure S10 Soluble ion contents after application of ABA/Pyr (experiment II). Figure S11 Proposed model of ABA/PYL application senescence suppression. [file PBI-16-530-s004.docx]

Supporting information

Figure S1. Division of the leaf blade in three strips at sampling.

Figure S2-4. Physiological and biochemical parameters through experiment I.

Figure S5-8. Endogenous hormonal contents through experiment I.

Figure S9. Antioxidant contents after application of ABA/Pyr (experiment II).

Figure S10. Soluble ions contents after application of ABA/Pyr (experiment II).

Figure S11. Proposed model of ABA/PYL application senescence suppression.

Table S1. Quality control of RNA-seq reads and assembly.

Table S2. DE transcripts up-regulated or down-regulated per each treatment.

Table S3. GO enrichment within the gene set up-regulated or down-regulated per each treatment.

Table S4. List of DE transcripts annotated with the GO term “GO:0043207 Response to external biotic stimulus”.

Physiological and biochemical analyses of experiment I and II

Figure S1. Division of the leaf blade in three strips at sampling. Most of the material was immediately frozen in liquid nitrogen. After freezing the material for biochemical and RNA-seq analyses, disks were cut from the reminding material.

Figure S2. Whole leaf biomass loss at experiment I.

Figure S3. Pigments contents and ratio at experiment I. Chl, total chlorophyll; Chl a/b, ratio between chlorophyll *a* and chlorophyll *b* (g/g); Car, total carotenoids.

Figure S4. Relative Water Content (RWC) and maximum photosystem II efficiency (*F*_v_/*F*_m_) through experiment I.

Figure S5. Endogenous hormonal contents through experiment I. ABA, abscisic acid; JA, jasmonic acid; SA, salicylic acid; ACC, 1-aminocyclopropane-1-carboxylic acid.

Figure S6. Endogenous hormonal contents through experiment I. Total cytokinins (CK total), *trans*-zeatin (*t*Z), *trans*-zeatin riboside (*t*ZR), and isopentenyladenosine (IPA).

Figure S7. Endogenous hormonal contents through experiment I. Indolacetic acid (IAA), dehydrozeatin (DHZ), dehydrozeatin riboside (DHZR), *trans*-zeatin riboside (*t*ZR), and 2-isopentenyladenine (2iP).

Figure S8. Vitamin E contents through experiment I. α-Toc, α-tocopherol; γ-Toc, γ-tocopherol; Total toc, total tocopherol (sum of the two detected tocopherols, α- and γ-tocopherol).

Figure S9. Antioxidant contents in the apical strip after 8 days of every other day application of ABA/Pyr (experiment II). Total Car, total carotenoids; α-Toc, α-tocopherol; AA, ascorbic acid; redox state, vitamin C redox state.

Figure S10. Soluble ion contents in the apical strip after 8 days of every other day application of ABA/Pyr (experiment II). Quantified by ICP-OES.

Figure S11.Proposed model of ABA/PYL application senescence suppression in detached *Brassica oleracea* var. *capitata* leaves. Continued application of ABA and its partial agonist Pyrabactin for 8 days rise ACC levels and change CKs profile. Major changes in the transcriptional and post-transcriptional regulation of hormonal metabolism and signaling were caused by the treatment: ABA, ACC/ethylene and CKs metabolism and signaling were affected. Ethylene signaling is a senescence promoter, while CKs are senescence inhibitors. These changes represented a major transcriptional deregulation of responses to abiotic and biotic stresses, including the metabolism of glucosinolates and phenylpropanoids with a role in the response to both kinds of stresses. ABA/Pyr application directly or indirectly represents an impact on physiological parameters and its underlying transcriptional regulation, presenting a senescence suppression effect. Including the protection of cell homeostasis, membrane integrity, and chloroplast functionality.
