# Supplementary material for: ABA signalling manipulation suppresses senescence of a leafy vegetable stored at room temperature
Source: Plant Biotechnol J. 2017 Aug 16;16(2):530–44. doi: 10.1111/pbi.12793 (PMC5787841; doi:10.1111/pbi.12793)
Supplement: Supplementary file 2 — Table S1 Quality control of RNA‐seq reads and assembly. [file PBI-16-530-s003.docx]

Table S1. Quality control of RNA-seq reads and assembly. rRNA mapping reads (%), rate of rRNA mapping reads per ribopicker; Q20, reads with an average Phred score above 20; High quality reads, reads used for assembly and differential expression after masking rRNA and low quality reads (average <20 Phred score); Genome mapping (%), ratio of reads mapped to *B. oleracea* reference genome; Conconrdant pair aligment (%), ratio of aligned concordant paired reads; Multiple aligments (%), ratio of reads aligned to multiple locus.

| Condition | Biological replicate | Total raw reads | rRNA mapping reads (%) | Q20 (%) | High quality reads | Genome mapping (%) | Conconrdant pair aligment (%) | Multiple aligments (%) |
| --- | --- | --- | --- | --- | --- | --- | --- | --- |
| Control | 1 | 30212286 | 0.50 | 99.42 | 29884978 | 45.7 | 36.3 | 4.0 |
|  | 2 | 28831788 | 0.34 | 99.43 | 28570074 | 47.3 | 38.4 | 3.2 |
|  | 3 | 25248168 | 0.34 | 99.39 | 25009044 | 46.6 | 37.8 | 3.4 |
| ABA | 1 | 25840650 | 0.48 | 99.32 | 25541102 | 45.3 | 36.7 | 4.2 |
|  | 2 | 24461962 | 0.24 | 99.35 | 24245404 | 45.0 | 36.2 | 3.3 |
|  | 3 | 25634420 | 0.79 | 99.41 | 25282650 | 44.2 | 36.1 | 4.3 |
| Pyr | 1 | 23192794 | 0.42 | 99.41 | 22960210 | 45.6 | 36.4 | 3.8 |
|  | 2 | 28479392 | 0.33 | 99.35 | 28202322 | 46.1 | 37.8 | 3.1 |
|  | 3 | 21770736 | 0.31 | 99.35 | 21563118 | 46.5 | 37.9 | 3.5 |
| ABA+Pyr | 1 | 22819486 | 0.52 | 98.79 | 22426164 | 45.5 | 36.4 | 5.5 |
|  | 2 | 26100180 | 0.40 | 99.40 | 25839476 | 45.0 | 35.8 | 3.8 |
|  | 3 | 23608536 | 0.29 | 99.29 | 23372984 | 45.2 | 35.8 | 3.5 |
