# Supplementary material for: ABA signalling manipulation suppresses senescence of a leafy vegetable stored at room temperature
Source: Plant Biotechnol J. 2017 Aug 16;16(2):530–44. doi: 10.1111/pbi.12793 (PMC5787841; doi:10.1111/pbi.12793)
Supplement: Supplementary file 3 — Table S2 DE transcripts up‐regulated or down‐regulated per each treatment. [file PBI-16-530-s002.doc]

## S2 DE transcripts upregulated or downregulated per each treatment

## ABA-up

Table S2.1. Upregulated gene products after 8 days of ABA treatment. Adjusted p-value <0.05. Gene name, as annotated by Blast2GO after best blastx in nr Viridiplantae database. Best Swiss-Prot hit (Uniprot manually annotated and reviewed protein database) as *Arabidopsis thaliana* locus name or Uniprot “Entry|Entry name” if best curated hit is from another species. ABA, Pyr and ABA+Pyr log2 fold change to control condition. Locus, *Brassica oleracea* v1.0 genomic locus (Liu et al. 2014).

| Isoform id | Gene name | Best Swiss-Prot hit | ABA | Pyr | ABA+Pyr | Locus |
| --- | --- | --- | --- | --- | --- | --- |
| TCONS_00004290 | -NA- | -- | Inf | 0 | Inf | C02:1..44046003:34492174-34494714 |
| TCONS_00005704 | -NA- | -- | Inf | Inf | Inf | C02:1..44046003:12630531-12630921 |
| TCONS_00015949 | -NA- | -- | Inf | Inf | Inf | C04:1..40895475:25686955-25687444 |
| TCONS_00015950 | -NA- | -- | Inf | Inf | Inf | C04:1..40895475:25687793-25687931 |
| TCONS_00017325 | -NA- | -- | Inf | 0 | Inf | C04:1..40895475:39422098-39422913 |
| TCONS_00025298 | adenine phosphoribosyltransferase | at4g22570 | Inf | Inf | Inf | C07:1..48346208:42449416-42452378 |
| TCONS_00028466 | -NA- | -- | Inf | Inf | Inf | C07:1..48346208:42445748-42446256 |
| TCONS_00021114 | beta-glucosidase-like SFR2 | at3g06510 | 0.6 | 0.1 | 0.9 | C05:1..32828328:32662736-32664778 |
| TCONS_00020982 | nitrile-specifier 5-like | at3g07720 | 0.8 | 0.2 | 0.9 | C05:1..32828328:32037668-32039570 |
| TCONS_00024700 | photosystem II subunit S | at1g44575 | 1.0 | 0.3 | 0.9 | C06:1..40704471:37716207-37717902 |
| TCONS_00019565 | plastid transcriptionally active 6 | at1g21600 | 0.8 | 0.1 | 0.9 | C05:1..32828328:16944601-16945718 |
| TCONS_00028075 | -NA- | at4g16420 | 0.7 | 0.1 | 0.9 | C07:1..48346208:38608372-38610869 |
| TCONS_00001950 | rna-binding protein cp31 | at4g24770 | 0.9 | 0.3 | 0.9 | C01:1..38761720:11127837-11129627 |
| TCONS_00030146 | 50S ribosomal chloroplastic-like | at1g48350 | 0.7 | 0.3 | 0.9 | C08:1..41516064:8359637-8360745 |
| TCONS_00016792 | 50S ribosomal chloroplast | at2g33450 | 0.7 | 0.2 | 0.9 | C04:1..40895475:35310081-35311148 |
| TCONS_00017472 | peroxisomal biogenesis factor 11 family | at2g45740 | 1.0 | 0.2 | 0.9 | C04:1..40895475:40446146-40447046 |
| TCONS_00035176 | -NA- | -- | 0.5 | 0.3 | 0.9 | C09:1..40126856:7514136-7514843 |
| TCONS_00012813 | -NA- | at4g35750 | 1.7 | 1.1 | 0.9 | C03:1..57781463:49539338-49541103 |
| TCONS_00016831 | 30S ribosomal S5 | at2g33800 | 0.8 | 0.3 | 0.9 | C04:1..40895475:35535707-35537045 |
| TCONS_00002941 | pyruvate kinase isozyme chloroplastic-like | at3g22960 | 0.5 | 0.2 | 1.0 | C01:1..38761720:27472325-27473742 |
| TCONS_00008560 | magnesium chelatase subunit H | at5g13630 | 0.9 | 0.3 | 1.0 | C03:1..57781463:2284083-2289728 |
| TCONS_00014802 | 30S ribosomal chloroplastic-like | at3g52150 | 0.8 | 0.1 | 1.0 | C04:1..40895475:8370598-8371878 |
| TCONS_00031753 | 50S ribosomal chloroplastic-like | at3g54210 | 0.7 | 0.2 | 1.0 | C08:1..41516064:28872290-28873441 |
| TCONS_00019478 | plastocyanin | at1g20340 | 0.7 | 0.5 | 1.0 | C05:1..32828328:15803255-15804195 |
| TCONS_00033293 | glyceraldehyde-3-phosphate dehydrogenase (NADP+) (phosphorylating) | at1g12900 | 1.0 | 0.2 | 1.0 | C08:1..41516064:38525919-38528643 |
| TCONS_00023472 | photosystem I reaction center subunit chloroplastic-like | at1g55670 | 1.1 | 0.0 | 1.0 | C06:1..40704471:20002536-20003428 |
| TCONS_00033592 | 40S ribosomal S15a-1-like | at5g59850 | 1.1 | 0.4 | 1.0 | C08:1..41516064:40159173-40160415 |
| TCONS_00035478 | ATP synthase gamma chloroplastic-like | at4g04640 | 0.9 | 0.1 | 1.0 | C09:1..40126856:11896583-11898390 |
| TCONS_00005422 | apo protein chloroplastic-like | at5g57930 | 1.0 | 0.2 | 1.0 | C02:1..44046003:8844458-8846267 |
| TCONS_00024928 | triacylglycerol lipase 1 | at2g15230 | 0.9 | 0.2 | 1.0 | C07:1..48346208:15712435-15739307 |
| TCONS_00009801 | ribulose bisphosphate carboxylase oxygenase activase chloroplastic-like | at2g39730 | 1.0 | 0.3 | 1.0 | C03:1..57781463:10909952-10911192 |
| TCONS_00025521 | Late embryogenesis abundant group 2 | at3g24600 | 0.4 | 1.1 | 1.0 | C07:1..48346208:2940522-2941423 |
| TCONS_00029216 | sigma factor regulation -like | at4g37470 | 1.3 | 0.2 | 1.0 | C07:1..48346208:47671803-47673399 |
| TCONS_00004545 | chaperone protein dnaj-like protein | at5g06130 | 1.1 | 0.0 | 1.0 | C02:1..44046003:1935986-1936761 |
| TCONS_00012296 | -NA- | -- | 1.4 | 0.2 | 1.0 | C03:1..57781463:43062431-43062892 |
| TCONS_00012224 | phototropin 1 | at3g45780 | 1.6 | 0.5 | 1.0 | C03:1..57781463:41340098-41342027 |
| TCONS_00029186 | chloroplast 1 precursor | p12359|psbo_spiol | 1.1 | 0.2 | 1.0 | C07:1..48346208:47504887-47506442 |
| TCONS_00036405 | MORN (Membrane Occupation and Recognition Nexus) repeat-containing | at5g22640 | 0.8 | 0.2 | 1.0 | C09:1..40126856:28672654-28674613 |
| TCONS_00017059 | protease inhibitor seed storage lipid transfer family | at2g37870 | 1.2 | 0.0 | 1.0 | C04:1..40895475:37495312-37495977 |
| TCONS_00013461 | chaperonin 60 subunit beta 1 | p21241|rubb_brana | 1.3 | -0.0 | 1.0 | C03:1..57781463:57613971-57615712 |
| TCONS_00021606 | photosystem II subunit R | p49108|psbr_bracm | 1.4 | 0.2 | 1.0 | C06:1..40704471:454506-455846 |
| TCONS_00024743 | -NA- | -- | 1.5 | -0.3 | 1.0 | C06:1..40704471:38538448-38540015 |
| TCONS_00018337 | P-loop containing nucleoside triphosphate hydrolase domain and Clp-N motif-containing | at1g07200 | 0.8 | 0.6 | 1.0 | C05:1..32828328:2483934-2485817 |
| TCONS_00036493 | aldehyde oxidase | at5g20960 | 1.3 | 0.3 | 1.0 | C09:1..40126856:29674621-29676610 |
| TCONS_00003290 | AP2 domain containing | at3g16770 | 0.8 | 0.1 | 1.0 | C01:1..38761720:32512517-32513943 |
| TCONS_00029270 | brassinosteroid insensitive 1 | at4g39400 | 1.4 | 0.2 | 1.0 | C07:1..48346208:48127673-48131795 |
| TCONS_00014119 | Signal transducer and transcription activator isoform 1 | at2g43340 | 1.3 | 0.5 | 1.0 | C04:1..40895475:1482758-1483877 |
| TCONS_00020990 | zinc finger CONSTANS-LIKE 9 | at3g07650 | 0.8 | -0.0 | 1.0 | C05:1..32828328:32074004-32075845 |
| TCONS_00030127 | signal recognition particle 43 kDa chloroplastic-like | at2g47450 | 1.2 | 0.5 | 1.0 | C08:1..41516064:8010136-8011804 |
| TCONS_00028748 | protochlorophyllide reductase precursor | at4g27440 | 0.8 | -0.0 | 1.0 | C07:1..48346208:44444106-44445792 |
| TCONS_00005581 | thiamin biosynthetic enzyme | at5g54770 | 1.2 | -0.2 | 1.0 | C02:1..44046003:10897466-10897986 |
| TCONS_00002338 | protein tic chloroplastic-like | at3g46780 | 1.3 | 0.1 | 1.1 | C01:1..38761720:15387080-15388514 |
| TCONS_00027983 | phosphoglycerate mutase family | at3g52155 | 1.0 | 0.3 | 1.1 | C07:1..48346208:37976260-37977749 |
| TCONS_00034585 | 6,7-dimethyl-8-ribityllumazine synthase | at3g32930 | 1.4 | 0.6 | 1.1 | C09:1..40126856:2507909-2509605 |
| TCONS_00005170 | glycosyl hydrolase family 3 | at5g20950 | 1.0 | 0.4 | 1.1 | C02:1..44046003:5805209-5808097 |
| TCONS_00035960 | 50S ribosomal L24 | at5g54600 | 1.1 | 0.2 | 1.1 | C09:1..40126856:22807017-22808374 |
| TCONS_00034923 | serine-rich | at5g25280 | 1.2 | 0.6 | 1.1 | C09:1..40126856:4674712-4677092 |
| TCONS_00003071 | AT3g20680 F3H11 7 | at3g20680 | 1.0 | 0.4 | 1.1 | C01:1..38761720:29867840-29869105 |
| TCONS_00024449 | -NA- | -- | 0.8 | 0.1 | 1.1 | C06:1..40704471:33188447-33189583 |
| TCONS_00037769 | -NA- | -- | 0.9 | 0.0 | 1.1 | C09:1..40126856:39041427-39042438 |
| TCONS_00020480 | late embryogenesis-abundant | p13934|lea76_brana | -0.4 | 0.8 | 1.1 | C05:1..32828328:27795054-27796031 |
| TCONS_00014656 | vacuolar amino acid transporter 1-like | at2g39130 | 1.9 | 0.5 | 1.1 | C04:1..40895475:6650885-6651820 |
| TCONS_00011434 | -NA- | -- | 1.2 | 0.6 | 1.1 | C03:1..57781463:26873059-26874059 |
| TCONS_00003108 | Delta(24)-sterol reductase | at3g19820 | 1.0 | 0.3 | 1.1 | C01:1..38761720:30379862-30382771 |
| TCONS_00019966 | Delta(24)-sterol reductase | at3g19820 | 1.1 | 0.1 | 1.1 | C05:1..32828328:22809838-22811935 |
| TCONS_00028016 | DEF (CLA1) | at4g15560 | 0.9 | 0.1 | 1.1 | C07:1..48346208:38249516-38251589 |
| TCONS_00006302 | ATP synthase gamma chloroplastic-like | at4g04640 | 1.4 | 0.3 | 1.1 | C02:1..44046003:23328554-23330257 |
| TCONS_00005098 | spermine synthase | at5g19530 | 1.1 | 0.4 | 1.1 | C02:1..44046003:5351311-5353046 |
| TCONS_00015904 | -NA- | -- | 0.9 | 0.6 | 1.1 | C04:1..40895475:25075867-25076673 |
| TCONS_00035303 | 30S ribosomal chloroplastic-like | at1g79850 | 1.1 | 0.3 | 1.1 | C09:1..40126856:9316785-9317469 |
| TCONS_00022513 | -NA- | at1g69523 | 1.4 | 0.6 | 1.1 | C06:1..40704471:7418002-7419799 |
| TCONS_00035286 | Nuclear transcription factor Y subunit B-3 | at5g47640 | 1.3 | 0.3 | 1.1 | C09:1..40126856:9165991-9168257 |
| TCONS_00018197 | 50S ribosomal chloroplastic-like | at1g05190 | 1.1 | 0.3 | 1.1 | C05:1..32828328:1562462-1563501 |
| TCONS_00029290 | fructose bisphosphate aldolase | at4g38970 | 1.2 | 0.2 | 1.1 | C07:1..48346208:48240739-48242318 |
| TCONS_00027896 | two-component response regulator-like APRR1-like | at5g61380 | 0.5 | -0.4 | 1.1 | C07:1..48346208:37244922-37248002 |
| TCONS_00034452 | cytochrome b6-f complex iron-sulfur subunit | at4g03280 | 1.4 | 0.4 | 1.1 | C09:1..40126856:1504398-1505418 |
| TCONS_00004754 | cbs domain protein | at5g10860 | 1.2 | 0.5 | 1.1 | C02:1..44046003:2925079-2926533 |
| TCONS_00005937 | ac016662 23 glucosyltransferase 88035-86003 | at1g73885 | 0.9 | 0.2 | 1.1 | C02:1..44046003:17058562-17059870 |
| TCONS_00007379 | sterol 3-beta-glucosyltransferase-like | at5g24750 | 0.9 | 0.2 | 1.1 | C02:1..44046003:43570757-43571637 |
| TCONS_00020991 | zinc finger CONSTANS-LIKE 9 | at3g07650 | 0.7 | -0.1 | 1.1 | C05:1..32828328:32075981-32076520 |
| TCONS_00014828 | rubredoxin family | at1g54500 | 1.0 | 0.5 | 1.1 | C04:1..40895475:8596850-8597845 |
| TCONS_00032646 | acetone-cyanohydrin lyase | at2g23620 | 0.6 | 0.3 | 1.1 | C08:1..41516064:34707342-34708587 |
| TCONS_00025376 | COP1-interacting | at5g43310 | 0.9 | 0.3 | 1.1 | C07:1..48346208:413582-416398 |
| TCONS_00019513 | -NA- | at1g20810 | 0.9 | 0.5 | 1.1 | C05:1..32828328:16187126-16188709 |
| TCONS_00012085 | -NA- | -- | 0.9 | 0.6 | 1.1 | C03:1..57781463:38625892-38626579 |
| TCONS_00001590 | protein | at4g20820 | 1.0 | 0.1 | 1.2 | C01:1..38761720:7452658-7454854 |
| TCONS_00026122 | zinc finger family | at2g15580 | 1.7 | 0.4 | 1.2 | C07:1..48346208:15093569-15094822 |
| TCONS_00037431 | 80A08 3 | -- | 0.4 | 0.6 | 1.2 | C09:1..40126856:37306213-37306863 |
| TCONS_00001984 | magnesium-protoporphyrin IX methyltransferase | at4g25080 | 1.5 | 0.2 | 1.2 | C01:1..38761720:11330065-11330701 |
| TCONS_00005582 | thiazole biosynthetic enzyme | at5g54770 | 1.3 | -0.0 | 1.2 | C02:1..44046003:10899188-10899617 |
| TCONS_00003960 | SET domain-containing | at3g07670 | 1.0 | -0.2 | 1.2 | C01:1..38761720:38399430-38400652 |
| TCONS_00014974 | phototropic-responsive NPH3 family | at1g67900 | 1.3 | 0.6 | 1.2 | C04:1..40895475:10432124-10434297 |
| TCONS_00017163 | ribulose bisphosphate carboxylase oxygenase activase | at2g39730 | 1.2 | 0.4 | 1.2 | C04:1..40895475:38243700-38246089 |
| TCONS_00002355 | bundle-sheath defective protein 2 family | at3g47650 | 1.1 | -0.1 | 1.2 | C01:1..38761720:15932008-15933150 |
| TCONS_00007062 | cytochrome c oxidase subunit 5c | at2g47380 | 1.2 | 0.5 | 1.2 | C02:1..44046003:39689068-39690633 |
| TCONS_00023301 | tubulin beta-2 beta-3 chain | at5g62700 | 0.9 | 0.5 | 1.2 | C06:1..40704471:17929302-17930277 |
| TCONS_00024100 | 50S ribosomal chloroplastic-like | at3g54210 | 0.9 | 0.4 | 1.2 | C06:1..40704471:29905031-29906051 |
| TCONS_00014967 | chloroplast sensor kinase | at1g67840 | 1.4 | 0.6 | 1.2 | C04:1..40895475:10302908-10304233 |
| TCONS_00016274 | fructose bisphosphate aldolase | at2g21330 | 0.8 | -0.0 | 1.2 | C04:1..40895475:29399696-29400822 |
| TCONS_00019087 | 2-succinylbenzoate-- chloroplastic peroxisomal-like | at1g30520 | 0.8 | 0.5 | 1.2 | C05:1..32828328:10809885-10811342 |
| TCONS_00016244 | chloride channel CLC-a | at5g40890 | -0.8 | -0.8 | 1.2 | C04:1..40895475:29151306-29152823 |
| TCONS_00037466 | transcription factor transcription regulator | at5g09460 | 1.0 | 0.1 | 1.2 | C09:1..40126856:37539203-37541938 |
| TCONS_00031914 | biotin lipoyl attachment domain-containing | at3g56130 | 1.2 | 0.5 | 1.2 | C08:1..41516064:30072237-30072786 |
| TCONS_00023685 | P-loop containing nucleoside triphosphate hydrolases superfamily | at1g33290 | 1.2 | 0.1 | 1.2 | C06:1..40704471:23527496-23529582 |
| TCONS_00032645 | acetone-cyanohydrin lyase | at2g23600 | 0.8 | 0.4 | 1.2 | C08:1..41516064:34706285-34707145 |
| TCONS_00011998 | transcription factor MYB48 | at3g46130 | 1.1 | -0.2 | 1.2 | C03:1..57781463:35480363-35481740 |
| TCONS_00018113 | cryptochrome partial | at1g04400 | 1.1 | 0.1 | 1.2 | C05:1..32828328:1185322-1186233 |
| TCONS_00001223 | pentatricopeptide repeat-containing protein | at4g30825 | 1.5 | 0.5 | 1.2 | C01:1..38761720:4164382-4167098 |
| TCONS_00035259 | -NA- | at5g47110 | 0.9 | 0.1 | 1.2 | C09:1..40126856:8901530-8902829 |
| TCONS_00032632 | ribosomal L35 | at2g24090 | 1.2 | 0.4 | 1.2 | C08:1..41516064:34534709-34535897 |
| TCONS_00035822 | thylakoid lumenal - | at5g53490 | 0.9 | 0.3 | 1.2 | C09:1..40126856:20654680-20656088 |
| TCONS_00028558 | acyl- -binding domain 3 | at4g24230 | 0.6 | 0.1 | 1.2 | C07:1..48346208:43428618-43430649 |
| TCONS_00026300 | photosystem II type I chlorophyll a b binding | at1g29930 | 0.9 | 0.3 | 1.2 | C07:1..48346208:17935719-17936674 |
| TCONS_00036492 | aldehyde oxidase | at5g20960 | 1.1 | 0.6 | 1.2 | C09:1..40126856:29673337-29674499 |
| TCONS_00018114 | cryptochrome partial | at1g04400 | 1.3 | 0.0 | 1.2 | C05:1..32828328:1186574-1187477 |
| TCONS_00001985 | magnesium-protoporphyrin ix methyltransferase | at4g25080 | 1.3 | 0.4 | 1.2 | C01:1..38761720:11330800-11331061 |
| TCONS_00018050 | thylakoid lumenal chloroplastic-like | at1g03600 | 1.3 | 0.3 | 1.3 | C05:1..32828328:886922-887698 |
| TCONS_00009632 | peroxidase ATP2a | at2g37130 | 0.7 | 0.3 | 1.3 | C03:1..57781463:9471849-9473894 |
| TCONS_00030001 | protochlorophyllide reductase C | at1g03630 | 1.4 | -0.2 | 1.3 | C08:1..41516064:2984690-2986263 |
| TCONS_00007110 | lactoylglutathione lyase glyoxalase I | at5g48480 | 0.9 | -0.0 | 1.3 | C02:1..44046003:40625466-40626623 |
| TCONS_00018428 | magnesium-chelatase subunit chloroplastic-like | at1g08520 | 1.3 | 0.4 | 1.3 | C05:1..32828328:3102733-3103575 |
| TCONS_00010964 | -NA- | at3g13130 | 1.7 | 0.8 | 1.3 | C03:1..57781463:21028463-21029340 |
| TCONS_00016275 | fructose-bisphosphate aldolase 1 | at2g21330 | 1.3 | 0.1 | 1.3 | C04:1..40895475:29400943-29401926 |
| TCONS_00020594 | subtilisin-like protease-like | at3g14240 | 0.8 | 0.0 | 1.3 | C05:1..32828328:28811002-28813916 |
| TCONS_00030766 | -NA- | -- | 0.5 | -0.4 | 1.3 | C08:1..41516064:18146344-18146911 |
| TCONS_00019462 | Subtilisin-like serine endopeptidase family | at1g20160 | 0.9 | 0.0 | 1.3 | C05:1..32828328:15620258-15621736 |
| TCONS_00000620 | polyol transporter 6 | at4g36670 | 1.5 | 0.6 | 1.3 | C01:1..38761720:1201660-1204665 |
| TCONS_00006504 | NUCLEAR FUSION DEFECTIVE 4-like [Brassica napus] | at5g45275 | 1.3 | 0.6 | 1.3 | C02:1..44046003:30565496-30567233 |
| TCONS_00030739 | At1g10180 F14N23 6 | at1g10180 | 1.6 | 0.7 | 1.3 | C08:1..41516064:17778548-17779618 |
| TCONS_00010138 | aquaporin PIP1-2 | at2g45960 | 0.3 | 0.8 | 1.3 | C03:1..57781463:13518999-13521393 |
| TCONS_00015490 | dienelactone hydrolase domain-containing | at2g32520 | 1.4 | 0.6 | 1.3 | C04:1..40895475:19128972-19129964 |
| TCONS_00009993 | ribose-phosphate pyrophosphokinase 4 | at2g42910 | 1.4 | 0.6 | 1.3 | C03:1..57781463:12329913-12331090 |
| TCONS_00006929 | cofactor assembly of complex C | at3g26710 | 1.2 | 0.6 | 1.3 | C02:1..44046003:38167302-38168637 |
| TCONS_00032343 | Zinc finger | at3g61460 | 1.0 | 0.2 | 1.3 | C08:1..41516064:32683341-32684312 |
| TCONS_00015660 | MTERF isoform 2 | at2g34620 | 1.3 | 0.3 | 1.3 | C04:1..40895475:21180907-21182255 |
| TCONS_00024569 | auxin efflux carrier component 7 | at1g70940 | 1.9 | 0.4 | 1.3 | C06:1..40704471:34592492-34595387 |
| TCONS_00034873 | hypothetical protein EUTSA v10004859mg | at5g23920 | 1.0 | 0.4 | 1.3 | C09:1..40126856:4277673-4279065 |
| TCONS_00032190 | GENOMES UNCOUPLED 4 | at3g59400 | 1.3 | 0.2 | 1.3 | C08:1..41516064:31708062-31709209 |
| TCONS_00033375 | monocysteinic thioredoxin CXXS1 | at1g11530 | 1.9 | 0.4 | 1.3 | C08:1..41516064:39013252-39014377 |
| TCONS_00023433 | Nuclear transcription factor Y subunit C-2 isoform 1 | at1g56170 | 1.2 | 0.1 | 1.3 | C06:1..40704471:19743119-19744371 |
| TCONS_00030360 | acylamino acid-releasing enzyme | at4g14570 | 1.7 | 0.2 | 1.3 | C08:1..41516064:12326750-12327479 |
| TCONS_00037246 | Tubulin beta chain | at5g12250 | 1.4 | 0.4 | 1.3 | C09:1..40126856:35982362-35984392 |
| TCONS_00015489 | dienelactone hydrolase family | at2g32520 | 1.0 | 0.4 | 1.3 | C04:1..40895475:19127426-19127874 |
| TCONS_00037079 | -NA- | at1g61240 | 2.0 | 0.3 | 1.3 | C09:1..40126856:34775880-34776925 |
| TCONS_00022512 | expansin A1 | at1g69530 | -0.2 | 0.6 | 1.3 | C06:1..40704471:7393617-7395125 |
| TCONS_00031881 | chloroplast sedoheptulose-1,7-bisphosphatase | at3g55800 | 1.4 | 0.4 | 1.3 | C08:1..41516064:29863935-29865714 |
| TCONS_00003659 | 2-cys peroxiredoxin bas1 | at3g11630 | 0.9 | 0.5 | 1.4 | C01:1..38761720:36411573-36412393 |
| TCONS_00003381 | trichome birefringence-like 11 | at5g19160 | 1.1 | 0.6 | 1.4 | C01:1..38761720:33717911-33719895 |
| TCONS_00004400 | transcription activator glk1-like | at5g44190 | 1.6 | 1.0 | 1.4 | C02:1..44046003:673951-675376 |
| TCONS_00006539 | magnesium-chelatase subunit chloroplastic-like | at5g45930 | 1.2 | 0.2 | 1.4 | C02:1..44046003:31291383-31293232 |
| TCONS_00006825 | expansin a1 | at2g03090 | 0.4 | -0.0 | 1.4 | C02:1..44046003:35612327-35613617 |
| TCONS_00020750 | FKBP-type peptidyl-prolyl cis-trans | at3g12345 | 1.4 | 0.1 | 1.4 | C05:1..32828328:30208642-30209440 |
| TCONS_00035368 | -NA- | -- | 0.7 | -0.2 | 1.4 | C09:1..40126856:10609089-10609677 |
| TCONS_00035438 | Wound-responsive family | at4g05070 | 1.7 | 0.2 | 1.4 | C09:1..40126856:11528956-11529684 |
| TCONS_00032433 | hypothetical protein EUTSA v10006025mg | at3g62630 | 1.7 | 0.6 | 1.4 | C08:1..41516064:33260443-33262113 |
| TCONS_00006713 | abc transporter g family member 7 | at2g01320 | 1.2 | 0.1 | 1.4 | C02:1..44046003:34162809-34164037 |
| TCONS_00015077 | NDH-dependent cyclic electron flow 1 | at1g64770 | 1.9 | 0.0 | 1.4 | C04:1..40895475:12386090-12387208 |
| TCONS_00006715 | abc transporter g family member 7 | at2g01320 | 1.6 | 0.3 | 1.4 | C02:1..44046003:34164839-34166632 |
| TCONS_00015753 | plastid-specific 50S ribosomal 5 | at3g56910 | 1.7 | 0.9 | 1.4 | C04:1..40895475:21975343-21976308 |
| TCONS_00031592 | RNA recognition motif-containing | at3g52150 | 0.9 | 0.6 | 1.4 | C08:1..41516064:27556198-27557969 |
| TCONS_00027388 | exonuclease family | at3g27970 | 0.9 | 0.2 | 1.4 | C07:1..48346208:33340683-33342372 |
| TCONS_00026585 | -NA- | -- | 1.7 | 0.6 | 1.4 | C07:1..48346208:24327004-24328161 |
| TCONS_00013116 | TPX2 (targeting for Xklp2) family | at4g32330 | 1.3 | 0.5 | 1.4 | C03:1..57781463:53737763-53738790 |
| TCONS_00023279 | Xanthine uracil permease family | at5g62890 | 0.8 | 0.5 | 1.4 | C06:1..40704471:17760375-17761428 |
| TCONS_00015848 | -NA- | -- | 1.2 | 0.6 | 1.4 | C04:1..40895475:24176329-24177975 |
| TCONS_00017904 | aquaporin PIP1-3 | at1g01620 | 1.2 | 0.2 | 1.4 | C05:1..32828328:60086-60719 |
| TCONS_00004577 | kinesin motor protein | at5g06670 | 1.0 | 0.0 | 1.4 | C02:1..44046003:2051282-2052040 |
| TCONS_00017903 | plasma membrane intrinsic 1 4 | at1g01620 | 1.2 | 0.2 | 1.4 | C05:1..32828328:59335-59989 |
| TCONS_00016846 | uncharacterized LOC101213935 | at2g34340 | 1.9 | 1.1 | 1.4 | C04:1..40895475:35681774-35682804 |
| TCONS_00005542 | trigger factor-like protein tig-like | at5g55220 | 1.3 | 0.0 | 1.4 | C02:1..44046003:10499000-10500724 |
| TCONS_00020086 | fiber Fb34 | at1g13380 | 0.8 | 0.4 | 1.4 | C05:1..32828328:24974263-24975485 |
| TCONS_00030842 | S-adenosyl-L-methionine-dependent methyltransferase domain-containing | at5g44590 | 1.5 | 0.1 | 1.4 | C08:1..41516064:18940232-18940928 |
| TCONS_00000426 | fructose bisphosphate aldolase | at4g38970 | 2.2 | 0.0 | 1.4 | C01:1..38761720:53262-53619 |
| TCONS_00037828 | ferredoxin-dependent glutamate synthase 1 | at5g04140 | 0.1 | -0.3 | 1.4 | C09:1..40126856:39350531-39350911 |
| TCONS_00001932 | crt homolog 1-like | at4g24460 | 1.2 | 0.4 | 1.4 | C01:1..38761720:11033693-11034874 |
| TCONS_00017007 | peroxidase ATP2a | at2g37130 | 0.7 | 0.0 | 1.4 | C04:1..40895475:37163568-37165550 |
| TCONS_00019829 | leucine-rich repeat receptor | at3g20820 | 1.5 | 0.6 | 1.5 | C05:1..32828328:20985137-20986551 |
| TCONS_00004766 | adenine phosphoribosyltransferase | at5g11160 | 1.3 | 0.5 | 1.5 | C02:1..44046003:3012176-3014072 |
| TCONS_00005541 | trigger factor-like protein tig-like | at5g55220 | 1.0 | -0.1 | 1.5 | C02:1..44046003:10497855-10498583 |
| TCONS_00021868 | vacuolar-type H+-ATPase subunit B3 (VHA-B3) | at1g20260 | 1.5 | 0.4 | 1.5 | C06:1..40704471:1952940-1953642 |
| TCONS_00021761 | -NA- | -- | 0.5 | 1.0 | 1.5 | C06:1..40704471:1243220-1244132 |
| TCONS_00005487 | kinase family | at5g56890 | 1.4 | 0.5 | 1.5 | C02:1..44046003:9982895-9986309 |
| TCONS_00005136 | phd finger family protein | at5g20510 | 1.8 | 0.8 | 1.5 | C02:1..44046003:5505594-5507408 |
| TCONS_00034933 | -NA- | at5g67370 | 1.6 | 0.6 | 1.5 | C09:1..40126856:4832865-4834992 |
| TCONS_00034263 | kinase family | at4g01330 | 1.6 | 0.8 | 1.5 | C09:1..40126856:259721-261217 |
| TCONS_00031829 | Rossmann-fold NAD(P)-binding domain-containing | at3g55290 | 1.8 | 0.6 | 1.5 | C08:1..41516064:29435514-29436942 |
| TCONS_00001951 | pectate lyase | at4g24780 | 0.8 | 0.2 | 1.5 | C01:1..38761720:11136356-11138260 |
| TCONS_00037221 | -NA- | at5g12440 | 1.3 | 0.5 | 1.5 | C09:1..40126856:35887755-35889621 |
| TCONS_00027109 | IQ-domain 28 | at2g02790 | 1.2 | 0.5 | 1.5 | C07:1..48346208:30587438-30588894 |
| TCONS_00009687 | acetyl-coenzyme A carboxylase carboxyl transferase subunit alpha | at2g38040 | 0.9 | 0.7 | 1.5 | C03:1..57781463:9993934-9995057 |
| TCONS_00005681 | zinc finger CONSTANS-LIKE 6 | at1g68520 | 1.0 | 0.2 | 1.5 | C02:1..44046003:12266267-12268119 |
| TCONS_00027914 | leucine-rich repeat receptor kinase TDR-like | at5g61480 | 0.5 | 0.2 | 1.5 | C07:1..48346208:37350522-37354397 |
| TCONS_00015099 | -NA- | -- | 2.0 | 0.3 | 1.5 | C04:1..40895475:13306532-13307549 |
| TCONS_00022004 | geranylgeranyl reductase | at1g74470 | 1.6 | 0.5 | 1.5 | C06:1..40704471:3136324-3137398 |
| TCONS_00018115 | cryptochrome 2 | p40115|phr1_sinal | 1.8 | 0.5 | 1.5 | C05:1..32828328:1187692-1188687 |
| TCONS_00006365 | nac domain protein | at4g01550 | 1.0 | 0.4 | 1.5 | C02:1..44046003:24887724-24890553 |
| TCONS_00008626 | EG2771 | at5g14910 | 1.6 | 0.3 | 1.5 | C03:1..57781463:2612563-2614303 |
| TCONS_00011464 | -NA- | -- | 0.8 | 0.6 | 1.5 | C03:1..57781463:27133836-27134831 |
| TCONS_00007060 | alpha- glucan phosphorylase l chloroplastic amyloplastic-like | at3g29320 | 1.2 | 0.5 | 1.5 | C02:1..44046003:39678213-39679678 |
| TCONS_00030949 | Chlorophyll a-b binding CP24 chloroplastic | at1g15820 | 1.7 | 0.8 | 1.5 | C08:1..41516064:19680658-19681755 |
| TCONS_00014817 | zinc finger CCCH domain-containing 46 | at3g51950 | 1.5 | 0.6 | 1.5 | C04:1..40895475:8446766-8449689 |
| TCONS_00010577 | M-type thioredoxin | q9xgs0|trxm_brana | 1.7 | 0.1 | 1.5 | C03:1..57781463:17927257-17928399 |
| TCONS_00022618 | clathrin assembly | at1g68110 | 1.8 | 1.1 | 1.6 | C06:1..40704471:8559328-8560842 |
| TCONS_00024686 | C-4 sterol methyl oxidase | at4g12110 | 0.5 | 0.4 | 1.6 | C06:1..40704471:37339031-37340730 |
| TCONS_00011789 | root phototropism 3-like | at5g64330 | 2.2 | 0.3 | 1.6 | C03:1..57781463:30876465-30877563 |
| TCONS_00027257 | tonoplast intrinsic | at3g26520 | 0.2 | 0.4 | 1.6 | C07:1..48346208:32275365-32276799 |
| TCONS_00000754 | plasma membrane intrinsic protein | at4g35100 | 1.8 | 0.1 | 1.6 | C01:1..38761720:1981818-1983621 |
| TCONS_00020592 | AT3g14310 MLN21 9 | at3g14310 | 1.5 | 0.8 | 1.6 | C05:1..32828328:28801232-28804908 |
| TCONS_00024568 | auxin efflux carrier component 4 | at1g70940 | 1.6 | 0.2 | 1.6 | C06:1..40704471:34590503-34592228 |
| TCONS_00032396 | peptidyl-prolyl cis-trans isomerase CYP20-3 | at3g62030 | 1.0 | 0.2 | 1.6 | C08:1..41516064:33119662-33120100 |
| TCONS_00035141 | AF375430 1 AT5g45310 K9E15 9 | at5g45310 | 1.8 | 0.7 | 1.6 | C09:1..40126856:7068212-7070127 |
| TCONS_00004181 | plastid ribosomal S21 | at3g27160 | 1.3 | 0.5 | 1.6 | C02:1..44046003:38532066-38544129 |
| TCONS_00020182 | -NA- | at5g51470 | 2.1 | -0.0 | 1.6 | C05:1..32828328:25556392-25557162 |
| TCONS_00003501 | beta galactosidase 1 | at3g13750 | 0.9 | 0.2 | 1.6 | C01:1..38761720:35081804-35082940 |
| TCONS_00015353 | phosphomethylpyrimidine synthase | at2g29630 | 1.5 | -0.3 | 1.6 | C04:1..40895475:16987661-16988701 |
| TCONS_00018427 | magnesium-chelatase subunit chloroplastic-like | o24133|chld_tobac | 1.9 | 0.4 | 1.6 | C05:1..32828328:3101304-3102265 |
| TCONS_00011653 | -NA- | at5g48050 | 0.5 | 0.6 | 1.6 | C03:1..57781463:29259565-29261480 |
| TCONS_00016754 | beta glucosidase 33 | at2g32860 | 1.4 | 0.4 | 1.6 | C04:1..40895475:34877958-34887437 |
| TCONS_00007249 | subtilisin-like protease-like | at5g67360 | 0.9 | 0.5 | 1.6 | C02:1..44046003:42117213-42119721 |
| TCONS_00006695 | -NA- | -- | 1.3 | 0.5 | 1.6 | C02:1..44046003:33938089-33939819 |
| TCONS_00027195 | ROTUNDIFOLIA like 15 | -- | 2.2 | 0.3 | 1.6 | C07:1..48346208:31588895-31589631 |
| TCONS_00007061 | alpha- glucan phosphorylase l chloroplastic amyloplastic-like | at3g29320 | 0.9 | 0.6 | 1.6 | C02:1..44046003:39680065-39680934 |
| TCONS_00012601 | fructose bisphosphate aldolase | at4g38970 | 1.8 | 0.7 | 1.7 | C03:1..57781463:46078595-46079467 |
| TCONS_00005631 | plastid ribosomal | at4g17560 | 1.2 | 0.3 | 1.7 | C02:1..44046003:11359433-11360081 |
| TCONS_00009167 | NADH dehydrogenase-like complex N | at5g58260 | 1.6 | 0.2 | 1.7 | C03:1..57781463:5672033-5672811 |
| TCONS_00019830 | 2-oxoglutarate (2OG) and Fe(II)-dependent oxygenase superfamily | at3g20810 | 1.5 | -0.8 | 1.7 | C05:1..32828328:20989927-20991494 |
| TCONS_00014324 | peroxisomal biogenesis factor 11 family | at2g45740 | 2.2 | 0.1 | 1.7 | C04:1..40895475:3039346-3039863 |
| TCONS_00015354 | phosphomethylpyrimidine synthase | at2g29630 | 1.6 | 0.0 | 1.7 | C04:1..40895475:16988851-16990866 |
| TCONS_00007244 | BTB POZ domain-containing At5g67385-like | at5g67385 | 1.9 | 0.8 | 1.7 | C02:1..44046003:42107126-42107770 |
| TCONS_00036086 | -NA- | -- | 0.9 | -0.0 | 1.7 | C09:1..40126856:24677001-24677468 |
| TCONS_00014325 | peroxisomal biogenesis factor 11 family | at2g45740 | 1.5 | 0.2 | 1.8 | C04:1..40895475:3040451-3041053 |
| TCONS_00005992 | ATP-dependent protease La domain-containing | at1g75460 | 1.7 | 0.8 | 1.8 | C02:1..44046003:18056956-18058455 |
| TCONS_00007358 | -NA- | at5g25240 | 2.5 | 0.8 | 1.8 | C02:1..44046003:43332227-43332827 |
| TCONS_00019083 | phosphoribulokinase precursor | at1g32060 | 1.7 | 0.4 | 1.8 | C05:1..32828328:10790167-10791025 |
| TCONS_00014422 | -NA- | -- | 0.6 | 0.3 | 1.8 | C04:1..40895475:4132460-4133685 |
| TCONS_00019084 | phosphoribulokinase precursor | at1g32060 | 2.0 | 0.0 | 1.8 | C05:1..32828328:10791474-10792284 |
| TCONS_00004533 | -NA- | at5g05965 | 1.1 | 0.8 | 1.8 | C02:1..44046003:1908528-1910129 |
| TCONS_00010917 | DNA-damage-repair toleration DRT100 | at3g12610 | 0.3 | 0.8 | 1.8 | C03:1..57781463:20755922-20757472 |
| TCONS_00005806 | auxin efflux carrier component 4 | at1g70940 | 1.2 | 0.0 | 1.8 | C02:1..44046003:14291604-14293565 |
| TCONS_00000424 | fructose bisphosphate aldolase | at4g38970 | 2.1 | 0.3 | 1.8 | C01:1..38761720:51222-52330 |
| TCONS_00024685 | C-4 sterol methyl oxidase | at4g12110 | 0.7 | 0.2 | 1.8 | C06:1..40704471:37337948-37338937 |
| TCONS_00024369 | walls are thin 1 | at1g75500 | 2.3 | 0.7 | 1.8 | C06:1..40704471:32252181-32254110 |
| TCONS_00005491 | beta-galactosidase | at4g26140 | 1.9 | 0.8 | 1.8 | C02:1..44046003:9988376-9989000 |
| TCONS_00001702 | peroxidase | at4g21960 | 1.6 | 0.4 | 1.8 | C01:1..38761720:8422164-8423921 |
| TCONS_00004455 | -NA- | -- | 0.1 | -0.8 | 1.9 | C02:1..44046003:1188420-1189129 |
| TCONS_00000425 | fructose bisphosphate aldolase | at4g38970 | 2.3 | 0.3 | 1.9 | C01:1..38761720:52447-52934 |
| TCONS_00034800 | timing of cab 1 | at5g61380 | 1.6 | -0.4 | 1.9 | C09:1..40126856:3881007-3882252 |
| TCONS_00022005 | geranylgeranyl reductase | at1g74470 | 2.3 | 0.5 | 1.9 | C06:1..40704471:3137574-3138633 |
| TCONS_00023376 | ZKT containing K-box and a TPR region | at1g55480 | 1.8 | -0.1 | 1.9 | C06:1..40704471:18816744-18817108 |
| TCONS_00011790 | root phototropism 3 | at5g64330 | 2.6 | 0.3 | 1.9 | C03:1..57781463:30877709-30878657 |
| TCONS_00006541 | gdsl esterase lipase at5g45960-like | at5g45960 | 2.1 | 0.9 | 1.9 | C02:1..44046003:31359735-31363539 |
| TCONS_00011573 | maternal effect embryo arrest 14 | at2g15890 | 1.8 | 0.1 | 2.0 | C03:1..57781463:28159219-28160964 |
| TCONS_00028600 | pectate lyase | at4g24780 | 0.8 | -0.1 | 2.0 | C07:1..48346208:43621101-43622461 |
| TCONS_00033348 | -NA- | -- | 0.5 | 0.8 | 2.0 | C08:1..41516064:38776468-38777236 |
| TCONS_00009611 | probable xyloglucan endotransglucosylase hydrolase 32 isoform X1 | at2g36870 | 0.7 | 1.0 | 2.0 | C03:1..57781463:9307056-9308948 |
| TCONS_00012483 | photosystem II type I chlorophyll a b binding | at1g29930 | 1.8 | 0.4 | 2.1 | C03:1..57781463:45048040-45049388 |
| TCONS_00000609 | PQ-loop repeat family transmembrane family | at4g36850 | 2.4 | 0.7 | 2.1 | C01:1..38761720:1135327-1135758 |
| TCONS_00034564 | AT3g29240 MXO21 9 | at3g29240 | 1.7 | 0.8 | 2.1 | C09:1..40126856:2370229-2371432 |
| TCONS_00008666 | ankyrin repeat-containing | at5g15500 | 1.7 | 0.7 | 2.1 | C03:1..57781463:2763984-2765241 |
| TCONS_00031672 | -NA- | -- | 2.5 | 0.5 | 2.1 | C08:1..41516064:28298418-28298862 |
| TCONS_00020642 | beta galactosidase 1 | at3g13750 | 2.1 | 1.1 | 2.1 | C05:1..32828328:29310627-29313468 |
| TCONS_00028533 | -NA- | at4g23870 | 1.8 | 1.1 | 2.2 | C07:1..48346208:43270642-43271791 |
| TCONS_00021904 | walls are thin 1 | at1g75500 | 1.4 | 0.6 | 2.3 | C06:1..40704471:2212467-2214229 |
| TCONS_00009634 | aquaporin PIP2-2-like [Brassica napus] | at2g37170 | -0.0 | 1.1 | 2.3 | C03:1..57781463:9483600-9485199 |
| TCONS_00018794 | Sterile alpha motif (SAM) domain-containing | at1g15760 | 2.1 | 0.7 | 2.3 | C05:1..32828328:6086664-6087816 |
| TCONS_00003224 | lipid transfer protein | at3g18280 | 2.2 | 0.0 | 2.5 | C01:1..38761720:31635212-31635810 |
| TCONS_00015382 | -NA- | -- | 1.4 | 0.2 | 2.5 | C04:1..40895475:17308952-17310248 |
| TCONS_00030419 | -NA- | at4g15990 | 1.1 | 1.5 | 2.5 | C08:1..41516064:13829680-13830366 |
| TCONS_00006786 | -NA- | -- | 2.9 | 0.7 | 2.6 | C02:1..44046003:35229522-35230321 |
| TCONS_00005658 | GDSL esterase lipase At3g26430-like | at1g67830 | 2.3 | 0.9 | 2.6 | C02:1..44046003:11763489-11764451 |
| TCONS_00005692 | probable nitrite transporter at1g68570-like | at1g68570 | 3.4 | 1.8 | 2.7 | C02:1..44046003:12441554-12443162 |
| TCONS_00029760 | -NA- | -- | 1.3 | 0.8 | 3.0 | C08:1..41516064:940752-941634 |

## ABA-down

Table S2.2. Downregulated gene products after 8 days of ABA treatment. Adjusted p-value <0.05. Gene name, as annotated by Blast2GO after best blastx in nr Viridiplantae database. Best Swiss-Prot hit (Uniprot manually annotated and reviewed protein database) as *Arabidopsis thaliana* locus name or Uniprot “Entry|Entry name” if best curated hit is from another species. ABA, Pyr and ABA+Pyr log2 fold change to control condition. Locus, *Brassica oleracea* v1.0 genomic locus (Liu et al. 2014).

| Isoform id | Gene name | Best Swiss-Prot hit | ABA | Pyr | ABA+Pyr | Locus |
| --- | --- | --- | --- | --- | --- | --- |
| TCONS_00010155 | jasmonic acid-amido synthetase JAR1 | at2g46370 | -3.1 | -0.4 | -5.0 | C03:1..57781463:13632243-13634255 |
| TCONS_00010154 | jasmonic acid-amido synthetase JAR1 | at2g46370 | -3.2 | -0.5 | -4.2 | C03:1..57781463:13630980-13632079 |
| TCONS_00021469 | -NA- | -- | -2.5 | -0.5 | -3.4 | C06:1..40704471:31279119-31282776 |
| TCONS_00027759 | probable WRKY transcription factor 41-like | at5g24110 | -3.1 | -0.4 | -3.2 | C07:1..48346208:36393305-36395049 |
| TCONS_00014480 | phenylalanine ammonia-lyase | at2g37040 | -3.1 | -0.5 | -3.2 | C04:1..40895475:4708274-4710312 |
| TCONS_00016702 | alanine acetyl transferase | at2g32020 | -4.6 | -1.0 | -3.2 | C04:1..40895475:34450981-34451830 |
| TCONS_00035370 | sulphotransferase 12 | at2g03760 | -4.5 | -0.8 | -3.1 | C09:1..40126856:10687818-10689022 |
| TCONS_00004587 | cytosolic sulfotransferase 15-like | at5g07010 | -2.4 | 0.3 | -3.0 | C02:1..44046003:2108784-2110354 |
| TCONS_00005654 | -NA- | -- | -3.7 | -0.3 | -2.9 | C02:1..44046003:11719515-11720109 |
| TCONS_00020934 | -NA- | -- | -3.1 | -1.2 | -2.8 | C05:1..32828328:31762375-31763017 |
| TCONS_00008279 | leucoanthocyanidin dioxygenase | at5g05600 | -3.1 | -1.2 | -2.6 | C03:1..57781463:899339-902214 |
| TCONS_00017000 | phenylalanine ammonia-lyase | at2g37040 | -2.2 | -1.3 | -2.6 | C04:1..40895475:37110384-37112545 |
| TCONS_00037798 | uncharacterized protein | at5g05300 | -3.9 | -0.8 | -2.5 | C09:1..40126856:39151017-39151681 |
| TCONS_00003691 | leucoanthocyanidin dioxygenase | at3g11180 | -4.8 | -0.6 | -2.5 | C01:1..38761720:36668622-36669910 |
| TCONS_00001643 | serine threonine kinase | at4g21390 | -2.9 | -0.6 | -2.5 | C01:1..38761720:7887535-7888146 |
| TCONS_00033004 | lipoxygenase 3 | at1g17420 | -3.0 | -1.6 | -2.5 | C08:1..41516064:36874625-36876372 |
| TCONS_00019154 | gibberellin 2-beta-dioxygenase 2 | at1g30040 | -2.7 | -1.0 | -2.5 | C05:1..32828328:11875592-11877914 |
| TCONS_00024296 | TOM1 2-like | at1g76970 | -3.5 | -0.7 | -2.5 | C06:1..40704471:31464226-31466581 |
| TCONS_00003693 | leucoanthocyanidin dioxygenase | at3g11180 | -3.5 | -0.6 | -2.5 | C01:1..38761720:36671428-36671890 |
| TCONS_00008737 | NAD(P)H dehydrogenase mitochondrial-like | at2g20800 | -4.2 | -0.9 | -2.4 | C03:1..57781463:3248310-3250561 |
| TCONS_00009333 | CBS domain-containing CBSX5-like | at5g53750 | -2.6 | -1.1 | -2.4 | C03:1..57781463:6938193-6939753 |
| TCONS_00024297 | retrotransposon like | p10978|polx_tobac | -3.9 | -0.3 | -2.4 | C06:1..40704471:31466722-31469455 |
| TCONS_00009737 | -NA- | at2g38790 | -3.0 | -0.9 | -2.4 | C03:1..57781463:10492454-10493659 |
| TCONS_00015945 | hypothetical protein EUTSA v10027932mg | at5g38310 | -2.7 | -0.3 | -2.3 | C04:1..40895475:25519949-25520864 |
| TCONS_00028551 | hypothetical protein EUTSA v10026459mg | at4g24130 | -2.1 | -0.7 | -2.3 | C07:1..48346208:43403720-43404556 |
| TCONS_00011087 | GATA transcription factor 17 | at3g16870 | -2.2 | -0.3 | -2.3 | C03:1..57781463:22368084-22368771 |
| TCONS_00012488 | gibberellin 2-beta-dioxygenase 2 | at1g30040 | -3.0 | -0.4 | -2.3 | C03:1..57781463:45127521-45130082 |
| TCONS_00014017 | Hop-interacting THI031 | at2g41730 | -3.1 | -1.1 | -2.3 | C04:1..40895475:627672-628367 |
| TCONS_00011901 | cytochrome P450 | at5g36220 | -1.9 | -0.9 | -2.2 | C03:1..57781463:33004911-33005566 |
| TCONS_00028567 | -NA- | -- | -2.2 | 0.0 | -2.2 | C07:1..48346208:43480675-43483475 |
| TCONS_00019355 | CDP-diacylglycerol--glycerol-3-phosphate 3- partial | at1g19020 | -3.1 | -0.0 | -2.2 | C05:1..32828328:14593080-14593710 |
| TCONS_00030925 | ATPDR7 PDR7 | at1g15210 | -3.7 | -1.2 | -2.2 | C08:1..41516064:19527996-19529428 |
| TCONS_00009282 | O-Glycosyl hydrolases family 17 | at5g55180 | -2.6 | -1.1 | -2.2 | C03:1..57781463:6438266-6440020 |
| TCONS_00034459 | probable indole-3-acetic acid-amido synthetase -like | at4g03400 | -2.0 | -0.4 | -2.2 | C09:1..40126856:1594791-1595652 |
| TCONS_00028119 | calcineurin B 1 | at4g17615 | -2.4 | 0.2 | -2.1 | C07:1..48346208:39014115-39015378 |
| TCONS_00011900 | cytochrome P450 | at5g36220 | -2.1 | -0.5 | -2.1 | C03:1..57781463:33002885-33004257 |
| TCONS_00036542 | leucine-rich repeat receptor kinase | at5g20480 | -1.9 | -0.5 | -2.1 | C09:1..40126856:30126146-30129161 |
| TCONS_00028307 | ras-related RABA1f-like | at4g18430 | -1.4 | -0.1 | -2.1 | C07:1..48346208:41033111-41033785 |
| TCONS_00010349 | -NA- | -- | -2.4 | -0.6 | -2.1 | C03:1..57781463:15441090-15441809 |
| TCONS_00032678 | VQ motif-containing | at2g22880 | -3.2 | -0.6 | -2.1 | C08:1..41516064:34949250-34950003 |
| TCONS_00030578 | uncharacterized protein | -- | -3.8 | -0.1 | -2.1 | C08:1..41516064:15930104-15930747 |
| TCONS_00014226 | RING U-box domain-containing | at2g44578 | -1.6 | -0.7 | -2.1 | C04:1..40895475:2374442-2375311 |
| TCONS_00019557 | -NA- | -- | -1.9 | -0.4 | -2.1 | C05:1..32828328:16843061-16843938 |
| TCONS_00011519 | TIR-NBS-LRR class disease resistance | at5g41750 | -2.2 | -0.4 | -2.0 | C03:1..57781463:27428088-27430533 |
| TCONS_00014195 | methylenetetrahydrofolate reductase | at2g44160 | -1.6 | -0.0 | -2.0 | C04:1..40895475:1863320-1864211 |
| TCONS_00011242 | -NA- | -- | -2.9 | -0.5 | -2.0 | C03:1..57781463:24120096-24120902 |
| TCONS_00030667 | -NA- | -- | -1.9 | -0.4 | -2.0 | C08:1..41516064:17222609-17222807 |
| TCONS_00002277 | vacuolar iron transporter-like protein | at4g27860 | -2.0 | -0.4 | -2.0 | C01:1..38761720:14063199-14063975 |
| TCONS_00005429 | -NA- | at5g57800 | -1.9 | -1.0 | -2.0 | C02:1..44046003:8877895-8879172 |
| TCONS_00005428 | protein eceriferum 3-like | at5g57800 | -2.4 | -0.7 | -2.0 | C02:1..44046003:8875621-8877500 |
| TCONS_00029242 | adenosine-5 -phosphosulfate-kinase | at4g39940 | -1.5 | 0.1 | -2.0 | C07:1..48346208:47912821-47914052 |
| TCONS_00034460 | Indole-3-acetic acid-amido synthetase | at4g03400 | -1.7 | -0.7 | -2.0 | C09:1..40126856:1595973-1597616 |
| TCONS_00032718 | -NA- | -- | -3.5 | -0.5 | -2.0 | C08:1..41516064:35292676-35294153 |
| TCONS_00017086 | anthocyanidin synthase | at2g38240 | -3.9 | -0.1 | -2.0 | C04:1..40895475:37685991-37688522 |
| TCONS_00005923 | nudix hydrolase mitochondrial-like | at1g73540 | -1.2 | -0.4 | -2.0 | C02:1..44046003:16738818-16739803 |
| TCONS_00031977 | metal ion binding | at3g56891 | -2.6 | -1.2 | -2.0 | C08:1..41516064:30515866-30516976 |
| TCONS_00026046 | receptor like 19 | at1g45616 | -1.8 | -0.2 | -1.9 | C07:1..48346208:13622982-13626522 |
| TCONS_00030926 | ATPDR7 PDR7 | at1g15210 | -2.8 | -1.5 | -1.9 | C08:1..41516064:19529552-19530582 |
| TCONS_00005545 | o-glycosyl hydrolases family 17 protein | at5g55180 | -3.1 | -0.5 | -1.9 | C02:1..44046003:10519385-10521094 |
| TCONS_00004528 | 2-oxoglutarate and fe -dependent oxygenase superfamily protein | at5g05600 | -2.5 | -0.2 | -1.9 | C02:1..44046003:1859779-1861985 |
| TCONS_00012358 | zinc finger | at1g27730 | -2.5 | -0.2 | -1.9 | C03:1..57781463:43718729-43719765 |
| TCONS_00020103 | tryptophan synthase beta chain chloroplastic-like | at5g28237 | -0.8 | -1.1 | -1.9 | C05:1..32828328:25102784-25103317 |
| TCONS_00022178 | TIFY 11B | at1g72450 | -1.6 | -0.7 | -1.9 | C06:1..40704471:4163378-4165555 |
| TCONS_00006824 | ribonuclease t2 | at2g02990 | -2.2 | -0.1 | -1.9 | C02:1..44046003:35578535-35579915 |
| TCONS_00011101 | S-adenosylmethionine synthetase | at3g17390 | -1.9 | -0.2 | -1.9 | C03:1..57781463:22532161-22534194 |
| TCONS_00033898 | -NA- | at5g52750 | -3.1 | -0.6 | -1.9 | C09:1..40126856:19179722-19192700 |
| TCONS_00006322 | MLO 1-like | at4g02600 | -0.8 | -0.7 | -1.9 | C02:1..44046003:24321674-24323606 |
| TCONS_00014637 | -NA- | at2g38790 | -2.3 | -0.4 | -1.9 | C04:1..40895475:6399338-6400385 |
| TCONS_00009283 | F-box family | at5g55150 | -3.6 | -0.9 | -1.9 | C03:1..57781463:6443157-6444442 |
| TCONS_00018728 | AC012189 5EST gb | at1g15010 | -2.9 | -0.8 | -1.9 | C05:1..32828328:5659318-5660043 |
| TCONS_00018560 | early-responsive to dehydration stress-related | at1g11960 | -2.0 | -0.6 | -1.9 | C05:1..32828328:3914957-3917318 |
| TCONS_00007931 | -NA- | at1g01680 | -3.2 | -1.1 | -1.9 | C03:1..57781463:13544901-13549304 |
| TCONS_00017212 | Laccase 12 | at2g40370 | -2.7 | -0.6 | -1.9 | C04:1..40895475:38615581-38617104 |
| TCONS_00008528 | -NA- | at5g13220 | -3.7 | -0.4 | -1.9 | C03:1..57781463:2161444-2162416 |
| TCONS_00024841 | -NA- | at1g48330 | -2.6 | 0.3 | -1.8 | C06:1..40704471:40281129-40282053 |
| TCONS_00005430 | -NA- | -- | -2.0 | -0.7 | -1.8 | C02:1..44046003:8879276-8879522 |
| TCONS_00036327 | zinc finger | at5g59820 | -2.3 | -0.1 | -1.8 | C09:1..40126856:27753436-27754196 |
| TCONS_00033718 | polygalacturonase At1g48100-like | at1g02460 | -2.3 | 0.3 | -1.8 | C08:1..41516064:41075460-41076773 |
| TCONS_00022779 | Sodium calcium exchanger family calcium-binding EF hand family | at1g53210 | -1.9 | -0.3 | -1.8 | C06:1..40704471:10485978-10488795 |
| TCONS_00001707 | wrky transcription factor 6-like | at4g22070 | -3.0 | -0.5 | -1.8 | C01:1..38761720:8522442-8524297 |
| TCONS_00004527 | 2-oxoglutarate and fe -dependent oxygenase superfamily protein | at5g05600 | -2.3 | -0.3 | -1.8 | C02:1..44046003:1858271-1859234 |
| TCONS_00025770 | 1-aminocyclopropane-1-carboxylate oxidase 1-like | at2g19590 | -3.4 | -0.2 | -1.8 | C07:1..48346208:7605748-7606927 |
| TCONS_00001234 | f-box family protein | at4g30640 | -3.0 | -0.7 | -1.8 | C01:1..38761720:4272206-4273630 |
| TCONS_00003400 | ethylene-responsive transcription factor erf109-like | at4g34410 | -2.0 | -0.9 | -1.8 | C01:1..38761720:33824967-33826210 |
| TCONS_00016989 | S-adenosylmethionine synthase 2-like | at2g36880 | -1.9 | 0.0 | -1.8 | C04:1..40895475:37013006-37014430 |
| TCONS_00004249 | omega-3 fatty acid desaturase | p48618|fad3c_brana | -2.6 | -0.8 | -1.8 | C02:1..44046003:10632458-10642384 |
| TCONS_00002326 | -NA- | at3g09110 | -2.7 | -0.6 | -1.8 | C01:1..38761720:15110236-15112673 |
| TCONS_00001034 | dehydration-responsive element-binding protein 3-like | at4g32800 | -1.7 | -0.9 | -1.8 | C01:1..38761720:3113738-3114908 |
| TCONS_00032685 | bHLH transcription factor | at2g22760 | -2.6 | -0.4 | -1.8 | C08:1..41516064:35106957-35109350 |
| TCONS_00003712 | AC009991 24hypothetical protein | -- | -3.0 | -0.4 | -1.8 | C01:1..38761720:36870019-36870601 |
| TCONS_00011614 | heat shock 70 | at5g02500 | -1.9 | -0.4 | -1.8 | C03:1..57781463:28794860-28797988 |
| TCONS_00023451 | -NA- | at1g56060 | -3.8 | -1.1 | -1.8 | C06:1..40704471:19830267-19831659 |
| TCONS_00028550 | metal-nicotianamine transporter YSL1-like | at4g24120 | -2.1 | -0.6 | -1.8 | C07:1..48346208:43401456-43402958 |
| TCONS_00020911 | omega-3 fatty acid desaturase | at3g11170 | -2.4 | -0.6 | -1.7 | C05:1..32828328:31621205-31624114 |
| TCONS_00027119 | ribonuclease 1 | at2g02990 | -2.4 | 0.0 | -1.7 | C07:1..48346208:30722275-30723653 |
| TCONS_00001588 | calcium binding protein | at4g20780 | -3.0 | -0.3 | -1.7 | C01:1..38761720:7435609-7436990 |
| TCONS_00018559 | ERD (early-responsive to dehydration stress) family | at1g11960 | -1.4 | -1.0 | -1.7 | C05:1..32828328:3913513-3914793 |
| TCONS_00022241 | -NA- | -- | -3.1 | -0.8 | -1.7 | C06:1..40704471:4416685-4417787 |
| TCONS_00023358 | ase inhibitor serpin | at1g55230 | -3.2 | -0.7 | -1.7 | C06:1..40704471:18661941-18663113 |
| TCONS_00003826 | probable glutathione S-transferase-like | at3g09270 | -2.0 | -0.7 | -1.7 | C01:1..38761720:37652222-37653848 |
| TCONS_00032657 | hypothetical protein ARALYDRAFT 900807 | at2g23270 | -2.4 | 1.1 | -1.7 | C08:1..41516064:34803930-34804629 |
| TCONS_00002276 | vacuolar iron transporter-like protein | at4g27860 | -2.0 | -0.6 | -1.7 | C01:1..38761720:14061791-14063110 |
| TCONS_00019915 | cytochrome P450 | at3g20140 | -2.6 | -2.4 | -1.7 | C05:1..32828328:22421879-22424258 |
| TCONS_00032763 | Dipeptide transport ATP-binding dppF | at2g21640 | -2.9 | -0.9 | -1.7 | C08:1..41516064:35650770-35652290 |
| TCONS_00024868 | Cys2 His2-type zinc finger 3 | at5g43170 | -2.6 | -0.2 | -1.7 | C07:1..48346208:552888-564382 |
| TCONS_00035433 | myb-related M4 | at4g05100 | -3.5 | -0.5 | -1.7 | C09:1..40126856:11505040-11506111 |
| TCONS_00016537 | leucine-rich repeat disease resistance | at2g33020 | -1.5 | -0.1 | -1.7 | C04:1..40895475:32370943-32373307 |
| TCONS_00005466 | calcium-transporting ATPase 8 | -- | -1.1 | -1.2 | -1.7 | C02:1..44046003:9709716-9710585 |
| TCONS_00030235 | At1g32920 F9L11 25 | at1g32920 | -2.5 | -0.7 | -1.7 | C08:1..41516064:10276561-10279873 |
| TCONS_00005471 | calcium-transporting ATPase 8 | at4g29900 | -1.5 | -0.4 | -1.7 | C02:1..44046003:9715460-9717553 |
| TCONS_00033731 | 3-ketoacyl- synthase 1 | at1g01120 | -1.5 | -1.3 | -1.7 | C08:1..41516064:41177474-41179315 |
| TCONS_00008973 | heat shock family | at5g20970 | -1.7 | -0.5 | -1.6 | C03:1..57781463:4477611-4478885 |
| TCONS_00027755 | squalene epoxidase homologue | o65726|erg12_brana | -0.5 | 0.6 | -1.6 | C07:1..48346208:36361373-36364329 |
| TCONS_00007018 | xyloglucan glycosyltransferase 4-like | at3g28180 | -2.3 | -0.1 | -1.6 | C02:1..44046003:39204549-39205464 |
| TCONS_00015057 | N-hydroxycinnamoyl benzoyltransferase | at5g42830 | -2.4 | -0.1 | -1.6 | C04:1..40895475:11949248-11950561 |
| TCONS_00029419 | -NA- | at1g19180 | -2.1 | 0.0 | -1.6 | C08:1..41516064:21111675-21165176 |
| TCONS_00021528 | cinnamoyl- reductase 2-2 | at1g80820 | -2.5 | -0.5 | -1.6 | C06:1..40704471:61740-63061 |
| TCONS_00019804 | -NA- | at1g76590 | -1.9 | -0.8 | -1.6 | C05:1..32828328:20350907-20352638 |
| TCONS_00019654 | metallo ase | at1g24140 | -3.2 | 0.0 | -1.6 | C05:1..32828328:17894433-17895860 |
| TCONS_00020872 | S-adenosylmethionine synthetase | at3g17390 | -1.8 | 1.3 | -1.6 | C05:1..32828328:31126473-31127979 |
| TCONS_00021791 | O-methyltransferase family 2 | at1g76790 | -1.4 | 0.2 | -1.6 | C06:1..40704471:1486444-1486949 |
| TCONS_00005446 | RNA-binding with serine-rich domain 1-like | at5g57510 | -3.2 | -0.5 | -1.6 | C02:1..44046003:9259465-9260621 |
| TCONS_00000827 | ethylene-responsive transcription factor erf109-like | at4g34410 | -3.3 | -0.6 | -1.6 | C01:1..38761720:2326030-2327105 |
| TCONS_00016009 | unnamed protein product | -- | -1.7 | -0.2 | -1.6 | C04:1..40895475:26504100-26505782 |
| TCONS_00017449 | leucine-rich repeat transmembrane kinase | at2g45340 | -1.5 | 0.0 | -1.6 | C04:1..40895475:40315832-40318014 |
| TCONS_00002259 | at5g54141 $at5g54141 | at4g27657 | -1.5 | -0.9 | -1.6 | C01:1..38761720:13783723-13784280 |
| TCONS_00015754 | metal ion binding | at3g56891 | -2.7 | -0.8 | -1.6 | C04:1..40895475:21977546-21978664 |
| TCONS_00000709 | -NA- | -- | -3.1 | -0.6 | -1.6 | C01:1..38761720:1789888-1790602 |
| TCONS_00022599 | hypothetical protein EUTSA v10019384mg | at1g68500 | -0.6 | 0.5 | -1.6 | C06:1..40704471:8255017-8256312 |
| TCONS_00007383 | -NA- | -- | -0.9 | -0.0 | -1.5 | C02:1..44046003:43621558-43622223 |
| TCONS_00029128 | hypothetical protein ARALYDRAFT 333079 | -- | -2.8 | -1.0 | -1.5 | C07:1..48346208:47130996-47131712 |
| TCONS_00029241 | adenosine-5 -phosphosulfate-kinase | -- | -1.8 | 0.3 | -1.5 | C07:1..48346208:47912156-47912757 |
| TCONS_00000632 | salicylate o-methyltransferase-like | at4g36470 | -2.3 | -0.4 | -1.5 | C01:1..38761720:1283035-1285760 |
| TCONS_00000515 | -NA- | -- | -1.8 | -0.7 | -1.5 | C01:1..38761720:564661-564971 |
| TCONS_00002207 | calcium-binding PBP1-like | at4g27280 | -1.9 | -0.5 | -1.5 | C01:1..38761720:13432930-13433438 |
| TCONS_00030205 | K+ uptake permease 9 | at4g19960 | -1.1 | -0.2 | -1.5 | C08:1..41516064:9160110-9163514 |
| TCONS_00004364 | bahd acyltransferase dcr-like | at5g01210 | -2.2 | -0.4 | -1.5 | C02:1..44046003:171099-173028 |
| TCONS_00014663 | nodulin family | at2g39210 | -1.2 | -0.5 | -1.5 | C04:1..40895475:6686754-6688376 |
| TCONS_00009735 | annexin D4-like | at2g38750 | -1.8 | -0.2 | -1.5 | C03:1..57781463:10482754-10484688 |
| TCONS_00016117 | short-chain type dehydrogenase reductase-like | at4g13180 | -2.5 | -0.0 | -1.5 | C04:1..40895475:27439798-27440758 |
| TCONS_00005470 | calcium-transporting ATPase 8 | at5g57110 | -1.5 | -0.6 | -1.5 | C02:1..44046003:9713563-9715317 |
| TCONS_00034874 | calcium-dependent lipid-binding domain-containing | at5g23950 | -1.6 | -0.2 | -1.5 | C09:1..40126856:4280592-4281958 |
| TCONS_00033005 | TIFY 11A | at1g17380 | -1.9 | -0.5 | -1.5 | C08:1..41516064:36896185-36898380 |
| TCONS_00033664 | glutaredoxin ATGRXS13 | at1g03850 | -3.3 | -0.8 | -1.5 | C08:1..41516064:40751704-40752547 |
| TCONS_00028752 | early nodulin 2 | at4g27520 | -0.5 | 0.1 | -1.5 | C07:1..48346208:44459965-44462398 |
| TCONS_00008687 | 3-oxo-5-alpha-steroid 4-dehydrogenase family | at5g16010 | -1.5 | -0.6 | -1.5 | C03:1..57781463:2979622-2980578 |
| TCONS_00035098 | nodulin family | at2g16660 | -3.2 | -1.0 | -1.5 | C09:1..40126856:6340077-6341360 |
| TCONS_00026047 | S-adenosyl-L-methionine-dependent methyltransferases superfamily | at3g44840 | -3.3 | 0.4 | -1.5 | C07:1..48346208:13675479-13676895 |
| TCONS_00011691 | -NA- | at2g18690 | -2.3 | -0.0 | -1.5 | C03:1..57781463:29786083-29787774 |
| TCONS_00035533 | 1-aminocyclopropane-1-carboxylate synthase | at4g11280 | -1.8 | 0.4 | -1.5 | C09:1..40126856:12934762-12936760 |
| TCONS_00031015 | TIFY 11A | at1g17380 | -2.1 | -0.4 | -1.5 | C08:1..41516064:20077291-20079028 |
| TCONS_00010565 | copper transport | at4g05030 | -3.1 | -0.6 | -1.5 | C03:1..57781463:17815308-17816011 |
| TCONS_00034425 | terpene synthase cyclase family | at1g48800 | -1.0 | -1.2 | -1.5 | C09:1..40126856:1371058-1372928 |
| TCONS_00013276 | At1g70420 F17O7 4 | at1g70420 | -2.5 | -0.1 | -1.5 | C03:1..57781463:55518243-55519261 |
| TCONS_00032864 | nucleotide-diphospho-sugar transferases superfamily | at2g19880 | -1.9 | -0.5 | -1.4 | C08:1..41516064:36126659-36128097 |
| TCONS_00003842 | uncharacterized LOC101216064 | at3g09032 | -1.2 | -0.3 | -1.4 | C01:1..38761720:37734325-37735044 |
| TCONS_00035248 | -NA- | at5g46910 | -2.8 | -0.9 | -1.4 | C09:1..40126856:8743271-8747039 |
| TCONS_00015379 | omega-3 fatty acid desaturase | p48624|fad3e_brana | -0.1 | 0.7 | -1.4 | C04:1..40895475:17289916-17290814 |
| TCONS_00003810 | phosphatidate phosphatase LPIN3-like | at3g09560 | -1.9 | -0.7 | -1.4 | C01:1..38761720:37483850-37485913 |
| TCONS_00012390 | Chromatin assembly factor 1 subunit | at1g28190 | -1.6 | -0.4 | -1.4 | C03:1..57781463:43921522-43922743 |
| TCONS_00026397 | -NA- | -- | -1.7 | -0.4 | -1.4 | C07:1..48346208:21172778-21173698 |
| TCONS_00037606 | unnamed protein product | at5g06980 | -1.2 | 0.1 | -1.4 | C09:1..40126856:37999425-38001141 |
| TCONS_00010932 | calcium-transporting ATPase plasma membrane-type-like | at3g21180 | -1.0 | -0.4 | -1.4 | C03:1..57781463:20915991-20916994 |
| TCONS_00021797 | calcium-binding CML38 | at1g76650 | -2.2 | -0.7 | -1.4 | C06:1..40704471:1525068-1526095 |
| TCONS_00011655 | Senescence dehydration-associated | at2g17840 | -1.4 | -0.4 | -1.4 | C03:1..57781463:29274568-29277236 |
| TCONS_00020294 | Coiled-coil domain-containing 109A | at1g09575 | -2.2 | -0.4 | -1.4 | C05:1..32828328:26145199-26147286 |
| TCONS_00019704 | lipoxygenase 5 | at3g22400 | -2.1 | -0.9 | -1.4 | C05:1..32828328:18816932-18819287 |
| TCONS_00005679 | hypothetical protein ARALYDRAFT 315868 | at1g68500 | -0.3 | 0.0 | -1.4 | C02:1..44046003:12243714-12244441 |
| TCONS_00029193 | heat shock transcription factor HSF4 | at4g36990 | -1.9 | 0.3 | -1.4 | C07:1..48346208:47544337-47545917 |
| TCONS_00005506 | heat shock 81-2 | at5g56030 | -1.7 | -0.3 | -1.4 | C02:1..44046003:10111612-10114630 |
| TCONS_00028388 | ethylene-responsive regulated nuclear | -- | -1.6 | -0.1 | -1.4 | C07:1..48346208:41880355-41880742 |
| TCONS_00002075 | P-glyco -2 | at4g25960 | -0.9 | -0.5 | -1.4 | C01:1..38761720:12065214-12066222 |
| TCONS_00002076 | ABC transporter B family member 2-like | at4g25960 | -1.2 | -0.4 | -1.4 | C01:1..38761720:12066331-12067116 |
| TCONS_00036410 | hypothetical protein CARUB v10000882mg | at5g22540 | -1.1 | -0.1 | -1.4 | C09:1..40126856:28807214-28808755 |
| TCONS_00031932 | manganese superoxide dismutase | at3g56350 | -2.5 | -0.3 | -1.4 | C08:1..41516064:30226943-30228499 |
| TCONS_00025481 | -NA- | at5g67180 | -1.4 | -0.0 | -1.4 | C07:1..48346208:1595813-1598823 |
| TCONS_00029724 | phospholipid glycerol acyltransferase family | at1g01610 | -1.3 | -0.8 | -1.4 | C08:1..41516064:41284076-41294724 |
| TCONS_00035948 | calcium-binding PBP1-like | at4g27280 | -1.2 | -0.5 | -1.3 | C09:1..40126856:22689797-22690704 |
| TCONS_00001291 | nuclease harbi1-like | at4g29780 | -1.5 | -0.6 | -1.3 | C01:1..38761720:4768503-4771076 |
| TCONS_00027577 | hypothetical protein CARUB v10027111mg | at5g48657 | -2.9 | -0.1 | -1.3 | C07:1..48346208:34876391-34878623 |
| TCONS_00029160 | aldehyde dehydrogenase family 3 member F1-like | at4g36250 | -0.8 | -0.1 | -1.3 | C07:1..48346208:47305267-47305797 |
| TCONS_00019706 | lipoxygenase | at3g22400 | -2.0 | -1.6 | -1.3 | C05:1..32828328:18820999-18822354 |
| TCONS_00003811 | phosphatidate phosphatase LPIN3-like | at3g09560 | -2.0 | -0.0 | -1.3 | C01:1..38761720:37486127-37487687 |
| TCONS_00021682 | pectin methyltransferase TSD2 | at1g78240 | -1.4 | -0.4 | -1.3 | C06:1..40704471:799436-800172 |
| TCONS_00007679 | 12-oxophytodienoate reductase 3-like | at2g06050 | -1.1 | -0.5 | -1.3 | C03:1..57781463:27298670-27303342 |
| TCONS_00009468 | cinnamate-4-hydroxylase | at2g30490 | -1.5 | -0.6 | -1.3 | C03:1..57781463:8130294-8141386 |
| TCONS_00010356 | phi-1-like phosphate-induced | at4g08950 | -1.1 | -0.1 | -1.3 | C03:1..57781463:15570328-15571749 |
| TCONS_00013968 | nodulin 21 -like transporter family | at2g40900 | -0.8 | -0.6 | -1.3 | C04:1..40895475:233170-233539 |
| TCONS_00005664 | unnamed protein product | at1g67920 | -2.6 | -0.9 | -1.3 | C02:1..44046003:11848783-11849327 |
| TCONS_00031019 | lipoxygenase | at1g17420 | -2.5 | -0.3 | -1.3 | C08:1..41516064:20153499-20188838 |
| TCONS_00030869 | salt tolerance-related | at1g13930 | 0.0 | 0.3 | -1.3 | C08:1..41516064:19145772-19146913 |
| TCONS_00012827 | phosphatase 2C | at2g30020 | -1.8 | -0.4 | -1.3 | C03:1..57781463:49699922-49701796 |
| TCONS_00012954 | cytochrome P450 | at1g57750 | -0.8 | 0.1 | -1.3 | C03:1..57781463:51832089-51833763 |
| TCONS_00019705 | lipoxygenase | at3g22400 | -2.0 | -1.5 | -1.3 | C05:1..32828328:18819424-18820874 |
| TCONS_00019551 | bidirectional sugar transporter SWEET1a-like | at1g21460 | -0.1 | 0.4 | -1.3 | C05:1..32828328:16784347-16786165 |
| TCONS_00008464 | cellulose synthase catalytic subunit | at5g09870 | -1.6 | -0.4 | -1.3 | C03:1..57781463:1819349-1820354 |
| TCONS_00009931 | Calmodulin | at2g41110 | -1.6 | -0.2 | -1.3 | C03:1..57781463:11838306-11839507 |
| TCONS_00013979 | VQ motif-containing | at2g41180 | -1.8 | -0.2 | -1.3 | C04:1..40895475:342072-343060 |
| TCONS_00019259 | ABC transporter G family member 11-like | at1g17840 | -0.4 | -0.8 | -1.3 | C05:1..32828328:13762253-13763101 |
| TCONS_00017905 | phospholipid glycerol acyltransferase family | at1g01610 | -0.5 | -0.1 | -1.3 | C05:1..32828328:62632-63429 |
| TCONS_00033351 | ACC oxidase | at1g62380 | -1.8 | 0.5 | -1.3 | C08:1..41516064:38823185-38824442 |
| TCONS_00015840 | GATA transcription factor 8 | at3g54810 | -1.8 | -0.4 | -1.3 | C04:1..40895475:23963440-23964910 |
| TCONS_00005265 | glycosyltransferase family 2 | at5g60700 | -2.8 | -0.3 | -1.3 | C02:1..44046003:6841038-6843852 |
| TCONS_00031043 | late embryogenesis abundant hydroxyproline-rich glyco | at1g17620 | -1.5 | -0.4 | -1.3 | C08:1..41516064:20533339-20534414 |
| TCONS_00021202 | glutathione S-transferase TAU 12 | at1g69920 | -1.8 | 0.0 | -1.3 | C06:1..40704471:5198955-5203064 |
| TCONS_00008561 | -NA- | at5g13660 | -2.1 | -0.1 | -1.3 | C03:1..57781463:2292451-2298823 |
| TCONS_00011793 | -NA- | -- | -2.7 | -0.5 | -1.3 | C03:1..57781463:30884342-30885637 |
| TCONS_00009544 | class IV chitinase | at2g43590 | -1.7 | 0.1 | -1.2 | C03:1..57781463:8645097-8645555 |
| TCONS_00020925 | dehydration-responsive element-binding 2A | at3g11020 | -3.3 | -0.6 | -1.2 | C05:1..32828328:31672872-31673618 |
| TCONS_00015594 | AP2 domain transcription factor | at2g33710 | -2.8 | -0.0 | -1.2 | C04:1..40895475:20351088-20353223 |
| TCONS_00002052 | xyloglucan endotransglucosylase hydrolase 22 | at4g25810 | -1.2 | -0.3 | -1.2 | C01:1..38761720:11886324-11887791 |
| TCONS_00021462 | major facilitator | at2g16990 | -0.2 | 0.2 | -1.2 | C06:1..40704471:28311735-28320202 |
| TCONS_00027404 | galacturonosyltransferase-like 10 | at3g28340 | -1.4 | -0.2 | -1.2 | C07:1..48346208:33491903-33493477 |
| TCONS_00003169 | polyol transporter 5-like | at3g18830 | -1.5 | -0.1 | -1.2 | C01:1..38761720:31139430-31143015 |
| TCONS_00036560 | ADIPOR-like receptor CG5315-like | at5g20270 | -0.6 | -0.7 | -1.2 | C09:1..40126856:30260644-30263218 |
| TCONS_00037492 | NAC domain-containing 102 | at5g08790 | -2.2 | -0.3 | -1.2 | C09:1..40126856:37611187-37612830 |
| TCONS_00036677 | inosine-uridine preferring nucleoside hydrolase family | at5g18860 | -1.6 | -0.4 | -1.2 | C09:1..40126856:31217814-31218815 |
| TCONS_00032867 | leucine-rich repeat family | at2g19780 | -2.1 | -0.5 | -1.2 | C08:1..41516064:36148732-36150751 |
| TCONS_00018901 | -NA- | at1g17030 | 0.8 | -0.1 | -1.2 | C05:1..32828328:6809943-6811824 |
| TCONS_00016583 | Kinase interacting (KIP1-like) family | at2g30500 | -0.9 | -0.5 | -1.2 | C04:1..40895475:33010778-33012563 |
| TCONS_00009401 | multidrug and toxin extrusion 2-like | at5g52050 | -2.8 | -0.0 | -1.2 | C03:1..57781463:7675814-7677807 |
| TCONS_00029156 | ferulate-5-hydroxylase | at4g36220 | -1.6 | -0.2 | -1.2 | C07:1..48346208:47280798-47282185 |
| TCONS_00037760 | myb family transcription factor | at5g05790 | -2.0 | -0.5 | -1.2 | C09:1..40126856:38974579-38976340 |
| TCONS_00014360 | cytochrome b5 | at2g46650 | -0.6 | 0.3 | -1.2 | C04:1..40895475:3282224-3283366 |
| TCONS_00023904 | aspartyl protease family | at3g59080 | -2.1 | -0.1 | -1.2 | C06:1..40704471:27199604-27201965 |
| TCONS_00017906 | -NA- | at1g01610 | -0.7 | -0.0 | -1.2 | C05:1..32828328:63978-65732 |
| TCONS_00021347 | pathogenesis-related | at1g78780 | -0.6 | 0.2 | -1.2 | C06:1..40704471:591165-601576 |
| TCONS_00028326 | heat stress transcription factor A-4a-like | at4g18880 | -2.5 | -0.3 | -1.2 | C07:1..48346208:41215080-41217249 |
| TCONS_00015689 | disease resistance | at2g34930 | -1.5 | -0.3 | -1.2 | C04:1..40895475:21467018-21467499 |
| TCONS_00013967 | nodulin 21 family | at2g40900 | -1.2 | -0.2 | -1.2 | C04:1..40895475:217050-232999 |
| TCONS_00014613 | WRKY transcription factor 33 | at2g38470 | -3.1 | -0.3 | -1.2 | C04:1..40895475:6218712-6221350 |
| TCONS_00021681 | probable pectin methyltransferase QUA2-like | at1g78240 | -1.1 | -0.6 | -1.2 | C06:1..40704471:796243-799298 |
| TCONS_00012158 | proton-dependent oligopeptide transport family | at3g47960 | -1.3 | -0.4 | -1.2 | C03:1..57781463:40018543-40020775 |
| TCONS_00014905 | -NA- | at3g57450 | -1.0 | -0.0 | -1.2 | C04:1..40895475:9589393-9590357 |
| TCONS_00028982 | WRKY transcription factor 11 | at4g31550 | -1.0 | -0.4 | -1.2 | C07:1..48346208:46097642-46099449 |
| TCONS_00001591 | reticuline oxidase-like protein | at4g20830 | -1.3 | -0.2 | -1.2 | C01:1..38761720:7481899-7484447 |
| TCONS_00017135 | nodulin family | at2g39210 | -1.2 | 0.2 | -1.2 | C04:1..40895475:38039637-38042458 |
| TCONS_00027963 | macrophage migration inhibitory factor homolog | at3g51660 | -0.7 | -0.0 | -1.2 | C07:1..48346208:37822356-37824491 |
| TCONS_00009556 | glucomannan 4-beta-mannosyltransferase 9-like | at2g35658 | -2.0 | -0.4 | -1.2 | C03:1..57781463:8735771-8736900 |
| TCONS_00018571 | phosphatase 2A regulatory subunit B eta | at1g13460 | -1.2 | -0.5 | -1.2 | C05:1..32828328:3972628-3981639 |
| TCONS_00001272 | -NA- | at4g29905 | -0.2 | 0.4 | -1.2 | C01:1..38761720:4700473-4740660 |
| TCONS_00002549 | heat shock 70 | at5g02500 | -1.3 | -0.3 | -1.1 | C01:1..38761720:20415594-20418365 |
| TCONS_00011838 | NAC domain-containing 62 | at3g49530 | -1.3 | -0.3 | -1.1 | C03:1..57781463:31316162-31318480 |
| TCONS_00029089 | phosphatase 2C family | at4g33920 | -1.3 | -0.3 | -1.1 | C07:1..48346208:46742381-46744637 |
| TCONS_00037394 | tyrosine phosphatase | at5g10695 | -1.8 | -0.2 | -1.1 | C09:1..40126856:36796090-36797647 |
| TCONS_00037188 | -NA- | at5g13220 | -2.5 | -0.0 | -1.1 | C09:1..40126856:35583686-35585860 |
| TCONS_00002785 | atp sulfurylase | at4g14680 | -1.3 | -0.2 | -1.1 | C01:1..38761720:24928393-24930732 |
| TCONS_00005870 | protein tify 11b | at1g72450 | -1.5 | -0.4 | -1.1 | C02:1..44046003:15839160-15841291 |
| TCONS_00001979 | protein | at4g25030 | -1.4 | -0.3 | -1.1 | C01:1..38761720:11253098-11255238 |
| TCONS_00010684 | systemic acquired resistance (SAR) regulator NIMIN-1 | at4g01897 | -0.8 | -0.0 | -1.1 | C03:1..57781463:19029661-19030498 |
| TCONS_00026208 | indole-3-acetic acid-amido synthetase | at1g28130 | -1.7 | -0.2 | -1.1 | C07:1..48346208:16556599-16558991 |
| TCONS_00023958 | methylenetetrahydrofolate reductase 1 | at3g59970 | -1.1 | 0.0 | -1.1 | C06:1..40704471:27683845-27684419 |
| TCONS_00022146 | hydroxyproline-rich glyco family | at1g72790 | -1.5 | -0.1 | -1.1 | C06:1..40704471:4025437-4027570 |
| TCONS_00032042 | guanylate kinase | at2g41880 | -1.4 | -0.4 | -1.1 | C08:1..41516064:30972962-30976514 |
| TCONS_00036958 | ---NA--- | at5g15490 | -1.7 | -0.2 | -1.1 | C09:1..40126856:33390095-33392410 |
| TCONS_00005214 | CCR4-associated factor 1 | at5g22250 | -1.1 | -0.2 | -1.1 | C02:1..44046003:6239297-6241550 |
| TCONS_00012105 | plant invertase pectin methylesterase inhibitor superfamily | at3g47380 | -1.8 | -0.1 | -1.1 | C03:1..57781463:39247243-39248260 |
| TCONS_00028358 | hypothetical protein EUTSA v10025579mg | -- | -1.1 | -0.2 | -1.1 | C07:1..48346208:41687640-41687816 |
| TCONS_00009286 | Rab3 GTPase-activating catalytic subunit isoform 1 | at5g55060 | -1.7 | -0.1 | -1.1 | C03:1..57781463:6471089-6471968 |
| TCONS_00022652 | probable calcium-binding CML23-like | at5g37770 | -1.1 | -0.4 | -1.1 | C06:1..40704471:8921022-8922068 |
| TCONS_00023108 | zinc finger | at3g53600 | -1.2 | -0.3 | -1.1 | C06:1..40704471:15890125-15891631 |
| TCONS_00027955 | probable galacturonosyltransferase-like 1-like | at3g50760 | -2.0 | 0.0 | -1.1 | C07:1..48346208:37754750-37756316 |
| TCONS_00028858 | -NA- | at4g29780 | -1.0 | -0.4 | -1.1 | C07:1..48346208:45327191-45329581 |
| TCONS_00019911 | -NA- | -- | -2.0 | -0.3 | -1.1 | C05:1..32828328:22345985-22359730 |
| TCONS_00020101 | tryptophan synthase beta chain chloroplastic-like | at5g28237 | -0.8 | -0.5 | -1.1 | C05:1..32828328:25099592-25100832 |
| TCONS_00005445 | xyloglucan endotransglycosylase hydrolase | at5g57560 | -1.7 | -0.7 | -1.1 | C02:1..44046003:9197431-9198539 |
| TCONS_00023372 | S-adenosyl-L-methionine-dependent methyltransferases superfamily | at1g55450 | -1.2 | -0.0 | -1.1 | C06:1..40704471:18796492-18798143 |
| TCONS_00023166 | ATP binding cassette subfamily B4 isoform 2 | at3g62150 | -1.4 | -0.5 | -1.1 | C06:1..40704471:16498132-16502650 |
| TCONS_00012363 | autoinhibited Ca2+-ATPase 1 | at1g27770 | -1.3 | -0.7 | -1.1 | C03:1..57781463:43772495-43776374 |
| TCONS_00011671 | P-loop containing nucleoside triphosphate hydrolases superfamily | at2g18193 | -1.8 | -0.8 | -1.1 | C03:1..57781463:29463527-29465253 |
| TCONS_00017098 | WRKY transcription factor 33 | at2g38470 | -1.4 | -0.0 | -1.1 | C04:1..40895475:37790388-37793626 |
| TCONS_00018898 | probable rhamnose biosynthetic enzyme 1-like | at1g78570 | -1.1 | -0.4 | -1.1 | C05:1..32828328:6799877-6802814 |
| TCONS_00001758 | cytochrome P450 | at4g22690 | -0.9 | -0.0 | -1.1 | C01:1..38761720:8992642-8994645 |
| TCONS_00031865 | -NA- | -- | -0.6 | -0.3 | -1.1 | C08:1..41516064:29715217-29715837 |
| TCONS_00006869 | cytosolic sulfotransferase 12-like | at2g03760 | -1.5 | -0.4 | -1.1 | C02:1..44046003:36642613-36644552 |
| TCONS_00012439 | WRKY transcription factor | at1g29280 | -1.5 | -0.3 | -1.1 | C03:1..57781463:44662892-44664575 |
| TCONS_00032018 | autoinhibited calcium ATPase | at3g57330 | -1.4 | -0.2 | -1.1 | C08:1..41516064:30783707-30787248 |
| TCONS_00030993 | -NA- | at1g16950 | -1.8 | -0.3 | -1.1 | C08:1..41516064:19888934-19889465 |
| TCONS_00014093 | pectinesterase family | at2g43050 | -1.3 | 0.3 | -1.1 | C04:1..40895475:1234292-1235621 |
| TCONS_00029155 | ferulate-5-hydroxylase | at4g36220 | -1.5 | 0.0 | -1.0 | C07:1..48346208:47279800-47280743 |
| TCONS_00024070 | GATA transcription factor 8 | at3g54810 | -1.8 | -0.4 | -1.0 | C06:1..40704471:29644586-29646909 |
| TCONS_00031139 | Sugar isoform 1 | at1g19380 | -0.8 | 0.1 | -1.0 | C08:1..41516064:21270658-21271628 |
| TCONS_00033201 | probable fucosyltransferase 4 | at1g14080 | -1.6 | 0.0 | -1.0 | C08:1..41516064:37968744-37970795 |
| TCONS_00012009 | E3 ubiquitin- ligase RING1-like | at3g46620 | -0.8 | -0.4 | -1.0 | C03:1..57781463:36090164-36091820 |
| TCONS_00027403 | zinc finger AN1 domain-containing stress-associated 12-like | at3g28210 | -1.5 | -0.2 | -1.0 | C07:1..48346208:33487606-33488800 |
| TCONS_00000487 | 3-deoxy-D-arabino-heptulosonate 7-phosphate synthase 1 | at4g39980 | -1.6 | -0.0 | -1.0 | C01:1..38761720:421862-424109 |
| TCONS_00023157 | kinase family | at3g61960 | -0.8 | -0.9 | -1.0 | C06:1..40704471:16385078-16386145 |
| TCONS_00029211 | -NA- | -- | -1.5 | -0.4 | -1.0 | C07:1..48346208:47659574-47660899 |
| TCONS_00032775 | -NA- | at2g21510 | -1.8 | -0.1 | -1.0 | C08:1..41516064:35708413-35710037 |
| TCONS_00016738 | glycine-rich 23 | at2g32690 | -0.7 | 0.0 | -1.0 | C04:1..40895475:34794634-34795836 |
| TCONS_00016712 | aceous RNase P chloroplastic mitochondrial-like | at2g32230 | -1.5 | -0.4 | -1.0 | C04:1..40895475:34583664-34584814 |
| TCONS_00023665 | abc transporter family | at1g51500 | -0.8 | -0.5 | -1.0 | C06:1..40704471:22773282-22776152 |
| TCONS_00007292 | -NA- | -- | -2.2 | 0.2 | -1.0 | C02:1..44046003:42560779-42562397 |
| TCONS_00029780 | UDP-D-apiose UDP-D-xylose synthase 1 | at2g27860 | -0.7 | -0.3 | -1.0 | C08:1..41516064:1186530-1186969 |
| TCONS_00026017 | domain-containing GPI-anchored | at2g17120 | -1.2 | -0.3 | -1.0 | C07:1..48346208:13021197-13023709 |
| TCONS_00013151 | WRKY transcription factor 11 | at4g31550 | -1.1 | -0.4 | -1.0 | C03:1..57781463:54127793-54129558 |
| TCONS_00014699 | kinase chloroplastic-like | at2g39660 | -0.8 | -0.4 | -1.0 | C04:1..40895475:7051958-7054133 |
| TCONS_00025476 | mitogen-activated kinase kinase kinase 19 | at5g67080 | -2.2 | -0.3 | -1.0 | C07:1..48346208:1488944-1490483 |
| TCONS_00037290 | -NA- | -- | -1.0 | -0.4 | -1.0 | C09:1..40126856:36198390-36199044 |
| TCONS_00011545 | adenylyl-sulfate kinase chloroplastic-like | at2g14750 | -0.9 | 0.6 | -1.0 | C03:1..57781463:27757846-27759849 |
| TCONS_00008929 | tetratricopeptide repeat domain-containing | at5g20190 | -1.5 | 0.0 | -1.0 | C03:1..57781463:4230417-4232596 |
| TCONS_00028470 | cytochrome P450 | at4g22690 | -2.5 | 0.4 | -1.0 | C07:1..48346208:42537003-42538885 |
| TCONS_00036725 | hypothetical protein EUTSA v10014852mg | at5g18310 | -1.5 | -0.4 | -1.0 | C09:1..40126856:31570162-31572155 |
| TCONS_00011052 | LURP-one-related 12-like | at3g15810 | -1.2 | -0.1 | -1.0 | C03:1..57781463:21713713-21715763 |
| TCONS_00011495 | 12-oxophytodienoate reductase 3 | at2g06050 | -1.0 | -0.1 | -1.0 | C03:1..57781463:27303595-27305258 |
| TCONS_00023793 | phospholipase C | at1g49740 | 0.3 | -0.1 | -1.0 | C06:1..40704471:25502970-25504741 |
| TCONS_00022243 | MATE efflux family | at1g71140 | -1.8 | -0.3 | -1.0 | C06:1..40704471:4438819-4440741 |
| TCONS_00010404 | cytochrome c-like | p00051|cyc_cucma | -1.2 | -0.1 | -1.0 | C03:1..57781463:16038752-16039697 |
| TCONS_00000759 | farnesylated protein | at4g35060 | -1.1 | -0.0 | -1.0 | C01:1..38761720:2014988-2016028 |
| TCONS_00031895 | zinc finger CCCH domain-containing 29 | at3g55980 | -0.8 | -0.2 | -1.0 | C08:1..41516064:29977580-29979857 |
| TCONS_00003463 | phosphoinositide phosphatase family | at3g14205 | -0.7 | 0.0 | -1.0 | C01:1..38761720:34767294-34770355 |
| TCONS_00018481 | phosphoglycerate bisphosphoglycerate mutase family | at1g08940 | -1.5 | -0.0 | -0.9 | C05:1..32828328:3283655-3284079 |
| TCONS_00023656 | 4-cumarate-COA-ligase | at1g51680 | -1.1 | -0.5 | -0.9 | C06:1..40704471:22688860-22692142 |
| TCONS_00031885 | Alpha beta hydrolase related | at3g55880 | -0.9 | -0.2 | -0.9 | C08:1..41516064:29907474-29910259 |
| TCONS_00006314 | glutathione s-transferase | at2g02930 | -1.4 | 0.1 | -0.9 | C02:1..44046003:24256420-24257704 |
| TCONS_00003282 | COBRA 7-like | at3g16860 | -1.2 | -0.3 | -0.9 | C01:1..38761720:32377297-32379480 |
| TCONS_00002275 | -NA- | -- | -1.8 | -1.8 | -Inf | C01:1..38761720:14061518-14061680 |
| TCONS_00005109 | -NA- | -- | -Inf | -1.3 | -Inf | C02:1..44046003:5413399-5413793 |
| TCONS_00014212 | beta-glucosidase 1 | at3g60120 | -1.7 | 0.7 | -Inf | C04:1..40895475:2163178-2163331 |

## Pyr-up

Table S2.3. Upregulated gene products after 8 days of Pyr treatment. Adjusted p-value <0.05. Gene name, as annotated by Blast2GO after best blastx in nr Viridiplantae database. Best Swiss-Prot hit (Uniprot manually annotated and reviewed protein database) as *Arabidopsis thaliana* locus name or Uniprot “Entry|Entry name” if best curated hit is from another species. ABA, Pyr and ABA+Pyr log2 fold change to control condition. Locus, *Brassica oleracea* v1.0 genomic locus (Liu et al. 2014).

| Isoform id | Gene name | Best Swiss-Prot hit | ABA | Pyr | ABA+Pyr | Locus |
| --- | --- | --- | --- | --- | --- | --- |
| TCONS_00001384 | -NA- | -- | -0.4 | 0.9 | 0.9 | C01:1..38761720:5694929-5696757 |
| TCONS_00014615 | lipid transfer | q42616|nltp3_brana | 0.7 | 1.0 | 1.8 | C04:1..40895475:6268407-6269306 |
| TCONS_00012813 | -NA- | at4g35750 | 0.9 | 1.1 | 1.7 | C03:1..57781463:49539338-49541103 |
| TCONS_00016846 | uncharacterized LOC101213935 | at2g34340 | 1.4 | 1.1 | 1.9 | C04:1..40895475:35681774-35682804 |
| TCONS_00034928 | AT5g25610 T14C9 150 | at5g25610 | -0.0 | 1.1 | 0.7 | C09:1..40126856:4757261-4758077 |
| TCONS_00035308 | major latex | at4g14060 | 0.0 | 1.1 | 2.0 | C09:1..40126856:9526862-9528076 |
| TCONS_00020642 | beta galactosidase 1 | at3g13750 | 2.1 | 1.1 | 2.1 | C05:1..32828328:29310627-29313468 |
| TCONS_00025521 | Late embryogenesis abundant group 2 | at3g24600 | 1.0 | 1.1 | 0.4 | C07:1..48346208:2940522-2941423 |
| TCONS_00034929 | -NA- | at5g25610 | -0.0 | 1.2 | 0.8 | C09:1..40126856:4758255-4759572 |
| TCONS_00007341 | AT5g25610 T14C9 150 | at5g25610 | -0.6 | 1.2 | 0.8 | C02:1..44046003:43100085-43100882 |
| TCONS_00010057 | F-box kelch-repeat | at2g44130 | 0.6 | 1.2 | 1.0 | C03:1..57781463:12888824-12890684 |
| TCONS_00024085 | anthranilate N-hydroxycinnamoyl benzoyltransferase | at5g67150 | 0.9 | 1.2 | 1.7 | C06:1..40704471:29827587-29829100 |
| TCONS_00034007 | peroxidase | at5g06730 | 0.1 | 1.2 | -0.2 | C09:1..40126856:38430093-38440694 |
| TCONS_00037634 | peptide methionine sulfoxide reductase | at5g07460 | -0.7 | 1.2 | -2.1 | C09:1..40126856:38231054-38232157 |
| TCONS_00004886 | -NA- | -- | -0.1 | 1.2 | 0.8 | C02:1..44046003:3725895-3726446 |
| TCONS_00031129 | -NA- | -- | -0.2 | 1.3 | -0.4 | C08:1..41516064:21108576-21109785 |
| TCONS_00007340 | dehydration-responsive RD22 | at5g25610 | -0.5 | 1.3 | 0.9 | C02:1..44046003:43098312-43099895 |
| TCONS_00015483 | Ent-kaurenoic acid oxidase 2 | at2g32440 | 0.4 | 1.3 | 0.5 | C04:1..40895475:19082863-19084034 |
| TCONS_00030870 | salt tolerance-related | at1g13930 | 0.1 | 1.3 | 0.6 | C08:1..41516064:19150964-19151735 |
| TCONS_00020872 | S-adenosylmethionine synthetase | at3g17390 | -1.6 | 1.3 | -1.8 | C05:1..32828328:31126473-31127979 |
| TCONS_00037766 | anthranilate synthase component I-1 | at5g05730 | -0.5 | 1.4 | -1.6 | C09:1..40126856:39024323-39026115 |
| TCONS_00034930 | AT5g25610 T14C9 150 | -- | 0.2 | 1.4 | 1.3 | C09:1..40126856:4760089-4760671 |
| TCONS_00032012 | beta-1,3-glucanase 2 | at3g57260 | -0.6 | 1.4 | -2.8 | C08:1..41516064:30761349-30765468 |
| TCONS_00019748 | nitrate transporter | at3g21670 | -0.4 | 1.4 | 0.7 | C05:1..32828328:19456057-19457965 |
| TCONS_00030419 | -NA- | at4g15990 | 2.5 | 1.5 | 1.1 | C08:1..41516064:13829680-13830366 |
| TCONS_00015482 | Ent-kaurenoic acid oxidase 2 | at2g32440 | 0.6 | 1.5 | 0.9 | C04:1..40895475:19081609-19082761 |
| TCONS_00037743 | cytochrome P450 | at2g30750 | -0.3 | 1.5 | -1.9 | C09:1..40126856:38893046-38895333 |
| TCONS_00031664 | glucosyl transferase | at3g53160 | -0.6 | 1.5 | -2.7 | C08:1..41516064:28212386-28214109 |
| TCONS_00013926 | protease inhibitor | at2g38870 | 0.0 | 1.7 | -1.4 | C04:1..40895475:37954208-37959375 |
| TCONS_00026477 | -NA- | -- | 1.4 | 1.8 | 2.0 | C07:1..48346208:22730167-22731441 |
| TCONS_00000561 | hypothetical protein CARUB v10007485mg | -- | -0.3 | 2.0 | -1.9 | C01:1..38761720:898989-899581 |
| TCONS_00008339 | steroid sulfotransferase | at5g07010 | 0.2 | 2.0 | 1.4 | C03:1..57781463:1241918-1243230 |
| TCONS_00034744 | alpha-helical IF | at5g26770 | -0.6 | 2.1 | -1.0 | C09:1..40126856:3433131-3434380 |
| TCONS_00006759 | glutamate decarboxylase | at2g02010 | -0.0 | 2.1 | -2.9 | C02:1..44046003:34684126-34686394 |
| TCONS_00012660 | cytochrome P450 [Arabidopsis thaliana] | o81345|c79b1_sinal | -0.6 | 2.1 | -1.6 | C03:1..57781463:46728170-46729086 |
| TCONS_00017138 | jacalin lectin family | at2g39310 | 0.8 | 2.4 | -0.5 | C04:1..40895475:38061964-38064182 |
| TCONS_00026244 | GDSL esterase lipase | at1g28640 | 0.3 | 2.5 | 0.6 | C07:1..48346208:17059802-17069269 |
| TCONS_00006248 | high-affinity k+ transporter partial | at4g10310 | -0.4 | 3.8 | 2.6 | C02:1..44046003:22401828-22405054 |

## Pyr-down

Table S2.4. Downregulated gene products after 8 days of Pyr treatment. Adjusted p-value <0.05. Gene name, as annotated by Blast2GO after best blastx in nr Viridiplantae database. Best Swiss-Prot hit (Uniprot manually annotated and reviewed protein database) as *Arabidopsis thaliana* locus name or Uniprot “Entry|Entry name” if best curated hit is from another species. ABA, Pyr and ABA+Pyr log2 fold change to control condition. Locus, *Brassica oleracea* v1.0 genomic locus (Liu et al. 2014).

| Isoform id | Gene name | Best Swiss-Prot hit | ABA | Pyr | ABA+Pyr | Locus |
| --- | --- | --- | --- | --- | --- | --- |
| TCONS_00034803 | hypothetical protein EUTSA v10005448mg | at5g61340 | -3.1 | -2.8 | -1.7 | C09:1..40126856:3899487-3900662 |
| TCONS_00019915 | cytochrome P450 | at3g20140 | -2.6 | -2.4 | -1.7 | C05:1..32828328:22421879-22424258 |
| TCONS_00035903 | -NA- | -- | -0.7 | -1.8 | 0.3 | C09:1..40126856:21918316-21919669 |
| TCONS_00017219 | -NA- | -- | -1.2 | -1.7 | 0.1 | C04:1..40895475:38695446-38696801 |
| TCONS_00008152 | -NA- | -- | -0.9 | -1.6 | 0.4 | C03:1..57781463:174702-175894 |
| TCONS_00019706 | lipoxygenase | at3g22400 | -2.0 | -1.6 | -1.3 | C05:1..32828328:18820999-18822354 |
| TCONS_00033004 | lipoxygenase 3 | at1g17420 | -3.0 | -1.6 | -2.5 | C08:1..41516064:36874625-36876372 |
| TCONS_00019705 | lipoxygenase | at3g22400 | -2.0 | -1.5 | -1.3 | C05:1..32828328:18819424-18820874 |
| TCONS_00030926 | ATPDR7 PDR7 | at1g15210 | -2.8 | -1.5 | -1.9 | C08:1..41516064:19529552-19530582 |
| TCONS_00033731 | 3-ketoacyl- synthase 1 | at1g01120 | -1.5 | -1.3 | -1.7 | C08:1..41516064:41177474-41179315 |
| TCONS_00017000 | phenylalanine ammonia-lyase | at2g37040 | -2.2 | -1.3 | -2.6 | C04:1..40895475:37110384-37112545 |
| TCONS_00030925 | ATPDR7 PDR7 | at1g15210 | -3.7 | -1.2 | -2.2 | C08:1..41516064:19527996-19529428 |
| TCONS_00008279 | leucoanthocyanidin dioxygenase | at5g05600 | -3.1 | -1.2 | -2.6 | C03:1..57781463:899339-902214 |
| TCONS_00034777 | -NA- | -- | -1.0 | -1.2 | -0.0 | C09:1..40126856:3667698-3668831 |
| TCONS_00031977 | metal ion binding | at3g56891 | -2.6 | -1.2 | -2.0 | C08:1..41516064:30515866-30516976 |
| TCONS_00009282 | O-Glycosyl hydrolases family 17 | at5g55180 | -2.6 | -1.1 | -2.2 | C03:1..57781463:6438266-6440020 |
| TCONS_00007931 | -NA- | at1g01680 | -3.2 | -1.1 | -1.9 | C03:1..57781463:13544901-13549304 |
| TCONS_00003168 | -NA- | -- | -4.1 | -Inf | -0.9 | C01:1..38761720:31119516-31120093 |
| TCONS_00014561 | -NA- | -- | -Inf | -Inf | -2.8 | C04:1..40895475:5485455-5485877 |
| TCONS_00027391 | DNA repair UVH3 | at3g28030 | -1.8 | -Inf | -1.3 | C07:1..48346208:33385279-33385445 |
| TCONS_00030479 | mitochondrial substrate carrier family | -- | 0.8 | -Inf | -0.2 | C08:1..41516064:14578517-14578773 |
| TCONS_00034839 | cysteine-type peptidase | at2g07240 | -1.4 | -Inf | -1.3 | C09:1..40126856:4110295-4112057 |
| TCONS_00035048 | -NA- | -- | -1.3 | -Inf | -2.0 | C09:1..40126856:5797988-5798195 |
| TCONS_00035087 | -NA- | -- | -1.2 | -Inf | -0.7 | C09:1..40126856:6212518-6213019 |

## ABA+Pyr-up

Table S2.5. Upregulated gene products after 8 days of ABA+Pyr treatment. Adjusted p-value <0.05. Gene name, as annotated by Blast2GO after best blastx in nr Viridiplantae database. Best Swiss-Prot hit (Uniprot manually annotated and reviewed protein database) as *Arabidopsis thaliana* locus name or Uniprot “Entry|Entry name” if best curated hit is from another species. ABA, Pyr and ABA+Pyr log2 fold change to control condition. Locus, *Brassica oleracea* v1.0 genomic locus (Liu et al. 2014).

| Isoform id | Gene name | Best Swiss-Prot hit | ABA | Pyr | ABA+Pyr | Locus |
| --- | --- | --- | --- | --- | --- | --- |
| TCONS_00004290 | -NA- | -- | Inf | 0 | Inf | C02:1..44046003:34492174-34494714 |
| TCONS_00005704 | -NA- | -- | Inf | Inf | Inf | C02:1..44046003:12630531-12630921 |
| TCONS_00015949 | -NA- | -- | Inf | Inf | Inf | C04:1..40895475:25686955-25687444 |
| TCONS_00034867 | -NA- | -- | Inf | Inf | Inf | C09:1..40126856:4264248-4264387 |
| TCONS_00037440 | cytochrome P450 | at5g09970 | Inf | Inf | Inf | C09:1..40126856:37357251-37358077 |
| TCONS_00005692 | probable nitrite transporter at1g68570-like | at1g68570 | 3.4 | 1.8 | 2.7 | C02:1..44046003:12441554-12443162 |
| TCONS_00030808 | ERF AP2 transcription factor DDF2 | at1g12610 | 3.0 | -0.7 | -1.3 | C08:1..41516064:18643323-18644192 |
| TCONS_00011791 | root phototropism 3 | at5g64330 | 2.9 | -0.1 | 1.5 | C03:1..57781463:30879140-30879766 |
| TCONS_00006786 | -NA- | -- | 2.9 | 0.7 | 2.6 | C02:1..44046003:35229522-35230321 |
| TCONS_00004418 | acid phosphatase 1-like | at5g44020 | 2.9 | 1.1 | 1.7 | C02:1..44046003:918945-919957 |
| TCONS_00006248 | high-affinity k+ transporter partial | at4g10310 | 2.6 | 3.8 | -0.4 | C02:1..44046003:22401828-22405054 |
| TCONS_00014385 | hypothetical protein EUTSA v10001588mg | at2g46940 | 2.6 | 0.3 | 1.5 | C04:1..40895475:3536561-3537501 |
| TCONS_00011790 | root phototropism 3 | at5g64330 | 2.6 | 0.3 | 1.9 | C03:1..57781463:30877709-30878657 |
| TCONS_00015738 | uncharacterized LOC101212292 | at2g35470 | 2.5 | 1.2 | 1.7 | C04:1..40895475:21799391-21800533 |
| TCONS_00007358 | -NA- | at5g25240 | 2.5 | 0.8 | 1.8 | C02:1..44046003:43332227-43332827 |
| TCONS_00023321 | plant invertase pectin methylesterase inhibitor superfamily | at5g38610 | 2.5 | 0.8 | 0.6 | C06:1..40704471:18176000-18176894 |
| TCONS_00031672 | -NA- | -- | 2.5 | 0.5 | 2.1 | C08:1..41516064:28298418-28298862 |
| TCONS_00035479 | uncharacterized LOC101209587 | at4g04630 | 2.4 | 0.9 | 1.8 | C09:1..40126856:11932164-11933322 |
| TCONS_00000609 | PQ-loop repeat family transmembrane family | at4g36850 | 2.4 | 0.7 | 2.1 | C01:1..38761720:1135327-1135758 |
| TCONS_00005658 | GDSL esterase lipase At3g26430-like | at1g67830 | 2.3 | 0.9 | 2.6 | C02:1..44046003:11763489-11764451 |
| TCONS_00014657 | -NA- | -- | 2.3 | 0.7 | 1.5 | C04:1..40895475:6651887-6652298 |
| TCONS_00010597 | TRANSPORT INHIBITOR RESPONSE 1 | at4g03190 | 2.3 | 1.4 | 1.7 | C03:1..57781463:18196360-18197099 |
| TCONS_00024369 | walls are thin 1 | at1g75500 | 2.3 | 0.7 | 1.8 | C06:1..40704471:32252181-32254110 |
| TCONS_00023200 | -NA- | at3g63090 | 2.3 | 0.7 | 0.8 | C06:1..40704471:16846526-16848062 |
| TCONS_00025651 | -NA- | -- | 2.3 | 1.2 | 1.8 | C07:1..48346208:4759840-4761209 |
| TCONS_00022005 | geranylgeranyl reductase | at1g74470 | 2.3 | 0.5 | 1.9 | C06:1..40704471:3137574-3138633 |
| TCONS_00000425 | fructose bisphosphate aldolase | at4g38970 | 2.3 | 0.3 | 1.9 | C01:1..38761720:52447-52934 |
| TCONS_00022807 | alpha beta-Hydrolases superfamily | at1g52700 | 2.2 | -0.1 | -0.1 | C06:1..40704471:10946856-10948196 |
| TCONS_00000426 | fructose bisphosphate aldolase | at4g38970 | 2.2 | 0.0 | 1.4 | C01:1..38761720:53262-53619 |
| TCONS_00003224 | lipid transfer protein | at3g18280 | 2.2 | 0.0 | 2.5 | C01:1..38761720:31635212-31635810 |
| TCONS_00027195 | ROTUNDIFOLIA like 15 | -- | 2.2 | 0.3 | 1.6 | C07:1..48346208:31588895-31589631 |
| TCONS_00035791 | enhancer of triptychon and caprice 2 | -- | 2.2 | 0.7 | 1.1 | C09:1..40126856:19967147-19968412 |
| TCONS_00010041 | formin 1-like | at2g43800 | 2.2 | 1.1 | 1.3 | C03:1..57781463:12776145-12777185 |
| TCONS_00014324 | peroxisomal biogenesis factor 11 family | at2g45740 | 2.2 | 0.1 | 1.7 | C04:1..40895475:3039346-3039863 |
| TCONS_00011789 | root phototropism 3-like | at5g64330 | 2.2 | 0.3 | 1.6 | C03:1..57781463:30876465-30877563 |
| TCONS_00037080 | Lysine ketoglutarate reductase trans-splicing | at1g61240 | 2.1 | 0.6 | 1.3 | C09:1..40126856:34777084-34777853 |
| TCONS_00024900 | senescence-associated 5 | at2g19580 | 2.1 | 0.7 | 1.4 | C07:1..48346208:7630862-7639178 |
| TCONS_00030357 | acylamino acid-releasing enzyme | at4g14570 | 2.1 | 0.1 | 1.4 | C08:1..41516064:12324341-12325399 |
| TCONS_00000424 | fructose bisphosphate aldolase | at4g38970 | 2.1 | 0.3 | 1.8 | C01:1..38761720:51222-52330 |
| TCONS_00020642 | beta galactosidase 1 | at3g13750 | 2.1 | 1.1 | 2.1 | C05:1..32828328:29310627-29313468 |
| TCONS_00006541 | gdsl esterase lipase at5g45960-like | at5g45960 | 2.1 | 0.9 | 1.9 | C02:1..44046003:31359735-31363539 |
| TCONS_00020182 | -NA- | at5g51470 | 2.1 | -0.0 | 1.6 | C05:1..32828328:25556392-25557162 |
| TCONS_00019084 | phosphoribulokinase precursor | at1g32060 | 2.0 | 0.0 | 1.8 | C05:1..32828328:10791474-10792284 |
| TCONS_00015099 | -NA- | -- | 2.0 | 0.3 | 1.5 | C04:1..40895475:13306532-13307549 |
| TCONS_00037079 | -NA- | at1g61240 | 2.0 | 0.3 | 1.3 | C09:1..40126856:34775880-34776925 |
| TCONS_00026477 | -NA- | -- | 2.0 | 1.8 | 1.4 | C07:1..48346208:22730167-22731441 |
| TCONS_00022808 | phospholipase carboxylesterase family | at1g52700 | 2.0 | 0.5 | -0.2 | C06:1..40704471:10948478-10950207 |
| TCONS_00014387 | transcription factor PIF6 | at2g46970 | 2.0 | -0.3 | 1.1 | C04:1..40895475:3544378-3546287 |
| TCONS_00006617 | protein | -- | 2.0 | 0.3 | 1.5 | C02:1..44046003:32304165-32304700 |
| TCONS_00035308 | major latex | at4g14060 | 2.0 | 1.1 | 0.0 | C09:1..40126856:9526862-9528076 |
| TCONS_00019007 | hypothetical protein EUTSA v10009221mg | -- | 1.9 | 0.8 | 0.9 | C05:1..32828328:9468189-9469158 |
| TCONS_00005491 | beta-galactosidase | at4g26140 | 1.9 | 0.8 | 1.8 | C02:1..44046003:9988376-9989000 |
| TCONS_00007244 | BTB POZ domain-containing At5g67385-like | at5g67385 | 1.9 | 0.8 | 1.7 | C02:1..44046003:42107126-42107770 |
| TCONS_00026041 | ubiquitin-conjugating enzyme E2 29 | at2g16740 | 1.9 | 0.9 | -0.0 | C07:1..48346208:13556726-13557804 |
| TCONS_00015077 | NDH-dependent cyclic electron flow 1 | at1g64770 | 1.9 | 0.0 | 1.4 | C04:1..40895475:12386090-12387208 |
| TCONS_00028645 | Beta-galactosidase related | -- | 1.9 | 1.1 | 1.0 | C07:1..48346208:43860675-43861698 |
| TCONS_00014656 | vacuolar amino acid transporter 1-like | at2g39130 | 1.9 | 0.5 | 1.1 | C04:1..40895475:6650885-6651820 |
| TCONS_00018427 | magnesium-chelatase subunit chloroplastic-like | o24133|chld_tobac | 1.9 | 0.4 | 1.6 | C05:1..32828328:3101304-3102265 |
| TCONS_00033375 | monocysteinic thioredoxin CXXS1 | at1g11530 | 1.9 | 0.4 | 1.3 | C08:1..41516064:39013252-39014377 |
| TCONS_00015076 | NDH-dependent cyclic electron flow 1 | at1g64770 | 1.9 | 0.9 | 1.3 | C04:1..40895475:12385240-12385633 |
| TCONS_00024569 | auxin efflux carrier component 7 | at1g70940 | 1.9 | 0.4 | 1.3 | C06:1..40704471:34592492-34595387 |
| TCONS_00015330 | -NA- | -- | 1.9 | 1.3 | 0.7 | C04:1..40895475:16485554-16487047 |
| TCONS_00034567 | transferase family | at3g29590 | 1.9 | 0.5 | 1.1 | C09:1..40126856:2386555-2388406 |
| TCONS_00035311 | -NA- | -- | 1.9 | 0.4 | 1.5 | C09:1..40126856:9646718-9647609 |
| TCONS_00016846 | uncharacterized LOC101213935 | at2g34340 | 1.9 | 1.1 | 1.4 | C04:1..40895475:35681774-35682804 |
| TCONS_00007726 | nonphototropic hypocotyl 1 | at3g45780 | 1.9 | 0.1 | 0.9 | C03:1..57781463:41333836-41337009 |
| TCONS_00005348 | lipid transfer protein precursor | at5g59320 | 1.9 | 0.6 | 0.7 | C02:1..44046003:7999562-8000572 |
| TCONS_00028533 | -NA- | at4g23870 | 1.8 | 1.1 | 2.2 | C07:1..48346208:43270642-43271791 |
| TCONS_00036609 | peptidase M28 family | at5g19740 | 1.8 | 0.5 | 0.9 | C09:1..40126856:30547903-30548838 |
| TCONS_00012221 | phototropin 1 | at3g45780 | 1.8 | 0.1 | 0.7 | C03:1..57781463:41337516-41338115 |
| TCONS_00012483 | photosystem II type I chlorophyll a b binding | at1g29930 | 1.8 | 0.4 | 2.1 | C03:1..57781463:45048040-45049388 |
| TCONS_00022618 | clathrin assembly | at1g68110 | 1.8 | 1.1 | 1.6 | C06:1..40704471:8559328-8560842 |
| TCONS_00023376 | ZKT containing K-box and a TPR region | at1g55480 | 1.8 | -0.1 | 1.9 | C06:1..40704471:18816744-18817108 |
| TCONS_00026258 | hypothetical protein CARUB v10012307mg, partial | at1g29270 | 1.8 | 0.3 | 0.1 | C07:1..48346208:17469491-17470543 |
| TCONS_00014615 | lipid transfer | q42616|nltp3_brana | 1.8 | 1.0 | 0.7 | C04:1..40895475:6268407-6269306 |
| TCONS_00005136 | phd finger family protein | at5g20510 | 1.8 | 0.8 | 1.5 | C02:1..44046003:5505594-5507408 |
| TCONS_00018115 | cryptochrome 2 | p40115|phr1_sinal | 1.8 | 0.5 | 1.5 | C05:1..32828328:1187692-1188687 |
| TCONS_00003036 | dof zinc finger | at3g21270 | 1.8 | 0.6 | 0.9 | C01:1..38761720:29136079-29137493 |
| TCONS_00031829 | Rossmann-fold NAD(P)-binding domain-containing | at3g55290 | 1.8 | 0.6 | 1.5 | C08:1..41516064:29435514-29436942 |
| TCONS_00011573 | maternal effect embryo arrest 14 | at2g15890 | 1.8 | 0.1 | 2.0 | C03:1..57781463:28159219-28160964 |
| TCONS_00029014 | aspartate aminotransferase | at4g31990 | 1.8 | 0.4 | 1.4 | C07:1..48346208:46256249-46256825 |
| TCONS_00016664 | -NA- | -- | 1.8 | -0.0 | -0.1 | C04:1..40895475:33995903-33998717 |
| TCONS_00001097 | tetratricopeptide repeat domain-containing protein | at4g32340 | 1.8 | 0.1 | 1.1 | C01:1..38761720:3396049-3397962 |
| TCONS_00019736 | peroxidase 30 | at3g21770 | 1.8 | 0.3 | 0.2 | C05:1..32828328:19231790-19232855 |
| TCONS_00012601 | fructose bisphosphate aldolase | at4g38970 | 1.8 | 0.7 | 1.7 | C03:1..57781463:46078595-46079467 |
| TCONS_00035141 | AF375430 1 AT5g45310 K9E15 9 | at5g45310 | 1.8 | 0.7 | 1.6 | C09:1..40126856:7068212-7070127 |
| TCONS_00012220 | -NA- | at3g45780 | 1.8 | 0.3 | 0.9 | C03:1..57781463:41332760-41333762 |
| TCONS_00001883 | metal-nicotianamine transporter ysl1-like | at4g24120 | 1.8 | 0.4 | 0.5 | C01:1..38761720:10772117-10775071 |
| TCONS_00012223 | phototropin 1 | at3g45780 | 1.8 | 0.6 | 1.2 | C03:1..57781463:41339429-41339960 |
| TCONS_00000754 | plasma membrane intrinsic protein | at4g35100 | 1.8 | 0.1 | 1.6 | C01:1..38761720:1981818-1983621 |
| TCONS_00023393 | FAD NAD(P)-binding oxidoreductase domain-containing | at1g55980 | 1.8 | 0.1 | 1.1 | C06:1..40704471:18998732-18999416 |
| TCONS_00035443 | -NA- | -- | 1.7 | 0.6 | 1.1 | C09:1..40126856:11606447-11607555 |
| TCONS_00034564 | AT3g29240 MXO21 9 | at3g29240 | 1.7 | 0.8 | 2.1 | C09:1..40126856:2370229-2371432 |
| TCONS_00003329 | sulfate transporter Sultr3 4 | at3g15990 | 1.7 | 0.5 | 0.8 | C01:1..38761720:33036866-33038589 |
| TCONS_00034932 | hypothetical protein EUTSA v10004607mg | at5g67370 | 1.7 | -0.0 | 1.1 | C09:1..40126856:4831935-4832570 |
| TCONS_00022475 | nitrate transporter -like | at1g69850 | 1.7 | 0.7 | 1.1 | C06:1..40704471:7060845-7061668 |
| TCONS_00030360 | acylamino acid-releasing enzyme | at4g14570 | 1.7 | 0.2 | 1.3 | C08:1..41516064:12326750-12327479 |
| TCONS_00008124 | one helix | at5g02120 | 1.7 | 0.7 | 0.9 | C03:1..57781463:49636-49793 |
| TCONS_00013986 | -NA- | -- | 1.7 | 0.9 | 0.5 | C04:1..40895475:422853-423148 |
| TCONS_00024085 | anthranilate N-hydroxycinnamoyl benzoyltransferase | at5g67150 | 1.7 | 1.2 | 0.9 | C06:1..40704471:29827587-29829100 |
| TCONS_00032433 | hypothetical protein EUTSA v10006025mg | at3g62630 | 1.7 | 0.6 | 1.4 | C08:1..41516064:33260443-33262113 |
| TCONS_00003351 | mutator-like transposase | -- | 1.7 | 0.1 | 1.5 | C01:1..38761720:33178169-33179131 |
| TCONS_00012813 | -NA- | at4g35750 | 1.7 | 1.1 | 0.9 | C03:1..57781463:49539338-49541103 |
| TCONS_00027624 | probable membrane-associated kinase regulator 1-like | at5g26230 | 1.7 | 0.2 | 1.0 | C07:1..48346208:35264958-35266275 |
| TCONS_00019083 | phosphoribulokinase precursor | at1g32060 | 1.7 | 0.4 | 1.8 | C05:1..32828328:10790167-10791025 |
| TCONS_00005992 | ATP-dependent protease La domain-containing | at1g75460 | 1.7 | 0.8 | 1.8 | C02:1..44046003:18056956-18058455 |
| TCONS_00011624 | RING-H2 finger RHA3b | at2g17450 | 1.7 | 0.9 | 1.4 | C03:1..57781463:28934157-28935040 |
| TCONS_00007351 | uncharacterized LOC101214063 | at5g25265 | 1.7 | 0.8 | 1.2 | C02:1..44046003:43310410-43310922 |
| TCONS_00026585 | -NA- | -- | 1.7 | 0.6 | 1.4 | C07:1..48346208:24327004-24328161 |
| TCONS_00026122 | zinc finger family | at2g15580 | 1.7 | 0.4 | 1.2 | C07:1..48346208:15093569-15094822 |
| TCONS_00015753 | plastid-specific 50S ribosomal 5 | at3g56910 | 1.7 | 0.9 | 1.4 | C04:1..40895475:21975343-21976308 |
| TCONS_00024782 | lysine histidine transporter-like 8-like | at1g47670 | 1.7 | -0.3 | -0.1 | C06:1..40704471:39418709-39420563 |
| TCONS_00010964 | -NA- | at3g13130 | 1.7 | 0.8 | 1.3 | C03:1..57781463:21028463-21029340 |
| TCONS_00035438 | Wound-responsive family | at4g05070 | 1.7 | 0.2 | 1.4 | C09:1..40126856:11528956-11529684 |
| TCONS_00010577 | M-type thioredoxin | q9xgs0|trxm_brana | 1.7 | 0.1 | 1.5 | C03:1..57781463:17927257-17928399 |
| TCONS_00030949 | Chlorophyll a-b binding CP24 chloroplastic | at1g15820 | 1.7 | 0.8 | 1.5 | C08:1..41516064:19680658-19681755 |
| TCONS_00019755 | AT3g21550 MIL23 11 | at3g21550 | 1.7 | 0.5 | -1.1 | C05:1..32828328:19524401-19525263 |
| TCONS_00026846 | potassium channel KAT1 | at5g46240 | 1.7 | 0.3 | 0.0 | C07:1..48346208:28013877-28015124 |
| TCONS_00022514 | S-adenosyl-L-methionine-dependent methyltransferases superfamily | at1g69523 | 1.7 | 0.4 | 1.1 | C06:1..40704471:7420085-7420864 |
| TCONS_00031924 | -NA- | -- | 1.7 | 0.2 | 0.7 | C08:1..41516064:30205672-30206376 |
| TCONS_00012602 | fructose-bisphosphate aldolase 2 | at4g38970 | 1.7 | 0.5 | 1.1 | C03:1..57781463:46079895-46080728 |
| TCONS_00008626 | EG2771 | at5g14910 | 1.6 | 0.3 | 1.5 | C03:1..57781463:2612563-2614303 |
| TCONS_00022004 | geranylgeranyl reductase | at1g74470 | 1.6 | 0.5 | 1.5 | C06:1..40704471:3136324-3137398 |
| TCONS_00001702 | peroxidase | at4g21960 | 1.6 | 0.4 | 1.8 | C01:1..38761720:8422164-8423921 |
| TCONS_00036123 | H(+)-ATPase 3 | at5g57350 | 1.6 | 0.0 | -0.4 | C09:1..40126856:25131756-25132567 |
| TCONS_00036261 | AT5g58960 k19m22 160 | at5g58960 | 1.6 | 0.6 | 1.1 | C09:1..40126856:26714539-26716635 |
| TCONS_00007243 | BTB POZ domain-containing At5g67385-like | at5g67385 | 1.6 | 0.5 | 1.3 | C02:1..44046003:42106309-42106881 |
| TCONS_00037386 | argininosuccinate lyase | at5g10920 | 1.6 | 0.3 | 1.0 | C09:1..40126856:36676117-36677052 |
| TCONS_00016755 | TRAF-like family | at2g32880 | 1.6 | 0.5 | 1.1 | C04:1..40895475:34889309-34891157 |
| TCONS_00004401 | golden2-like transcription factor | at5g44190 | 1.6 | 1.0 | 0.9 | C02:1..44046003:675704-676557 |
| TCONS_00014655 | vacuolar amino acid transporter 1-like | at2g39130 | 1.6 | 0.6 | 1.0 | C04:1..40895475:6647579-6650715 |
| TCONS_00006715 | abc transporter g family member 7 | at2g01320 | 1.6 | 0.3 | 1.4 | C02:1..44046003:34164839-34166632 |
| TCONS_00004400 | transcription activator glk1-like | at5g44190 | 1.6 | 1.0 | 1.4 | C02:1..44046003:673951-675376 |
| TCONS_00014241 | -NA- | at2g44670 | 1.6 | 0.3 | 0.9 | C04:1..40895475:2412152-2413012 |
| TCONS_00030361 | -NA- | -- | 1.6 | -0.0 | 1.3 | C08:1..41516064:12328206-12328669 |
| TCONS_00024568 | auxin efflux carrier component 4 | at1g70940 | 1.6 | 0.2 | 1.6 | C06:1..40704471:34590503-34592228 |
| TCONS_00034263 | kinase family | at4g01330 | 1.6 | 0.8 | 1.5 | C09:1..40126856:259721-261217 |
| TCONS_00034264 | kinase family | at4g01330 | 1.6 | 0.7 | 1.3 | C09:1..40126856:261374-261732 |
| TCONS_00034800 | timing of cab 1 | at5g61380 | 1.6 | -0.4 | 1.9 | C09:1..40126856:3881007-3882252 |
| TCONS_00036122 | Plasma membrane H+-ATPase | at5g57350 | 1.6 | 0.5 | 0.2 | C09:1..40126856:25129867-25131617 |
| TCONS_00009167 | NADH dehydrogenase-like complex N | at5g58260 | 1.6 | 0.2 | 1.7 | C03:1..57781463:5672033-5672811 |
| TCONS_00014538 | -NA- | -- | 1.6 | 0.0 | 0.2 | C04:1..40895475:5206698-5206830 |
| TCONS_00030739 | At1g10180 F14N23 6 | at1g10180 | 1.6 | 0.7 | 1.3 | C08:1..41516064:17778548-17779618 |
| TCONS_00015354 | phosphomethylpyrimidine synthase | at2g29630 | 1.6 | 0.0 | 1.7 | C04:1..40895475:16988851-16990866 |
| TCONS_00014616 | non-green plastid inner envelope membrane | at2g38550 | 1.6 | 0.8 | 0.9 | C04:1..40895475:6291747-6292185 |
| TCONS_00035887 | -NA- | -- | 1.6 | 0.4 | 0.8 | C09:1..40126856:21587064-21587992 |
| TCONS_00005460 | chloroplast import apparatus 2 | at5g57180 | 1.6 | 0.2 | 1.0 | C02:1..44046003:9574643-9576521 |
| TCONS_00036733 | myb-related 1 | at5g18240 | 1.6 | 0.2 | 0.2 | C09:1..40126856:31639749-31641207 |
| TCONS_00009540 | AF361619 1 At2g35260 | at2g35260 | 1.6 | 0.2 | 1.1 | C03:1..57781463:8606549-8607423 |
| TCONS_00012224 | phototropin 1 | at3g45780 | 1.6 | 0.5 | 1.0 | C03:1..57781463:41340098-41342027 |
| TCONS_00034933 | -NA- | at5g67370 | 1.6 | 0.6 | 1.5 | C09:1..40126856:4832865-4834992 |
| TCONS_00021868 | vacuolar-type H+-ATPase subunit B3 (VHA-B3) | at1g20260 | 1.5 | 0.4 | 1.5 | C06:1..40704471:1952940-1953642 |
| TCONS_00036445 | -NA- | at5g22090 | 1.5 | 0.1 | 0.3 | C09:1..40126856:29292189-29294436 |
| TCONS_00030179 | lipid transfer | at1g48750 | 1.5 | 0.7 | 0.3 | C08:1..41516064:8864276-8865366 |
| TCONS_00030036 | EPIDERMAL PATTERNING FACTOR 9-like | at4g12970 | 1.5 | 0.5 | 1.3 | C08:1..41516064:4575789-4576763 |
| TCONS_00000620 | polyol transporter 6 | at4g36670 | 1.5 | 0.6 | 1.3 | C01:1..38761720:1201660-1204665 |
| TCONS_00001395 | nac domain-containing protein 21 22-like | at4g28530 | 1.5 | 0.5 | -0.8 | C01:1..38761720:5770605-5771540 |
| TCONS_00022963 | Potassium sodium hyperpolarization-activated cyclic nucleotide-gated channel 1 | at1g51100 | 1.5 | 0.4 | 1.0 | C06:1..40704471:13074080-13075163 |
| TCONS_00018054 | protochlorophyllide reductase B | at1g03630 | 1.5 | 0.1 | 1.2 | C05:1..32828328:902024-902882 |
| TCONS_00001984 | magnesium-protoporphyrin IX methyltransferase | at4g25080 | 1.5 | 0.2 | 1.2 | C01:1..38761720:11330065-11330701 |
| TCONS_00015353 | phosphomethylpyrimidine synthase | at2g29630 | 1.5 | -0.3 | 1.6 | C04:1..40895475:16987661-16988701 |
| TCONS_00020592 | AT3g14310 MLN21 9 | at3g14310 | 1.5 | 0.8 | 1.6 | C05:1..32828328:28801232-28804908 |
| TCONS_00010076 | -NA- | -- | 1.5 | 0.6 | 0.8 | C03:1..57781463:12986323-12986928 |
| TCONS_00033473 | indole-3-acetic acid inducible 14 | at3g23050 | 1.5 | 0.4 | 0.8 | C08:1..41516064:39621984-39623445 |
| TCONS_00012707 | -NA- | -- | 1.5 | 0.5 | 1.3 | C03:1..57781463:47596754-47597484 |
| TCONS_00017930 | AP2 ERF domain-containing transcription factor | at1g01250 | 1.5 | 0.2 | 0.6 | C05:1..32828328:262696-263647 |
| TCONS_00017188 | transferase family | at2g39980 | 1.5 | 0.2 | 1.3 | C04:1..40895475:38382518-38384118 |
| TCONS_00024350 | GDSL esterase lipase EXL1 | at1g75880 | 1.5 | 0.0 | 0.3 | C06:1..40704471:31999763-32000682 |
| TCONS_00036120 | AF428289 1 AT5g57340 MJB24 15 | at5g57340 | 1.5 | 0.8 | 1.0 | C09:1..40126856:25112506-25116271 |
| TCONS_00022309 | EST gb | at1g70100 | 1.5 | 0.3 | 1.1 | C06:1..40704471:5110397-5111573 |
| TCONS_00019830 | 2-oxoglutarate (2OG) and Fe(II)-dependent oxygenase superfamily | at3g20810 | 1.5 | -0.8 | 1.7 | C05:1..32828328:20989927-20991494 |
| TCONS_00019829 | leucine-rich repeat receptor | at3g20820 | 1.5 | 0.6 | 1.5 | C05:1..32828328:20985137-20986551 |
| TCONS_00033405 | At1g10900 | at1g10900 | 1.5 | 0.6 | 0.4 | C08:1..41516064:39169587-39170360 |
| TCONS_00030842 | S-adenosyl-L-methionine-dependent methyltransferase domain-containing | at5g44590 | 1.5 | 0.1 | 1.4 | C08:1..41516064:18940232-18940928 |
| TCONS_00013609 | photosystem II reaction center W | at2g30570 | 1.5 | 0.4 | 0.8 | C04:1..40895475:17842118-17880765 |
| TCONS_00013332 | -NA- | -- | 1.5 | 0.2 | 0.9 | C03:1..57781463:56216720-56218459 |
| TCONS_00001223 | pentatricopeptide repeat-containing protein | at4g30825 | 1.5 | 0.5 | 1.2 | C01:1..38761720:4164382-4167098 |
| TCONS_00029289 | fructose-bisphosphate aldolase 2 | at4g38970 | 1.5 | 0.3 | 1.2 | C07:1..48346208:48240034-48240330 |
| TCONS_00024743 | -NA- | -- | 1.5 | -0.3 | 1.0 | C06:1..40704471:38538448-38540015 |
| TCONS_00014325 | peroxisomal biogenesis factor 11 family | at2g45740 | 1.5 | 0.2 | 1.8 | C04:1..40895475:3040451-3041053 |
| TCONS_00002036 | Pheophorbide a oxygenase | at4g25650 | 1.5 | 0.4 | 0.8 | C01:1..38761720:11815613-11816041 |
| TCONS_00014817 | zinc finger CCCH domain-containing 46 | at3g51950 | 1.5 | 0.6 | 1.5 | C04:1..40895475:8446766-8449689 |
| TCONS_00013578 | -NA- | at1g67870 | 1.4 | 0.4 | 0.0 | C04:1..40895475:10328168-10333551 |
| TCONS_00005487 | kinase family | at5g56890 | 1.4 | 0.5 | 1.5 | C02:1..44046003:9982895-9986309 |
| TCONS_00037894 | -NA- | at5g03420 | 1.4 | 0.3 | 1.2 | C09:1..40126856:39699673-39700567 |
| TCONS_00008134 | kinesin KIF22-like | at5g02370 | 1.4 | 0.5 | 0.1 | C03:1..57781463:97207-101580 |
| TCONS_00026436 | SOB five-like 1 | at1g26210 | 1.4 | 1.2 | 0.8 | C07:1..48346208:21998752-22001557 |
| TCONS_00037396 | flowering promoting factor 1 | at5g10625 | 1.4 | 0.3 | -0.5 | C09:1..40126856:36842173-36843030 |
| TCONS_00028226 | indole-3-acetic acid inducible 13 | at2g33310 | 1.4 | 0.1 | 0.6 | C07:1..48346208:40415152-40417761 |
| TCONS_00015111 | -NA- | -- | 1.4 | 0.7 | 1.0 | C04:1..40895475:13580163-13580487 |
| TCONS_00034143 | H(+)-ATPase 3 | at5g57350 | 1.4 | -0.2 | -0.1 | C09:1..40126856:25132667-25136804 |
| TCONS_00030273 | Rapid alkalinization factor (RALF) family | at4g14020 | 1.4 | 0.3 | -0.1 | C08:1..41516064:10776818-10777672 |
| TCONS_00016754 | beta glucosidase 33 | at2g32860 | 1.4 | 0.4 | 1.6 | C04:1..40895475:34877958-34887437 |
| TCONS_00011954 | xyloglucan endo-transglycosylase | -- | 1.4 | 0.3 | 0.8 | C03:1..57781463:34127011-34127602 |
| TCONS_00023025 | dienelactone hydrolase | at1g35420 | 1.4 | 0.5 | 0.9 | C06:1..40704471:13903923-13905238 |
| TCONS_00037464 | chaperone -domain containing | at5g09540 | 1.4 | 0.4 | 1.3 | C09:1..40126856:37532558-37533763 |
| TCONS_00034585 | 6,7-dimethyl-8-ribityllumazine synthase | at3g32930 | 1.4 | 0.6 | 1.1 | C09:1..40126856:2507909-2509605 |
| TCONS_00005152 | auxin response factor 2 | -- | 1.4 | 0.2 | 0.8 | C02:1..44046003:5643373-5644679 |
| TCONS_00034870 | probable amino acid permease 7-like | at5g23810 | 1.4 | 0.7 | 0.6 | C09:1..40126856:4266475-4267189 |
| TCONS_00023585 | legume lectin | at1g53070 | 1.4 | 0.7 | 0.6 | C06:1..40704471:21634581-21635630 |
| TCONS_00022262 | -NA- | -- | 1.4 | -0.1 | 1.1 | C06:1..40704471:4567278-4568214 |
| TCONS_00013147 | -NA- | -- | 1.4 | 0.3 | 1.0 | C03:1..57781463:54011315-54011706 |
| TCONS_00037849 | signal recognition particle 54 kDa chloroplastic-like | at5g03940 | 1.4 | 0.1 | 1.1 | C09:1..40126856:39484287-39485633 |
| TCONS_00012296 | -NA- | -- | 1.4 | 0.2 | 1.0 | C03:1..57781463:43062431-43062892 |
| TCONS_00034297 | AT4g01050 F2N1 31 | at4g01050 | 1.4 | 0.3 | 1.0 | C09:1..40126856:375339-376120 |
| TCONS_00014967 | chloroplast sensor kinase | at1g67840 | 1.4 | 0.6 | 1.2 | C04:1..40895475:10302908-10304233 |
| TCONS_00006714 | abc transporter g family member 7 | at2g01320 | 1.4 | -0.2 | 1.3 | C02:1..44046003:34164202-34164674 |
| TCONS_00016856 | urease accessory G | at2g34470 | 1.4 | 0.2 | 0.7 | C04:1..40895475:35726844-35727364 |
| TCONS_00037246 | Tubulin beta chain | at5g12250 | 1.4 | 0.4 | 1.3 | C09:1..40126856:35982362-35984392 |
| TCONS_00021606 | photosystem II subunit R | p49108|psbr_bracm | 1.4 | 0.2 | 1.0 | C06:1..40704471:454506-455846 |
| TCONS_00022513 | -NA- | at1g69523 | 1.4 | 0.6 | 1.1 | C06:1..40704471:7418002-7419799 |
| TCONS_00020750 | FKBP-type peptidyl-prolyl cis-trans | at3g12345 | 1.4 | 0.1 | 1.4 | C05:1..32828328:30208642-30209440 |
| TCONS_00024351 | GDSL esterase lipase EXL1 | at1g75880 | 1.4 | -0.1 | 0.2 | C06:1..40704471:32001791-32002831 |
| TCONS_00034452 | cytochrome b6-f complex iron-sulfur subunit | at4g03280 | 1.4 | 0.4 | 1.1 | C09:1..40126856:1504398-1505418 |
| TCONS_00031881 | chloroplast sedoheptulose-1,7-bisphosphatase | at3g55800 | 1.4 | 0.4 | 1.3 | C08:1..41516064:29863935-29865714 |
| TCONS_00029270 | brassinosteroid insensitive 1 | at4g39400 | 1.4 | 0.2 | 1.0 | C07:1..48346208:48127673-48131795 |
| TCONS_00032575 | glutathione peroxidase partial | at4g31870 | 1.4 | 0.8 | 0.7 | C08:1..41516064:34068834-34070690 |
| TCONS_00003785 | adenosine kinase | at5g03300 | 1.4 | 0.6 | 1.3 | C01:1..38761720:37426483-37426740 |
| TCONS_00022422 | probable peptide nitrate transporter | at3g43790 | 1.4 | 0.5 | 0.6 | C06:1..40704471:6423977-6424835 |
| TCONS_00037790 | Heavy metal transport detoxification superfamily | at5g05365 | 1.4 | -0.0 | 0.1 | C09:1..40126856:39130695-39130927 |
| TCONS_00009993 | ribose-phosphate pyrophosphokinase 4 | at2g42910 | 1.4 | 0.6 | 1.3 | C03:1..57781463:12329913-12331090 |
| TCONS_00024308 | -NA- | -- | 1.4 | 0.3 | 0.5 | C06:1..40704471:31619251-31619807 |
| TCONS_00015110 | indole-3-acetic acid inducible 14 | at3g23050 | 1.4 | 0.8 | 0.8 | C04:1..40895475:13579048-13579686 |
| TCONS_00003725 | alpha-L-arabinofuranosidase | at3g10740 | 1.4 | -0.1 | 0.5 | C01:1..38761720:36944065-36944548 |
| TCONS_00002951 | early light-induced protein | at3g22840 | 1.4 | 0.5 | -0.1 | C01:1..38761720:27637916-27639468 |
| TCONS_00006302 | ATP synthase gamma chloroplastic-like | at4g04640 | 1.4 | 0.3 | 1.1 | C02:1..44046003:23328554-23330257 |
| TCONS_00015490 | dienelactone hydrolase domain-containing | at2g32520 | 1.4 | 0.6 | 1.3 | C04:1..40895475:19128972-19129964 |
| TCONS_00034284 | -NA- | -- | 1.4 | 0.4 | 1.0 | C09:1..40126856:312763-313659 |
| TCONS_00035281 | sodium-dicarboxylate cotransporter-like | at5g47560 | 1.4 | 0.5 | 0.7 | C09:1..40126856:9141863-9143406 |
| TCONS_00030001 | protochlorophyllide reductase C | at1g03630 | 1.4 | -0.2 | 1.3 | C08:1..41516064:2984690-2986263 |
| TCONS_00011046 | glycolate oxidase | at3g14415 | 1.4 | 0.4 | 1.2 | C03:1..57781463:21591634-21603240 |
| TCONS_00029856 | AC025290 10 3-oxoacyl-(acyl-carrier- ) synthase from Arabidopsis thaliana gb | at1g06240 | 1.3 | 0.4 | 1.1 | C08:1..41516064:1985115-1986221 |
| TCONS_00016275 | fructose-bisphosphate aldolase 1 | at2g21330 | 1.3 | 0.1 | 1.3 | C04:1..40895475:29400943-29401926 |
| TCONS_00009754 | plasma membrane intrinsic 2E | at2g39010 | 1.3 | 1.1 | 1.2 | C03:1..57781463:10637685-10638782 |
| TCONS_00018050 | thylakoid lumenal chloroplastic-like | at1g03600 | 1.3 | 0.3 | 1.3 | C05:1..32828328:886922-887698 |
| TCONS_00008836 | uncharacterized LOC101218710 | at5g18850 | 1.3 | 0.4 | 1.1 | C03:1..57781463:3913231-3913941 |
| TCONS_00035764 | probable membrane-associated kinase regulator 6-like | at5g52900 | 1.3 | 0.4 | 1.1 | C09:1..40126856:19600914-19602299 |
| TCONS_00010491 | legume lectin | at3g16530 | 1.3 | 0.4 | 0.0 | C03:1..57781463:17059795-17062622 |
| TCONS_00023662 | FMN binding | at1g51560 | 1.3 | 0.4 | 1.1 | C06:1..40704471:22752802-22753691 |
| TCONS_00014119 | Signal transducer and transcription activator isoform 1 | at2g43340 | 1.3 | 0.5 | 1.0 | C04:1..40895475:1482758-1483877 |
| TCONS_00019803 | -NA- | -- | 1.3 | 0.6 | 0.6 | C05:1..32828328:20346768-20348971 |
| TCONS_00033323 | ATP-dependent Clp protease proteolytic subunit-related chloroplastic-like | at1g12410 | 1.3 | -0.0 | 0.3 | C08:1..41516064:38647340-38647476 |
| TCONS_00011465 | -NA- | -- | 1.3 | 0.9 | 0.8 | C03:1..57781463:27137294-27138603 |
| TCONS_00015108 | indole-3-acetic acid inducible 2 | at4g14560 | 1.3 | 0.7 | 0.4 | C04:1..40895475:13507198-13508608 |
| TCONS_00005542 | trigger factor-like protein tig-like | at5g55220 | 1.3 | 0.0 | 1.4 | C02:1..44046003:10499000-10500724 |
| TCONS_00006482 | -NA- | -- | 1.3 | 0.0 | 0.7 | C02:1..44046003:29703159-29703562 |
| TCONS_00009111 | nonspecific lipid-transfer precursor | -- | 1.3 | 0.5 | 0.3 | C03:1..57781463:5402643-5403166 |
| TCONS_00015104 | -NA- | -- | 1.3 | 0.2 | 0.5 | C04:1..40895475:13430758-13431604 |
| TCONS_00036493 | aldehyde oxidase | at5g20960 | 1.3 | 0.3 | 1.0 | C09:1..40126856:29674621-29676610 |
| TCONS_00034286 | CUT1A ARATH ame: Full= CURVATURE THYLAKOID chloroplastic Flags: Precursor | -- | 1.3 | 0.1 | 0.4 | C09:1..40126856:314330-314613 |
| TCONS_00014556 | protease inhibitor seed storage lipid transfer family | at2g37870 | 1.3 | -0.1 | 0.7 | C04:1..40895475:5353922-5354806 |
| TCONS_00013320 | DCC family chloroplastic-like | at1g52590 | 1.3 | 0.0 | 0.6 | C03:1..57781463:55934960-55935164 |
| TCONS_00025054 | phytoalexin deficient 4 | at3g52430 | 1.3 | 0.3 | 1.2 | C07:1..48346208:38030042-38034499 |
| TCONS_00001985 | magnesium-protoporphyrin ix methyltransferase | at4g25080 | 1.3 | 0.4 | 1.2 | C01:1..38761720:11330800-11331061 |
| TCONS_00005582 | thiazole biosynthetic enzyme | at5g54770 | 1.3 | -0.0 | 1.2 | C02:1..44046003:10899188-10899617 |
| TCONS_00013116 | TPX2 (targeting for Xklp2) family | at4g32330 | 1.3 | 0.5 | 1.4 | C03:1..57781463:53737763-53738790 |
| TCONS_00006504 | NUCLEAR FUSION DEFECTIVE 4-like [Brassica napus] | at5g45275 | 1.3 | 0.6 | 1.3 | C02:1..44046003:30565496-30567233 |
| TCONS_00032190 | GENOMES UNCOUPLED 4 | at3g59400 | 1.3 | 0.2 | 1.3 | C08:1..41516064:31708062-31709209 |
| TCONS_00012723 | PHLOEM 2-LIKE A1 | at4g19840 | 1.3 | 0.2 | 0.0 | C03:1..57781463:48133935-48135669 |
| TCONS_00006695 | -NA- | -- | 1.3 | 0.5 | 1.6 | C02:1..44046003:33938089-33939819 |
| TCONS_00018532 | -NA- | at1g12320 | 1.3 | 0.2 | 0.9 | C05:1..32828328:3601523-3602612 |
| TCONS_00037909 | hypothetical protein EUTSA v10014814mg | at5g03230 | 1.3 | 0.4 | 0.6 | C09:1..40126856:39792489-39793588 |
| TCONS_00034930 | AT5g25610 T14C9 150 | -- | 1.3 | 1.4 | 0.2 | C09:1..40126856:4760089-4760671 |
| TCONS_00034451 | cytochrome b6-f complex iron-sulfur subunit | at4g03280 | 1.3 | 0.3 | 0.9 | C09:1..40126856:1503540-1504238 |
| TCONS_00014974 | phototropic-responsive NPH3 family | at1g67900 | 1.3 | 0.6 | 1.2 | C04:1..40895475:10432124-10434297 |
| TCONS_00026344 | -NA- | -- | 1.3 | 0.3 | 0.9 | C07:1..48346208:19037400-19038758 |
| TCONS_00029216 | sigma factor regulation -like | at4g37470 | 1.3 | 0.2 | 1.0 | C07:1..48346208:47671803-47673399 |
| TCONS_00031735 | fructose-bisphosphatase precursor | q07204|f16p1_brana | 1.3 | 0.1 | 0.5 | C08:1..41516064:28777742-28778360 |
| TCONS_00014493 | thioredoxin chloroplastic-like | at2g37240 | 1.3 | 0.2 | 0.6 | C04:1..40895475:4802988-4803763 |
| TCONS_00024408 | AC008263 13ESTs gb | at1g74940 | 1.3 | 0.2 | 0.8 | C06:1..40704471:32547845-32549126 |
| TCONS_00002338 | protein tic chloroplastic-like | at3g46780 | 1.3 | 0.1 | 1.1 | C01:1..38761720:15387080-15388514 |
| TCONS_00024755 | abscisic acid responsive elements-binding factor 2 | at1g45249 | 1.3 | 0.1 | 0.8 | C06:1..40704471:38729116-38731550 |
| TCONS_00016467 | alpha-L-fucosidase 1 | at2g28100 | 1.3 | 0.5 | 0.6 | C04:1..40895475:30984678-30985901 |
| TCONS_00035286 | Nuclear transcription factor Y subunit B-3 | at5g47640 | 1.3 | 0.3 | 1.1 | C09:1..40126856:9165991-9168257 |
| TCONS_00005502 | ER membrane complex subunit 8 9 homolog | at5g55940 | 1.3 | 0.4 | 1.0 | C02:1..44046003:10097398-10098170 |
| TCONS_00015660 | MTERF isoform 2 | at2g34620 | 1.3 | 0.3 | 1.3 | C04:1..40895475:21180907-21182255 |
| TCONS_00018428 | magnesium-chelatase subunit chloroplastic-like | at1g08520 | 1.3 | 0.4 | 1.3 | C05:1..32828328:3102733-3103575 |
| TCONS_00004635 | -NA- | -- | 1.3 | 0.2 | 0.8 | C02:1..44046003:2388769-2390173 |
| TCONS_00032022 | -NA- | at3g57400 | 1.3 | 0.3 | 0.8 | C08:1..41516064:30818849-30820736 |
| TCONS_00018114 | cryptochrome partial | at1g04400 | 1.3 | 0.0 | 1.2 | C05:1..32828328:1186574-1187477 |
| TCONS_00004766 | adenine phosphoribosyltransferase | at5g11160 | 1.3 | 0.5 | 1.5 | C02:1..44046003:3012176-3014072 |
| TCONS_00004181 | plastid ribosomal S21 | at3g27160 | 1.3 | 0.5 | 1.6 | C02:1..44046003:38532066-38544129 |
| TCONS_00013461 | chaperonin 60 subunit beta 1 | p21241|rubb_brana | 1.3 | -0.0 | 1.0 | C03:1..57781463:57613971-57615712 |
| TCONS_00017903 | plasma membrane intrinsic 1 4 | at1g01620 | 1.2 | 0.2 | 1.4 | C05:1..32828328:59335-59989 |
| TCONS_00006539 | magnesium-chelatase subunit chloroplastic-like | at5g45930 | 1.2 | 0.2 | 1.4 | C02:1..44046003:31291383-31293232 |
| TCONS_00006713 | abc transporter g family member 7 | at2g01320 | 1.2 | 0.1 | 1.4 | C02:1..44046003:34162809-34164037 |
| TCONS_00031914 | biotin lipoyl attachment domain-containing | at3g56130 | 1.2 | 0.5 | 1.2 | C08:1..41516064:30072237-30072786 |
| TCONS_00023685 | P-loop containing nucleoside triphosphate hydrolases superfamily | at1g33290 | 1.2 | 0.1 | 1.2 | C06:1..40704471:23527496-23529582 |
| TCONS_00001396 | protein cup-shaped cotyledon 3-like | at4g28530 | 1.2 | 0.3 | -0.0 | C01:1..38761720:5772690-5774698 |
| TCONS_00010324 | UDP-arabinose mutase | at5g50750 | 1.2 | 0.1 | 0.3 | C03:1..57781463:15148546-15150181 |
| TCONS_00027134 | -NA- | at2g03350 | 1.2 | 0.2 | 1.1 | C07:1..48346208:30898678-30899460 |
| TCONS_00034923 | serine-rich | at5g25280 | 1.2 | 0.6 | 1.1 | C09:1..40126856:4674712-4677092 |
| TCONS_00031736 | fructose-bisphosphatase precursor | q07204|f16p1_brana | 1.2 | 0.3 | 0.9 | C08:1..41516064:28778758-28780302 |
| TCONS_00004754 | cbs domain protein | at5g10860 | 1.2 | 0.5 | 1.1 | C02:1..44046003:2925079-2926533 |
| TCONS_00025682 | RNAse E G | at2g04270 | 1.2 | 0.2 | 0.9 | C07:1..48346208:5930578-5932166 |
| TCONS_00011434 | -NA- | -- | 1.2 | 0.6 | 1.1 | C03:1..57781463:26873059-26874059 |
| TCONS_00016768 | hypothetical protein EUTSA v10017331mg | at2g33180 | 1.2 | 0.3 | 0.9 | C04:1..40895475:35101638-35102715 |
| TCONS_00017059 | protease inhibitor seed storage lipid transfer family | at2g37870 | 1.2 | 0.0 | 1.0 | C04:1..40895475:37495312-37495977 |
| TCONS_00021823 | Dolichyl-diphosphooligosaccharide- glycosyltransferase subunit | at1g76400 | 1.2 | 0.5 | 0.9 | C06:1..40704471:1708281-1709383 |
| TCONS_00016510 | chloroplast-targeted copper chaperone | at2g28660 | 1.2 | 0.4 | 0.9 | C04:1..40895475:31829099-31831119 |
| TCONS_00019215 | hypothetical protein EUTSA v10008795mg | at1g32690 | 1.2 | 0.4 | 0.8 | C05:1..32828328:12739214-12740186 |
| TCONS_00023433 | Nuclear transcription factor Y subunit C-2 isoform 1 | at1g56170 | 1.2 | 0.1 | 1.3 | C06:1..40704471:19743119-19744371 |
| TCONS_00023196 | -NA- | -- | 1.2 | 0.2 | 0.7 | C06:1..40704471:16821796-16822258 |
| TCONS_00016845 | -NA- | at2g34310 | 1.2 | 0.2 | 0.7 | C04:1..40895475:35675990-35678018 |
| TCONS_00026239 | ras-related RABA1i | at1g28540 | 1.2 | 0.3 | 0.6 | C07:1..48346208:17030057-17031268 |
| TCONS_00036607 | peptidase M28 family | at5g19740 | 1.2 | 0.4 | 0.8 | C09:1..40126856:30545930-30546800 |
| TCONS_00034269 | 50S ribosomal chloroplastic-like | at4g01310 | 1.2 | 0.1 | 0.9 | C09:1..40126856:266885-268522 |
| TCONS_00017163 | ribulose bisphosphate carboxylase oxygenase activase | at2g39730 | 1.2 | 0.4 | 1.2 | C04:1..40895475:38243700-38246089 |
| TCONS_00010191 | universal stress family | at2g47710 | 1.2 | 0.1 | 1.0 | C03:1..57781463:14003011-14004298 |
| TCONS_00025388 | hypothetical protein EUTSA v10003296mg | at5g43150 | 1.2 | 0.3 | 0.7 | C07:1..48346208:574710-575973 |
| TCONS_00029843 | oxygen-evolving enhancer 2 | p11594|psbp_sinal | 1.2 | 0.4 | 0.9 | C08:1..41516064:1841744-1844955 |
| TCONS_00013295 | photosystem I subunit VI precursor | at1g52240 | 1.2 | 0.3 | 0.7 | C03:1..57781463:55815872-55818212 |
| TCONS_00030127 | signal recognition particle 43 kDa chloroplastic-like | at2g47450 | 1.2 | 0.5 | 1.0 | C08:1..41516064:8010136-8011804 |
| TCONS_00010166 | hypothetical protein CARUB v10023889mg | at2g46940 | 1.2 | 0.3 | 0.0 | C03:1..57781463:13782042-13783205 |
| TCONS_00007062 | cytochrome c oxidase subunit 5c | at2g47380 | 1.2 | 0.5 | 1.2 | C02:1..44046003:39689068-39690633 |
| TCONS_00028445 | -NA- | -- | 1.2 | 0.6 | 0.4 | C07:1..48346208:42253146-42254604 |
| TCONS_00015360 | zinc finger family | at2g29660 | 1.2 | 0.3 | 1.1 | C04:1..40895475:17027227-17029001 |
| TCONS_00005786 | carbamoyl-phosphate synthase small chain-like | at3g27740 | 1.2 | 0.2 | 1.1 | C02:1..44046003:13680049-13681587 |
| TCONS_00004692 | peroxisomal nad-malate dehydrogenase 2 | q9xfw3|mdhg2_brana | 1.2 | 0.1 | 0.8 | C02:1..44046003:2594805-2595696 |
| TCONS_00024224 | myb family transcription factor APL | at1g79430 | 1.2 | 0.4 | 0.2 | C06:1..40704471:30796456-30797370 |
| TCONS_00002425 | -NA- | -- | 1.2 | 0.1 | 1.0 | C01:1..38761720:17613573-17614697 |
| TCONS_00015554 | -NA- | -- | 1.2 | 0.2 | 0.5 | C04:1..40895475:19803813-19804718 |
| TCONS_00012464 | -NA- | at1g29530 | 1.2 | 0.4 | 0.9 | C03:1..57781463:44836769-44837611 |
| TCONS_00021882 | GA-responsive GAST1 | at1g75750 | 1.2 | 0.4 | 0.1 | C06:1..40704471:2083785-2085033 |
| TCONS_00031529 | hypothetical protein EUTSA v10011124mg, partial | at3g50350 | 1.2 | 0.7 | 0.8 | C08:1..41516064:27135156-27135996 |
| TCONS_00005581 | thiamin biosynthetic enzyme | at5g54770 | 1.2 | -0.2 | 1.0 | C02:1..44046003:10897466-10897986 |
| TCONS_00027364 | LOB domain-containing 25-like | at3g27650 | 1.2 | 0.5 | 1.0 | C07:1..48346208:33058548-33059793 |
| TCONS_00029290 | fructose bisphosphate aldolase | at4g38970 | 1.2 | 0.2 | 1.1 | C07:1..48346208:48240739-48242318 |
| TCONS_00020517 | 4 -phosphopantetheinyl transferase superfamily | at3g11470 | 1.2 | 0.4 | 0.0 | C05:1..32828328:28120310-28123473 |
| TCONS_00037619 | IQ-domain 24 | at5g07240 | 1.2 | 0.6 | 0.6 | C09:1..40126856:38131534-38134217 |
| TCONS_00032632 | ribosomal L35 | at2g24090 | 1.2 | 0.4 | 1.2 | C08:1..41516064:34534709-34535897 |
| TCONS_00017904 | aquaporin PIP1-3 | at1g01620 | 1.2 | 0.2 | 1.4 | C05:1..32828328:60086-60719 |
| TCONS_00010722 | calmodulin 2 family | -- | 1.2 | 0.1 | 0.8 | C03:1..57781463:19444161-19445379 |
| TCONS_00011370 | heat stress transcription factor C-1-like | at3g24520 | 1.2 | 0.2 | 0.7 | C03:1..57781463:26090510-26091842 |
| TCONS_00014368 | Rho GTPase activating with PAK-box P21-Rho-binding domain | at2g46710 | 1.1 | 0.6 | 0.7 | C04:1..40895475:3352414-3354311 |
| TCONS_00014049 | plastid-lipid-associated | at2g42130 | 1.1 | 0.2 | 0.2 | C04:1..40895475:873014-874915 |
| TCONS_00019786 | Dof zinc finger | at3g21270 | 1.1 | 0.1 | 0.5 | C05:1..32828328:19804404-19805603 |
| TCONS_00018197 | 50S ribosomal chloroplastic-like | at1g05190 | 1.1 | 0.3 | 1.1 | C05:1..32828328:1562462-1563501 |
| TCONS_00034836 | transcription factor TCP7-like | at5g23280 | 1.1 | 0.3 | 0.6 | C09:1..40126856:4092942-4094727 |
| TCONS_00007349 | -NA- | -- | 1.1 | 0.4 | 0.4 | C02:1..44046003:43294537-43295864 |
| TCONS_00018113 | cryptochrome partial | at1g04400 | 1.1 | 0.1 | 1.2 | C05:1..32828328:1185322-1186233 |
| TCONS_00016251 | -NA- | -- | 1.1 | 0.1 | 0.4 | C04:1..40895475:29231274-29232267 |
| TCONS_00005272 | AF412087 1 AT3g45210 T14D3 150 | at5g60680 | 1.1 | 0.3 | 0.9 | C02:1..44046003:7002785-7003546 |
| TCONS_00006766 | pcp17c2 | -- | 1.1 | 1.0 | 0.8 | C02:1..44046003:34848843-34850108 |
| TCONS_00029186 | chloroplast 1 precursor | p12359|psbo_spiol | 1.1 | 0.2 | 1.0 | C07:1..48346208:47504887-47506442 |
| TCONS_00006737 | -NA- | -- | 1.1 | 0.4 | 0.8 | C02:1..44046003:34403253-34404783 |
| TCONS_00036556 | sucrose phosphate synthase | at5g20280 | 1.1 | 0.1 | 0.3 | C09:1..40126856:30247622-30249585 |
| TCONS_00024066 | chlorophyll a-b binding chloroplastic-like | at3g54890 | 1.1 | 0.1 | 0.8 | C06:1..40704471:29550708-29551669 |
| TCONS_00037628 | amidase family | at5g07330 | 1.1 | 0.1 | 0.6 | C09:1..40126856:38199034-38200652 |
| TCONS_00033592 | 40S ribosomal S15a-1-like | at5g59850 | 1.1 | 0.4 | 1.0 | C08:1..41516064:40159173-40160415 |
| TCONS_00014617 | non-green plastid inner envelope membrane | at2g38550 | 1.1 | 0.2 | 0.6 | C04:1..40895475:6292531-6293189 |
| TCONS_00034395 | chloroplast thylakoid lumen | at4g02530 | 1.1 | 0.0 | 0.8 | C09:1..40126856:1181593-1182132 |
| TCONS_00033322 | ATP-dependent Clp protease proteolytic subunit-related chloroplastic-like | at1g12410 | 1.1 | 0.1 | 0.5 | C08:1..41516064:38646523-38646703 |
| TCONS_00002355 | bundle-sheath defective protein 2 family | at3g47650 | 1.1 | -0.1 | 1.2 | C01:1..38761720:15932008-15933150 |
| TCONS_00026225 | -NA- | -- | 1.1 | 0.1 | 0.9 | C07:1..48346208:16734167-16735857 |
| TCONS_00015555 | -NA- | -- | 1.1 | -0.0 | 0.3 | C04:1..40895475:19808548-19808752 |
| TCONS_00007000 | chlorophyll a b binding protein | at3g27690 | 1.1 | 0.6 | 0.0 | C02:1..44046003:38953194-38954364 |
| TCONS_00007242 | Root phototropism | at5g67385 | 1.1 | 0.3 | 1.0 | C02:1..44046003:42105299-42106044 |
| TCONS_00036492 | aldehyde oxidase | at5g20960 | 1.1 | 0.6 | 1.2 | C09:1..40126856:29673337-29674499 |
| TCONS_00033554 | photosystem I subunit O | at1g08380 | 1.1 | 0.3 | 0.8 | C08:1..41516064:39984626-39985559 |
| TCONS_00035014 | -NA- | -- | 1.1 | 0.5 | 0.6 | C09:1..40126856:5542359-5543583 |
| TCONS_00005098 | spermine synthase | at5g19530 | 1.1 | 0.4 | 1.1 | C02:1..44046003:5351311-5353046 |
| TCONS_00012594 | proline-rich 4 | at4g38770 | 1.1 | 0.8 | -0.5 | C03:1..57781463:45996398-45998215 |
| TCONS_00005501 | calcineurin B 2 | at5g55990 | 1.1 | 0.0 | 0.4 | C02:1..44046003:10093705-10096223 |
| TCONS_00035303 | 30S ribosomal chloroplastic-like | at1g79850 | 1.1 | 0.3 | 1.1 | C09:1..40126856:9316785-9317469 |
| TCONS_00022423 | probable peptide nitrate transporter | at3g43790 | 1.1 | 0.4 | 0.5 | C06:1..40704471:6424959-6427773 |
| TCONS_00019966 | Delta(24)-sterol reductase | at3g19820 | 1.1 | 0.1 | 1.1 | C05:1..32828328:22809838-22811935 |
| TCONS_00035960 | 50S ribosomal L24 | at5g54600 | 1.1 | 0.2 | 1.1 | C09:1..40126856:22807017-22808374 |
| TCONS_00013484 | photosystem I reaction center subunit chloroplastic-like | at1g55670 | 1.1 | 0.5 | 0.6 | C03:1..57781463:57745327-57745496 |
| TCONS_00031247 | hypothetical protein EUTSA v10008900mg | at1g26920 | 1.1 | 0.3 | -0.1 | C08:1..41516064:22518936-22520133 |
| TCONS_00002775 | -NA- | -- | 1.1 | 0.1 | 0.5 | C01:1..38761720:24691917-24693399 |
| TCONS_00020855 | translation elongation factor EF1B ribosomal S6 family | at3g17170 | 1.1 | 0.1 | 0.8 | C05:1..32828328:30930226-30930599 |
| TCONS_00005960 | geranylgeranyl reductase | at1g74470 | 1.1 | 0.2 | 1.0 | C02:1..44046003:17408598-17410638 |
| TCONS_00009802 | ribulose bisphosphate carboxylase oxygenase activase | at2g39730 | 1.1 | -0.3 | 0.9 | C03:1..57781463:10911808-10912597 |
| TCONS_00012641 | S-adenosylmethionine carrier 1 | at4g39460 | 1.1 | 0.5 | 1.0 | C03:1..57781463:46428224-46428850 |
| TCONS_00028320 | NAC domain containing 50 | at1g73740 | 1.1 | 0.2 | 0.6 | C07:1..48346208:41129368-41131201 |
| TCONS_00023472 | photosystem I reaction center subunit chloroplastic-like | at1g55670 | 1.1 | 0.0 | 1.0 | C06:1..40704471:20002536-20003428 |
| TCONS_00037951 | hypothetical protein EUTSA v10014890mg | at5g02640 | 1.1 | 0.3 | 0.1 | C09:1..40126856:40045141-40046112 |
| TCONS_00015312 | beta-galactosidase 8 | at2g28470 | 1.1 | 0.5 | -0.0 | C04:1..40895475:16235954-16238567 |
| TCONS_00031429 | adenosylhomocysteinase | at3g23810 | 1.1 | 0.4 | 0.7 | C08:1..41516064:25289339-25291633 |
| TCONS_00027147 | probable carboxylesterase 7 | at2g03550 | 1.1 | 0.4 | 0.5 | C07:1..48346208:31001546-31003381 |
| TCONS_00024458 | mitotic-spindle organizing 1B-like | at4g09550 | 1.1 | 0.2 | 0.7 | C06:1..40704471:33219836-33220549 |
| TCONS_00012700 | sigma factor sigB regulation rsbQ | at4g37470 | 1.1 | 0.1 | 0.6 | C03:1..57781463:47319204-47320026 |
| TCONS_00028855 | chloroplastic group IIA intron splicing facilitator chloroplastic-like | at4g29750 | 1.1 | 0.0 | 1.0 | C07:1..48346208:45317301-45319917 |
| TCONS_00004545 | chaperone protein dnaj-like protein | at5g06130 | 1.1 | 0.0 | 1.0 | C02:1..44046003:1935986-1936761 |
| TCONS_00033910 | -NA- | at5g54585 | 1.1 | 0.8 | 1.0 | C09:1..40126856:22776482-22783652 |
| TCONS_00011091 | phytochrome B | at2g18790 | 1.1 | 0.2 | 0.8 | C03:1..57781463:22395837-22397812 |
| TCONS_00017366 | glyoxalase 2-1 | at2g43430 | 1.1 | 0.2 | 0.7 | C04:1..40895475:39773308-39774132 |
| TCONS_00037066 | PSI type III chlorophyll a b-binding | at1g61520 | 1.1 | 0.5 | 0.5 | C09:1..40126856:34586652-34588047 |
| TCONS_00002034 | protochlorophyllide-dependent translocon component chloroplastic-like | at4g25650 | 1.1 | 0.2 | 0.9 | C01:1..38761720:11813560-11814532 |
| TCONS_00025657 | -NA- | -- | 1.1 | 0.2 | 0.0 | C07:1..48346208:5094646-5095733 |
| TCONS_00027765 | -NA- | at5g24060 | 1.1 | 0.2 | 0.7 | C07:1..48346208:36429730-36431477 |
| TCONS_00007048 | -NA- | -- | 1.0 | 0.3 | 0.7 | C02:1..44046003:39555339-39556280 |
| TCONS_00008188 | pentatricopeptide repeat-containing | at5g03560 | 1.0 | 0.0 | 0.5 | C03:1..57781463:321886-322348 |
| TCONS_00014491 | chloroplast RNA-binding 29 | at2g37220 | 1.0 | 0.1 | 0.6 | C04:1..40895475:4800347-4801063 |
| TCONS_00034497 | armadillo repeat only 4 | at3g26600 | 1.0 | 0.1 | 0.6 | C09:1..40126856:1854769-1857737 |
| TCONS_00008336 | multiple C2 and transmembrane domain-containing 2-like | at5g06850 | 1.0 | 0.2 | 0.1 | C03:1..57781463:1218772-1221662 |
| TCONS_00020110 | plastid developmental DAG | at1g11440 | 1.0 | 0.7 | 1.0 | C05:1..32828328:25121663-25123996 |
| TCONS_00034404 | MLO 1 | -- | 1.0 | 0.3 | 0.9 | C09:1..40126856:1197428-1198166 |
| TCONS_00037466 | transcription factor transcription regulator | at5g09460 | 1.0 | 0.1 | 1.2 | C09:1..40126856:37539203-37541938 |
| TCONS_00006160 | glutathione transferase | at1g78380 | 1.0 | 0.2 | 0.7 | C02:1..44046003:20696006-20697265 |
| TCONS_00015744 | SWI SNF-related matrix-associated actin-dependent regulator of chromatin subfamily A member 3-like 2-like | -- | 1.0 | 0.1 | 0.5 | C04:1..40895475:21855012-21857012 |
| TCONS_00011454 | chlorophyll a b binding | at3g27690 | 1.0 | 0.6 | 0.5 | C03:1..57781463:26994471-26995551 |
| TCONS_00016855 | urease accessory G | at2g34470 | 1.0 | 0.3 | 0.9 | C04:1..40895475:35725591-35726563 |
| TCONS_00027191 | Hydroxyproline-rich glyco family isoform 1 | at3g25690 | 1.0 | 0.4 | 0.8 | C07:1..48346208:31542678-31545310 |
| TCONS_00017103 | LURP-one-related 8-like | at2g38640 | 1.0 | 0.4 | 0.1 | C04:1..40895475:37884468-37885868 |
| TCONS_00023159 | -NA- | at3g62090 | 1.0 | 0.0 | 0.6 | C06:1..40704471:16462817-16464897 |
| TCONS_00003071 | AT3g20680 F3H11 7 | at3g20680 | 1.0 | 0.4 | 1.1 | C01:1..38761720:29867840-29869105 |
| TCONS_00035972 | thiazole biosynthetic enzyme | at5g54770 | 1.0 | -0.0 | 0.3 | C09:1..40126856:23121320-23122219 |
| TCONS_00035669 | xanthine uracil permease family | at5g49990 | 1.0 | 0.3 | 0.5 | C09:1..40126856:16605749-16606740 |
| TCONS_00022476 | probable peptide nitrate transporter | at1g69850 | 1.0 | 0.1 | 0.7 | C06:1..40704471:7061817-7063121 |
| TCONS_00008904 | probable plastid-lipid-associated chloroplastic-like | at5g19940 | 1.0 | 0.2 | 0.7 | C03:1..57781463:4148910-4150314 |
| TCONS_00033199 | RNA-binding KH domain-containing | at1g14170 | 1.0 | 0.4 | 0.6 | C08:1..41516064:37942751-37944957 |
| TCONS_00005365 | kinase family | at5g58950 | 1.0 | 0.5 | 0.8 | C02:1..44046003:8309220-8311353 |
| TCONS_00004691 | peroxisomal nad-malate dehydrogenase 2 | q9xfw3|mdhg2_brana | 1.0 | 0.0 | 0.9 | C02:1..44046003:2593281-2594362 |
| TCONS_00027983 | phosphoglycerate mutase family | at3g52155 | 1.0 | 0.3 | 1.1 | C07:1..48346208:37976260-37977749 |
| TCONS_00033293 | glyceraldehyde-3-phosphate dehydrogenase (NADP+) (phosphorylating) | at1g12900 | 1.0 | 0.2 | 1.0 | C08:1..41516064:38525919-38528643 |
| TCONS_00010995 | Disease resistance-responsive (dirigent ) family | at3g13650 | 1.0 | 0.1 | 0.6 | C03:1..57781463:21270528-21271429 |
| TCONS_00005422 | apo protein chloroplastic-like | at5g57930 | 1.0 | 0.2 | 1.0 | C02:1..44046003:8844458-8846267 |
| TCONS_00019567 | plastid transcriptionally active 6 | at1g21600 | 1.0 | 0.0 | 0.9 | C05:1..32828328:16946155-16946906 |
| TCONS_00014828 | rubredoxin family | at1g54500 | 1.0 | 0.5 | 1.1 | C04:1..40895475:8596850-8597845 |
| TCONS_00001470 | magnesium-chelatase subunit chloroplastic-like | at4g18480 | 1.0 | 0.2 | 0.8 | C01:1..38761720:6521856-6524158 |
| TCONS_00030469 | -NA- | -- | 1.0 | 0.2 | 0.4 | C08:1..41516064:14476694-14477704 |
| TCONS_00013449 | AT3g13510 MRP15 15 | at1g55360 | 1.0 | 0.3 | 0.8 | C03:1..57781463:57547073-57547757 |
| TCONS_00003108 | Delta(24)-sterol reductase | at3g19820 | 1.0 | 0.3 | 1.1 | C01:1..38761720:30379862-30382771 |
| TCONS_00028313 | magnesium-chelatase subunit chlI | at4g18480 | 1.0 | 0.4 | 0.7 | C07:1..48346208:41038696-41039974 |
| TCONS_00010057 | F-box kelch-repeat | at2g44130 | 1.0 | 1.2 | 0.6 | C03:1..57781463:12888824-12890684 |
| TCONS_00011953 | xyloglucan endo-transglycosylase | at3g44990 | 1.0 | 0.4 | 0.8 | C03:1..57781463:34124974-34126639 |
| TCONS_00034873 | hypothetical protein EUTSA v10004859mg | at5g23920 | 1.0 | 0.4 | 1.3 | C09:1..40126856:4277673-4279065 |
| TCONS_00037385 | argininosuccinate lyase | at5g10920 | 1.0 | 0.3 | 0.8 | C09:1..40126856:36674658-36675669 |
| TCONS_00013485 | photosystem I subunit G | -- | 1.0 | 0.4 | 0.7 | C03:1..57781463:57745664-57746052 |
| TCONS_00005170 | glycosyl hydrolase family 3 | at5g20950 | 1.0 | 0.4 | 1.1 | C02:1..44046003:5805209-5808097 |
| TCONS_00021845 | cytoplasmic tRNA 2-thiolation 1-like | at2g44270 | 1.0 | 0.0 | 0.6 | C06:1..40704471:1855116-1857659 |
| TCONS_00014539 | -NA- | -- | 1.0 | -0.3 | 0.0 | C04:1..40895475:5207083-5207369 |
| TCONS_00009801 | ribulose bisphosphate carboxylase oxygenase activase chloroplastic-like | at2g39730 | 1.0 | 0.3 | 1.0 | C03:1..57781463:10909952-10911192 |
| TCONS_00017472 | peroxisomal biogenesis factor 11 family | at2g45740 | 1.0 | 0.2 | 0.9 | C04:1..40895475:40446146-40447046 |
| TCONS_00024700 | photosystem II subunit S | at1g44575 | 1.0 | 0.3 | 0.9 | C06:1..40704471:37716207-37717902 |
| TCONS_00024651 | serine racemase | at4g11640 | 1.0 | 0.4 | 0.8 | C06:1..40704471:36498725-36500482 |
| TCONS_00027897 | ribulose-phosphate 3-epimerase | q43843|rpe_soltu | 1.0 | 0.1 | 0.8 | C07:1..48346208:37248072-37249522 |
| TCONS_00027840 | Extracellular Ca2+ sensing receptor | at5g23060 | 1.0 | 0.4 | 0.8 | C07:1..48346208:36856079-36858578 |
| TCONS_00031983 | magnesium-protoporphyrin IX monomethyl ester [oxidative] cyclase | at3g56940 | 1.0 | 0.1 | 0.7 | C08:1..41516064:30538553-30539178 |
| TCONS_00034296 | -NA- | at4g01050 | 1.0 | 0.2 | 0.9 | C09:1..40126856:373493-374512 |
| TCONS_00016735 | -like cupins superfamily | at2g32650 | 1.0 | 0.4 | 0.8 | C04:1..40895475:34685424-34686130 |
| TCONS_00006389 | -NA- | -- | 1.0 | 0.2 | 0.7 | C02:1..44046003:26631809-26632949 |
| TCONS_00035595 | chlorophyll a-b binding CP26 | at4g10340 | 1.0 | 0.3 | 0.6 | C09:1..40126856:14087021-14088986 |
| TCONS_00034907 | zinc finger CONSTANS-LIKE 4 | at5g24930 | 0.9 | 0.4 | 0.6 | C09:1..40126856:4607801-4609863 |
| TCONS_00021053 | oxidoreductase -like | at5g67290 | 0.9 | 0.2 | 0.4 | C05:1..32828328:32340060-32341762 |
| TCONS_00029075 | -NA- | at4g33630 | 0.9 | 0.2 | 0.5 | C07:1..48346208:46628579-46631258 |
| TCONS_00016088 | -NA- | -- | 0.9 | 0.3 | 0.1 | C04:1..40895475:27167021-27167976 |
| TCONS_00014490 | chloroplast RNA binding | at2g37220 | 0.9 | 0.1 | 0.7 | C04:1..40895475:4799181-4800127 |
| TCONS_00024928 | triacylglycerol lipase 1 | at2g15230 | 0.9 | 0.2 | 1.0 | C07:1..48346208:15712435-15739307 |
| TCONS_00002323 | tet3 arath ame: full=tetraspanin-3 | at3g45600 | 0.9 | 0.4 | 0.2 | C01:1..38761720:14938858-14939922 |
| TCONS_00018759 | SNF1-related kinase regulatory subunit gamma-like PV42a-like | at1g15330 | 0.9 | 0.1 | 0.6 | C05:1..32828328:5799009-5800504 |
| TCONS_00003442 | plasma membrane intrinsic 3 | at4g35100 | 0.9 | 0.1 | 1.0 | C01:1..38761720:34382152-34384152 |
| TCONS_00001950 | rna-binding protein cp31 | at4g24770 | 0.9 | 0.3 | 0.9 | C01:1..38761720:11127837-11129627 |
| TCONS_00033527 | -NA- | at1g08760 | 0.9 | 0.2 | 0.7 | C08:1..41516064:39888152-39891232 |
| TCONS_00006892 | -NA- | -- | 0.9 | 0.2 | 0.7 | C02:1..44046003:37335846-37337838 |
| TCONS_00024100 | 50S ribosomal chloroplastic-like | at3g54210 | 0.9 | 0.4 | 1.2 | C06:1..40704471:29905031-29906051 |
| TCONS_00004876 | basic leucine-zipper 42 | at5g15830 | 0.9 | 0.7 | 0.8 | C02:1..44046003:3683056-3684132 |
| TCONS_00022086 | alpha dioxygenase | at1g73680 | 0.9 | 0.4 | 0.8 | C06:1..40704471:3562192-3564577 |
| TCONS_00012515 | photosystem I reaction center subunit X psaK | at1g30380 | 0.9 | 0.2 | 0.6 | C03:1..57781463:45567159-45568310 |
| TCONS_00006704 | -NA- | -- | 0.9 | 0.4 | 0.6 | C02:1..44046003:34137512-34138547 |
| TCONS_00005937 | ac016662 23 glucosyltransferase 88035-86003 | at1g73885 | 0.9 | 0.2 | 1.1 | C02:1..44046003:17058562-17059870 |
| TCONS_00006581 | tetratricopeptide repeat domain-containing protein | at5g20190 | 0.9 | 0.5 | 0.8 | C02:1..44046003:32086735-32088691 |
| TCONS_00008921 | Sulfate adenylyltransferase subunit 2 | at5g20130 | 0.9 | 0.6 | 0.6 | C03:1..57781463:4204587-4206111 |
| TCONS_00027008 | phototropic-responsive NPH3 family | at5g47800 | 0.9 | 0.0 | 0.8 | C07:1..48346208:29499178-29503342 |
| TCONS_00028016 | DEF (CLA1) | at4g15560 | 0.9 | 0.1 | 1.1 | C07:1..48346208:38249516-38251589 |
| TCONS_00035478 | ATP synthase gamma chloroplastic-like | at4g04640 | 0.9 | 0.1 | 1.0 | C09:1..40126856:11896583-11898390 |
| TCONS_00005112 | sucrose phosphate synthase | at5g20280 | 0.9 | 0.3 | 0.6 | C02:1..44046003:5429492-5430864 |
| TCONS_00008560 | magnesium chelatase subunit H | at5g13630 | 0.9 | 0.3 | 1.0 | C03:1..57781463:2284083-2289728 |
| TCONS_00019597 | regulatory component of ABA receptor 1 | at1g01360 | 0.9 | 0.3 | 0.3 | C05:1..32828328:17217058-17218519 |
| TCONS_00018353 | tropinone reductase | at1g07440 | 0.9 | 0.2 | 0.2 | C05:1..32828328:2559736-2561422 |
| TCONS_00013786 | PGPS D10 | at1g67910 | 0.9 | 0.2 | 0.5 | C04:1..40895475:10444917-10458716 |
| TCONS_00018660 | RNA recognition motif-containing | at1g14340 | 0.9 | 0.2 | 0.4 | C05:1..32828328:5045839-5046373 |

## ABA+Pyr-down

Table S2.6. Downregulated gene products after 8 days of ABA+Pyr treatment. Adjusted p-value <0.05. Gene name, as annotated by Blast2GO after best blastx in nr Viridiplantae database. Best Swiss-Prot hit (Uniprot manually annotated and reviewed protein database) as *Arabidopsis thaliana* locus name or Uniprot “Entry|Entry name” if best curated hit is from another species. ABA, Pyr and ABA+Pyr log2 fold change to control condition. Locus, *Brassica oleracea* v1.0 genomic locus (Liu et al. 2014).

| Isoform id | Gene name | Best Swiss-Prot hit | ABA | Pyr | ABA+Pyr | Locus |
| --- | --- | --- | --- | --- | --- | --- |
| TCONS_00005109 | -NA- | -- | -Inf | -1.3 | -Inf | C02:1..44046003:5413399-5413793 |
| TCONS_00005623 | serine carboxypeptidase-like 42-like | -- | -Inf | -1.0 | -1.0 | C02:1..44046003:11287143-11287310 |
| TCONS_00006173 | Arabidopsis thaliana chromosome complete sequence | -- | -Inf | -0.4 | -2.9 | C02:1..44046003:21296332-21296909 |
| TCONS_00009383 | Cytochrome P450 | at5g52400 | -Inf | 1.1 | -0.9 | C03:1..57781463:7511998-7512659 |
| TCONS_00009829 | proline transporter 1 | at3g55740 | -Inf | -1.1 | -3.9 | C03:1..57781463:11025697-11025969 |
| TCONS_00009831 | proline transporter 1 | at3g55740 | -Inf | -1.1 | -4.7 | C03:1..57781463:11026725-11026936 |
| TCONS_00013012 | -NA- | -- | -Inf | 0.1 | -3.0 | C03:1..57781463:52546040-52546328 |
| TCONS_00013046 | -NA- | -- | -Inf | -1.7 | -1.9 | C03:1..57781463:53088088-53088720 |
| TCONS_00014085 | NAC domain-containing 42 | at2g43000 | -Inf | 0.9 | -0.4 | C04:1..40895475:1206498-1206774 |
| TCONS_00014561 | -NA- | -- | -Inf | -Inf | -2.8 | C04:1..40895475:5485455-5485877 |
| TCONS_00015798 | -NA- | -- | -Inf | -4.1 | -4.2 | C04:1..40895475:23043638-23044157 |
| TCONS_00018335 | phosphatase 2C | at1g07160 | -Inf | -2.6 | -2.4 | C05:1..32828328:2475199-2475736 |
| TCONS_00021122 | -NA- | -- | -Inf | -1.9 | -1.0 | C05:1..32828328:32765190-32765601 |
| TCONS_00024236 | -NA- | -- | -Inf | -1.2 | -4.4 | C06:1..40704471:30919513-30920883 |
| TCONS_00025771 | -NA- | -- | -Inf | -0.1 | -3.7 | C07:1..48346208:7607045-7607224 |
| TCONS_00026625 | Glutathione S-transferase family | at4g19880 | -Inf | -1.6 | -2.7 | C07:1..48346208:24835983-24836313 |
| TCONS_00028474 | -NA- | -- | -Inf | 0.0 | -1.8 | C07:1..48346208:42569537-42569907 |
| TCONS_00031524 | -NA- | -- | -Inf | -0.4 | -2.5 | C08:1..41516064:27022822-27022936 |
| TCONS_00032255 | beta glucosidase 27 | at3g60120 | -Inf | 0.8 | -0.8 | C08:1..41516064:32068565-32068724 |
| TCONS_00035202 | WRKY transcription factor 8 | -- | -Inf | 1.8 | 0.5 | C09:1..40126856:7993776-7993982 |
| TCONS_00003691 | leucoanthocyanidin dioxygenase | at3g11180 | -4.8 | -0.6 | -2.5 | C01:1..38761720:36668622-36669910 |
| TCONS_00008737 | NAD(P)H dehydrogenase mitochondrial-like | at2g20800 | -4.2 | -0.9 | -2.4 | C03:1..57781463:3248310-3250561 |
| TCONS_00017086 | anthocyanidin synthase | at2g38240 | -3.9 | -0.1 | -2.0 | C04:1..40895475:37685991-37688522 |
| TCONS_00023451 | -NA- | at1g56060 | -3.8 | -1.1 | -1.8 | C06:1..40704471:19830267-19831659 |
| TCONS_00030578 | uncharacterized protein | -- | -3.8 | -0.1 | -2.1 | C08:1..41516064:15930104-15930747 |
| TCONS_00030925 | ATPDR7 PDR7 | at1g15210 | -3.7 | -1.2 | -2.2 | C08:1..41516064:19527996-19529428 |
| TCONS_00005654 | -NA- | -- | -3.7 | -0.3 | -2.9 | C02:1..44046003:11719515-11720109 |
| TCONS_00009283 | F-box family | at5g55150 | -3.6 | -0.9 | -1.9 | C03:1..57781463:6443157-6444442 |
| TCONS_00020926 | -NA- | -- | -3.6 | -1.1 | -1.0 | C05:1..32828328:31673703-31674197 |
| TCONS_00032718 | -NA- | -- | -3.5 | -0.5 | -2.0 | C08:1..41516064:35292676-35294153 |
| TCONS_00035433 | myb-related M4 | at4g05100 | -3.5 | -0.5 | -1.7 | C09:1..40126856:11505040-11506111 |
| TCONS_00014215 | glycosyl hydrolase family 1 | at3g60120 | -3.5 | 0.8 | -0.9 | C04:1..40895475:2165006-2165908 |
| TCONS_00024296 | TOM1 2-like | at1g76970 | -3.5 | -0.7 | -2.5 | C06:1..40704471:31464226-31466581 |
| TCONS_00000989 | calmodulin-binding protein | at4g33050 | -3.4 | 0.3 | -1.0 | C01:1..38761720:2893586-2895020 |
| TCONS_00025770 | 1-aminocyclopropane-1-carboxylate oxidase 1-like | at2g19590 | -3.4 | -0.2 | -1.8 | C07:1..48346208:7605748-7606927 |
| TCONS_00037465 | -NA- | at5g09530 | -3.4 | -1.2 | -0.7 | C09:1..40126856:37534330-37535438 |
| TCONS_00033897 | -NA- | at5g52750 | -3.4 | -0.4 | -2.0 | C09:1..40126856:19179722-19192700 |
| TCONS_00026047 | S-adenosyl-L-methionine-dependent methyltransferases superfamily | at3g44840 | -3.3 | 0.4 | -1.5 | C07:1..48346208:13675479-13676895 |
| TCONS_00000827 | ethylene-responsive transcription factor erf109-like | at4g34410 | -3.3 | -0.6 | -1.6 | C01:1..38761720:2326030-2327105 |
| TCONS_00033664 | glutaredoxin ATGRXS13 | at1g03850 | -3.3 | -0.8 | -1.5 | C08:1..41516064:40751704-40752547 |
| TCONS_00020925 | dehydration-responsive element-binding 2A | at3g11020 | -3.3 | -0.6 | -1.2 | C05:1..32828328:31672872-31673618 |
| TCONS_00019145 | TIFY 5A | at1g30135 | -3.3 | -0.5 | -0.9 | C05:1..32828328:11549997-11550923 |
| TCONS_00010154 | jasmonic acid-amido synthetase JAR1 | at2g46370 | -3.2 | -0.5 | -4.2 | C03:1..57781463:13630980-13632079 |
| TCONS_00032332 | -NA- | at3g61390 | -3.2 | -0.6 | -0.8 | C08:1..41516064:32648527-32650667 |
| TCONS_00032678 | VQ motif-containing | at2g22880 | -3.2 | -0.6 | -2.1 | C08:1..41516064:34949250-34950003 |
| TCONS_00019654 | metallo ase | at1g24140 | -3.2 | 0.0 | -1.6 | C05:1..32828328:17894433-17895860 |
| TCONS_00005446 | RNA-binding with serine-rich domain 1-like | at5g57510 | -3.2 | -0.5 | -1.6 | C02:1..44046003:9259465-9260621 |
| TCONS_00013810 | -NA- | at2g30670 | -3.2 | 0.6 | -2.0 | C04:1..40895475:16592016-16675941 |
| TCONS_00023358 | ase inhibitor serpin | at1g55230 | -3.2 | -0.7 | -1.7 | C06:1..40704471:18661941-18663113 |
| TCONS_00000555 | indole-3-acetic acid-amido synthetase | at4g37390 | -3.2 | -0.3 | -0.6 | C01:1..38761720:854882-857391 |
| TCONS_00007931 | -NA- | at1g01680 | -3.2 | -1.1 | -1.9 | C03:1..57781463:13544901-13549304 |
| TCONS_00035098 | nodulin family | at2g16660 | -3.2 | -1.0 | -1.5 | C09:1..40126856:6340077-6341360 |
| TCONS_00033898 | -NA- | at5g52750 | -3.1 | -0.6 | -1.9 | C09:1..40126856:19179722-19192700 |
| TCONS_00008279 | leucoanthocyanidin dioxygenase | at5g05600 | -3.1 | -1.2 | -2.6 | C03:1..57781463:899339-902214 |
| TCONS_00025359 | basic helix-loop-helix family | at5g43650 | -3.1 | -1.1 | -1.4 | C07:1..48346208:43104-43503 |
| TCONS_00010565 | copper transport | at4g05030 | -3.1 | -0.6 | -1.5 | C03:1..57781463:17815308-17816011 |
| TCONS_00037086 | GHMP kinase family | at5g14470 | -3.1 | -0.5 | 0.3 | C09:1..40126856:34974586-34975626 |
| TCONS_00014017 | Hop-interacting THI031 | at2g41730 | -3.1 | -1.1 | -2.3 | C04:1..40895475:627672-628367 |
| TCONS_00010155 | jasmonic acid-amido synthetase JAR1 | at2g46370 | -3.1 | -0.4 | -5.0 | C03:1..57781463:13632243-13634255 |
| TCONS_00037087 | GHMP kinase family | at5g14470 | -3.1 | -0.9 | -0.2 | C09:1..40126856:34975817-34976667 |
| TCONS_00014480 | phenylalanine ammonia-lyase | at2g37040 | -3.1 | -0.5 | -3.2 | C04:1..40895475:4708274-4710312 |
| TCONS_00000709 | -NA- | -- | -3.1 | -0.6 | -1.6 | C01:1..38761720:1789888-1790602 |
| TCONS_00014510 | zinc finger | at2g37430 | -3.1 | 0.0 | -0.9 | C04:1..40895475:4900177-4900916 |
| TCONS_00014613 | WRKY transcription factor 33 | at2g38470 | -3.1 | -0.3 | -1.2 | C04:1..40895475:6218712-6221350 |
| TCONS_00027759 | probable WRKY transcription factor 41-like | at5g24110 | -3.1 | -0.4 | -3.2 | C07:1..48346208:36393305-36395049 |
| TCONS_00019355 | CDP-diacylglycerol--glycerol-3-phosphate 3- partial | at1g19020 | -3.1 | -0.0 | -2.2 | C05:1..32828328:14593080-14593710 |
| TCONS_00016931 | yellow-leaf-specific gene 9 | at2g35980 | -3.1 | 0.5 | -0.7 | C04:1..40895475:36554083-36555013 |
| TCONS_00034803 | hypothetical protein EUTSA v10005448mg | at5g61340 | -3.1 | -2.8 | -1.7 | C09:1..40126856:3899487-3900662 |
| TCONS_00020934 | -NA- | -- | -3.1 | -1.2 | -2.8 | C05:1..32828328:31762375-31763017 |
| TCONS_00005545 | o-glycosyl hydrolases family 17 protein | at5g55180 | -3.1 | -0.5 | -1.9 | C02:1..44046003:10519385-10521094 |
| TCONS_00022241 | -NA- | -- | -3.1 | -0.8 | -1.7 | C06:1..40704471:4416685-4417787 |
| TCONS_00001234 | f-box family protein | at4g30640 | -3.0 | -0.7 | -1.8 | C01:1..38761720:4272206-4273630 |
| TCONS_00033004 | lipoxygenase 3 | at1g17420 | -3.0 | -1.6 | -2.5 | C08:1..41516064:36874625-36876372 |
| TCONS_00003712 | AC009991 24hypothetical protein | -- | -3.0 | -0.4 | -1.8 | C01:1..38761720:36870019-36870601 |
| TCONS_00004683 | hydroxyproline-rich glycoprotein family protein | at5g09530 | -3.0 | -0.6 | -1.0 | C02:1..44046003:2571582-2573287 |
| TCONS_00012488 | gibberellin 2-beta-dioxygenase 2 | at1g30040 | -3.0 | -0.4 | -2.3 | C03:1..57781463:45127521-45130082 |
| TCONS_00001707 | wrky transcription factor 6-like | at4g22070 | -3.0 | -0.5 | -1.8 | C01:1..38761720:8522442-8524297 |
| TCONS_00009737 | -NA- | at2g38790 | -3.0 | -0.9 | -2.4 | C03:1..57781463:10492454-10493659 |
| TCONS_00001588 | calcium binding protein | at4g20780 | -3.0 | -0.3 | -1.7 | C01:1..38761720:7435609-7436990 |
| TCONS_00027577 | hypothetical protein CARUB v10027111mg | at5g48657 | -2.9 | -0.1 | -1.3 | C07:1..48346208:34876391-34878623 |
| TCONS_00001755 | auxin-responsive family | at4g22620 | -2.9 | 0.3 | -0.0 | C01:1..38761720:8948929-8950550 |
| TCONS_00033871 | allene oxide cyclase | at3g25780 | -2.9 | -0.7 | -1.9 | C09:1..40126856:10969271-10980417 |
| TCONS_00001643 | serine threonine kinase | at4g21390 | -2.9 | -0.6 | -2.5 | C01:1..38761720:7887535-7888146 |
| TCONS_00018728 | AC012189 5EST gb | at1g15010 | -2.9 | -0.8 | -1.9 | C05:1..32828328:5659318-5660043 |
| TCONS_00021440 | -NA- | -- | -2.9 | -0.2 | -1.5 | C06:1..40704471:18829725-18845667 |
| TCONS_00024865 | transcription factor bHLH92 | at5g43650 | -2.9 | -0.4 | -1.1 | C07:1..48346208:43929-47253 |
| TCONS_00011284 | non-specific lipid-transfer At2g13820-like | at3g22600 | -2.9 | 1.0 | -0.6 | C03:1..57781463:24913396-24914659 |
| TCONS_00032763 | Dipeptide transport ATP-binding dppF | at2g21640 | -2.9 | -0.9 | -1.7 | C08:1..41516064:35650770-35652290 |
| TCONS_00011242 | -NA- | -- | -2.9 | -0.5 | -2.0 | C03:1..57781463:24120096-24120902 |
| TCONS_00009401 | multidrug and toxin extrusion 2-like | at5g52050 | -2.8 | -0.0 | -1.2 | C03:1..57781463:7675814-7677807 |
| TCONS_00000728 | sec7-like guanine nucleotide exchange family protein | at4g35380 | -2.8 | -1.1 | -1.4 | C01:1..38761720:1858769-1859537 |
| TCONS_00032012 | beta-1,3-glucanase 2 | at3g57260 | -2.8 | 1.4 | -0.6 | C08:1..41516064:30761349-30765468 |
| TCONS_00005265 | glycosyltransferase family 2 | at5g60700 | -2.8 | -0.3 | -1.3 | C02:1..44046003:6841038-6843852 |
| TCONS_00035248 | -NA- | at5g46910 | -2.8 | -0.9 | -1.4 | C09:1..40126856:8743271-8747039 |
| TCONS_00001843 | cation H(+) antiporter 18-like | at4g23700 | -2.8 | 0.2 | -0.7 | C01:1..38761720:10379717-10381285 |
| TCONS_00017689 | beta- insoluble isoenzyme CWINV1 | at3g13790 | -2.8 | -0.2 | -1.1 | C05:1..32828328:29290340-29297928 |
| TCONS_00031166 | -NA- | -- | -2.8 | -0.4 | -0.4 | C08:1..41516064:21650351-21651637 |
| TCONS_00015594 | AP2 domain transcription factor | at2g33710 | -2.8 | -0.0 | -1.2 | C04:1..40895475:20351088-20353223 |
| TCONS_00030926 | ATPDR7 PDR7 | at1g15210 | -2.8 | -1.5 | -1.9 | C08:1..41516064:19529552-19530582 |
| TCONS_00029128 | hypothetical protein ARALYDRAFT 333079 | -- | -2.8 | -1.0 | -1.5 | C07:1..48346208:47130996-47131712 |
| TCONS_00011793 | -NA- | -- | -2.7 | -0.5 | -1.3 | C03:1..57781463:30884342-30885637 |
| TCONS_00017212 | Laccase 12 | at2g40370 | -2.7 | -0.6 | -1.9 | C04:1..40895475:38615581-38617104 |
| TCONS_00020596 | GDSL esterase lipase 4 | at3g14225 | -2.7 | 0.7 | -0.4 | C05:1..32828328:28863371-28865042 |
| TCONS_00009085 | zinc finger | at5g59820 | -2.7 | -0.5 | -0.8 | C03:1..57781463:5166355-5167590 |
| TCONS_00015945 | hypothetical protein EUTSA v10027932mg | at5g38310 | -2.7 | -0.3 | -2.3 | C04:1..40895475:25519949-25520864 |
| TCONS_00008453 | -NA- | at5g09530 | -2.7 | -1.0 | -1.0 | C03:1..57781463:1740162-1741368 |
| TCONS_00002326 | -NA- | at3g09110 | -2.7 | -0.6 | -1.8 | C01:1..38761720:15110236-15112673 |
| TCONS_00027237 | cytochrome P450 | at3g26190 | -2.7 | 0.7 | -0.8 | C07:1..48346208:32089851-32090675 |
| TCONS_00031049 | pyridoxal phosphate phosphatase-related | at1g17710 | -2.7 | -0.5 | -0.9 | C08:1..41516064:20543412-20544838 |
| TCONS_00024327 | Poly polymerase | at1g76600 | -2.7 | -0.0 | -0.3 | C06:1..40704471:31764845-31765895 |
| TCONS_00019154 | gibberellin 2-beta-dioxygenase 2 | at1g30040 | -2.7 | -1.0 | -2.5 | C05:1..32828328:11875592-11877914 |
| TCONS_00015754 | metal ion binding | at3g56891 | -2.7 | -0.8 | -1.6 | C04:1..40895475:21977546-21978664 |
| TCONS_00003467 | sequence-specific DNA binding transcription factor | at3g14180 | -2.7 | -0.5 | -0.6 | C01:1..38761720:34777139-34779097 |
| TCONS_00001844 | cation H(+) antiporter 18-like | at4g23700 | -2.7 | -0.2 | -0.8 | C01:1..38761720:10381384-10382389 |
| TCONS_00009577 | Spotted leaf | at2g35930 | -2.7 | -0.5 | -1.4 | C03:1..57781463:8905168-8906778 |
| TCONS_00036339 | -NA- | -- | -2.6 | -0.4 | 0.0 | C09:1..40126856:27813119-27813845 |
| TCONS_00036563 | blue copper binding | at5g20230 | -2.6 | -0.2 | -1.0 | C09:1..40126856:30298080-30299630 |
| TCONS_00009333 | CBS domain-containing CBSX5-like | at5g53750 | -2.6 | -1.1 | -2.4 | C03:1..57781463:6938193-6939753 |
| TCONS_00032685 | bHLH transcription factor | at2g22760 | -2.6 | -0.4 | -1.8 | C08:1..41516064:35106957-35109350 |
| TCONS_00005664 | unnamed protein product | at1g67920 | -2.6 | -0.9 | -1.3 | C02:1..44046003:11848783-11849327 |
| TCONS_00036104 | cytochrome P450 monooxygenase | at5g57220 | -2.6 | 0.5 | -0.9 | C09:1..40126856:24991334-24992990 |
| TCONS_00019915 | cytochrome P450 | at3g20140 | -2.6 | -2.4 | -1.7 | C05:1..32828328:22421879-22424258 |
| TCONS_00031977 | metal ion binding | at3g56891 | -2.6 | -1.2 | -2.0 | C08:1..41516064:30515866-30516976 |
| TCONS_00021147 | -NA- | -- | -2.6 | 0.9 | -0.3 | C06:1..40704471:1371113-1376520 |
| TCONS_00004249 | omega-3 fatty acid desaturase | p48618|fad3c_brana | -2.6 | -0.8 | -1.8 | C02:1..44046003:10632458-10642384 |
| TCONS_00009282 | O-Glycosyl hydrolases family 17 | at5g55180 | -2.6 | -1.1 | -2.2 | C03:1..57781463:6438266-6440020 |
| TCONS_00024841 | -NA- | at1g48330 | -2.6 | 0.3 | -1.8 | C06:1..40704471:40281129-40282053 |
| TCONS_00028398 | cationic amino acid | at4g21120 | -2.6 | 0.0 | -1.6 | C07:1..48346208:41930815-41931525 |
| TCONS_00032719 | -NA- | -- | -2.6 | -0.4 | -1.1 | C08:1..41516064:35295122-35296133 |
| TCONS_00025019 | receptor kinase HSL1-like | at5g25930 | -2.6 | -0.0 | -1.0 | C07:1..48346208:35349381-35365785 |
| TCONS_00037189 | AT5g13210 T31B5 30 | at5g13210 | -2.6 | 0.0 | -1.0 | C09:1..40126856:35589942-35592227 |
| TCONS_00024868 | Cys2 His2-type zinc finger 3 | at5g43170 | -2.6 | -0.2 | -1.7 | C07:1..48346208:552888-564382 |
| TCONS_00021469 | -NA- | -- | -2.5 | -0.5 | -3.4 | C06:1..40704471:31279119-31282776 |
| TCONS_00004528 | 2-oxoglutarate and fe -dependent oxygenase superfamily protein | at5g05600 | -2.5 | -0.2 | -1.9 | C02:1..44046003:1859779-1861985 |
| TCONS_00037188 | -NA- | at5g13220 | -2.5 | -0.0 | -1.1 | C09:1..40126856:35583686-35585860 |
| TCONS_00034491 | cytochrome p450 | at2g02580 | -2.5 | -0.9 | -1.2 | C09:1..40126856:1823550-1825710 |
| TCONS_00008465 | -NA- | at5g09876 | -2.5 | -0.5 | -1.2 | C03:1..57781463:1821310-1822145 |
| TCONS_00012358 | zinc finger | at1g27730 | -2.5 | -0.2 | -1.9 | C03:1..57781463:43718729-43719765 |
| TCONS_00008700 | core-2 I-branching beta-1,6-N-acetylglucosaminyltransferase family | at5g16170 | -2.5 | -0.3 | -1.2 | C03:1..57781463:3012872-3014796 |
| TCONS_00021528 | cinnamoyl- reductase 2-2 | at1g80820 | -2.5 | -0.5 | -1.6 | C06:1..40704471:61740-63061 |
| TCONS_00031019 | lipoxygenase | at1g17420 | -2.5 | -0.3 | -1.3 | C08:1..41516064:20153499-20188838 |
| TCONS_00028470 | cytochrome P450 | at4g22690 | -2.5 | 0.4 | -1.0 | C07:1..48346208:42537003-42538885 |
| TCONS_00031932 | manganese superoxide dismutase | at3g56350 | -2.5 | -0.3 | -1.4 | C08:1..41516064:30226943-30228499 |
| TCONS_00032256 | glycosyl hydrolase family 1 | at3g60120 | -2.5 | 1.0 | -1.1 | C08:1..41516064:32069386-32071307 |
| TCONS_00013276 | At1g70420 F17O7 4 | at1g70420 | -2.5 | -0.1 | -1.5 | C03:1..57781463:55518243-55519261 |
| TCONS_00009747 | protease inhibitor | -- | -2.5 | -0.4 | -0.8 | C03:1..57781463:10518188-10518967 |
| TCONS_00016361 | fatty acid binding 2 | at2g26310 | -2.5 | -1.0 | -0.2 | C04:1..40895475:30040004-30040918 |
| TCONS_00030235 | At1g32920 F9L11 25 | at1g32920 | -2.5 | -0.7 | -1.7 | C08:1..41516064:10276561-10279873 |
| TCONS_00028326 | heat stress transcription factor A-4a-like | at4g18880 | -2.5 | -0.3 | -1.2 | C07:1..48346208:41215080-41217249 |
| TCONS_00005873 | lipoxygenase 3 | at1g72520 | -2.5 | 0.3 | -0.8 | C02:1..44046003:15958758-15962882 |
| TCONS_00016117 | short-chain type dehydrogenase reductase-like | at4g13180 | -2.5 | -0.0 | -1.5 | C04:1..40895475:27439798-27440758 |
| TCONS_00009797 | PBS1-like 1 kinase | at2g39660 | -2.4 | 0.1 | -1.0 | C03:1..57781463:10894186-10894963 |
| TCONS_00010349 | -NA- | -- | -2.4 | -0.6 | -2.1 | C03:1..57781463:15441090-15441809 |
| TCONS_00035390 | -NA- | -- | -2.4 | 0.1 | -1.3 | C09:1..40126856:11011395-11012538 |
| TCONS_00036437 | PHD finger MALE STERILITY 1-like | at5g22260 | -2.4 | -0.6 | -1.4 | C09:1..40126856:29143492-29144946 |
| TCONS_00027568 | peptidyl-prolyl cis-trans isomerase FKBP65 | at5g48570 | -2.4 | -0.1 | 0.1 | C07:1..48346208:34852243-34853245 |
| TCONS_00020637 | beta- insoluble isoenzyme CWINV1 | at3g13790 | -2.4 | -0.0 | -1.0 | C05:1..32828328:29290340-29297928 |
| TCONS_00032657 | hypothetical protein ARALYDRAFT 900807 | at2g23270 | -2.4 | 1.1 | -1.7 | C08:1..41516064:34803930-34804629 |
| TCONS_00021901 | SHI-related sequence 7 | at1g75520 | -2.4 | 0.0 | -0.4 | C06:1..40704471:2197642-2199952 |
| TCONS_00015057 | N-hydroxycinnamoyl benzoyltransferase | at5g42830 | -2.4 | -0.1 | -1.6 | C04:1..40895475:11949248-11950561 |
| TCONS_00005428 | protein eceriferum 3-like | at5g57800 | -2.4 | -0.7 | -2.0 | C02:1..44046003:8875621-8877500 |
| TCONS_00000531 | BTB and TAZ domain 4 | at4g37610 | -2.4 | 0.0 | -0.4 | C01:1..38761720:724344-726112 |
| TCONS_00036564 | blue copper binding | at5g20230 | -2.4 | -0.3 | -1.0 | C09:1..40126856:30299939-30300722 |
| TCONS_00005701 | protein ida | at1g68765 | -2.4 | -0.1 | -1.0 | C02:1..44046003:12609449-12610246 |
| TCONS_00027119 | ribonuclease 1 | at2g02990 | -2.4 | 0.0 | -1.7 | C07:1..48346208:30722275-30723653 |
| TCONS_00022094 | nudix hydrolase 4 | at1g73540 | -2.4 | 0.1 | -0.5 | C06:1..40704471:3642221-3643792 |
| TCONS_00003812 | exocyst subunit exo70 family H4 | at3g09520 | -2.4 | 0.4 | -0.4 | C01:1..38761720:37490725-37492953 |
| TCONS_00004587 | cytosolic sulfotransferase 15-like | at5g07010 | -2.4 | 0.3 | -3.0 | C02:1..44046003:2108784-2110354 |
| TCONS_00028119 | calcineurin B 1 | at4g17615 | -2.4 | 0.2 | -2.1 | C07:1..48346208:39014115-39015378 |
| TCONS_00028413 | MYB transcription partial | at4g21440 | -2.4 | -0.0 | -0.5 | C07:1..48346208:42002990-42003930 |
| TCONS_00020911 | omega-3 fatty acid desaturase | at3g11170 | -2.4 | -0.6 | -1.7 | C05:1..32828328:31621205-31624114 |
| TCONS_00033718 | polygalacturonase At1g48100-like | at1g02460 | -2.3 | 0.3 | -1.8 | C08:1..41516064:41075460-41076773 |
| TCONS_00014637 | -NA- | at2g38790 | -2.3 | -0.4 | -1.9 | C04:1..40895475:6399338-6400385 |
| TCONS_00011738 | ethylene-responsive transcription factor ABR1-like | at5g64750 | -2.3 | 0.1 | -0.1 | C03:1..57781463:30375665-30376620 |
| TCONS_00023943 | concanavalin A-like lectin kinase | at3g59700 | -2.3 | 0.2 | -0.5 | C06:1..40704471:27608725-27610024 |
| TCONS_00011691 | -NA- | at2g18690 | -2.3 | -0.0 | -1.5 | C03:1..57781463:29786083-29787774 |
| TCONS_00036327 | zinc finger | at5g59820 | -2.3 | -0.1 | -1.8 | C09:1..40126856:27753436-27754196 |
| TCONS_00011285 | protease inhibitor seed storage lipid transfer family | at3g22620 | -2.3 | 0.0 | -0.3 | C03:1..57781463:24916392-24917482 |
| TCONS_00024272 | Glutathione S-transferase TAU 19 | at1g78340 | -2.3 | -0.7 | -1.6 | C06:1..40704471:31335832-31336323 |
| TCONS_00006070 | protein | at1g76600 | -2.3 | -0.1 | -0.5 | C02:1..44046003:19137639-19139107 |
| TCONS_00004527 | 2-oxoglutarate and fe -dependent oxygenase superfamily protein | at5g05600 | -2.3 | -0.3 | -1.8 | C02:1..44046003:1858271-1859234 |
| TCONS_00011107 | retroelement pol poly | at4g23160 | -2.3 | 0.6 | 0.8 | C03:1..57781463:22610106-22614339 |
| TCONS_00018828 | Solute carrier family 22 member | at1g16370 | -2.3 | 0.1 | -0.7 | C05:1..32828328:6436167-6438363 |
| TCONS_00032626 | leucine-rich repeat receptor-like serine threonine- kinase At2g24130-like | at2g24130 | -2.3 | -0.0 | -0.5 | C08:1..41516064:34496632-34500042 |
| TCONS_00005316 | zinc finger | at5g59820 | -2.3 | -0.5 | -0.9 | C02:1..44046003:7567382-7568185 |
| TCONS_00000632 | salicylate o-methyltransferase-like | at4g36470 | -2.3 | -0.4 | -1.5 | C01:1..38761720:1283035-1285760 |
| TCONS_00007018 | xyloglucan glycosyltransferase 4-like | at3g28180 | -2.3 | -0.1 | -1.6 | C02:1..44046003:39204549-39205464 |
| TCONS_00009328 | lipid-binding START domain-containing | at5g54170 | -2.3 | -0.3 | -0.9 | C03:1..57781463:6819320-6822501 |
| TCONS_00028788 | hypothetical protein EUTSA v10026985mg | at4g28085 | -2.3 | -0.2 | -1.4 | C07:1..48346208:44619954-44620474 |
| TCONS_00037800 | -NA- | at5g05250 | -2.3 | -0.3 | -1.0 | C09:1..40126856:39170763-39171974 |
| TCONS_00010410 | HSP22 | at4g10250 | -2.3 | -0.1 | 0.3 | C03:1..57781463:16053634-16054660 |
| TCONS_00025703 | serine threonine- kinase OXI1-like | at3g25250 | -2.3 | -0.0 | -0.4 | C07:1..48346208:6347794-6349460 |
| TCONS_00005893 | inorganic pyrophosphatase 2-like | at1g73010 | -2.2 | 0.1 | -1.0 | C02:1..44046003:16293648-16295325 |
| TCONS_00037492 | NAC domain-containing 102 | at5g08790 | -2.2 | -0.3 | -1.2 | C09:1..40126856:37611187-37612830 |
| TCONS_00036013 | O-Glycosyl hydrolases family 17 | at5g55180 | -2.2 | -0.4 | -0.7 | C09:1..40126856:23649329-23651551 |
| TCONS_00011519 | TIR-NBS-LRR class disease resistance | at5g41750 | -2.2 | -0.4 | -2.0 | C03:1..57781463:27428088-27430533 |
| TCONS_00004364 | bahd acyltransferase dcr-like | at5g01210 | -2.2 | -0.4 | -1.5 | C02:1..44046003:171099-173028 |
| TCONS_00037786 | dehydration-responsive element-binding 2A | at5g05410 | -2.2 | -0.2 | -0.0 | C09:1..40126856:39114313-39116884 |
| TCONS_00011087 | GATA transcription factor 17 | at3g16870 | -2.2 | -0.3 | -2.3 | C03:1..57781463:22368084-22368771 |
| TCONS_00009713 | WRKY transcription factor 33 | at2g38470 | -2.2 | -0.3 | -1.1 | C03:1..57781463:10356829-10360216 |
| TCONS_00032034 | endo-polygalacturonase 1 | at3g57510 | -2.2 | -0.0 | -0.9 | C08:1..41516064:30909574-30911335 |
| TCONS_00017194 | alpha beta hydrolase related | at2g40095 | -2.2 | -0.1 | -0.8 | C04:1..40895475:38438908-38441537 |
| TCONS_00017000 | phenylalanine ammonia-lyase | at2g37040 | -2.2 | -1.3 | -2.6 | C04:1..40895475:37110384-37112545 |
| TCONS_00021797 | calcium-binding CML38 | at1g76650 | -2.2 | -0.7 | -1.4 | C06:1..40704471:1525068-1526095 |
| TCONS_00017130 | MLO 2 | at2g39200 | -2.2 | 0.1 | -0.8 | C04:1..40895475:38018844-38019630 |
| TCONS_00010070 | cysteine histidine-rich C1 domain-containing | at2g44380 | -2.2 | 0.0 | -1.5 | C03:1..57781463:12963722-12964944 |
| TCONS_00005833 | uncharacterized protein | at1g71910 | -2.2 | -0.0 | -0.9 | C02:1..44046003:14988411-14989526 |
| TCONS_00024603 | glutathione S-transferase TAU 11 | at1g69930 | -2.2 | 0.3 | -0.9 | C06:1..40704471:35021451-35022604 |
| TCONS_00015959 | calmodulin-binding heat-shock | at5g37710 | -2.2 | -0.3 | -0.8 | C04:1..40895475:25881142-25882542 |
| TCONS_00016905 | plant glycogenin-like starch initiation 7 | at2g35710 | -2.2 | -0.1 | -1.2 | C04:1..40895475:36332844-36333815 |
| TCONS_00033802 | glutathione peroxidase | at2g48150 | -2.2 | -0.4 | -0.6 | C08:1..41516064:41511787-41513473 |
| TCONS_00028755 | Intracellular transport USO1 isoform 5 | at4g27610 | -2.2 | -0.8 | -0.4 | C07:1..48346208:44478627-44479755 |
| TCONS_00011523 | AGD2-like defense response 1 | at2g13810 | -2.2 | 0.6 | -1.1 | C03:1..57781463:27509787-27511206 |
| TCONS_00020294 | Coiled-coil domain-containing 109A | at1g09575 | -2.2 | -0.4 | -1.4 | C05:1..32828328:26145199-26147286 |
| TCONS_00011428 | cysteine-rich receptor kinase 11 isoform X2 | at4g23190 | -2.2 | -0.6 | -1.4 | C03:1..57781463:26797507-26798560 |
| TCONS_00006824 | ribonuclease t2 | at2g02990 | -2.2 | -0.1 | -1.9 | C02:1..44046003:35578535-35579915 |
| TCONS_00024636 | -NA- | -- | -2.2 | -0.0 | -0.4 | C06:1..40704471:35682772-35683696 |
| TCONS_00025076 | FAD-binding Berberine family | at4g20830 | -2.2 | 1.1 | -1.0 | C07:1..48346208:41857844-41867677 |
| TCONS_00016122 | serine threonine kinase | at4g23180 | -2.2 | 0.6 | -0.4 | C04:1..40895475:27507900-27509214 |
| TCONS_00028567 | -NA- | -- | -2.2 | 0.0 | -2.2 | C07:1..48346208:43480675-43483475 |
| TCONS_00025476 | mitogen-activated kinase kinase kinase 19 | at5g67080 | -2.2 | -0.3 | -1.0 | C07:1..48346208:1488944-1490483 |
| TCONS_00010263 | phospholipase A 2A | at2g26560 | -2.2 | 0.6 | -0.2 | C03:1..57781463:14597739-14598898 |
| TCONS_00029597 | -NA- | at1g13520 | -2.2 | -0.1 | -1.1 | C08:1..41516064:19077384-19083032 |
| TCONS_00017531 | ATP binding cassette subfamily B4 isoform 2 | at2g47000 | -2.2 | -0.3 | -1.0 | C04:1..40895475:40848637-40850338 |
| TCONS_00007292 | -NA- | -- | -2.2 | 0.2 | -1.0 | C02:1..44046003:42560779-42562397 |
| TCONS_00032443 | xyloglucan 6-xylosyltransferase | at3g62720 | -2.2 | -0.3 | -1.3 | C08:1..41516064:33340751-33341222 |
| TCONS_00007273 | ring finger | at5g27420 | -2.2 | 0.1 | -0.9 | C02:1..44046003:42485886-42487593 |
| TCONS_00010262 | patatin group A-3-like | at2g26560 | -2.2 | 0.8 | -0.3 | C03:1..57781463:14596319-14597583 |
| TCONS_00019369 | -NA- | at1g19180 | -2.2 | 0.0 | -0.8 | C05:1..32828328:14662317-14664075 |
| TCONS_00001420 | calcineurin b-like protein 1 | at4g17615 | -2.2 | -0.1 | -0.9 | C01:1..38761720:5944976-5948097 |
| TCONS_00023904 | aspartyl protease family | at3g59080 | -2.1 | -0.1 | -1.2 | C06:1..40704471:27199604-27201965 |
| TCONS_00019704 | lipoxygenase 5 | at3g22400 | -2.1 | -0.9 | -1.4 | C05:1..32828328:18816932-18819287 |
| TCONS_00007080 | proline dehydrogenase | at3g30775 | -2.1 | -0.1 | 0.0 | C02:1..44046003:40281813-40284695 |
| TCONS_00000057 | FAD-linked oxidoreductase | at4g20860 | -2.1 | -0.1 | -1.1 | C01:1..38761720:7535762-7544286 |
| TCONS_00035743 | C-repeat binding factor 4 | at5g51990 | -2.1 | -1.3 | -0.7 | C09:1..40126856:18732236-18733228 |
| TCONS_00018555 | ACC oxidase | at1g62380 | -2.1 | 0.5 | -1.2 | C05:1..32828328:3896863-3897973 |
| TCONS_00017532 | ATP binding cassette subfamily B4 isoform 2 | at2g47000 | -2.1 | -0.7 | -0.8 | C04:1..40895475:40850418-40852393 |
| TCONS_00001754 | -NA- | -- | -2.1 | -0.6 | -0.4 | C01:1..38761720:8913473-8914291 |
| TCONS_00037700 | partial | at5g06320 | -2.1 | -0.3 | -0.9 | C09:1..40126856:38658078-38659605 |
| TCONS_00011900 | cytochrome P450 | at5g36220 | -2.1 | -0.5 | -2.1 | C03:1..57781463:33002885-33004257 |
| TCONS_00005305 | CCT motif family | at5g59990 | -2.1 | -0.7 | -1.0 | C02:1..44046003:7484537-7486701 |
| TCONS_00033516 | BONZAI 3-like | at1g08860 | -2.1 | -0.1 | -0.7 | C08:1..41516064:39861046-39862379 |
| TCONS_00029419 | -NA- | at1g19180 | -2.1 | 0.0 | -1.6 | C08:1..41516064:21111675-21165176 |
| TCONS_00008561 | -NA- | at5g13660 | -2.1 | -0.1 | -1.3 | C03:1..57781463:2292451-2298823 |
| TCONS_00032867 | leucine-rich repeat family | at2g19780 | -2.1 | -0.5 | -1.2 | C08:1..41516064:36148732-36150751 |
| TCONS_00034769 | hexose transporter | at5g26340 | -2.1 | -0.0 | -0.6 | C09:1..40126856:3546235-3550587 |
| TCONS_00027763 | G-type lectin S-receptor-like serine threonine- kinase At5g24080-like | at5g24080 | -2.1 | 0.0 | -0.7 | C07:1..48346208:36412871-36421611 |
| TCONS_00028846 | -NA- | -- | -2.1 | 0.5 | 0.2 | C07:1..48346208:45145077-45146556 |
| TCONS_00020885 | -NA- | at3g47680 | -2.1 | -1.0 | -1.1 | C05:1..32828328:31374481-31375846 |
| TCONS_00026406 | At1g68440 T2E12 1 | at1g25400 | -2.1 | -0.2 | -0.3 | C07:1..48346208:21529385-21531678 |
| TCONS_00028550 | metal-nicotianamine transporter YSL1-like | at4g24120 | -2.1 | -0.6 | -1.8 | C07:1..48346208:43401456-43402958 |
| TCONS_00028551 | hypothetical protein EUTSA v10026459mg | at4g24130 | -2.1 | -0.7 | -2.3 | C07:1..48346208:43403720-43404556 |
| TCONS_00031195 | Indole glucosinolate O-methyltransferase 4 | at1g21130 | -2.1 | 1.5 | -0.4 | C08:1..41516064:21870656-21879099 |
| TCONS_00029255 | L-ascorbate oxidase | at4g39830 | -2.1 | 0.3 | -0.5 | C07:1..48346208:47996556-47997957 |
| TCONS_00031015 | TIFY 11A | at1g17380 | -2.1 | -0.4 | -1.5 | C08:1..41516064:20077291-20079028 |
| TCONS_00017154 | PBS1-like 1 kinase | at2g39660 | -2.1 | -0.9 | -1.2 | C04:1..40895475:38194846-38195805 |
| TCONS_00037634 | peptide methionine sulfoxide reductase | at5g07460 | -2.1 | 1.2 | -0.7 | C09:1..40126856:38231054-38232157 |
| TCONS_00037613 | CBL-interacting kinase 2 | at5g07070 | -2.1 | -0.5 | -1.1 | C09:1..40126856:38048258-38049980 |
| TCONS_00018783 | pleiotropic drug resistance 1-like | at1g15520 | -2.1 | 0.2 | -0.1 | C05:1..32828328:5903321-5904609 |
| TCONS_00006712 | by genscan and genefinder | at2g01300 | -2.1 | -0.0 | -0.4 | C02:1..44046003:34160676-34161661 |
| TCONS_00014408 | plant glycogenin-like starch initiation 7 | at2g35710 | -2.0 | -0.4 | -1.9 | C04:1..40895475:3845040-3846047 |
| TCONS_00004514 | dehydration-responsive element-binding protein 2a | at5g05410 | -2.0 | -0.1 | -0.3 | C02:1..44046003:1803094-1804863 |
| TCONS_00010664 | -NA- | at4g02170 | -2.0 | -0.3 | -1.4 | C03:1..57781463:18864321-18865262 |
| TCONS_00021976 | GNS1 SUR4 membrane family | at1g75000 | -2.0 | 0.2 | -0.1 | C06:1..40704471:2956825-2959092 |
| TCONS_00007753 | hypothetical protein CARUB v10009513mg | at1g30170 | -2.0 | -0.4 | -1.4 | C03:1..57781463:45228778-45274055 |
| TCONS_00007808 | probable carboxylesterase 15 | at5g06570 | -2.0 | -0.1 | -0.8 | C03:1..57781463:1089886-1095802 |
| TCONS_00003400 | ethylene-responsive transcription factor erf109-like | at4g34410 | -2.0 | -0.9 | -1.8 | C01:1..38761720:33824967-33826210 |
| TCONS_00015645 | cytochrome P450 | at2g34500 | -2.0 | 0.5 | -0.5 | C04:1..40895475:20925419-20927673 |
| TCONS_00026777 | berberine bridge enzyme | at5g44380 | -2.0 | -0.1 | -0.7 | C07:1..48346208:27002960-27058693 |
| TCONS_00009192 | Galactosyltransferase family | at5g57500 | -2.0 | 0.1 | -1.0 | C03:1..57781463:5872644-5874537 |
| TCONS_00022319 | glutathione S-transferase | at1g69930 | -2.0 | 0.2 | -0.8 | C06:1..40704471:5192856-5194199 |
| TCONS_00020752 | -NA- | at3g12320 | -2.0 | -0.6 | -0.9 | C05:1..32828328:30210841-30211927 |
| TCONS_00026112 | Cupredoxin superfamily | at2g15780 | -2.0 | 0.0 | -0.6 | C07:1..48346208:14859847-14861796 |
| TCONS_00009556 | glucomannan 4-beta-mannosyltransferase 9-like | at2g35658 | -2.0 | -0.4 | -1.2 | C03:1..57781463:8735771-8736900 |
| TCONS_00022648 | Pre-mRNA cleavage complex II | at1g66500 | -2.0 | 0.0 | -0.5 | C06:1..40704471:8851921-8853584 |
| TCONS_00005430 | -NA- | -- | -2.0 | -0.7 | -1.8 | C02:1..44046003:8879276-8879522 |
| TCONS_00003826 | probable glutathione S-transferase-like | at3g09270 | -2.0 | -0.7 | -1.7 | C01:1..38761720:37652222-37653848 |
| TCONS_00019705 | lipoxygenase | at3g22400 | -2.0 | -1.5 | -1.3 | C05:1..32828328:18819424-18820874 |
| TCONS_00014371 | pseudo-response regulator 9 | at2g46790 | -2.0 | -0.4 | -0.7 | C04:1..40895475:3453872-3455836 |
| TCONS_00018560 | early-responsive to dehydration stress-related | at1g11960 | -2.0 | -0.6 | -1.9 | C05:1..32828328:3914957-3917318 |
| TCONS_00003811 | phosphatidate phosphatase LPIN3-like | at3g09560 | -2.0 | -0.0 | -1.3 | C01:1..38761720:37486127-37487687 |
| TCONS_00020751 | MATH and LRR domain-containing isoform 2 | at3g12320 | -2.0 | -0.5 | -0.9 | C05:1..32828328:30209935-30210712 |
| TCONS_00002277 | vacuolar iron transporter-like protein | at4g27860 | -2.0 | -0.4 | -2.0 | C01:1..38761720:14063199-14063975 |
| TCONS_00037760 | myb family transcription factor | at5g05790 | -2.0 | -0.5 | -1.2 | C09:1..40126856:38974579-38976340 |
| TCONS_00019911 | -NA- | -- | -2.0 | -0.3 | -1.1 | C05:1..32828328:22345985-22359730 |
| TCONS_00035168 | uncharacterized protein | at5g45630 | -2.0 | -0.5 | -0.6 | C09:1..40126856:7427312-7428279 |
| TCONS_00034459 | probable indole-3-acetic acid-amido synthetase -like | at4g03400 | -2.0 | -0.4 | -2.2 | C09:1..40126856:1594791-1595652 |
| TCONS_00007319 | Cam-binding 60-like G | at5g26920 | -2.0 | -0.4 | -1.0 | C02:1..44046003:42704302-42705727 |
| TCONS_00002276 | vacuolar iron transporter-like protein | at4g27860 | -2.0 | -0.6 | -1.7 | C01:1..38761720:14061791-14063110 |
| TCONS_00018449 | -NA- | -- | -2.0 | -0.0 | -0.5 | C05:1..32828328:3165735-3166838 |
| TCONS_00012438 | CBL-interacting serine threonine- kinase 18 | at1g29230 | -2.0 | 0.1 | -0.5 | C03:1..57781463:44616181-44618352 |
| TCONS_00032036 | polygalacturonase ADPG1 | at3g57510 | -2.0 | -0.1 | -0.7 | C08:1..41516064:30913010-30913398 |
| TCONS_00027955 | probable galacturonosyltransferase-like 1-like | at3g50760 | -2.0 | 0.0 | -1.1 | C07:1..48346208:37754750-37756316 |
| TCONS_00012577 | phospholipase (PEARLI 4) domain-containing | at4g38560 | -2.0 | 0.3 | -0.6 | C03:1..57781463:45903850-45906944 |
| TCONS_00014597 | dehydration-responsive element-binding 2E | at2g38340 | -2.0 | -0.3 | -0.7 | C04:1..40895475:5979969-5981280 |
| TCONS_00032639 | TET8 ARATH ame: Full=Tetraspanin-8 | at2g23810 | -2.0 | -0.1 | -0.7 | C08:1..41516064:34596565-34599058 |
| TCONS_00019706 | lipoxygenase | at3g22400 | -2.0 | -1.6 | -1.3 | C05:1..32828328:18820999-18822354 |
| TCONS_00003810 | phosphatidate phosphatase LPIN3-like | at3g09560 | -1.9 | -0.7 | -1.4 | C01:1..38761720:37483850-37485913 |
| TCONS_00002315 | high affinity nitrate transporter | at3g45060 | -1.9 | -0.1 | -1.0 | C01:1..38761720:14744419-14747622 |
| TCONS_00032035 | polygalacturonase ADPG1 | at3g57510 | -1.9 | -0.3 | -0.6 | C08:1..41516064:30911583-30912742 |
| TCONS_00019557 | -NA- | -- | -1.9 | -0.4 | -2.1 | C05:1..32828328:16843061-16843938 |
| TCONS_00011101 | S-adenosylmethionine synthetase | at3g17390 | -1.9 | -0.2 | -1.9 | C03:1..57781463:22532161-22534194 |
| TCONS_00010788 | -NA- | at3g01650 | -1.9 | -0.2 | -0.4 | C03:1..57781463:19743077-19746485 |
| TCONS_00037743 | cytochrome P450 | at2g30750 | -1.9 | 1.5 | -0.3 | C09:1..40126856:38893046-38895333 |
| TCONS_00001553 | -NA- | -- | -1.9 | 0.0 | -0.9 | C01:1..38761720:7046738-7048703 |
| TCONS_00027894 | hypothetical protein EUTSA v10005448mg | at5g61340 | -1.9 | -0.0 | -0.7 | C07:1..48346208:37231008-37232650 |
| TCONS_00001996 | heat shock protein | at4g25200 | -1.9 | -0.3 | -0.0 | C01:1..38761720:11430729-11431687 |
| TCONS_00002207 | calcium-binding PBP1-like | at4g27280 | -1.9 | -0.5 | -1.5 | C01:1..38761720:13432930-13433438 |
| TCONS_00019117 | phospholipase A1- chloroplastic-like | at1g30370 | -1.9 | 0.1 | -1.3 | C05:1..32828328:11035269-11037180 |
| TCONS_00000561 | hypothetical protein CARUB v10007485mg | -- | -1.9 | 2.0 | -0.3 | C01:1..38761720:898989-899581 |
| TCONS_00032037 | endo-polygalacturonase 1 | at3g57510 | -1.9 | -0.3 | -0.5 | C08:1..41516064:30913596-30913979 |
| TCONS_00016989 | S-adenosylmethionine synthase 2-like | at2g36880 | -1.9 | 0.0 | -1.8 | C04:1..40895475:37013006-37014430 |
| TCONS_00022364 | uncharacterized protein | -- | -1.9 | -0.3 | -0.5 | C06:1..40704471:5778394-5779222 |
| TCONS_00006660 | ethylene responsive element binding factor 2 | at5g47220 | -1.9 | -0.2 | -0.6 | C02:1..44046003:32857213-32858483 |
| TCONS_00024298 | -NA- | -- | -1.9 | 0.4 | -0.5 | C06:1..40704471:31490254-31491837 |
| TCONS_00000094 | cellulose synthase isolog | at4g24000 | -1.9 | 0.1 | -1.0 | C01:1..38761720:10610126-10617654 |
| TCONS_00019951 | -NA- | at3g19920 | -1.9 | -0.1 | 0.0 | C05:1..32828328:22735135-22736184 |
| TCONS_00036542 | leucine-rich repeat receptor kinase | at5g20480 | -1.9 | -0.5 | -2.1 | C09:1..40126856:30126146-30129161 |
| TCONS_00035835 | F-box FBD LRR-repeat | at1g51370 | -1.9 | -0.2 | -0.8 | C09:1..40126856:20826459-20828713 |
| TCONS_00005429 | -NA- | at5g57800 | -1.9 | -1.0 | -2.0 | C02:1..44046003:8877895-8879172 |
| TCONS_00037704 | cytokinin riboside 5 -monophosphate phosphoribohydrolase LOG7-like | at5g06300 | -1.9 | -0.3 | -1.0 | C09:1..40126856:38675210-38678033 |
| TCONS_00029193 | heat shock transcription factor HSF4 | at4g36990 | -1.9 | 0.3 | -1.4 | C07:1..48346208:47544337-47545917 |
| TCONS_00019818 | NAD(H) kinase 1 | at3g21070 | -1.9 | 0.0 | -0.8 | C05:1..32828328:20744702-20746187 |
| TCONS_00016925 | RING-H2 finger ATL70-like | at2g35910 | -1.9 | -0.0 | -0.4 | C04:1..40895475:36466160-36467344 |
| TCONS_00033005 | TIFY 11A | at1g17380 | -1.9 | -0.5 | -1.5 | C08:1..41516064:36896185-36898380 |
| TCONS_00022779 | Sodium calcium exchanger family calcium-binding EF hand family | at1g53210 | -1.9 | -0.3 | -1.8 | C06:1..40704471:10485978-10488795 |
| TCONS_00003572 | auxin-responsive family | at3g12830 | -1.9 | -0.2 | -0.6 | C01:1..38761720:35800722-35801593 |
| TCONS_00014182 | ARGOS | at2g44080 | -1.9 | 0.0 | -0.5 | C04:1..40895475:1797785-1798908 |
| TCONS_00028437 | hypothetical protein EUTSA v10026602mg | at4g21920 | -1.9 | -0.3 | -1.1 | C07:1..48346208:42197320-42198114 |
| TCONS_00011901 | cytochrome P450 | at5g36220 | -1.9 | -0.9 | -2.2 | C03:1..57781463:33004911-33005566 |
| TCONS_00032864 | nucleotide-diphospho-sugar transferases superfamily | at2g19880 | -1.9 | -0.5 | -1.4 | C08:1..41516064:36126659-36128097 |
| TCONS_00030667 | -NA- | -- | -1.9 | -0.4 | -2.0 | C08:1..41516064:17222609-17222807 |
| TCONS_00018782 | pleiotropic drug resistance 1-like | at1g15520 | -1.9 | 0.3 | -0.1 | C05:1..32828328:5901523-5902933 |
| TCONS_00028772 | salt tolerance 5 | at4g27890 | -1.9 | -0.4 | -0.8 | C07:1..48346208:44564238-44565578 |
| TCONS_00011614 | heat shock 70 | at5g02500 | -1.9 | -0.4 | -1.8 | C03:1..57781463:28794860-28797988 |
| TCONS_00000122 | OSBP(oxysterol binding )-related 4B | at4g25850 | -1.9 | -1.3 | -1.6 | C01:1..38761720:11938721-11947155 |
| TCONS_00007721 | ABC transporter | at3g47780 | -1.9 | 0.0 | -1.2 | C03:1..57781463:39807478-39815034 |
| TCONS_00032831 | drought responsive element binding 5 | at2g20880 | -1.9 | 0.2 | -0.8 | C08:1..41516064:35936519-35938037 |
| TCONS_00009855 | zinc finger CCCH domain-containing 29 | at2g40140 | -1.9 | -0.3 | -0.8 | C03:1..57781463:11172807-11175392 |
| TCONS_00019804 | -NA- | at1g76590 | -1.9 | -0.8 | -1.6 | C05:1..32828328:20350907-20352638 |
| TCONS_00029484 | KDEL motif-containing 1 | at3g61290 | -1.8 | 0.2 | -1.0 | C08:1..41516064:32611396-32627311 |
| TCONS_00026046 | receptor like 19 | at1g45616 | -1.8 | -0.2 | -1.9 | C07:1..48346208:13622982-13626522 |
| TCONS_00021202 | glutathione S-transferase TAU 12 | at1g69920 | -1.8 | 0.0 | -1.3 | C06:1..40704471:5198955-5203064 |
| TCONS_00023186 | UDP-glucuronic acid decarboxylase 2 | at3g62830 | -1.8 | -0.1 | -0.7 | C06:1..40704471:16695612-16696835 |
| TCONS_00005615 | pinus taeda anonymous locus cl2598contig1 03 genomic sequence | at5g54400 | -1.8 | -0.1 | -0.7 | C02:1..44046003:11205657-11207435 |
| TCONS_00004834 | uncharacterized protein | at5g15190 | -1.8 | -0.6 | -0.9 | C02:1..44046003:3463935-3465061 |
| TCONS_00018856 | kinase domain-containing | at1g16670 | -1.8 | -0.0 | -0.4 | C05:1..32828328:6663152-6665640 |
| TCONS_00011671 | P-loop containing nucleoside triphosphate hydrolases superfamily | at2g18193 | -1.8 | -0.8 | -1.1 | C03:1..57781463:29463527-29465253 |
| TCONS_00009735 | annexin D4-like | at2g38750 | -1.8 | -0.2 | -1.5 | C03:1..57781463:10482754-10484688 |
| TCONS_00015404 | cinnamate-4-hydroxylase | at2g30490 | -1.8 | -0.2 | -0.8 | C04:1..40895475:17675073-17677788 |
| TCONS_00009770 | Nitrate and chloride transporter | at2g39210 | -1.8 | 0.2 | -0.7 | C03:1..57781463:10775274-10776960 |
| TCONS_00036806 | glutamate decarboxylase 1-like | at5g17330 | -1.8 | -1.1 | -2.3 | C09:1..40126856:32266976-32276595 |
| TCONS_00004017 | myb domain 78 | at3g06490 | -1.8 | -0.0 | -0.5 | C01:1..38761720:38748205-38750895 |
| TCONS_00010910 | 70 kDa heat shock | at3g12580 | -1.8 | -0.4 | -1.1 | C03:1..57781463:20733731-20737094 |
| TCONS_00004031 | ATAF2 | at5g08790 | -1.8 | -0.0 | -1.0 | C02:1..44046003:2486619-2492886 |
| TCONS_00034722 | F-box LRR-repeat 3-like | at5g27920 | -1.8 | 0.0 | -0.9 | C09:1..40126856:3270650-3310922 |
| TCONS_00022243 | MATE efflux family | at1g71140 | -1.8 | -0.3 | -1.0 | C06:1..40704471:4438819-4440741 |
| TCONS_00009153 | -NA- | -- | -1.8 | -0.4 | -0.6 | C03:1..57781463:5646764-5647868 |
| TCONS_00003593 | protein fyd | at3g12570 | -1.8 | -0.3 | -1.0 | C01:1..38761720:35947955-35953212 |
| TCONS_00020872 | S-adenosylmethionine synthetase | at3g17390 | -1.8 | 1.3 | -1.6 | C05:1..32828328:31126473-31127979 |
| TCONS_00033351 | ACC oxidase | at1g62380 | -1.8 | 0.5 | -1.3 | C08:1..41516064:38823185-38824442 |
| TCONS_00037394 | tyrosine phosphatase | at5g10695 | -1.8 | -0.2 | -1.1 | C09:1..40126856:36796090-36797647 |
| TCONS_00024924 | NEP1-interacting 2 | at2g17730 | -1.8 | -0.4 | -0.3 | C07:1..48346208:11822007-11828894 |
| TCONS_00035447 | L-type lectin-domain containing receptor kinase -like | at4g04960 | -1.8 | -0.2 | -0.7 | C09:1..40126856:11666693-11669081 |
| TCONS_00018739 | ATPDR7 PDR7 | at1g15210 | -1.8 | 0.3 | -0.9 | C05:1..32828328:5716656-5747041 |
| TCONS_00032775 | -NA- | at2g21510 | -1.8 | -0.1 | -1.0 | C08:1..41516064:35708413-35710037 |
| TCONS_00015659 | hypothetical protein EUTSA v10017803mg | at2g34610 | -1.8 | -0.4 | -0.2 | C04:1..40895475:21171374-21172995 |
| TCONS_00027505 | proline dehydrogenase | at3g30775 | -1.8 | 0.0 | -0.1 | C07:1..48346208:34472655-34474206 |
| TCONS_00012827 | phosphatase 2C | at2g30020 | -1.8 | -0.4 | -1.3 | C03:1..57781463:49699922-49701796 |
| TCONS_00009841 | auxin transporter 2-like | at2g38120 | -1.8 | 0.1 | -0.6 | C03:1..57781463:11090625-11092597 |
| TCONS_00026913 | transcription factor jumonji (jmj) family zinc finger (C5HC2 type) family | at5g46910 | -1.8 | -0.3 | -0.8 | C07:1..48346208:28863136-28865294 |
| TCONS_00013668 | -NA- | at2g27080 | -1.8 | -0.0 | -0.9 | C04:1..40895475:30287290-30294217 |
| TCONS_00010237 | heat stress transcription factor A-2 | at2g26150 | -1.8 | -0.0 | -0.1 | C03:1..57781463:14355844-14357047 |
| TCONS_00024400 | pathogenesis-related 5-like | at1g75050 | -1.8 | -0.0 | -0.6 | C06:1..40704471:32439676-32441155 |
| TCONS_00013979 | VQ motif-containing | at2g41180 | -1.8 | -0.2 | -1.3 | C04:1..40895475:342072-343060 |
| TCONS_00012105 | plant invertase pectin methylesterase inhibitor superfamily | at3g47380 | -1.8 | -0.1 | -1.1 | C03:1..57781463:39247243-39248260 |
| TCONS_00029220 | BTB POZ and TAZ domain-containing 4-like | at4g37610 | -1.8 | -0.3 | -0.3 | C07:1..48346208:47718929-47721247 |
| TCONS_00037030 | WRKY transcription factor 6-like | at1g62300 | -1.8 | 0.1 | -0.1 | C09:1..40126856:34030683-34033848 |
| TCONS_00035533 | 1-aminocyclopropane-1-carboxylate synthase | at4g11280 | -1.8 | 0.4 | -1.5 | C09:1..40126856:12934762-12936760 |
| TCONS_00017211 | dehydration-responsive element-binding 2C | at2g40340 | -1.8 | -0.0 | -0.2 | C04:1..40895475:38609124-38611667 |
| TCONS_00006866 | late embryogenesis abundant domain-containing protein | at2g03740 | -1.8 | -0.3 | -1.0 | C02:1..44046003:36631724-36632425 |
| TCONS_00024070 | GATA transcription factor 8 | at3g54810 | -1.8 | -0.4 | -1.0 | C06:1..40704471:29644586-29646909 |
| TCONS_00014969 | -NA- | at1g67850 | -1.8 | 0.0 | -0.8 | C04:1..40895475:10309268-10311121 |
| TCONS_00030993 | -NA- | at1g16950 | -1.8 | -0.3 | -1.1 | C08:1..41516064:19888934-19889465 |
| TCONS_00000515 | -NA- | -- | -1.8 | -0.7 | -1.5 | C01:1..38761720:564661-564971 |
| TCONS_00026313 | gibberellin 2-oxidase | at1g30040 | -1.8 | 0.2 | -0.9 | C07:1..48346208:18129553-18130431 |
| TCONS_00025344 | transcription factor | at4g33450 | -1.8 | -0.7 | -0.1 | C07:1..48346208:46579735-46585464 |
| TCONS_00029241 | adenosine-5 -phosphosulfate-kinase | -- | -1.8 | 0.3 | -1.5 | C07:1..48346208:47912156-47912757 |
| TCONS_00015840 | GATA transcription factor 8 | at3g54810 | -1.8 | -0.4 | -1.3 | C04:1..40895475:23963440-23964910 |
| TCONS_00011397 | disease resistance | at1g71390 | -1.8 | -0.2 | -0.7 | C03:1..57781463:26366346-26368835 |
| TCONS_00031016 | lipoxygenase | at1g17420 | -1.8 | -0.7 | -1.1 | C08:1..41516064:20152382-20153337 |
| TCONS_00003816 | metallothionein 2A | p69164|mt2_brara | -1.8 | -0.0 | 0.1 | C01:1..38761720:37532648-37535732 |
| TCONS_00017116 | ubiquitin-conjugating enzyme RWD | at2g38830 | -1.8 | -0.0 | -0.4 | C04:1..40895475:37945432-37948429 |
| TCONS_00032551 | hypothetical protein CARUB v10022942mg | at2g25460 | -1.8 | 0.1 | -0.0 | C08:1..41516064:33916625-33921035 |
| TCONS_00002996 | vq motif-containing protein | at3g22160 | -1.7 | -0.1 | -0.9 | C01:1..38761720:28340075-28341303 |
| TCONS_00018731 | glutamine amidotransferase | at1g15040 | -1.7 | -0.1 | -0.5 | C05:1..32828328:5668483-5670011 |
| TCONS_00012645 | Mediator of RNA polymerase II transcription subunit | at4g39840 | -1.7 | 0.0 | -0.7 | C03:1..57781463:46585636-46587601 |
| TCONS_00022244 | TRANSPARENT TESTA 12 | at1g71140 | -1.7 | -0.4 | -1.0 | C06:1..40704471:4440875-4441223 |
| TCONS_00008075 | elicitor-activated gene 3 | at4g37990 | -1.7 | -0.1 | -0.9 | C03:1..57781463:46880372-46906655 |
| TCONS_00000644 | beta-galactosidase like protein | at4g36360 | -1.7 | -0.2 | -1.0 | C01:1..38761720:1332712-1335644 |
| TCONS_00014106 | pirin -like | at2g43120 | -1.7 | -0.0 | -0.3 | C04:1..40895475:1400869-1402765 |
| TCONS_00011313 | adenosylhomocysteinase | at3g23810 | -1.7 | 0.5 | -1.2 | C03:1..57781463:25414810-25415593 |
| TCONS_00030788 | nitrate transporter | at1g12110 | -1.7 | -0.7 | -0.8 | C08:1..41516064:18424242-18428279 |
| TCONS_00031515 | R2R3-MYB transcription factor | at3g50060 | -1.7 | -0.4 | -0.6 | C08:1..41516064:26882392-26883551 |
| TCONS_00036958 | ---NA--- | at5g15490 | -1.7 | -0.2 | -1.1 | C09:1..40126856:33390095-33392410 |
| TCONS_00005506 | heat shock 81-2 | at5g56030 | -1.7 | -0.3 | -1.4 | C02:1..44046003:10111612-10114630 |
| TCONS_00009267 | probable serine threonine- kinase WNK11-like | at5g55560 | -1.7 | 0.0 | -0.7 | C03:1..57781463:6347366-6349095 |
| TCONS_00000727 | sec7-like guanine nucleotide exchange family protein | at4g35380 | -1.7 | -0.0 | -0.9 | C01:1..38761720:1856821-1858458 |
| TCONS_00009544 | class IV chitinase | at2g43590 | -1.7 | 0.1 | -1.2 | C03:1..57781463:8645097-8645555 |
| TCONS_00015797 | translation initiation factor IF6 | at3g55620 | -1.7 | -0.1 | -0.8 | C04:1..40895475:23041044-23043455 |
| TCONS_00001522 | major intrinsic | at4g18910 | -1.7 | 0.2 | -0.3 | C01:1..38761720:6846387-6847874 |
| TCONS_00001034 | dehydration-responsive element-binding protein 3-like | at4g32800 | -1.7 | -0.9 | -1.8 | C01:1..38761720:3113738-3114908 |
| TCONS_00009286 | Rab3 GTPase-activating catalytic subunit isoform 1 | at5g55060 | -1.7 | -0.1 | -1.1 | C03:1..57781463:6471089-6471968 |
| TCONS_00028688 | 1-amino-cyclopropane-1-carboxylate synthase 7 | at4g26200 | -1.7 | 0.0 | -0.2 | C07:1..48346208:44139256-44141195 |
| TCONS_00023484 | -NA- | -- | -1.7 | 0.1 | -0.5 | C06:1..40704471:20310521-20310893 |
| TCONS_00035294 | lipid phosphate phosphatase 1 | at2g01180 | -1.7 | -0.1 | -0.8 | C09:1..40126856:9233981-9235922 |
| TCONS_00016009 | unnamed protein product | -- | -1.7 | -0.2 | -1.6 | C04:1..40895475:26504100-26505782 |
| TCONS_00026208 | indole-3-acetic acid-amido synthetase | at1g28130 | -1.7 | -0.2 | -1.1 | C07:1..48346208:16556599-16558991 |
| TCONS_00034544 | transferring glycosyl | at3g28340 | -1.7 | -0.1 | -1.3 | C09:1..40126856:2112743-2114093 |
| TCONS_00017729 | NPK1-related MAP kinase | at1g05100 | -1.7 | 0.3 | -0.0 | C05:1..32828328:1489810-1541127 |
| TCONS_00022015 | ---NA--- | at1g74360 | -1.7 | -0.2 | -0.9 | C06:1..40704471:3217961-3223312 |
| TCONS_00017197 | pollen Ole e 1 allergen and extensin family | at2g40113 | -1.7 | -0.3 | -0.7 | C04:1..40895475:38449809-38450855 |
| TCONS_00022411 | ethylene responsive element binding factor 2 (ATERF2) | at4g17500 | -1.7 | -0.4 | -0.9 | C06:1..40704471:6230410-6231443 |
| TCONS_00008315 | partial | at5g06320 | -1.7 | -0.2 | -0.6 | C03:1..57781463:1072815-1073975 |
| TCONS_00010833 | UDP-arabinopyranose mutase 1 | at5g15650 | -1.7 | 0.0 | -0.6 | C03:1..57781463:19984605-19987340 |
| TCONS_00009601 | hypothetical protein CARUB v10023598mg | at2g36470 | -1.7 | -0.5 | -0.8 | C03:1..57781463:9192897-9194478 |
| TCONS_00034460 | Indole-3-acetic acid-amido synthetase | at4g03400 | -1.7 | -0.7 | -2.0 | C09:1..40126856:1595973-1597616 |
| TCONS_00003394 | -NA- | at5g42330 | -1.7 | 0.1 | -0.5 | C01:1..38761720:33796630-33797707 |
| TCONS_00009012 | glycerophosphodiester phosphodiesterase 1 | at3g02040 | -1.7 | 0.1 | -0.4 | C03:1..57781463:4669885-4670233 |
| TCONS_00003516 | arabinogalactan protein 12 | -- | -1.7 | 0.1 | -0.8 | C01:1..38761720:35366960-35367728 |
| TCONS_00027235 | cytochrome P450 | at3g26170 | -1.7 | 0.1 | -0.8 | C07:1..48346208:32073560-32075458 |
| TCONS_00008973 | heat shock family | at5g20970 | -1.7 | -0.5 | -1.6 | C03:1..57781463:4477611-4478885 |
| TCONS_00033513 | sugar transporter ERD6-like 2 | at1g08930 | -1.7 | 0.0 | -0.8 | C08:1..41516064:39839855-39840652 |
| TCONS_00018480 | phosphoglycerate bisphosphoglycerate mutase family | at1g08940 | -1.7 | -0.5 | -1.0 | C05:1..32828328:3282227-3282834 |
| TCONS_00027285 | hydroxyproline-rich glyco family | at3g26910 | -1.7 | -0.3 | -0.9 | C07:1..48346208:32528687-32530292 |
| TCONS_00032040 | calcium-dependent kinase | at3g57530 | -1.7 | -0.3 | -0.7 | C08:1..41516064:30945900-30949626 |
| TCONS_00009105 | polynucleotidyl ribonuclease H-like superfamily | -- | -1.7 | -0.4 | -0.9 | C03:1..57781463:5308605-5309947 |
| TCONS_00009235 | pollen-specific SF21-like | at5g56750 | -1.7 | -0.0 | -0.8 | C03:1..57781463:6134770-6136409 |
| TCONS_00001649 | myb-related M4 | at4g21440 | -1.7 | -0.3 | -0.4 | C01:1..38761720:7931733-7933307 |
| TCONS_00024504 | -NA- | at3g24255 | -1.7 | -0.1 | -0.4 | C06:1..40704471:33585769-33587541 |
| TCONS_00022359 | Ferredoxin- chloroplast | at2g27510 | -1.7 | -0.5 | -0.7 | C06:1..40704471:5732980-5734435 |
| TCONS_00005445 | xyloglucan endotransglycosylase hydrolase | at5g57560 | -1.7 | -0.7 | -1.1 | C02:1..44046003:9197431-9198539 |
| TCONS_00030848 | Regulator of Vps4 activity in the MVB pathway | at1g13340 | -1.7 | -0.3 | -0.8 | C08:1..41516064:18978399-18982261 |
| TCONS_00010301 | high-affinity nitrate transporter | at5g50200 | -1.7 | -0.0 | -0.0 | C03:1..57781463:14985550-14986936 |
| TCONS_00023696 | leucine-rich repeat (LRR) family | at1g33590 | -1.7 | -0.0 | -0.7 | C06:1..40704471:24037503-24039347 |
| TCONS_00022307 | -NA- | at1g70140 | -1.7 | -0.2 | -0.9 | C06:1..40704471:5099386-5101805 |
| TCONS_00026397 | -NA- | -- | -1.7 | -0.4 | -1.4 | C07:1..48346208:21172778-21173698 |
| TCONS_00001753 | trehalose-6-phosphate phosphatase ( ) | at4g22590 | -1.7 | -0.2 | -0.5 | C01:1..38761720:8910981-8913264 |
| TCONS_00021783 | ---NA--- | -- | -1.7 | 0.5 | -0.9 | C06:1..40704471:1409350-1410764 |
| TCONS_00001408 | ethylene responsive element binding factor 2 | at4g17500 | -1.7 | 0.0 | -0.9 | C01:1..38761720:5866018-5867098 |
| TCONS_00033467 | glutamyl-tRNA reductase | at1g09940 | -1.7 | -0.0 | -0.8 | C08:1..41516064:39541744-39542292 |
| TCONS_00011312 | adenosylhomocysteinase | at3g23810 | -1.7 | 1.1 | -0.6 | C03:1..57781463:25412835-25414262 |
| TCONS_00034810 | endonuclease or glycosyl hydrolase with C2H2-type zinc finger domain | at5g61190 | -1.7 | -0.6 | -0.8 | C09:1..40126856:3917871-3921716 |
| TCONS_00003671 | glycerol-3-phosphate acyltransferase 5-like | at3g11430 | -1.7 | 0.3 | -0.1 | C01:1..38761720:36563583-36564844 |
| TCONS_00037766 | anthranilate synthase component I-1 | at5g05730 | -1.6 | 1.4 | -0.5 | C09:1..40126856:39024323-39026115 |
| TCONS_00027451 | calcium-binding EF hand family | at3g29000 | -1.6 | -0.0 | -0.7 | C07:1..48346208:33961290-33962224 |
| TCONS_00014195 | methylenetetrahydrofolate reductase | at2g44160 | -1.6 | -0.0 | -2.0 | C04:1..40895475:1863320-1864211 |
| TCONS_00014226 | RING U-box domain-containing | at2g44578 | -1.6 | -0.7 | -2.1 | C04:1..40895475:2374442-2375311 |
| TCONS_00013253 | heat shock 90 | at5g56030 | -1.6 | -0.2 | -0.6 | C03:1..57781463:55254697-55255970 |
| TCONS_00006493 | probable lrr receptor-like serine threonine-protein kinase at3g47570-like | at3g47110 | -1.6 | -0.3 | -0.4 | C02:1..44046003:30171807-30175291 |
| TCONS_00009796 | uncharacterized protein | at2g39650 | -1.6 | -0.1 | -0.9 | C03:1..57781463:10884001-10886438 |
| TCONS_00016971 | late embryogenesis abundant | at2g36640 | -1.6 | -0.8 | 0.0 | C04:1..40895475:36931586-36934644 |
| TCONS_00033201 | probable fucosyltransferase 4 | at1g14080 | -1.6 | 0.0 | -1.0 | C08:1..41516064:37968744-37970795 |
| TCONS_00028324 | shaggy-related kinase eta-like | at4g18710 | -1.6 | 0.3 | -0.7 | C07:1..48346208:41155084-41155965 |
| TCONS_00024323 | O-methyltransferase family 2 | at1g21100 | -1.6 | 1.0 | -1.3 | C06:1..40704471:31670735-31671815 |
| TCONS_00027674 | cytochrome P450 | -- | -1.6 | 0.3 | -0.6 | C07:1..48346208:35720714-35720817 |
| TCONS_00028388 | ethylene-responsive regulated nuclear | -- | -1.6 | -0.1 | -1.4 | C07:1..48346208:41880355-41880742 |
| TCONS_00009543 | class IV chitinase | at2g43590 | -1.6 | 0.4 | -0.9 | C03:1..57781463:8643378-8644243 |
| TCONS_00009931 | Calmodulin | at2g41110 | -1.6 | -0.2 | -1.3 | C03:1..57781463:11838306-11839507 |
| TCONS_00034044 | S-type anion channel SLAH3-like | at5g24030 | -1.6 | 0.0 | -0.6 | C09:1..40126856:4299850-4305987 |
| TCONS_00022346 | transcription factor FER-LIKE IRON DEFICIENCY-INDUCED TRANSCRIPTION FACTOR-like | at2g28160 | -1.6 | -0.6 | -0.8 | C06:1..40704471:5539005-5540824 |
| TCONS_00026511 | probable inactive poly [ADP-ribose] polymerase SRO2 | at1g23550 | -1.6 | -0.5 | -1.1 | C07:1..48346208:23336942-23338633 |
| TCONS_00029156 | ferulate-5-hydroxylase | at4g36220 | -1.6 | -0.2 | -1.2 | C07:1..48346208:47280798-47282185 |
| TCONS_00017917 | 1-aminocyclopropane-1-carboxylate synthase 2 | at1g01480 | -1.6 | 0.3 | -0.2 | C05:1..32828328:171344-174738 |
| TCONS_00022523 | leucine-rich repeat (LRR) family | at1g33590 | -1.6 | 0.0 | -0.6 | C06:1..40704471:7510356-7512454 |
| TCONS_00027116 | sucrose transporter | at2g02860 | -1.6 | -0.4 | -0.7 | C07:1..48346208:30660123-30661696 |
| TCONS_00001524 | integrin-linked protein kinase family protein | at4g18950 | -1.6 | -0.1 | -0.6 | C01:1..38761720:6881103-6884011 |
| TCONS_00000487 | 3-deoxy-D-arabino-heptulosonate 7-phosphate synthase 1 | at4g39980 | -1.6 | -0.0 | -1.0 | C01:1..38761720:421862-424109 |
| TCONS_00014348 | basic helix-loop-helix family | at2g46510 | -1.6 | -0.5 | -0.9 | C04:1..40895475:3178663-3181063 |
| TCONS_00017475 | BON association 1 | at2g45760 | -1.6 | 0.1 | -0.9 | C04:1..40895475:40453132-40453957 |
| TCONS_00020832 | AP2 B3-like transcriptional factor family | at3g11580 | -1.6 | -0.2 | -0.8 | C05:1..32828328:30734128-30737285 |
| TCONS_00024770 | Type I inositol-1,4,5-trisphosphate 5-phosphatase 11 | at1g47510 | -1.6 | 0.0 | -0.7 | C06:1..40704471:39306439-39309110 |
| TCONS_00022178 | TIFY 11B | at1g72450 | -1.6 | -0.7 | -1.9 | C06:1..40704471:4163378-4165555 |
| TCONS_00012682 | DNA binding | at4g37900 | -1.6 | -0.1 | -0.7 | C03:1..57781463:46997636-46999990 |
| TCONS_00014487 | plasma membrane intrinsic 2a | at2g37180 | -1.6 | 0.4 | -0.6 | C04:1..40895475:4772583-4773217 |
| TCONS_00008464 | cellulose synthase catalytic subunit | at5g09870 | -1.6 | -0.4 | -1.3 | C03:1..57781463:1819349-1820354 |
| TCONS_00006771 | phytosulfokine receptor 1-like | at2g02220 | -1.6 | -0.0 | -0.3 | C02:1..44046003:34979353-34982678 |
| TCONS_00022316 | leucine-rich repeat kinase family | at1g69990 | -1.6 | 0.0 | -0.4 | C06:1..40704471:5165667-5166982 |
| TCONS_00030995 | secologanin synthase-like | at1g17060 | -1.6 | -0.5 | -0.9 | C08:1..41516064:19904625-19906127 |
| TCONS_00012390 | Chromatin assembly factor 1 subunit | at1g28190 | -1.6 | -0.4 | -1.4 | C03:1..57781463:43921522-43922743 |
| TCONS_00024572 | -NA- | at1g70780 | -1.6 | 0.2 | -0.7 | C06:1..40704471:34675968-34677537 |
| TCONS_00034874 | calcium-dependent lipid-binding domain-containing | at5g23950 | -1.6 | -0.2 | -1.5 | C09:1..40126856:4280592-4281958 |
| TCONS_00031133 | -NA- | at1g19270 | -1.6 | -0.2 | -0.8 | C08:1..41516064:21223878-21225367 |
| TCONS_00032821 | disease resistance response 206-like | at2g21100 | -1.6 | 0.0 | -0.4 | C08:1..41516064:35890396-35891237 |
| TCONS_00022360 | glucan endo-1,3-beta-glucosidase | at2g27500 | -1.6 | -0.1 | -0.8 | C06:1..40704471:5743876-5746578 |
| TCONS_00037869 | heat stress transcription factor A-3-like | at5g03720 | -1.6 | -0.5 | -0.8 | C09:1..40126856:39592081-39593214 |
| TCONS_00024748 | triacylglycerol lipase-like 1 | at1g45201 | -1.6 | -0.0 | -1.5 | C06:1..40704471:38573260-38596156 |
| TCONS_00025994 | AF361581 1 AT4g35320 F23E12 120 | at2g17300 | -1.6 | -0.5 | -0.8 | C07:1..48346208:12676382-12677386 |
| TCONS_00007066 | ninja-family protein afp3 | at3g29575 | -1.6 | -0.2 | -0.6 | C02:1..44046003:39739486-39741610 |
| TCONS_00025024 | 6-phosphogluconolactonase | at5g24420 | -1.6 | -0.2 | -0.7 | C07:1..48346208:36144500-36170141 |
| TCONS_00035986 | integral membrane transporter family | at5g54860 | -1.6 | -0.2 | -1.0 | C09:1..40126856:23363237-23365076 |
| TCONS_00036677 | inosine-uridine preferring nucleoside hydrolase family | at5g18860 | -1.6 | -0.4 | -1.2 | C09:1..40126856:31217814-31218815 |
| TCONS_00027114 | sucrose transport SUC3 | at2g02860 | -1.6 | 0.1 | -0.7 | C07:1..48346208:30656713-30658612 |
| TCONS_00030730 | receptor kinase HAIKU2-like | at1g09970 | -1.6 | -0.3 | -0.6 | C08:1..41516064:17695069-17698491 |
| TCONS_00008644 | 2-aminoethanethiol dioxygenase-like | at5g15120 | -1.6 | -0.4 | -0.3 | C03:1..57781463:2665570-2666258 |
| TCONS_00002513 | probable xyloglucan endotransglucosylase hydrolase protein 30-like | at1g32170 | -1.6 | -0.0 | -0.3 | C01:1..38761720:19269882-19270962 |
| TCONS_00015256 | calcium-transporting ATPase plasma membrane-type-like | at3g63380 | -1.6 | -0.4 | -0.8 | C04:1..40895475:15203951-15208810 |
| TCONS_00017121 | -NA- | -- | -1.6 | 0.1 | -0.0 | C04:1..40895475:37978609-37979636 |
| TCONS_00024498 | receptor kinase-like | at1g73080 | -1.5 | -0.6 | -1.1 | C06:1..40704471:33554638-33558790 |
| TCONS_00009706 | PRA1 family B4-like | at2g38360 | -1.5 | 0.0 | -0.6 | C03:1..57781463:10291304-10294537 |
| TCONS_00016537 | leucine-rich repeat disease resistance | at2g33020 | -1.5 | -0.1 | -1.7 | C04:1..40895475:32370943-32373307 |
| TCONS_00027403 | zinc finger AN1 domain-containing stress-associated 12-like | at3g28210 | -1.5 | -0.2 | -1.0 | C07:1..48346208:33487606-33488800 |
| TCONS_00008929 | tetratricopeptide repeat domain-containing | at5g20190 | -1.5 | 0.0 | -1.0 | C03:1..57781463:4230417-4232596 |
| TCONS_00004360 | trihelix transcription factor gt-3b-like | at5g01380 | -1.5 | -0.4 | -0.7 | C02:1..44046003:119504-121509 |
| TCONS_00001291 | nuclease harbi1-like | at4g29780 | -1.5 | -0.6 | -1.3 | C01:1..38761720:4768503-4771076 |
| TCONS_00000526 | basic leucine-zipper 6 | at4g37730 | -1.5 | -0.1 | -1.0 | C01:1..38761720:702225-703458 |
| TCONS_00006381 | -NA- | -- | -1.5 | -0.6 | -1.1 | C02:1..44046003:26166640-26167807 |
| TCONS_00008687 | 3-oxo-5-alpha-steroid 4-dehydrogenase family | at5g16010 | -1.5 | -0.6 | -1.5 | C03:1..57781463:2979622-2980578 |
| TCONS_00014599 | alanine:glyoxylate aminotransferase 3 | at2g38400 | -1.5 | 0.1 | -0.0 | C04:1..40895475:5990333-5991646 |
| TCONS_00019646 | UDP-glucosyl transferase 74b1 | at1g24100 | -1.5 | -0.1 | -0.9 | C05:1..32828328:17867510-17869223 |
| TCONS_00015488 | mitogen-activated kinase kinase kinase A-like | at2g32510 | -1.5 | 0.3 | 0.4 | C04:1..40895475:19118168-19120460 |
| TCONS_00026910 | -NA- | -- | -1.5 | -0.1 | -0.7 | C07:1..48346208:28860254-28861390 |
| TCONS_00022570 | IDA ARATH ame: Full= IDA ame: Full= INFLORESCENCE DEFICIENT IN ABSCISSION Flags: Precursor | at1g68765 | -1.5 | -0.1 | -0.9 | C06:1..40704471:8000367-8001395 |
| TCONS_00023257 | BTB and TAZ domain 1 | at5g63160 | -1.5 | -0.1 | -0.1 | C06:1..40704471:17483033-17485209 |
| TCONS_00009468 | cinnamate-4-hydroxylase | at2g30490 | -1.5 | -0.6 | -1.3 | C03:1..57781463:8130294-8141386 |
| TCONS_00005471 | calcium-transporting ATPase 8 | at4g29900 | -1.5 | -0.4 | -1.7 | C02:1..44046003:9715460-9717553 |
| TCONS_00033237 | -NA- | at1g13470 | -1.5 | -0.3 | -0.9 | C08:1..41516064:38210401-38212045 |
| TCONS_00002291 | ethylene-responsive transcription factor erf054-like | at4g28140 | -1.5 | -0.6 | -1.2 | C01:1..38761720:14236197-14237362 |
| TCONS_00017449 | leucine-rich repeat transmembrane kinase | at2g45340 | -1.5 | 0.0 | -1.6 | C04:1..40895475:40315832-40318014 |
| TCONS_00029801 | probable phosphatase 2C 4-like isoform 2 | at1g07630 | -1.5 | -0.5 | -1.0 | C08:1..41516064:1435368-1438117 |
| TCONS_00016221 | elicitor like | at4g14420 | -1.5 | 0.0 | -0.6 | C04:1..40895475:28696380-28698107 |
| TCONS_00010366 | -NA- | -- | -1.5 | -0.3 | -0.4 | C03:1..57781463:15643574-15644720 |
| TCONS_00018520 | ACT domain repeat 8 | at1g12420 | -1.5 | -0.7 | -0.7 | C05:1..32828328:3558481-3560079 |
| TCONS_00010236 | heat shock transcription factor A2 | at2g26150 | -1.5 | -0.4 | -0.2 | C03:1..57781463:14355216-14355706 |
| TCONS_00031722 | L-type lectin-domain containing receptor kinase | at3g53810 | -1.5 | -0.3 | -0.7 | C08:1..41516064:28648629-28651686 |
| TCONS_00032690 | rhamnogalacturonate lyase | at2g22620 | -1.5 | 0.3 | -0.4 | C08:1..41516064:35187264-35188554 |
| TCONS_00002259 | at5g54141 $at5g54141 | at4g27657 | -1.5 | -0.9 | -1.6 | C01:1..38761720:13783723-13784280 |
| TCONS_00005870 | protein tify 11b | at1g72450 | -1.5 | -0.4 | -1.1 | C02:1..44046003:15839160-15841291 |
| TCONS_00036725 | hypothetical protein EUTSA v10014852mg | at5g18310 | -1.5 | -0.4 | -1.0 | C09:1..40126856:31570162-31572155 |
| TCONS_00010522 | -NA- | at4g04650 | -1.5 | 0.0 | -0.6 | C03:1..57781463:17412916-17414100 |
| TCONS_00018561 | stigma-specific Stig1 family | at1g11925 | -1.5 | 0.3 | -0.6 | C05:1..32828328:3929967-3930793 |
| TCONS_00029211 | -NA- | -- | -1.5 | -0.4 | -1.0 | C07:1..48346208:47659574-47660899 |
| TCONS_00031748 | S-adenosyl-L-methionine-dependent methyltransferases superfamily | at3g54150 | -1.5 | 0.1 | -0.8 | C08:1..41516064:28852640-28853744 |
| TCONS_00026016 | glycerol-3-phosphate transporter 5-like | at2g13100 | -1.5 | -0.1 | -0.8 | C07:1..48346208:13018338-13020272 |
| TCONS_00012338 | anthranilate synthase beta subunit | at1g25220 | -1.5 | 0.3 | -0.8 | C03:1..57781463:43538474-43540754 |
| TCONS_00016712 | aceous RNase P chloroplastic mitochondrial-like | at2g32230 | -1.5 | -0.4 | -1.0 | C04:1..40895475:34583664-34584814 |
| TCONS_00005891 | TIR-NBS class of disease resistance | at1g72890 | -1.5 | -0.1 | -0.8 | C02:1..44046003:16257334-16259701 |
| TCONS_00007985 | basic endochitinase | q09023|chi2_brana | -1.5 | -0.3 | -0.6 | C03:1..57781463:20709965-20722652 |
| TCONS_00009672 | heme-binding chloroplastic-like | at2g37970 | -1.5 | -0.3 | -0.7 | C03:1..57781463:9878426-9880602 |
| TCONS_00020665 | arabinogalactan 12 | -- | -1.5 | -0.0 | -0.7 | C05:1..32828328:29499451-29501060 |
| TCONS_00015689 | disease resistance | at2g34930 | -1.5 | -0.3 | -1.2 | C04:1..40895475:21467018-21467499 |
| TCONS_00022365 | uncharacterized protein | -- | -1.5 | 0.0 | -0.8 | C06:1..40704471:5788687-5789510 |
| TCONS_00012439 | WRKY transcription factor | at1g29280 | -1.5 | -0.3 | -1.1 | C03:1..57781463:44662892-44664575 |
| TCONS_00019722 | UDP-glycosyltransferase 82A1-like | at3g22250 | -1.5 | 0.0 | -0.8 | C05:1..32828328:18920816-18924533 |
| TCONS_00016817 | ethylene-responsive transcription factor ERF112 | at2g33710 | -1.5 | -0.0 | -0.5 | C04:1..40895475:35409187-35409966 |
| TCONS_00018481 | phosphoglycerate bisphosphoglycerate mutase family | at1g08940 | -1.5 | -0.0 | -0.9 | C05:1..32828328:3283655-3284079 |
| TCONS_00035442 | -NA- | at4g05010 | -1.5 | -0.1 | -0.9 | C09:1..40126856:11545152-11546324 |
| TCONS_00004555 | cytokinin riboside 5 -monophosphate phosphoribohydrolase log7-like | at5g06300 | -1.5 | 0.0 | -1.0 | C02:1..44046003:1992190-1992990 |
| TCONS_00017697 | syntaxin 121 family | at3g11820 | -1.5 | -0.2 | -0.9 | C05:1..32828328:30554417-30558624 |
| TCONS_00003169 | polyol transporter 5-like | at3g18830 | -1.5 | -0.1 | -1.2 | C01:1..38761720:31139430-31143015 |
| TCONS_00008441 | NAC domain-containing 102 | at5g08790 | -1.5 | -0.2 | -0.7 | C03:1..57781463:1704636-1706757 |
| TCONS_00028327 | BES1 BZR1 3 | at4g18890 | -1.5 | -0.0 | -0.6 | C07:1..48346208:41219809-41235603 |
| TCONS_00010401 | esterase lipase HI 0193-like | at4g10030 | -1.5 | -0.0 | -1.2 | C03:1..57781463:16033161-16033555 |
| TCONS_00000706 | serine threonine-protein kinase cx32 | at4g35600 | -1.5 | -0.4 | -0.9 | C01:1..38761720:1781784-1782790 |
| TCONS_00022146 | hydroxyproline-rich glyco family | at1g72790 | -1.5 | -0.1 | -1.1 | C06:1..40704471:4025437-4027570 |
| TCONS_00000703 | senescence-associated protein din1 | p27626|din1_rapsa | -1.5 | 0.2 | 0.5 | C01:1..38761720:1740175-1742421 |
| TCONS_00002948 | calcium-transporting ATPase plasma membrane-type-like | at3g22910 | -1.5 | -0.5 | -0.7 | C01:1..38761720:27565131-27569386 |
| TCONS_00017352 | -NA- | -- | -1.5 | 0.1 | -0.3 | C04:1..40895475:39677426-39678265 |
| TCONS_00030666 | At1g09070 F7G19 6 | at1g09070 | -1.5 | -0.1 | -1.1 | C08:1..41516064:17221341-17222465 |
| TCONS_00020601 | chaperone -domain containing | at3g14200 | -1.5 | -0.3 | -0.5 | C05:1..32828328:28993485-28994746 |
| TCONS_00029155 | ferulate-5-hydroxylase | at4g36220 | -1.5 | 0.0 | -1.0 | C07:1..48346208:47279800-47280743 |
| TCONS_00033731 | 3-ketoacyl- synthase 1 | at1g01120 | -1.5 | -1.3 | -1.7 | C08:1..41516064:41177474-41179315 |
| TCONS_00015291 | zinc finger | at2g28710 | -1.5 | 0.2 | -0.7 | C04:1..40895475:15806345-15807156 |
| TCONS_00006869 | cytosolic sulfotransferase 12-like | at2g03760 | -1.5 | -0.4 | -1.1 | C02:1..44046003:36642613-36644552 |
| TCONS_00005470 | calcium-transporting ATPase 8 | at5g57110 | -1.5 | -0.6 | -1.5 | C02:1..44046003:9713563-9715317 |
| TCONS_00031156 | opc-8:0 ligase1 | at1g20510 | -1.5 | -0.3 | -1.1 | C08:1..41516064:21566039-21581812 |
| TCONS_00033826 | AAA-ATPase 1 | at3g28580 | -1.5 | -0.1 | -0.7 | C09:1..40126856:2184797-2191138 |
| TCONS_00033354 | stigma-specific Stig1 family | at1g11925 | -1.5 | 0.0 | -0.6 | C08:1..41516064:38844508-38845420 |
| TCONS_00029242 | adenosine-5 -phosphosulfate-kinase | at4g39940 | -1.5 | 0.1 | -2.0 | C07:1..48346208:47912821-47914052 |
| TCONS_00018401 | C3HC4-type RING finger-containing | at1g08050 | -1.5 | 0.1 | -0.1 | C05:1..32828328:2845843-2848064 |
| TCONS_00035702 | trehalose-phosphate phosphatase | at5g51460 | -1.5 | 0.2 | -0.1 | C09:1..40126856:17603529-17604931 |
| TCONS_00018448 | phosphoenolpyruvate carboxylase kinase 1 | at1g08650 | -1.5 | -0.1 | -0.4 | C05:1..32828328:3149126-3151142 |
| TCONS_00014968 | AF385745 1 At1g67850 F12A21 2 | -- | -1.5 | -0.5 | -0.4 | C04:1..40895475:10306550-10309116 |
| TCONS_00033701 | dehydration-INDUCED 19-5 | at1g02750 | -1.5 | -0.3 | -0.8 | C08:1..41516064:40990690-40992682 |
| TCONS_00014083 | aspartyl protease family | at2g42980 | -1.5 | 0.0 | -0.9 | C04:1..40895475:1199096-1201250 |
| TCONS_00022247 | -NA- | at1g71110 | -1.5 | -0.0 | -0.6 | C06:1..40704471:4452936-4456691 |
| TCONS_00002721 | glucose-6-phosphate phosphate translocator chloroplastic-like | at1g61800 | -1.5 | -0.0 | -0.6 | C01:1..38761720:23292163-23294774 |
| TCONS_00031043 | late embryogenesis abundant hydroxyproline-rich glyco | at1g17620 | -1.5 | -0.4 | -1.3 | C08:1..41516064:20533339-20534414 |
| TCONS_00005479 | AT5g56980 MHM17 10 | at5g56980 | -1.5 | -0.2 | -0.6 | C02:1..44046003:9799551-9802289 |
| TCONS_00016049 | auxin-responsive family | at5g35735 | -1.5 | -0.1 | -0.8 | C04:1..40895475:26873434-26876766 |
| TCONS_00022797 | Argonaute family | at1g31280 | -1.5 | -0.3 | -0.7 | C06:1..40704471:10725964-10727982 |
| TCONS_00003772 | phosphate phosphoenolpyruvate translocator | at3g10290 | -1.4 | -0.0 | -0.7 | C01:1..38761720:37279767-37281585 |
| TCONS_00012018 | GTP-binding Rab11 | at3g46830 | -1.4 | -0.1 | -1.0 | C03:1..57781463:36297318-36299478 |
| TCONS_00017098 | WRKY transcription factor 33 | at2g38470 | -1.4 | -0.0 | -1.1 | C04:1..40895475:37790388-37793626 |
| TCONS_00030085 | Serine hydroxymethyltransferase | at4g13930 | -1.4 | -0.3 | -0.8 | C08:1..41516064:6935819-6936659 |
| TCONS_00022260 | chaperone -like | at1g71000 | -1.4 | 0.0 | -0.4 | C06:1..40704471:4558866-4560789 |
| TCONS_00018063 | glutaredoxin ATGRXS13 | at1g03850 | -1.4 | -0.0 | -0.6 | C05:1..32828328:972772-973901 |
| TCONS_00008547 | IQ-domain 11 | at5g13460 | -1.4 | -0.0 | -0.8 | C03:1..57781463:2245696-2248385 |
| TCONS_00025481 | -NA- | at5g67180 | -1.4 | -0.0 | -1.4 | C07:1..48346208:1595813-1598823 |
| TCONS_00019011 | subtilase family | at1g32940 | -1.4 | 0.2 | -1.0 | C05:1..32828328:9520989-9526810 |
| TCONS_00006314 | glutathione s-transferase | at2g02930 | -1.4 | 0.1 | -0.9 | C02:1..44046003:24256420-24257704 |
| TCONS_00004793 | nadp-dependent malic enzyme-like | at5g25880 | -1.4 | 0.5 | -0.9 | C02:1..44046003:3216086-3216700 |
| TCONS_00013274 | carbonic anhydrase | at1g70410 | -1.4 | -0.0 | -0.6 | C03:1..57781463:55511764-55513689 |
| TCONS_00021874 | -NA- | at1g75860 | -1.4 | -0.1 | -0.6 | C06:1..40704471:2008571-2010500 |
| TCONS_00021420 | nitrate transporter | at1g69870 | -1.4 | -0.1 | -0.8 | C06:1..40704471:14300341-14306089 |
| TCONS_00023989 | -NA- | at2g17120 | -1.4 | 0.0 | -0.8 | C06:1..40704471:28109592-28111684 |
| TCONS_00003091 | extracellular ligand-gated ion channel | at3g20300 | -1.4 | -0.1 | -0.7 | C01:1..38761720:30105712-30108252 |
| TCONS_00018559 | ERD (early-responsive to dehydration stress) family | at1g11960 | -1.4 | -1.0 | -1.7 | C05:1..32828328:3913513-3914793 |
| TCONS_00028487 | UDP-galactose transporter 2 | at4g23010 | -1.4 | -0.0 | -0.7 | C07:1..48346208:42710040-42712861 |
| TCONS_00033706 | -NA- | at5g38700 | -1.4 | -0.3 | -0.3 | C08:1..41516064:41000021-41002215 |
| TCONS_00025948 | -methionine-s-oxide reductase | at2g17705 | -1.4 | -0.0 | -1.0 | C07:1..48346208:11858068-11858959 |
| TCONS_00011655 | Senescence dehydration-associated | at2g17840 | -1.4 | -0.4 | -1.4 | C03:1..57781463:29274568-29277236 |
| TCONS_00012669 | rhamnogalacturonate lyase | at1g65210 | -1.4 | 0.4 | -0.3 | C03:1..57781463:46863197-46864641 |
| TCONS_00000894 | inositol-tetrakisphosphate 1-kinase 3 | at4g33770 | -1.4 | -0.1 | -0.6 | C01:1..38761720:2630577-2632320 |
| TCONS_00003193 | -NA- | at3g18560 | -1.4 | -0.2 | -0.9 | C01:1..38761720:31412750-31413805 |
| TCONS_00023166 | ATP binding cassette subfamily B4 isoform 2 | at3g62150 | -1.4 | -0.5 | -1.1 | C06:1..40704471:16498132-16502650 |
| TCONS_00037175 | -NA- | at5g13320 | -1.4 | -0.0 | -0.1 | C09:1..40126856:35516717-35518367 |
| TCONS_00032224 | IQ-DOMAIN 14 | at3g59690 | -1.4 | -0.2 | -0.6 | C08:1..41516064:31863420-31867493 |
| TCONS_00031424 | UDP-glucuronate 4-epimerase 6-like | at3g23820 | -1.4 | -0.0 | -0.6 | C08:1..41516064:25194231-25196142 |
| TCONS_00030452 | cysteine ase inhibitor | at4g16500 | -1.4 | -0.2 | -0.8 | C08:1..41516064:14383400-14384841 |
| TCONS_00005458 | cytochrome P450 monooxygenase | at5g57220 | -1.4 | 0.6 | -0.2 | C02:1..44046003:9523734-9525885 |
| TCONS_00000615 | minor allergen | at4g36750 | -1.4 | -0.0 | -0.1 | C01:1..38761720:1163310-1165244 |
| TCONS_00022487 | hypothetical protein EUTSA v10019695mg | at1g69760 | -1.4 | -0.0 | -0.6 | C06:1..40704471:7160902-7162130 |
| TCONS_00018766 | hypothetical protein EUTSA v10009244mg | at1g15385 | -1.4 | -0.0 | -0.9 | C05:1..32828328:5826604-5827611 |
| TCONS_00011617 | phospholipase-like (PEARLI 4) family | at2g16900 | -1.4 | -0.6 | -0.8 | C03:1..57781463:28838000-28839402 |
| TCONS_00025755 | myo-inositol oxygenase 2 | at2g19800 | -1.4 | -0.1 | -0.1 | C07:1..48346208:7394376-7397683 |
| TCONS_00027404 | galacturonosyltransferase-like 10 | at3g28340 | -1.4 | -0.2 | -1.2 | C07:1..48346208:33491903-33493477 |
| TCONS_00017232 | AP2 ERF domain-containing transcription factor | at3g16280 | -1.4 | -0.0 | -0.3 | C04:1..40895475:38849790-38850905 |
| TCONS_00010028 | Chitinase family | q06209|chi4_brana | -1.4 | -0.1 | -0.3 | C03:1..57781463:12657043-12658886 |
| TCONS_00024484 | trypsin inhibitor | at1g73260 | -1.4 | 0.3 | -0.4 | C06:1..40704471:33443525-33444601 |
| TCONS_00009852 | -NA- | -- | -1.4 | 0.1 | -0.4 | C03:1..57781463:11129830-11131329 |
| TCONS_00021118 | myb domain 78 | at3g06490 | -1.4 | 0.0 | -0.1 | C05:1..32828328:32687085-32689611 |
| TCONS_00032826 | phospholipase (PEARLI 4) domain-containing | at2g20960 | -1.4 | 0.2 | -0.6 | C08:1..41516064:35917472-35919132 |
| TCONS_00012896 | uncharacterized protein | at4g21865 | -1.4 | -0.3 | -0.9 | C03:1..57781463:50621357-50621789 |
| TCONS_00021682 | pectin methyltransferase TSD2 | at1g78240 | -1.4 | -0.4 | -1.3 | C06:1..40704471:799436-800172 |
| TCONS_00005789 | transcription factor hb29-like | at1g69600 | -1.4 | 0.5 | -0.3 | C02:1..44046003:13722619-13724018 |
| TCONS_00018658 | F-box kelch-repeat SKIP11 | at1g14330 | -1.4 | -0.0 | -0.6 | C05:1..32828328:5034080-5035857 |
| TCONS_00022270 | -NA- | at1g70780 | -1.4 | -0.0 | -0.9 | C06:1..40704471:4695256-4697719 |
| TCONS_00004797 | -NA- | -- | -1.4 | -0.4 | -0.9 | C02:1..44046003:3227596-3228351 |
| TCONS_00024553 | somatic embryogenesis receptor kinase | at1g71830 | -1.4 | -0.1 | -0.6 | C06:1..40704471:34453806-34455071 |
| TCONS_00001979 | protein | at4g25030 | -1.4 | -0.3 | -1.1 | C01:1..38761720:11253098-11255238 |
| TCONS_00015068 | Feruloyl esterase A | at5g42930 | -1.4 | -0.3 | -0.7 | C04:1..40895475:12086622-12090100 |
| TCONS_00032042 | guanylate kinase | at2g41880 | -1.4 | -0.4 | -1.1 | C08:1..41516064:30972962-30976514 |
| TCONS_00022039 | sulfotransferase 16 | at1g74100 | -1.4 | 0.6 | -0.8 | C06:1..40704471:3322341-3323877 |
| TCONS_00014486 | plasma membrane intrinsic 2A | at2g37170 | -1.4 | 0.5 | -0.7 | C04:1..40895475:4771418-4772464 |
| TCONS_00032018 | autoinhibited calcium ATPase | at3g57330 | -1.4 | -0.2 | -1.1 | C08:1..41516064:30783707-30787248 |
| TCONS_00027541 | UPF0463 transmembrane | at5g48210 | -1.4 | 0.9 | -0.5 | C07:1..48346208:34677465-34678715 |
| TCONS_00008546 | IQ-domain 11 | at5g13460 | -1.4 | -0.5 | -0.6 | C03:1..57781463:2244764-2245373 |
| TCONS_00037191 | NAC domain IPR003441 | at5g13180 | -1.4 | -0.2 | -0.8 | C09:1..40126856:35599438-35601064 |
| TCONS_00008912 | probable receptor kinase At5g20050-like | at5g20050 | -1.4 | -0.5 | -0.4 | C03:1..57781463:4182799-4184609 |
| TCONS_00022582 | ---NA--- | at1g68620 | -1.3 | -0.2 | -0.6 | C06:1..40704471:8105974-8107655 |
| TCONS_00011749 | U-box domain-containing | at5g64660 | -1.3 | -0.0 | -0.8 | C03:1..57781463:30604002-30605610 |
| TCONS_00012410 | MACPF domain-containing NSL1-like | at1g28380 | -1.3 | -0.1 | -0.7 | C03:1..57781463:44259246-44260885 |
| TCONS_00013200 | glutamine amidotransferase class-I domain-containing | at4g30530 | -1.3 | 0.5 | -0.9 | C03:1..57781463:54529697-54530778 |
| TCONS_00011731 | flotillin 3 | at5g64870 | -1.3 | -0.1 | -0.7 | C03:1..57781463:30308492-30309781 |
| TCONS_00023268 | -NA- | at5g62960 | -1.3 | -0.3 | -0.6 | C06:1..40704471:17632538-17634419 |
| TCONS_00003443 | phospholipase-like (PEARLI 4) family | at4g35110 | -1.3 | -0.2 | -0.7 | C01:1..38761720:34393354-34394674 |
| TCONS_00022170 | hypothetical protein EUTSA v10019228mg | at1g72510 | -1.3 | -0.2 | -1.0 | C06:1..40704471:4110227-4111456 |
| TCONS_00005324 | exocyst subunit exo70 family H7 | at5g59730 | -1.3 | 0.1 | -0.4 | C02:1..44046003:7671891-7674410 |
| TCONS_00019711 | alternative oxidase | at3g22370 | -1.3 | 0.0 | -0.4 | C05:1..32828328:18855070-18857340 |
| TCONS_00030017 | cytochrome P450 monooxygenase | at4g31500 | -1.3 | -0.0 | -1.0 | C08:1..41516064:3582181-3584209 |
| TCONS_00008140 | WD-40 repeat family | at5g02430 | -1.3 | 0.0 | -0.4 | C03:1..57781463:116079-117120 |
| TCONS_00019234 | alternative oxidase 1a | at1g32350 | -1.3 | -0.0 | -0.7 | C05:1..32828328:13386674-13389001 |
| TCONS_00001591 | reticuline oxidase-like protein | at4g20830 | -1.3 | -0.2 | -1.2 | C01:1..38761720:7481899-7484447 |
| TCONS_00020397 | phospholipase D p1-like | at3g16785 | -1.3 | -0.2 | -0.3 | C05:1..32828328:27069399-27070129 |
| TCONS_00002549 | heat shock 70 | at5g02500 | -1.3 | -0.3 | -1.1 | C01:1..38761720:20415594-20418365 |
| TCONS_00030157 | B-cell receptor-associated 31 | -- | -1.3 | -0.2 | -0.6 | C08:1..41516064:8434847-8436278 |
| TCONS_00012158 | proton-dependent oligopeptide transport family | at3g47960 | -1.3 | -0.4 | -1.2 | C03:1..57781463:40018543-40020775 |
| TCONS_00027680 | probable folate-biopterin transporter 2-like | at5g25050 | -1.3 | 0.0 | -0.6 | C07:1..48346208:35763542-35766083 |
| TCONS_00006621 | jumonji domain protein | at5g46910 | -1.3 | -0.2 | -0.3 | C02:1..44046003:32418632-32421064 |
| TCONS_00003124 | magnesium transporter MRS2-3-like | at3g19640 | -1.3 | 0.3 | -0.3 | C01:1..38761720:30473655-30475345 |
| TCONS_00036143 | E3 ubiquitin- ligase XBAT32 | at5g57740 | -1.3 | 0.0 | -0.5 | C09:1..40126856:25540948-25541984 |
| TCONS_00009294 | uncharacterized protein | at5g54970 | -1.3 | -0.2 | -1.0 | C03:1..57781463:6520466-6520923 |
| TCONS_00027234 | cytochrome P450 | at3g26160 | -1.3 | -0.3 | -0.2 | C07:1..48346208:32056741-32058550 |
| TCONS_00037127 | AT5g13880 MAC12 16 | at5g13880 | -1.3 | -0.6 | -0.5 | C09:1..40126856:35197499-35198936 |
| TCONS_00008145 | -NA- | at5g02480 | -1.3 | -0.4 | -0.6 | C03:1..57781463:134364-136757 |
| TCONS_00037196 | AT5g13100 T19L5 60 | at5g13100 | -1.3 | -0.3 | -0.7 | C09:1..40126856:35669214-35670472 |
| TCONS_00023687 | serine threonine kinase | at4g23190 | -1.3 | -0.2 | -0.7 | C06:1..40704471:23582549-23585647 |
| TCONS_00012363 | autoinhibited Ca2+-ATPase 1 | at1g27770 | -1.3 | -0.7 | -1.1 | C03:1..57781463:43772495-43776374 |
| TCONS_00010564 | F-box SKIP27-like | at4g05010 | -1.3 | -0.4 | -0.7 | C03:1..57781463:17808867-17810061 |
| TCONS_00033261 | phospholipid-transporting ATPase 4-like | at1g13210 | -1.3 | -0.0 | -0.9 | C08:1..41516064:38371059-38372735 |
| TCONS_00011569 | hypothetical protein ARALYDRAFT 899811 | -- | -1.3 | 0.5 | -0.7 | C03:1..57781463:28133526-28134121 |
| TCONS_00027958 | diacylglycerol O-acyltransferase 2 | at3g51520 | -1.3 | -0.2 | -0.8 | C07:1..48346208:37782876-37784413 |
| TCONS_00030218 | -NA- | -- | -1.3 | -0.0 | -1.0 | C08:1..41516064:9843235-9844487 |
| TCONS_00009673 | O-fucosyltransferase family | at2g37980 | -1.3 | 0.0 | -0.0 | C03:1..57781463:9880886-9882781 |
| TCONS_00035700 | trehalose-phosphate phosphatase | at5g51460 | -1.3 | -0.3 | -0.1 | C09:1..40126856:17601076-17602988 |
| TCONS_00026312 | -NA- | at1g30040 | -1.3 | 0.1 | -0.7 | C07:1..48346208:18127764-18129426 |
| TCONS_00029228 | heptahelical transmembrane HHP4 | at4g37680 | -1.3 | -0.0 | -0.5 | C07:1..48346208:47770475-47772354 |
| TCONS_00032598 | AF411794 1 At2g24550 | at2g24550 | -1.3 | -0.3 | -0.6 | C08:1..41516064:34296133-34298349 |
| TCONS_00033466 | glutamyl-tRNA reductase | at1g09940 | -1.3 | -0.4 | -0.7 | C08:1..41516064:39540463-39541345 |
| TCONS_00003477 | hypothetical protein ARALYDRAFT 478815 | at3g14060 | -1.3 | 0.3 | -0.0 | C01:1..38761720:34803741-34804351 |
| TCONS_00012141 | armadillo beta-catenin repeat family | at3g47820 | -1.3 | -0.1 | -0.5 | C03:1..57781463:39891218-39893524 |
| TCONS_00018698 | MAC Perforin domain-containing | at1g14780 | -1.3 | -0.4 | -0.9 | C05:1..32828328:5451136-5455593 |
| TCONS_00007632 | LOR9 ARATH ame: Full= LURP-one-related 9 | at3g10986 | -1.3 | -0.5 | -0.6 | C03:1..57781463:20247481-20321532 |
| TCONS_00037703 | cytokinin riboside 5 -monophosphate phosphoribohydrolase LOG7-like | -- | -1.3 | -0.2 | -0.7 | C09:1..40126856:38674058-38675134 |
| TCONS_00003306 | -NA- | -- | -1.3 | -0.4 | -1.1 | C01:1..38761720:32732435-32733412 |
| TCONS_00011838 | NAC domain-containing 62 | at3g49530 | -1.3 | -0.3 | -1.1 | C03:1..57781463:31316162-31318480 |
| TCONS_00030323 | -NA- | -- | -1.3 | -1.3 | 0.6 | C08:1..41516064:11786613-11788100 |
| TCONS_00028998 | WRKY DNA-binding 18 | at4g31800 | -1.3 | -0.3 | -0.7 | C07:1..48346208:46176077-46178337 |
| TCONS_00029089 | phosphatase 2C family | at4g33920 | -1.3 | -0.3 | -1.1 | C07:1..48346208:46742381-46744637 |
| TCONS_00018918 | RING U-box domain-containing | at1g17147 | -1.3 | -0.1 | -0.9 | C05:1..32828328:6970179-6971953 |
| TCONS_00019463 | UPF0496 At1g20180-like | at1g20180 | -1.3 | -0.0 | -0.3 | C05:1..32828328:15633100-15635972 |
| TCONS_00002293 | glycine-rich DOT1-like | -- | -1.3 | -0.1 | -0.0 | C01:1..38761720:14242076-14242788 |
| TCONS_00024855 | -NA- | -- | -1.3 | 0.0 | -0.8 | C06:1..40704471:40574201-40574865 |
| TCONS_00000650 | gata transcription factor 5-like | at4g36240 | -1.3 | -0.4 | -0.8 | C01:1..38761720:1378675-1379813 |
| TCONS_00003060 | protein | at3g20898 | -1.3 | 0.0 | -0.5 | C01:1..38761720:29613907-29614784 |
| TCONS_00036726 | aquaporin SIP1-2 | at5g18290 | -1.3 | 0.2 | -0.2 | C09:1..40126856:31589196-31591186 |
| TCONS_00025433 | phototropic-responsive NPH3 family | at5g66560 | -1.3 | -0.5 | -1.2 | C07:1..48346208:904508-905257 |
| TCONS_00014147 | trypsin inhibitor 1 | p26780|iti2_sinal | -1.3 | -0.1 | -0.8 | C04:1..40895475:1565360-1596681 |
| TCONS_00028740 | indole-3-acetic acid-amido synthetase | at4g27260 | -1.3 | 0.1 | -0.3 | C07:1..48346208:44390473-44393486 |
| TCONS_00029724 | phospholipid glycerol acyltransferase family | at1g01610 | -1.3 | -0.8 | -1.4 | C08:1..41516064:41284076-41294724 |
| TCONS_00023902 | probable polyamine oxidase 2-like | at3g59050 | -1.3 | -0.1 | -0.3 | C06:1..40704471:27191239-27193283 |
| TCONS_00026763 | late embryogenesis abundant (LEA) hydroxyproline-rich glyco family member | at5g56050 | -1.3 | -0.6 | -0.8 | C07:1..48346208:26829591-26831033 |
| TCONS_00015681 | Reticuline oxidase | at2g34810 | -1.3 | -0.0 | -0.5 | C04:1..40895475:21363706-21365958 |
| TCONS_00022310 | probable galacturonosyltransferase-like 9-like | at1g70090 | -1.3 | -0.6 | -0.7 | C06:1..40704471:5113508-5114924 |
| TCONS_00034723 | F-box LRR-repeat | at5g27920 | -1.3 | -0.3 | -0.5 | C09:1..40126856:3311267-3312508 |
| TCONS_00000758 | sbp (s-ribonuclease binding protein) family protein | at4g35070 | -1.3 | -0.2 | -0.7 | C01:1..38761720:2001240-2002691 |
| TCONS_00013201 | glutamine amidotransferase class-I domain-containing | at4g30530 | -1.3 | 0.4 | -0.7 | C03:1..57781463:54531250-54531756 |
| TCONS_00007015 | non-LTR retroelement reverse transcriptase | at1g10000 | -1.3 | -0.2 | -0.7 | C02:1..44046003:39199691-39200763 |
| TCONS_00037463 | -NA- | -- | -1.3 | -0.1 | -0.6 | C09:1..40126856:37527602-37528912 |
| TCONS_00023391 | serine acetyltransferase | at1g55920 | -1.3 | 0.2 | -1.0 | C06:1..40704471:18991473-18992982 |
| TCONS_00008901 | Chaperonin-like isoform 1 | at5g19855 | -1.3 | 0.0 | -0.5 | C03:1..57781463:4141042-4143075 |
| TCONS_00019015 | At1g32920 F9L11 25 | at1g32920 | -1.3 | -0.2 | -0.7 | C05:1..32828328:9583725-9584681 |
| TCONS_00030549 | ethylene-responsive transcription factor CRF4 | at4g27950 | -1.3 | -0.1 | -1.0 | C08:1..41516064:15709682-15711038 |
| TCONS_00022598 | -NA- | -- | -1.3 | -0.2 | -0.4 | C06:1..40704471:8252700-8253482 |
| TCONS_00018781 | pentatricopeptide repeat-containing chloroplastic-like | at1g15510 | -1.3 | 0.4 | -0.2 | C05:1..32828328:5896959-5901464 |
| TCONS_00037135 | ZINC INDUCED FACILITATOR-LIKE 1 | at5g13750 | -1.3 | 0.0 | -0.4 | C09:1..40126856:35246480-35250639 |
| TCONS_00034935 | RING U-box superfamily with ARM repeat domain isoform 1 | at5g67340 | -1.3 | 0.0 | -0.4 | C09:1..40126856:4934272-4936613 |
| TCONS_00002785 | atp sulfurylase | at4g14680 | -1.3 | -0.2 | -1.1 | C01:1..38761720:24928393-24930732 |
| TCONS_00024714 | nodulin 21 family | at1g44800 | -1.3 | -0.1 | -0.7 | C06:1..40704471:37939892-37943265 |
| TCONS_00014093 | pectinesterase family | at2g43050 | -1.3 | 0.3 | -1.1 | C04:1..40895475:1234292-1235621 |
| TCONS_00037174 | ethylene-responsive transcription factor ERF113 | at5g13330 | -1.3 | -0.5 | -0.5 | C09:1..40126856:35509572-35512489 |
| TCONS_00032914 | probable WRKY transcription factor 72 | at1g18860 | -1.2 | -0.0 | -0.0 | C08:1..41516064:36396114-36398918 |
| TCONS_00032707 | mitochondrial dicarboxylate carrier | at2g22500 | -1.2 | -0.1 | -0.8 | C08:1..41516064:35271041-35272882 |
| TCONS_00019370 | probable carboxylesterase 1 | at1g19190 | -1.2 | 0.0 | -0.6 | C05:1..32828328:14664294-14665608 |
| TCONS_00017495 | AT-rich interactive domain-containing 1-like | at2g46040 | -1.2 | -0.0 | -0.7 | C04:1..40895475:40566663-40569212 |
| TCONS_00008527 | NAC domain IPR003441 | at5g13180 | -1.2 | 0.1 | -0.4 | C03:1..57781463:2143842-2145603 |
| TCONS_00018571 | phosphatase 2A regulatory subunit B eta | at1g13460 | -1.2 | -0.5 | -1.2 | C05:1..32828328:3972628-3981639 |
| TCONS_00033494 | Reticuline oxidase | at5g44400 | -1.2 | 0.3 | 0.0 | C08:1..41516064:39740442-39741368 |
| TCONS_00023031 | Actin cross-linking | at1g69900 | -1.2 | -0.0 | -0.4 | C06:1..40704471:14274497-14276994 |
| TCONS_00006307 | at3g10020 t22k18 16 | at3g10020 | -1.2 | 0.0 | -0.1 | C02:1..44046003:24053648-24055213 |
| TCONS_00035723 | hydroxyproline-rich glyco family | at5g51680 | -1.2 | -0.2 | -0.6 | C09:1..40126856:18115140-18116572 |
| TCONS_00030084 | serine hydroxymethyltransferase 1-like | at4g13930 | -1.2 | 0.0 | -0.5 | C08:1..41516064:6934405-6935630 |
| TCONS_00010581 | AT4g03420 F9H3 4 | at4g03420 | -1.2 | 0.2 | -0.7 | C03:1..57781463:18021878-18023844 |
| TCONS_00023372 | S-adenosyl-L-methionine-dependent methyltransferases superfamily | at1g55450 | -1.2 | -0.0 | -1.1 | C06:1..40704471:18796492-18798143 |
| TCONS_00031230 | purine permease 17 | at1g19770 | -1.2 | 0.0 | -0.7 | C08:1..41516064:22234456-22236458 |
| TCONS_00017950 | AP2 B3-like transcriptional factor family | at1g01030 | -1.2 | -0.1 | -0.3 | C05:1..32828328:352921-355292 |
| TCONS_00013372 | -NA- | -- | -1.2 | 0.2 | -0.6 | C03:1..57781463:56783037-56784007 |
| TCONS_00024104 | ABC transporter G family member 36-like | at1g59870 | -1.2 | -0.1 | -0.8 | C06:1..40704471:29953584-29957681 |
| TCONS_00035125 | serine threonine kinase | at4g00970 | -1.2 | -0.2 | -0.5 | C09:1..40126856:6732624-6735518 |
| TCONS_00008957 | sucrose synthase 1 [Arabidopsis thaliana] | at5g20830 | -1.2 | 0.4 | -0.0 | C03:1..57781463:4429074-4430808 |
| TCONS_00000514 | heat shock 70 kDa mitochondrial-like | at4g37910 | -1.2 | -0.6 | -0.9 | C01:1..38761720:562627-564576 |
| TCONS_00037199 | WRKY DNA-binding 75 | at5g13080 | -1.2 | -0.2 | -0.7 | C09:1..40126856:35683468-35684800 |
| TCONS_00017219 | -NA- | -- | -1.2 | -1.7 | 0.1 | C04:1..40895475:38695446-38696801 |
| TCONS_00033236 | -NA- | at1g13520 | -1.2 | 0.1 | -0.3 | C08:1..41516064:38202011-38203290 |
| TCONS_00035821 | -NA- | at5g53486 | -1.2 | 0.0 | -0.3 | C09:1..40126856:20636259-20637767 |
| TCONS_00031667 | nodulin glutamate-ammonia ligase | at3g53180 | -1.2 | -0.0 | -0.1 | C08:1..41516064:28220482-28222961 |
| TCONS_00003282 | COBRA 7-like | at3g16860 | -1.2 | -0.3 | -0.9 | C01:1..38761720:32377297-32379480 |
| TCONS_00032410 | phosphatase 2C 49 | at3g62260 | -1.2 | -0.2 | -0.4 | C08:1..41516064:33170237-33172284 |
| TCONS_00037908 | polyubiquitin 3 | at5g03240 | -1.2 | 0.0 | -0.3 | C09:1..40126856:39786776-39788568 |
| TCONS_00008763 | -NA- | -- | -1.2 | -0.1 | -0.6 | C03:1..57781463:3487507-3489399 |
| TCONS_00005923 | nudix hydrolase mitochondrial-like | at1g73540 | -1.2 | -0.4 | -2.0 | C02:1..44046003:16738818-16739803 |
| TCONS_00033390 | kinase family | at1g11050 | -1.2 | -0.2 | -0.5 | C08:1..41516064:39128688-39130713 |
| TCONS_00003466 | -NA- | -- | -1.2 | 0.0 | 0.1 | C01:1..38761720:34775915-34777088 |
| TCONS_00026893 | VQ motif-containing | at5g46780 | -1.2 | -0.3 | -0.8 | C07:1..48346208:28511669-28512921 |
| TCONS_00022485 | kinase chloroplastic-like | at1g69790 | -1.2 | -0.2 | -0.5 | C06:1..40704471:7099195-7102796 |
| TCONS_00020080 | jasmonate-zim-domain 3 | at3g17860 | -1.2 | -0.0 | -0.4 | C05:1..32828328:24811009-24813151 |
| TCONS_00008970 | E3 ubiquitin- ligase AIP2-like isoform 1 | at5g20910 | -1.2 | -0.0 | -0.6 | C03:1..57781463:4470225-4472813 |
| TCONS_00015794 | proline transporter 2 | at3g55740 | -1.2 | -0.0 | -0.9 | C04:1..40895475:22893763-22896602 |
| TCONS_00002935 | ethylene receptor | at3g23150 | -1.2 | 0.1 | -0.4 | C01:1..38761720:27220862-27223520 |
| TCONS_00018907 | secologanin synthase-like | at1g17060 | -1.2 | -0.2 | -0.7 | C05:1..32828328:6913241-6916841 |
| TCONS_00035571 | UDP-glycosyltransferase 91C1-like | at5g49690 | -1.2 | -0.2 | -0.8 | C09:1..40126856:13449456-13451358 |
| TCONS_00010404 | cytochrome c-like | p00051|cyc_cucma | -1.2 | -0.1 | -1.0 | C03:1..57781463:16038752-16039697 |
| TCONS_00017135 | nodulin family | at2g39210 | -1.2 | 0.2 | -1.2 | C04:1..40895475:38039637-38042458 |
| TCONS_00000577 | heat shock transcription factor HSF4 | at4g36990 | -1.2 | -0.2 | -0.7 | C01:1..38761720:1014151-1016074 |
| TCONS_00019780 | uncharacterized RNA methyltransferase CT0009-like | at3g21300 | -1.2 | -0.4 | -0.8 | C05:1..32828328:19772592-19776203 |
| TCONS_00028337 | methyltransferase PMT21 | at4g19120 | -1.2 | -0.1 | -0.7 | C07:1..48346208:41310702-41314582 |
| TCONS_00016767 | leucine-rich repeat receptor kinase | at2g33170 | -1.2 | 0.0 | -0.5 | C04:1..40895475:35091176-35095182 |
| TCONS_00032755 | hypothetical protein EUTSA v10000578mg | at2g21780 | -1.2 | -0.2 | -0.4 | C08:1..41516064:35598899-35599890 |
| TCONS_00033188 | kinase chloroplastic-like | at1g14370 | -1.2 | -0.3 | -0.4 | C08:1..41516064:37872044-37873184 |
| TCONS_00026618 | NPR1 3 | -- | -1.2 | -0.5 | -0.5 | C07:1..48346208:24803976-24804593 |
| TCONS_00014663 | nodulin family | at2g39210 | -1.2 | -0.5 | -1.5 | C04:1..40895475:6686754-6688376 |
| TCONS_00032318 | BON association 1 | at3g61190 | -1.2 | -0.0 | -0.1 | C08:1..41516064:32540069-32541732 |
| TCONS_00013967 | nodulin 21 family | at2g40900 | -1.2 | -0.2 | -1.2 | C04:1..40895475:217050-232999 |
| TCONS_00023108 | zinc finger | at3g53600 | -1.2 | -0.3 | -1.1 | C06:1..40704471:15890125-15891631 |
| TCONS_00030026 | bZIP transcription factor | at1g42990 | -1.2 | -0.3 | -0.8 | C08:1..41516064:3794917-3796874 |
| TCONS_00016562 | mitogen-activated kinase kinase kinase 13 | at2g30040 | -1.2 | 0.1 | -0.1 | C04:1..40895475:32690127-32691784 |
| TCONS_00018347 | RAB GTPase homolog A2B | at1g07410 | -1.2 | -0.1 | -0.6 | C05:1..32828328:2543608-2544758 |
| TCONS_00028806 | 26S proteasome non-ATPase regulatory subunit 2 1A-like | at2g20580 | -1.2 | -0.2 | -0.8 | C07:1..48346208:44756327-44758306 |
| TCONS_00036722 | probable receptor kinase | at5g18500 | -1.2 | -0.2 | -0.6 | C09:1..40126856:31534130-31536857 |
| TCONS_00037606 | unnamed protein product | at5g06980 | -1.2 | 0.1 | -1.4 | C09:1..40126856:37999425-38001141 |
| TCONS_00027239 | cytochrome P450 | at3g26200 | -1.2 | 0.1 | -0.3 | C07:1..48346208:32096552-32099011 |
| TCONS_00022486 | homeobox-leucine zipper ATHB-13-like | at1g69780 | -1.2 | -0.0 | -0.8 | C06:1..40704471:7121178-7123220 |
| TCONS_00015105 | SNF1 related kinase | at3g23000 | -1.2 | 0.0 | -0.4 | C04:1..40895475:13448388-13449873 |
| TCONS_00004111 | dehydroascorbate reductase | at1g75270 | -1.2 | 1.0 | -0.2 | C02:1..44046003:17889214-17893285 |
| TCONS_00037110 | major facilitator | at5g14130 | -1.2 | -0.2 | -0.5 | C09:1..40126856:35085749-35088778 |
| TCONS_00015146 | purple acid phosphatase 17 | at3g17790 | -1.2 | -0.3 | -0.4 | C04:1..40895475:13891439-13892928 |
| TCONS_00022096 | alpha beta-Hydrolases superfamily | at1g73480 | -1.2 | -0.3 | -0.8 | C06:1..40704471:3661596-3664287 |
| TCONS_00022022 | heat shock 101 | at1g74300 | -1.2 | -0.1 | 0.1 | C06:1..40704471:3238719-3241686 |
| TCONS_00026288 | MACPF domain-containing CAD1-like | at1g29690 | -1.2 | -0.2 | -0.7 | C07:1..48346208:17797118-17798793 |
| TCONS_00009851 | alpha beta hydrolase related | at2g40095 | -1.2 | 0.0 | -0.3 | C03:1..57781463:11127911-11129765 |
| TCONS_00002052 | xyloglucan endotransglucosylase hydrolase 22 | at4g25810 | -1.2 | -0.3 | -1.2 | C01:1..38761720:11886324-11887791 |
| TCONS_00008572 | AT5g13880 MAC12 16 | at5g13880 | -1.2 | 0.1 | -0.0 | C03:1..57781463:2337561-2339112 |
| TCONS_00035948 | calcium-binding PBP1-like | at4g27280 | -1.2 | -0.5 | -1.3 | C09:1..40126856:22689797-22690704 |
| TCONS_00029757 | hypothetical protein EUTSA v10011959mg | at1g49800 | -1.2 | -0.0 | -0.8 | C08:1..41516064:898788-899515 |
| TCONS_00035605 | cytochrome c | at4g10040 | -1.2 | -0.3 | -0.3 | C09:1..40126856:14282409-14284488 |
| TCONS_00019208 | UDP-glycosyltransferase 91C1-like | at5g49690 | -1.2 | 0.0 | -0.6 | C05:1..32828328:12482615-12484709 |
| TCONS_00026017 | domain-containing GPI-anchored | at2g17120 | -1.2 | -0.3 | -1.0 | C07:1..48346208:13021197-13023709 |
| TCONS_00021862 | hypothetical protein EUTSA v10018992mg | at1g76070 | -1.2 | -0.0 | -0.8 | C06:1..40704471:1900206-1901736 |
| TCONS_00035771 | -NA- | at5g53050 | -1.2 | -0.0 | -0.3 | C09:1..40126856:19733052-19737063 |
| TCONS_00005194 | MTD1 family | at5g21940 | -1.2 | -0.1 | 0.1 | C02:1..44046003:6050031-6051569 |
| TCONS_00015387 | mitogen-activated kinase kinase kinase 13 | at2g30040 | -1.2 | 0.2 | -0.0 | C04:1..40895475:17447822-17449528 |
| TCONS_00037060 | retrovirus-related POL poly s | -- | -1.2 | 0.0 | -0.5 | C09:1..40126856:34368643-34369184 |
| TCONS_00010749 | C2 calcium lipid-binding plant phosphoribosyltransferase family | at4g00700 | -1.2 | -0.4 | -0.4 | C03:1..57781463:19524878-19528832 |
| TCONS_00016930 | late embryogenesis abundant hydroxyproline-rich glyco | at2g35960 | -1.2 | 0.2 | -0.9 | C04:1..40895475:36539488-36540818 |
| TCONS_00018733 | phosphatidylserine synthase 2 | at1g15110 | -1.2 | -0.0 | -0.6 | C05:1..32828328:5690962-5694200 |
| TCONS_00034799 | exonuclease family | at5g61390 | -1.2 | -0.8 | -0.5 | C09:1..40126856:3878344-3880933 |
| TCONS_00011052 | LURP-one-related 12-like | at3g15810 | -1.2 | -0.1 | -1.0 | C03:1..57781463:21713713-21715763 |
| TCONS_00010949 | organic cation carnitine transporter 7-like | at3g13050 | -1.2 | 0.1 | -0.6 | C03:1..57781463:20976715-20977599 |
| TCONS_00026333 | ---NA--- | at1g49050 | -1.2 | -0.1 | -0.8 | C07:1..48346208:18636825-18637935 |
| TCONS_00023629 | -NA- | at1g52410 | -1.2 | -0.4 | -1.1 | C06:1..40704471:22227339-22228620 |
| TCONS_00020167 | -NA- | at1g10700 | -1.2 | 0.0 | -0.4 | C05:1..32828328:25454778-25455717 |
| TCONS_00012434 | At1g29190 F28N24 12 | at1g29195 | -1.1 | -0.1 | -0.6 | C03:1..57781463:44596288-44597791 |
| TCONS_00018588 | Glucan endo-1,3-beta-glucosidase | at1g13830 | -1.1 | 0.0 | -0.4 | C05:1..32828328:4178522-4180035 |
| TCONS_00016731 | leucine-rich repeat disease resistance -like | at2g32680 | -1.1 | 0.0 | -0.4 | C04:1..40895475:34657678-34661001 |
| TCONS_00032613 | transcription factor bHLH66-like | at2g24260 | -1.1 | -0.1 | -0.4 | C08:1..41516064:34421194-34423103 |
| TCONS_00031235 | Serine arginine repetitive matrix | at3g48020 | -1.1 | 0.0 | -0.5 | C08:1..41516064:22327404-22328190 |
| TCONS_00026007 | serine threonine-specific kinase kin3 | at2g17220 | -1.1 | -0.2 | -0.8 | C07:1..48346208:12903130-12905732 |
| TCONS_00027219 | homocysteine S-methyltransferase 1 | at3g25900 | -1.1 | 0.1 | -0.5 | C07:1..48346208:31926680-31927913 |
| TCONS_00022370 | DNA polymerase epsilon subunit 2-like | at5g22110 | -1.1 | 0.2 | -0.1 | C06:1..40704471:5868949-5869949 |
| TCONS_00027526 | -NA- | at5g47920 | -1.1 | 0.1 | -0.4 | C07:1..48346208:34581792-34582861 |
| TCONS_00034150 | TOE2 | at5g60142 | -1.1 | -0.3 | -0.7 | C09:1..40126856:27948756-27953928 |
| TCONS_00022368 | F-box family | at2g27310 | -1.1 | -0.1 | -0.3 | C06:1..40704471:5846809-5848183 |
| TCONS_00010103 | pectinesterase 2-like | at2g45220 | -1.1 | -0.0 | -0.5 | C03:1..57781463:13200356-13202655 |
| TCONS_00010058 | Methionine aminopeptidase 2 | at2g44180 | -1.1 | 0.2 | -0.6 | C03:1..57781463:12896929-12898316 |
| TCONS_00018362 | GTP-binding Obg | at1g07620 | -1.1 | -0.1 | -0.3 | C05:1..32828328:2644023-2645767 |
| TCONS_00015713 | DCD (Development and Cell Death) domain | at2g35150 | -1.1 | -0.1 | -0.2 | C04:1..40895475:21543082-21547638 |
| TCONS_00014711 | aspartyl protease family | at2g39710 | -1.1 | -0.0 | -0.4 | C04:1..40895475:7129289-7131099 |
| TCONS_00026859 | WRKY DNA-binding 28 | at5g46350 | -1.1 | 0.1 | -0.4 | C07:1..48346208:28156967-28157778 |
| TCONS_00024290 | polyubiquitin | at4g05050 | -1.1 | -0.1 | -0.5 | C06:1..40704471:31437948-31439402 |
| TCONS_00020741 | basic endochitinase | q09023|chi2_brana | -1.1 | 0.6 | -0.6 | C05:1..32828328:30177338-30178208 |
| TCONS_00006885 | phospholipid-transporting atpase 9-like | at3g25610 | -1.1 | -0.0 | -0.1 | C02:1..44046003:37085387-37089002 |
| TCONS_00002705 | transcription factor bHLH112 | at1g61660 | -1.1 | -0.4 | -0.8 | C01:1..38761720:22965141-22966668 |
| TCONS_00010356 | phi-1-like phosphate-induced | at4g08950 | -1.1 | -0.1 | -1.3 | C03:1..57781463:15570328-15571749 |
| TCONS_00020810 | U-box domain-containing 24 | at3g11840 | -1.1 | -0.0 | -0.7 | C05:1..32828328:30543439-30545771 |
| TCONS_00019291 | nudix hydrolase 4-like | at1g18300 | -1.1 | -0.1 | -0.8 | C05:1..32828328:14016526-14017788 |
| TCONS_00012468 | MACPF domain-containing CAD1-like | at1g29690 | -1.1 | 0.0 | -0.7 | C03:1..57781463:44889718-44890626 |
| TCONS_00023958 | methylenetetrahydrofolate reductase 1 | at3g59970 | -1.1 | 0.0 | -1.1 | C06:1..40704471:27683845-27684419 |
| TCONS_00026617 | mitochondrial transcription termination factor family | at5g45113 | -1.1 | -0.0 | -0.4 | C07:1..48346208:24799586-24803794 |
| TCONS_00023955 | ARGOS | at3g59900 | -1.1 | 0.1 | -0.2 | C06:1..40704471:27670505-27671662 |
| TCONS_00030418 | RING zinc finger -like | at3g16720 | -1.1 | 0.0 | -0.8 | C08:1..41516064:13810931-13812482 |
| TCONS_00011075 | Calcium-dependent lipid-binding domain-containing | at3g16510 | -1.1 | -0.3 | -0.7 | C03:1..57781463:22241471-22243014 |
| TCONS_00034456 | TIC 20- chloroplastic-like | at4g03320 | -1.1 | -0.0 | -0.5 | C09:1..40126856:1549393-1551270 |
| TCONS_00026498 | AF428370 1 At1g63720 F24D7 9 | at1g63720 | -1.1 | -0.1 | -0.4 | C07:1..48346208:23021280-23023217 |
| TCONS_00007679 | 12-oxophytodienoate reductase 3-like | at2g06050 | -1.1 | -0.5 | -1.3 | C03:1..57781463:27298670-27303342 |
| TCONS_00017067 | heme-binding chloroplastic-like | at2g37970 | -1.1 | -0.1 | -0.8 | C04:1..40895475:37530598-37533492 |
| TCONS_00035268 | ras-related small GTP-binding | at5g47200 | -1.1 | -0.1 | -0.6 | C09:1..40126856:8926794-8928061 |
| TCONS_00025762 | -NA- | at2g19710 | -1.1 | 0.0 | -0.3 | C07:1..48346208:7527940-7532007 |
| TCONS_00009517 | glutathione peroxidase | at2g31570 | -1.1 | -0.1 | -0.7 | C03:1..57781463:8503867-8506137 |
| TCONS_00000347 | Sulfite exporter family | at1g61740 | -1.1 | -0.4 | -0.4 | C01:1..38761720:23144426-23149915 |
| TCONS_00010825 | Ninja-family AFP3 | at3g02140 | -1.1 | -0.1 | -0.0 | C03:1..57781463:19931884-19933515 |
| TCONS_00000163 | serine threonine kinase | at1g61610 | -1.1 | -0.5 | -0.9 | C01:1..38761720:22811191-22829356 |
| TCONS_00025764 | phospholipase A2-beta | at2g19690 | -1.1 | -0.4 | -0.8 | C07:1..48346208:7535282-7536692 |
| TCONS_00003771 | calcium-binding CML49 | at3g10300 | -1.1 | -0.1 | -0.6 | C01:1..38761720:37276988-37278829 |
| TCONS_00028537 | UDP-glucose 4-epimerase | at4g23930 | -1.1 | -0.0 | -0.5 | C07:1..48346208:43287621-43291607 |
| TCONS_00011103 | serine threonine kinase | at3g17410 | -1.1 | -0.2 | -0.3 | C03:1..57781463:22540082-22541328 |
| TCONS_00018462 | Intracellular transport | at1g08800 | -1.1 | -0.0 | -0.7 | C05:1..32828328:3214910-3218379 |
| TCONS_00007054 | at3g29240 mxo21 9 | at3g29240 | -1.1 | 0.0 | -0.2 | C02:1..44046003:39639219-39640473 |
| TCONS_00028358 | hypothetical protein EUTSA v10025579mg | -- | -1.1 | -0.2 | -1.1 | C07:1..48346208:41687640-41687816 |
| TCONS_00027402 | xyloglucan glycosyltransferase 4 | at3g28180 | -1.1 | -0.1 | -0.9 | C07:1..48346208:33483492-33485126 |
| TCONS_00011959 | phytic acid metabolising | at3g45090 | -1.1 | 0.0 | -0.5 | C03:1..57781463:34512389-34515236 |
| TCONS_00008537 | ethylene-responsive transcription factor ERF113 | at5g13330 | -1.1 | -0.1 | -0.5 | C03:1..57781463:2208158-2211043 |
| TCONS_00001405 | 26s proteasome regulatory subunit | at2g20580 | -1.1 | 0.0 | -0.4 | C01:1..38761720:5808122-5810357 |
| TCONS_00014786 | syntaxin synt4 | at3g52400 | -1.1 | -0.0 | -0.8 | C04:1..40895475:8243140-8244202 |
| TCONS_00019559 | probable calcium-binding CML44-like | at1g21550 | -1.1 | -0.2 | -0.9 | C05:1..32828328:16917066-16918057 |
| TCONS_00026860 | WRKY DNA-binding 28 | at5g46350 | -1.1 | -0.2 | -0.5 | C07:1..48346208:28157873-28160484 |
| TCONS_00005214 | CCR4-associated factor 1 | at5g22250 | -1.1 | -0.2 | -1.1 | C02:1..44046003:6239297-6241550 |
| TCONS_00006376 | allene oxide synthase | at5g42650 | -1.1 | 0.1 | -0.6 | C02:1..44046003:25711862-25714234 |
| TCONS_00016954 | ATPDR6 PDR6 | at2g36380 | -1.1 | 0.1 | 0.0 | C04:1..40895475:36820789-36822760 |
| TCONS_00030006 | phosphatase 2C 52 | at1g03590 | -1.1 | -0.2 | -0.8 | C08:1..41516064:3035800-3038110 |
| TCONS_00023156 | kinase family | at3g61960 | -1.1 | -0.5 | -0.9 | C06:1..40704471:16382847-16384868 |
| TCONS_00021317 | leucine-rich repeat family | at1g74190 | -1.1 | 0.0 | -0.1 | C06:1..40704471:32944398-32962496 |
| TCONS_00034862 | nodulin 3 family | at5g23660 | -1.1 | -0.5 | -0.5 | C09:1..40126856:4212570-4214908 |
| TCONS_00030205 | K+ uptake permease 9 | at4g19960 | -1.1 | -0.2 | -1.5 | C08:1..41516064:9160110-9163514 |
| TCONS_00004169 | probable inactive purple acid phosphatase 2-like | at2g03450 | -1.1 | -0.2 | -0.1 | C02:1..44046003:36448922-36457016 |
| TCONS_00018898 | probable rhamnose biosynthetic enzyme 1-like | at1g78570 | -1.1 | -0.4 | -1.1 | C05:1..32828328:6799877-6802814 |
| TCONS_00009379 | -NA- | -- | -1.1 | 0.0 | -0.2 | C03:1..57781463:7480209-7481318 |
| TCONS_00027630 | 80A08 24 | -- | -1.1 | 0.1 | 0.0 | C07:1..48346208:35323020-35324419 |
| TCONS_00013319 | TARGET OF EARLY ACTIVATION TAGGED 1 | at2g28550 | -1.1 | -0.0 | -0.5 | C03:1..57781463:55918896-55920676 |
| TCONS_00002910 | pho1-like protein | at3g23430 | -1.1 | -0.2 | -0.5 | C01:1..38761720:26724852-26726275 |
| TCONS_00002460 | heat shock protein 70 -interacting | at3g50030 | -1.1 | -0.2 | -0.6 | C01:1..38761720:18329747-18331814 |
| TCONS_00022652 | probable calcium-binding CML23-like | at5g37770 | -1.1 | -0.4 | -1.1 | C06:1..40704471:8921022-8922068 |
| TCONS_00008591 | major facilitator | at5g14120 | -1.1 | -0.5 | -0.7 | C03:1..57781463:2449743-2451991 |
| TCONS_00018364 | probable phosphatase 2C 4-like isoform 2 | at1g07630 | -1.1 | -0.3 | -0.4 | C05:1..32828328:2649384-2651169 |
| TCONS_00032028 | -NA- | at3g57450 | -1.1 | -0.0 | -0.9 | C08:1..41516064:30863978-30864725 |
| TCONS_00027605 | Pyridoxal phosphate (PLP)-dependent transferases superfamily | at5g26600 | -1.1 | 0.0 | -0.5 | C07:1..48346208:35085600-35087654 |
| TCONS_00032612 | basic helix-loop-helix family | at2g24260 | -1.1 | -0.3 | -0.4 | C08:1..41516064:34419926-34421143 |
| TCONS_00032616 | potassium channel tetramerization domain-containing | at2g24240 | -1.1 | -0.4 | -0.8 | C08:1..41516064:34433772-34436047 |
| TCONS_00016093 | -NA- | at4g12830 | -1.1 | 0.0 | -0.2 | C04:1..40895475:27202645-27204228 |
| TCONS_00018729 | -NA- | at1g15030 | -1.1 | -0.1 | -0.1 | C05:1..32828328:5666353-5667194 |
| TCONS_00003126 | probable beta-D-xylosidase 5-like | at3g19615 | -1.1 | 0.5 | -0.5 | C01:1..38761720:30481442-30482041 |
| TCONS_00028905 | double Clp-N motif-containing P-loop nucleoside triphosphate hydrolases superfamily | at4g30350 | -1.1 | -0.3 | -0.6 | C07:1..48346208:45632951-45634397 |
| TCONS_00033160 | MAC Perforin domain-containing | at1g14780 | -1.1 | -0.0 | -0.7 | C08:1..41516064:37686851-37688964 |
| TCONS_00002829 | potassium channel | at2g26650 | -1.1 | -0.0 | -0.3 | C01:1..38761720:25379229-25380725 |
| TCONS_00000759 | farnesylated protein | at4g35060 | -1.1 | -0.0 | -1.0 | C01:1..38761720:2014988-2016028 |
| TCONS_00021681 | probable pectin methyltransferase QUA2-like | at1g78240 | -1.1 | -0.6 | -1.2 | C06:1..40704471:796243-799298 |
| TCONS_00037868 | serine threonine- kinase CTR1 | at5g03730 | -1.1 | -0.0 | -0.5 | C09:1..40126856:39590060-39591760 |
| TCONS_00020405 | RING zinc finger -like | at3g16720 | -1.1 | -0.0 | -0.5 | C05:1..32828328:27136757-27138105 |
| TCONS_00023656 | 4-cumarate-COA-ligase | at1g51680 | -1.1 | -0.5 | -0.9 | C06:1..40704471:22688860-22692142 |
| TCONS_00005948 | desulfoglucosinolate sulfotransferase | at1g74100 | -1.1 | 0.2 | -0.6 | C02:1..44046003:17155591-17156985 |
| TCONS_00033154 | -NA- | -- | -1.1 | -0.2 | -0.9 | C08:1..41516064:37669119-37669319 |
| TCONS_00013151 | WRKY transcription factor 11 | at4g31550 | -1.1 | -0.4 | -1.0 | C03:1..57781463:54127793-54129558 |
| TCONS_00001539 | methyltransferase pmt20 | at4g19120 | -1.0 | -0.2 | -0.8 | C01:1..38761720:6963379-6966284 |
| TCONS_00019657 | formin 8 | at1g70140 | -1.0 | -0.5 | -0.3 | C05:1..32828328:17943635-17944949 |
| TCONS_00012029 | FAD NAD(P)-binding oxidoreductase family | at3g44190 | -1.0 | -0.2 | -0.6 | C03:1..57781463:36522616-36524324 |
| TCONS_00024550 | -NA- | at1g71880 | -1.0 | -0.2 | -0.4 | C06:1..40704471:34360159-34362351 |
| TCONS_00037290 | -NA- | -- | -1.0 | -0.4 | -1.0 | C09:1..40126856:36198390-36199044 |
| TCONS_00016441 | arogenate dehydratase prephenate dehydratase chloroplastic-like | at2g27820 | -1.0 | -0.3 | -0.6 | C04:1..40895475:30844456-30845875 |
| TCONS_00034889 | CDPK-related kinase 6 ( 6) | at5g24430 | -1.0 | -0.2 | -0.6 | C09:1..40126856:4439990-4440862 |
| TCONS_00009838 | nematode resistance | at2g40000 | -1.0 | -0.1 | -0.3 | C03:1..57781463:11059239-11064327 |
| TCONS_00029088 | Peroxisomal membrane Mpv17 PMP22 | at4g33890 | -1.0 | 0.0 | -0.3 | C07:1..48346208:46735434-46739828 |
| TCONS_00033585 | L-ascorbate peroxidase 1 | at1g07890 | -1.0 | -0.0 | -0.8 | C08:1..41516064:40133613-40136778 |
| TCONS_00009154 | probable serine threonine- kinase WNK4-like | at5g58350 | -1.0 | -0.1 | -0.5 | C03:1..57781463:5648152-5648959 |
| TCONS_00010086 | AP2 domain transcription factor | at2g44940 | -1.0 | 0.0 | -0.9 | C03:1..57781463:13080327-13081632 |
| TCONS_00016241 | glutaredoxin | -- | -1.0 | -0.1 | -0.9 | C04:1..40895475:29121797-29123505 |
| TCONS_00006903 | cytochrome P450 | at3g26200 | -1.0 | 0.2 | -0.3 | C02:1..44046003:37680475-37682461 |
| TCONS_00011495 | 12-oxophytodienoate reductase 3 | at2g06050 | -1.0 | -0.1 | -1.0 | C03:1..57781463:27303595-27305258 |
| TCONS_00030918 | invertase pectin methylesterase inhibitor family | at1g14890 | -1.0 | 0.0 | -0.2 | C08:1..41516064:19489288-19490310 |
| TCONS_00008137 | SMR3 ARATH ame: Full=Cyclin-dependent kinase inhibitor SMR3 ame: Full= SIAMESE-RELATED 3 | at5g02420 | -1.0 | 0.1 | -0.4 | C03:1..57781463:111324-112565 |
| TCONS_00029735 | cotton-Golgi related 3 | at5g65810 | -1.0 | -0.0 | -0.5 | C08:1..41516064:94136-95627 |
| TCONS_00014439 | zinc finger | at2g36320 | -1.0 | -0.0 | -0.3 | C04:1..40895475:4296831-4298699 |
| TCONS_00026217 | VQ motif-containing | at1g28280 | -1.0 | -0.1 | -0.5 | C07:1..48346208:16677970-16679885 |
| TCONS_00015755 | VQ motif-containing | at3g56880 | -1.0 | -0.6 | -0.7 | C04:1..40895475:21980929-21982365 |
| TCONS_00037111 | major facilitator | at5g14120 | -1.0 | -0.4 | -0.5 | C09:1..40126856:35088936-35090430 |
| TCONS_00036805 | uncharacterized protein | at5g17350 | -1.0 | -0.1 | -0.8 | C09:1..40126856:32224961-32226231 |
| TCONS_00027486 | phytochelatin synthetase | at3g29810 | -1.0 | -0.1 | -0.4 | C07:1..48346208:34235549-34236574 |
| TCONS_00033111 | L-type lectin-domain containing receptor kinase -like | at1g15530 | -1.0 | 0.0 | -0.4 | C08:1..41516064:37464238-37466526 |
| TCONS_00035838 | xyloglucan galactosyltransferase KATAMARI1-like | at2g20370 | -1.0 | -0.2 | -0.6 | C09:1..40126856:20963394-20966525 |
| TCONS_00001317 | regulator of vps4 activity protein | at4g29440 | -1.0 | -0.0 | -0.2 | C01:1..38761720:5022833-5025167 |
| TCONS_00037044 | mate efflux family | at1g61890 | -1.0 | -0.1 | -0.8 | C09:1..40126856:34266761-34268329 |
| TCONS_00018271 | probable alpha,alpha-trehalose-phosphate synthase [UDP-forming] 7-like | at1g06410 | -1.0 | -0.0 | -0.5 | C05:1..32828328:2217138-2220505 |
| TCONS_00014905 | -NA- | at3g57450 | -1.0 | -0.0 | -1.2 | C04:1..40895475:9589393-9590357 |
| TCONS_00011426 | serine threonine kinase | at2g04570 | -1.0 | -0.2 | -0.7 | C03:1..57781463:26775887-26778117 |
| TCONS_00008735 | syntaxin homologue | -- | -1.0 | -0.0 | -0.4 | C03:1..57781463:3239357-3241486 |
| TCONS_00028858 | -NA- | at4g29780 | -1.0 | -0.4 | -1.1 | C07:1..48346208:45327191-45329581 |
| TCONS_00032411 | phosphatase 2C 49 | -- | -1.0 | -0.4 | -0.6 | C08:1..41516064:33172459-33172975 |
| TCONS_00034470 | callose synthase 12-like | at4g03550 | -1.0 | -0.3 | -0.4 | C09:1..40126856:1659552-1663891 |
| TCONS_00033750 | Erythronate-4-phosphate dehydrogenase | at1g01500 | -1.0 | -0.1 | -0.6 | C08:1..41516064:41262777-41264321 |
| TCONS_00037092 | E3 ubiquitin- ligase RGLG2-like | at5g14420 | -1.0 | -0.1 | -0.1 | C09:1..40126856:34983459-34986183 |
| TCONS_00030258 | -NA- | -- | -1.0 | -0.1 | -0.6 | C08:1..41516064:10534984-10535131 |
| TCONS_00036946 | reversibly glycosylated polypeptide-3 | at5g15650 | -1.0 | -0.0 | -0.5 | C09:1..40126856:33280210-33280681 |
| TCONS_00022291 | cysteine-rich receptor kinase 2-like | at1g70520 | -1.0 | -0.4 | -0.8 | C06:1..40704471:4850856-4852712 |
| TCONS_00010060 | -NA- | at2g44200 | -1.0 | -0.1 | -0.4 | C03:1..57781463:12901992-12904109 |
| TCONS_00002296 | gag-pol poly | -- | -1.0 | -0.3 | -0.2 | C01:1..38761720:14246349-14247939 |
| TCONS_00011383 | CLAVATA3 ESR-related 41 | at3g24770 | -1.0 | -0.0 | -0.5 | C03:1..57781463:26303721-26305208 |
| TCONS_00020625 | ATPase E1-E2 type family haloacid dehalogenase-like hydrolase family | at3g13900 | -1.0 | 0.0 | -0.2 | C05:1..32828328:29161562-29163474 |
| TCONS_00006041 | L-ascorbate oxidase homolog | at1g76160 | -1.0 | -0.4 | -0.6 | C02:1..44046003:18757450-18759109 |
| TCONS_00012955 | uncharacterized partial | at1g57680 | -1.0 | -0.1 | -0.3 | C03:1..57781463:51881091-51883352 |
| TCONS_00011799 | 2-nitropropane dioxygenase | at5g64250 | -1.0 | 0.1 | -0.2 | C03:1..57781463:30931858-30934668 |
| TCONS_00036304 | E3 ubiquitin- ligase RING1-like | at5g59550 | -1.0 | -0.2 | -0.6 | C09:1..40126856:27383204-27385072 |
| TCONS_00031067 | trypsin and protease inhibitor family | at1g17860 | -1.0 | 0.1 | -0.5 | C08:1..41516064:20615074-20616898 |
| TCONS_00033587 | kinase family | at1g07870 | -1.0 | -0.2 | -0.5 | C08:1..41516064:40141588-40146053 |
| TCONS_00019260 | ATP-binding cassette transporter | at1g17840 | -1.0 | -0.4 | -0.8 | C05:1..32828328:13763451-13767007 |
| TCONS_00028982 | WRKY transcription factor 11 | at4g31550 | -1.0 | -0.4 | -1.2 | C07:1..48346208:46097642-46099449 |
| TCONS_00014532 | transcription factor AS1 | at2g37630 | -1.0 | -0.3 | -0.7 | C04:1..40895475:5136466-5138382 |
| TCONS_00000861 | cyclin d3 | at4g34160 | -1.0 | -0.1 | -0.6 | C01:1..38761720:2481529-2486536 |
| TCONS_00022727 | F-box At1g67340-like | at1g67340 | -1.0 | -0.0 | -0.0 | C06:1..40704471:9617046-9618644 |
| TCONS_00006072 | 12-oxophytodienoate reductase 1 | at1g76680 | -1.0 | -0.2 | -0.6 | C02:1..44046003:19208965-19210910 |
| TCONS_00019058 | heat shock 81-2 | at5g56000 | -1.0 | -0.2 | -0.9 | C05:1..32828328:10483817-10485166 |
| TCONS_00036960 | ---NA--- | at5g15470 | -1.0 | -0.3 | -0.5 | C09:1..40126856:33394865-33397080 |
| TCONS_00019066 | glycerol-3-phosphate acyltransferase | q39639|plsb_cucsa | -1.0 | -0.1 | -0.1 | C05:1..32828328:10535161-10536509 |
| TCONS_00021050 | callose synthase 9 | at3g07160 | -1.0 | -0.3 | -0.5 | C05:1..32828328:32330832-32332149 |
| TCONS_00026334 | -NA- | at1g49050 | -1.0 | -0.4 | -0.9 | C07:1..48346208:18638008-18639997 |
| TCONS_00031861 | DNA-binding ESCAROLA-like | at3g55560 | -1.0 | -0.1 | -0.6 | C08:1..41516064:29665790-29667733 |
| TCONS_00036302 | scarecrow 30 | at5g59450 | -1.0 | -0.4 | -0.5 | C09:1..40126856:27351814-27354636 |
| TCONS_00029784 | C3HC4-type RING finger-containing | at1g08050 | -1.0 | -0.1 | -0.5 | C08:1..41516064:1215168-1218265 |
| TCONS_00006595 | probable receptor-like protein kinase at5g20050-like | at5g20050 | -1.0 | -0.0 | -0.4 | C02:1..44046003:32191070-32192869 |
| TCONS_00005262 | C3H4 type zinc finger | at5g60710 | -1.0 | -0.0 | -0.2 | C02:1..44046003:6830102-6832246 |
| TCONS_00000679 | thaumatin-like protein 1-like | at4g36010 | -1.0 | -0.0 | -0.8 | C01:1..38761720:1582144-1584201 |
| TCONS_00021942 | F-box kelch-repeat At1g67480-like | at1g67480 | -1.0 | 0.0 | -0.3 | C06:1..40704471:2501705-2504417 |
| TCONS_00008406 | -NA- | -- | -1.0 | 0.0 | -0.3 | C03:1..57781463:1603239-1605349 |
| TCONS_00028455 | acyl- thioesterase 1-like | at4g22300 | -1.0 | -0.0 | -0.2 | C07:1..48346208:42388550-42390280 |
| TCONS_00003248 | protein tify 6b-like | at3g17860 | -1.0 | -0.1 | -0.4 | C01:1..38761720:31896056-31898148 |
| TCONS_00002876 | adenosylhomocysteinase | at3g23810 | -0.9 | -0.1 | -0.3 | C01:1..38761720:26054215-26056436 |
| TCONS_00036769 | Cax7 | at5g17860 | -0.9 | -0.2 | -0.5 | C09:1..40126856:31875727-31877843 |
| TCONS_00007228 | hydroxyproline-rich glyco family | at5g65660 | -0.9 | -0.1 | -0.0 | C02:1..44046003:41936498-41937844 |
| TCONS_00017208 | kinase family | at2g40270 | -0.9 | -0.1 | -0.5 | C04:1..40895475:38534593-38536585 |
| TCONS_00000527 | heptahelical transmembrane HHP4 | at4g37680 | -0.9 | -0.0 | -0.5 | C01:1..38761720:707064-709485 |
| TCONS_00001758 | cytochrome P450 | at4g22690 | -0.9 | -0.0 | -1.1 | C01:1..38761720:8992642-8994645 |
| TCONS_00028360 | Nucleotide-diphospho-sugar transferase family | at5g44820 | -0.9 | 0.1 | -0.8 | C07:1..48346208:41688850-41689460 |
| TCONS_00023960 | methionine aminopeptidase 2B | at3g59970 | -0.9 | 0.0 | -0.8 | C06:1..40704471:27684933-27688373 |
| TCONS_00011983 | mitogen-activated kinase 3 | at3g45640 | -0.9 | -0.1 | -0.6 | C03:1..57781463:35095355-35097505 |
| TCONS_00010975 | proteasome activating 200 | at3g13330 | -0.9 | 0.0 | -0.3 | C03:1..57781463:21082716-21086178 |
| TCONS_00028344 | abscisic acid 8 -hydroxylase 1 | at4g19230 | -0.9 | -0.2 | -0.6 | C07:1..48346208:41380978-41383742 |
| TCONS_00032996 | phospholipid-transporting ATPase 4-like | at1g17500 | -0.9 | -0.0 | -0.4 | C08:1..41516064:36856346-36857330 |
| TCONS_00002328 | scarecrow 30 | at3g46600 | -0.9 | -0.3 | -0.7 | C01:1..38761720:15167953-15170484 |
| TCONS_00023911 | callose synthase 7-like | at3g59100 | -0.9 | -0.1 | -0.2 | C06:1..40704471:27214259-27216596 |
| TCONS_00021656 | mechanosensitive channel of small conductance-like 5 | at1g78610 | -0.9 | -0.0 | -0.3 | C06:1..40704471:665372-667328 |
| TCONS_00017290 | vacuolar amino acid transporter 1-like | at2g41190 | -0.9 | -0.3 | -0.8 | C04:1..40895475:39163457-39166815 |
| TCONS_00029040 | glycine-rich | at4g32920 | -0.9 | -0.1 | -0.2 | C07:1..48346208:46437952-46442312 |
| TCONS_00010689 | -related isoform 1 | at4g01870 | -0.9 | -0.2 | -0.8 | C03:1..57781463:19064742-19067195 |
| TCONS_00022758 | Leucine-rich repeat transmembrane kinase | at1g53430 | -0.9 | -0.2 | -0.8 | C06:1..40704471:10163464-10165380 |
| TCONS_00020901 | nucleotide-sugar transporter family | at3g11320 | -0.9 | -0.1 | -0.6 | C05:1..32828328:31542163-31544555 |
| TCONS_00037043 | mate efflux family | at1g61890 | -0.9 | -0.4 | -0.7 | C09:1..40126856:34264844-34266423 |
| TCONS_00002157 | glycerophosphoryl diester phosphodiesterase 3 | at4g26690 | -0.9 | -0.2 | -0.5 | C01:1..38761720:12970626-12972552 |
| TCONS_00012248 | probable LRR receptor-like serine threonine- kinase | at5g65240 | -0.9 | 0.0 | -0.2 | C03:1..57781463:42511651-42512662 |
| TCONS_00006377 | l-type lectin-domain containing receptor kinase -like | at2g37710 | -0.9 | -0.0 | -0.6 | C02:1..44046003:25859667-25862117 |
| TCONS_00022683 | probable metal-nicotianamine transporter YSL7-like | at1g65730 | -0.9 | -0.0 | -0.4 | C06:1..40704471:9264877-9266307 |
| TCONS_00027641 | -NA- | at5g25820 | -0.9 | -0.3 | -0.6 | C07:1..48346208:35399514-35402602 |
| TCONS_00018691 | -NA- | at1g14710 | -0.9 | -0.1 | -0.4 | C05:1..32828328:5385570-5388819 |
| TCONS_00011545 | adenylyl-sulfate kinase chloroplastic-like | at2g14750 | -0.9 | 0.6 | -1.0 | C03:1..57781463:27757846-27759849 |
| TCONS_00031885 | Alpha beta hydrolase related | at3g55880 | -0.9 | -0.2 | -0.9 | C08:1..41516064:29907474-29910259 |
| TCONS_00027916 | sugar transport 3 | at5g61520 | -0.9 | 0.1 | -0.4 | C07:1..48346208:37365013-37366510 |
| TCONS_00005814 | probable ribose-5-phosphate isomerase-like | at1g71100 | -0.8 | -0.4 | -0.9 | C02:1..44046003:14482126-14483308 |
