# Supplementary material for: ABA signalling manipulation suppresses senescence of a leafy vegetable stored at room temperature
Source: Plant Biotechnol J. 2017 Aug 16;16(2):530–44. doi: 10.1111/pbi.12793 (PMC5787841; doi:10.1111/pbi.12793)
Supplement: Supplementary file 4 — Table S3 GO enrichment within the gene set up‐regulated or down‐regulated per each treatment. [file PBI-16-530-s001.docx]

# S3. GO enrichment within the gene set upregulated or downregulated per each treatment

## Summary up/down

Table S3.1. Summary of GO annotation and GO enrichment

|  |  |  | enriched GO | total GO terms |
| --- | --- | --- | --- | --- |
|  | annotated | total | FDR 0.05 | all |
| Population | 29412 | 37964 | - | 187824 |
| ABA-up | 227 | 273 | 234 | 1877 |
| ABA-down | 274 | 320 | 96 | 1738 |
| Pyr-up | 31 | 46 | 9 | 576 |
| Pyr-down | 15 | 25 | 11 | 348 |
| ABA_Pyr-up | 392 | 475 | 249 | 2251 |
| ABA_Pyr-down | 882 | 1015 | 194 | 3004 |

## ABA-up

Table S3.2. Enriched GO categories among the gene products upregulated in ABA treatment. FDR, False discovery rate by Benjamini-Hochberg multitest correction

| GO term | Description | Background |  | Condition |  | FDR |
| --- | --- | --- | --- | --- | --- | --- |
| GO:0080064 | 4,4-Dimethyl-9beta,19-cyclopropylsterol oxidation | 7/29142 | 0.0% | 2/227 | 0.9% | 3.85E-02 |
| GO:0003999 | Adenine phosphoribosyltransferase activity | 3/29142 | 0.0% | 2/227 | 0.9% | 7.86E-03 |
| GO:0019144 | ADP-sugar diphosphatase activity | 4/29142 | 0.0% | 2/227 | 0.9% | 4.84E-03 |
| GO:0015665 | Alcohol transmembrane transporter activity | 33/29142 | 0.1% | 4/227 | 1.8% | 8.29E-03 |
| GO:0016832 | Aldehyde-lyase activity | 34/29142 | 0.1% | 6/227 | 2.6% | 6.36E-04 |
| GO:1901607 | Alpha-amino acid biosynthetic process | 567/29142 | 1.9% | 11/227 | 4.8% | 1.13E-02 |
| GO:0042886 | Amide transport | 142/29142 | 0.5% | 4/227 | 1.8% | 3.80E-02 |
| GO:0006026 | Aminoglycan catabolic process | 66/29142 | 0.2% | 4/227 | 1.8% | 1.90E-02 |
| GO:0009653 | Anatomical structure morphogenesis | 1717/29142 | 5.9% | 27/227 | 11.9% | 7.00E-03 |
| GO:0048046 | Apoplast | 659/29142 | 2.3% | 24/227 | 10.6% | 1.42E-03 |
| GO:0009073 | Aromatic amino acid family biosynthetic process | 131/29142 | 0.4% | 7/227 | 3.1% | 7.86E-03 |
| GO:0009072 | Aromatic amino acid family metabolic process | 351/29142 | 1.2% | 9/227 | 4.0% | 3.29E-02 |
| GO:0010315 | Auxin efflux | 35/29142 | 0.1% | 5/227 | 2.2% | 1.76E-03 |
| GO:0009341 | Beta-galactosidase complex | 33/29142 | 0.1% | 4/227 | 1.8% | 1.19E-03 |
| GO:0008150 | Biological_process | 22261/29142 | 76.4% | 196/227 | 86.3% | 2.52E-03 |
| GO:0000254 | C-4 methylsterol oxidase activity | 6/29142 | 0.0% | 2/227 | 0.9% | 1.78E-02 |
| GO:0016051 | Carbohydrate biosynthetic process | 1038/29142 | 3.6% | 25/227 | 11.0% | 1.49E-03 |
| GO:1901135 | Carbohydrate derivative metabolic process | 1944/29142 | 6.7% | 41/227 | 18.1% | 2.31E-06 |
| GO:0005975 | Carbohydrate metabolic process | 2109/29142 | 7.2% | 38/227 | 16.7% | 2.34E-04 |
| GO:0015977 | Carbon fixation | 72/29142 | 0.2% | 4/227 | 1.8% | 3.31E-02 |
| GO:0015976 | Carbon utilization | 179/29142 | 0.6% | 11/227 | 4.8% | 7.97E-05 |
| GO:0006081 | Cellular aldehyde metabolic process | 404/29142 | 1.4% | 21/227 | 9.3% | 1.39E-08 |
| GO:0034637 | Cellular carbohydrate biosynthetic process | 495/29142 | 1.7% | 13/227 | 5.7% | 1.48E-02 |
| GO:0044262 | Cellular carbohydrate metabolic process | 1041/29142 | 3.6% | 21/227 | 9.3% | 5.63E-03 |
| GO:0071840 | Cellular component organization or biogenesis | 5164/29142 | 17.7% | 65/227 | 28.6% | 1.18E-02 |
| GO:0044255 | Cellular lipid metabolic process | 1580/29142 | 5.4% | 25/227 | 11.0% | 3.48E-02 |
| GO:0044271 | Cellular nitrogen compound biosynthetic process | 5248/29142 | 18.0% | 60/227 | 26.4% | 4.46E-02 |
| GO:0033692 | Cellular polysaccharide biosynthetic process | 428/29142 | 1.5% | 13/227 | 5.7% | 6.36E-04 |
| GO:0044264 | Cellular polysaccharide metabolic process | 864/29142 | 3.0% | 17/227 | 7.5% | 2.74E-03 |
| GO:0043623 | Cellular protein complex assembly | 527/29142 | 1.8% | 13/227 | 5.7% | 1.68E-02 |
| GO:0005575 | Cellular_component | 26061/29142 | 89.4% | 215/227 | 94.7% | 3.26E-02 |
| GO:0016168 | Chlorophyll binding | 42/29142 | 0.1% | 5/227 | 2.2% | 1.26E-02 |
| GO:0015995 | Chlorophyll biosynthetic process | 126/29142 | 0.4% | 17/227 | 7.5% | 1.70E-12 |
| GO:0009941 | Chloroplast envelope | 1087/29142 | 3.7% | 56/227 | 24.7% | 2.25E-03 |
| GO:0044434 | Chloroplast part | 2121/29142 | 7.3% | 78/227 | 34.4% | 3.47E-11 |
| GO:0009902 | Chloroplast relocation | 70/29142 | 0.2% | 8/227 | 3.5% | 1.72E-03 |
| GO:0009534 | Chloroplast thylakoid | 609/29142 | 2.1% | 41/227 | 18.1% | 1.56E-11 |
| GO:0009535 | Chloroplast thylakoid membrane | 448/29142 | 1.5% | 33/227 | 14.5% | 3.88E-04 |
| GO:0098807 | Chloroplast thylakoid membrane protein complex | 34/29142 | 0.1% | 5/227 | 2.2% | 2.58E-02 |
| GO:0048037 | Cofactor binding | 696/29142 | 2.4% | 14/227 | 6.2% | 1.23E-02 |
| GO:0051188 | Cofactor biosynthetic process | 514/29142 | 1.8% | 23/227 | 10.1% | 1.19E-07 |
| GO:0051186 | Cofactor metabolic process | 1152/29142 | 4.0% | 35/227 | 15.4% | 6.30E-09 |
| GO:0019344 | Cysteine biosynthetic process | 124/29142 | 0.4% | 8/227 | 3.5% | 2.23E-02 |
| GO:0006534 | Cysteine metabolic process | 182/29142 | 0.6% | 8/227 | 3.5% | 1.23E-04 |
| GO:0005737 | Cytoplasm | 16268/29142 | 55.8% | 158/227 | 69.6% | 7.42E-04 |
| GO:0044444 | Cytoplasmic part | 14057/29142 | 48.2% | 148/227 | 65.2% | 1.81E-05 |
| GO:0006952 | Defense response | 2010/29142 | 6.9% | 32/227 | 14.1% | 4.70E-04 |
| GO:0098542 | Defense response to other organism | 1310/29142 | 4.5% | 27/227 | 11.9% | 4.62E-02 |
| GO:0009055 | Electron carrier activity | 237/29142 | 0.8% | 8/227 | 3.5% | 1.08E-02 |
| GO:0031975 | Envelope | 1782/29142 | 6.1% | 58/227 | 25.6% | 1.24E-17 |
| GO:0051656 | Establishment of organelle localization | 111/29142 | 0.4% | 8/227 | 3.5% | 7.42E-04 |
| GO:0051667 | Establishment of plastid localization | 70/29142 | 0.2% | 8/227 | 3.5% | 1.04E-05 |
| GO:0072596 | Establishment of protein localization to chloroplast | 92/29142 | 0.3% | 4/227 | 1.8% | 7.86E-03 |
| GO:0030312 | External encapsulating structure | 987/29142 | 3.4% | 20/227 | 8.8% | 4.14E-03 |
| GO:0005576 | Extracellular region | 2206/29142 | 7.6% | 40/227 | 17.6% | 7.55E-05 |
| GO:0050660 | Flavin adenine dinucleotide binding | 214/29142 | 0.7% | 8/227 | 3.5% | 4.38E-03 |
| GO:0042044 | Fluid transport | 133/29142 | 0.5% | 7/227 | 3.1% | 3.13E-02 |
| GO:0006000 | Fructose metabolic process | 112/29142 | 0.4% | 7/227 | 3.1% | 3.31E-02 |
| GO:0015925 | Galactosidase activity | 43/29142 | 0.1% | 4/227 | 1.8% | 3.85E-02 |
| GO:0006091 | Generation of precursor metabolites and energy | 977/29142 | 3.4% | 31/227 | 13.7% | 2.89E-08 |
| GO:0009250 | Glucan biosynthetic process | 330/29142 | 1.1% | 13/227 | 5.7% | 1.71E-02 |
| GO:0051156 | Glucose 6-phosphate metabolic process | 190/29142 | 0.7% | 11/227 | 4.8% | 2.37E-04 |
| GO:0019682 | Glyceraldehyde-3-phosphate metabolic process | 263/29142 | 0.9% | 21/227 | 9.3% | 2.09E-11 |
| GO:0015168 | Glycerol transmembrane transporter activity | 25/29142 | 0.1% | 4/227 | 1.8% | 7.35E-03 |
| GO:0015793 | Glycerol transport | 25/29142 | 0.1% | 4/227 | 1.8% | 2.64E-03 |
| GO:0006546 | Glycine catabolic process | 23/29142 | 0.1% | 4/227 | 1.8% | 8.29E-03 |
| GO:0019758 | Glycosinolate biosynthetic process | 150/29142 | 0.5% | 9/227 | 4.0% | 1.06E-02 |
| GO:0019757 | Glycosinolate metabolic process | 202/29142 | 0.7% | 9/227 | 4.0% | 1.23E-02 |
| GO:1901659 | Glycosyl compound biosynthetic process | 360/29142 | 1.2% | 15/227 | 6.6% | 2.70E-03 |
| GO:1901657 | Glycosyl compound metabolic process | 1010/29142 | 3.5% | 27/227 | 11.9% | 2.27E-04 |
| GO:0042744 | Hydrogen peroxide catabolic process | 92/29142 | 0.3% | 7/227 | 3.1% | 2.41E-05 |
| GO:0042802 | Identical protein binding | 423/29142 | 1.5% | 11/227 | 4.8% | 8.41E-03 |
| GO:0006020 | Inositol metabolic process | 51/29142 | 0.2% | 6/227 | 2.6% | 4.10E-04 |
| GO:0005779 | Integral component of peroxisomal membrane | 12/29142 | 0.0% | 3/227 | 1.3% | 2.06E-02 |
| GO:0044446 | Intracellular organelle part | 7469/29142 | 25.6% | 103/227 | 45.4% | 3.29E-08 |
| GO:0030522 | Intracellular receptor signaling pathway | 39/29142 | 0.1% | 6/227 | 2.6% | 7.13E-05 |
| GO:0031231 | Intrinsic component of peroxisomal membrane | 12/29142 | 0.0% | 3/227 | 1.3% | 1.88E-02 |
| GO:0009240 | Isopentenyl diphosphate biosynthetic process | 114/29142 | 0.4% | 14/227 | 6.2% | 1.57E-07 |
| GO:0019288 | Isopentenyl diphosphate biosynthetic process, methylerythritol 4-phosphate pathway | 105/29142 | 0.4% | 14/227 | 6.2% | 3.41E-04 |
| GO:0046490 | Isopentenyl diphosphate metabolic process | 114/29142 | 0.4% | 14/227 | 6.2% | 2.45E-08 |
| GO:0008299 | Isoprenoid biosynthetic process | 360/29142 | 1.2% | 15/227 | 6.6% | 7.79E-05 |
| GO:0006720 | Isoprenoid metabolic process | 438/29142 | 1.5% | 15/227 | 6.6% | 8.97E-03 |
| GO:0009965 | Leaf morphogenesis | 239/29142 | 0.8% | 10/227 | 4.4% | 2.07E-02 |
| GO:0051002 | Ligase activity, forming nitrogen-metal bonds | 9/29142 | 0.0% | 3/227 | 1.3% | 7.78E-04 |
| GO:0030076 | Light-harvesting complex | 36/29142 | 0.1% | 3/227 | 1.3% | 3.05E-02 |
| GO:0008610 | Lipid biosynthetic process | 1068/29142 | 3.7% | 21/227 | 9.3% | 3.62E-02 |
| GO:0006629 | Lipid metabolic process | 1870/29142 | 6.4% | 30/227 | 13.2% | 1.06E-02 |
| GO:0009106 | Lipoate metabolic process | 23/29142 | 0.1% | 4/227 | 1.8% | 1.37E-03 |
| GO:0016829 | Lyase activity | 551/29142 | 1.9% | 11/227 | 4.8% | 3.90E-02 |
| GO:0010007 | Magnesium chelatase complex | 10/29142 | 0.0% | 4/227 | 1.8% | 9.62E-04 |
| GO:0046406 | Magnesium protoporphyrin IX methyltransferase activity | 4/29142 | 0.0% | 2/227 | 0.9% | 1.71E-02 |
| GO:0000023 | Maltose metabolic process | 74/29142 | 0.3% | 7/227 | 3.1% | 5.87E-04 |
| GO:0016020 | Membrane | 11376/29142 | 39.0% | 115/227 | 50.7% | 2.24E-02 |
| GO:0061024 | Membrane organization | 614/29142 | 2.1% | 15/227 | 6.6% | 3.25E-02 |
| GO:0098796 | Membrane protein complex | 846/29142 | 2.9% | 16/227 | 7.0% | 3.57E-02 |
| GO:0031903 | Microbody membrane | 62/29142 | 0.2% | 4/227 | 1.8% | 1.26E-02 |
| GO:0044438 | Microbody part | 68/29142 | 0.2% | 4/227 | 1.8% | 3.89E-02 |
| GO:0032787 | Monocarboxylic acid metabolic process | 1547/29142 | 5.3% | 29/227 | 12.8% | 1.17E-03 |
| GO:0016556 | mRNA modification | 78/29142 | 0.3% | 3/227 | 1.3% | 2.39E-02 |
| GO:0010598 | NAD(P)H dehydrogenase complex (plastoquinone) | 7/29142 | 0.0% | 2/227 | 0.9% | 1.61E-02 |
| GO:0006739 | NADP metabolic process | 206/29142 | 0.7% | 12/227 | 5.3% | 3.17E-02 |
| GO:0034660 | ncRNA metabolic process | 655/29142 | 2.2% | 14/227 | 6.2% | 1.75E-03 |
| GO:0034470 | ncRNA processing | 466/29142 | 1.6% | 12/227 | 5.3% | 1.26E-02 |
| GO:0055086 | Nucleobase-containing small molecule metabolic process | 1434/29142 | 4.9% | 25/227 | 11.0% | 9.66E-03 |
| GO:0009116 | Nucleoside metabolic process | 791/29142 | 2.7% | 19/227 | 8.4% | 4.32E-02 |
| GO:0031967 | Organelle envelope | 1782/29142 | 6.1% | 58/227 | 25.6% | 1.19E-17 |
| GO:0051640 | Organelle localization | 207/29142 | 0.7% | 8/227 | 3.5% | 5.86E-03 |
| GO:0044422 | Organelle part | 7478/29142 | 25.7% | 104/227 | 45.8% | 6.28E-08 |
| GO:0031984 | Organelle subcompartment | 1270/29142 | 4.4% | 41/227 | 18.1% | 1.14E-11 |
| GO:1901615 | Organic hydroxy compound metabolic process | 699/29142 | 2.4% | 16/227 | 7.0% | 6.10E-03 |
| GO:1901618 | Organic hydroxy compound transmembrane transporter activity | 38/29142 | 0.1% | 4/227 | 1.8% | 1.32E-02 |
| GO:0015850 | Organic hydroxy compound transport | 50/29142 | 0.2% | 4/227 | 1.8% | 3.00E-02 |
| GO:1901576 | Organic substance biosynthetic process | 8227/29142 | 28.2% | 88/227 | 38.8% | 1.91E-02 |
| GO:1901566 | Organonitrogen compound biosynthetic process | 2727/29142 | 9.4% | 51/227 | 22.5% | 1.01E-05 |
| GO:1901564 | Organonitrogen compound metabolic process | 5468/29142 | 18.8% | 73/227 | 32.2% | 1.90E-04 |
| GO:0090407 | Organophosphate biosynthetic process | 815/29142 | 2.8% | 20/227 | 8.8% | 4.53E-03 |
| GO:0019637 | Organophosphate metabolic process | 1781/29142 | 6.1% | 32/227 | 14.1% | 9.70E-04 |
| GO:0018131 | Oxazole or thiazole biosynthetic process | 7/29142 | 0.0% | 2/227 | 0.9% | 2.36E-02 |
| GO:0046484 | Oxazole or thiazole metabolic process | 7/29142 | 0.0% | 2/227 | 0.9% | 1.88E-02 |
| GO:0044439 | Peroxisomal part | 68/29142 | 0.2% | 4/227 | 1.8% | 3.81E-02 |
| GO:0016559 | Peroxisome fission | 22/29142 | 0.1% | 3/227 | 1.3% | 1.76E-03 |
| GO:0008654 | Phospholipid biosynthetic process | 308/29142 | 1.1% | 14/227 | 6.2% | 5.75E-05 |
| GO:0006644 | Phospholipid metabolic process | 527/29142 | 1.8% | 14/227 | 6.2% | 1.61E-02 |
| GO:0008974 | Phosphoribulokinase activity | 4/29142 | 0.0% | 2/227 | 0.9% | 2.21E-03 |
| GO:0004645 | Phosphorylase activity | 11/29142 | 0.0% | 2/227 | 0.9% | 2.95E-02 |
| GO:0009881 | Photoreceptor activity | 28/29142 | 0.1% | 4/227 | 1.8% | 3.58E-02 |
| GO:0015979 | Photosynthesis | 379/29142 | 1.3% | 36/227 | 15.9% | 1.53E-24 |
| GO:0019685 | Photosynthesis, dark reaction | 35/29142 | 0.1% | 6/227 | 2.6% | 1.61E-02 |
| GO:0019684 | Photosynthesis, light reaction | 243/29142 | 0.8% | 21/227 | 9.3% | 2.89E-03 |
| GO:0034357 | Photosynthetic membrane | 503/29142 | 1.7% | 35/227 | 15.4% | 3.12E-17 |
| GO:0009521 | Photosystem | 95/29142 | 0.3% | 10/227 | 4.4% | 8.29E-03 |
| GO:0010207 | Photosystem II assembly | 65/29142 | 0.2% | 9/227 | 4.0% | 3.51E-03 |
| GO:0031409 | Pigment binding | 20/29142 | 0.1% | 4/227 | 1.8% | 4.40E-04 |
| GO:0046148 | Pigment biosynthetic process | 273/29142 | 0.9% | 19/227 | 8.4% | 2.23E-02 |
| GO:0042440 | Pigment metabolic process | 373/29142 | 1.3% | 19/227 | 8.4% | 1.12E-06 |
| GO:1905392 | Plant organ morphogenesis | 774/29142 | 2.7% | 16/227 | 7.0% | 4.51E-02 |
| GO:0009536 | Plastid | 5694/29142 | 19.5% | 109/227 | 48.0% | 7.14E-20 |
| GO:0009526 | Plastid envelope | 1115/29142 | 3.8% | 56/227 | 24.7% | 3.18E-06 |
| GO:0051644 | Plastid localization | 70/29142 | 0.2% | 8/227 | 3.5% | 2.55E-03 |
| GO:0009668 | Plastid membrane organization | 108/29142 | 0.4% | 10/227 | 4.4% | 4.58E-03 |
| GO:0009657 | Plastid organization | 427/29142 | 1.5% | 18/227 | 7.9% | 5.27E-07 |
| GO:0044435 | Plastid part | 2165/29142 | 7.4% | 79/227 | 34.8% | 2.74E-25 |
| GO:0031976 | Plastid thylakoid | 616/29142 | 2.1% | 41/227 | 18.1% | 1.21E-12 |
| GO:0055035 | Plastid thylakoid membrane | 455/29142 | 1.6% | 33/227 | 14.5% | 5.92E-04 |
| GO:0032544 | Plastid translation | 18/29142 | 0.1% | 4/227 | 1.8% | 3.43E-03 |
| GO:0010287 | Plastoglobule | 92/29142 | 0.3% | 10/227 | 4.4% | 1.72E-02 |
| GO:0000271 | Polysaccharide biosynthetic process | 508/29142 | 1.7% | 15/227 | 6.6% | 8.60E-04 |
| GO:0005976 | Polysaccharide metabolic process | 968/29142 | 3.3% | 19/227 | 8.4% | 4.53E-03 |
| GO:0006779 | Porphyrin-containing compound biosynthetic process | 156/29142 | 0.5% | 17/227 | 7.5% | 6.06E-05 |
| GO:0006778 | Porphyrin-containing compound metabolic process | 255/29142 | 0.9% | 17/227 | 7.5% | 6.27E-03 |
| GO:0009891 | Positive regulation of biosynthetic process | 555/29142 | 1.9% | 13/227 | 5.7% | 3.54E-02 |
| GO:0033240 | Positive regulation of cellular amine metabolic process | 7/29142 | 0.0% | 2/227 | 0.9% | 4.25E-02 |
| GO:0045764 | Positive regulation of cellular amino acid metabolic process | 7/29142 | 0.0% | 2/227 | 0.9% | 1.29E-02 |
| GO:0031328 | Positive regulation of cellular biosynthetic process | 499/29142 | 1.7% | 12/227 | 5.3% | 3.17E-02 |
| GO:0031325 | Positive regulation of cellular metabolic process | 615/29142 | 2.1% | 15/227 | 6.6% | 5.71E-03 |
| GO:0010628 | Positive regulation of gene expression | 473/29142 | 1.6% | 12/227 | 5.3% | 3.13E-03 |
| GO:0010557 | Positive regulation of macromolecule biosynthetic process | 471/29142 | 1.6% | 12/227 | 5.3% | 4.58E-03 |
| GO:0010604 | Positive regulation of macromolecule metabolic process | 587/29142 | 2.0% | 12/227 | 5.3% | 1.72E-02 |
| GO:0009893 | Positive regulation of metabolic process | 703/29142 | 2.4% | 16/227 | 7.0% | 7.69E-03 |
| GO:0051173 | Positive regulation of nitrogen compound metabolic process | 511/29142 | 1.8% | 14/227 | 6.2% | 4.46E-03 |
| GO:1903508 | Positive regulation of nucleic acid-templated transcription | 425/29142 | 1.5% | 12/227 | 5.3% | 1.38E-04 |
| GO:0045935 | Positive regulation of nucleobase-containing compound metabolic process | 460/29142 | 1.6% | 12/227 | 5.3% | 5.56E-03 |
| GO:1902680 | Positive regulation of RNA biosynthetic process | 425/29142 | 1.5% | 12/227 | 5.3% | 1.16E-04 |
| GO:0051254 | Positive regulation of RNA metabolic process | 434/29142 | 1.5% | 12/227 | 5.3% | 5.87E-04 |
| GO:0045893 | Positive regulation of transcription, DNA-templated | 424/29142 | 1.5% | 12/227 | 5.3% | 1.23E-04 |
| GO:0090358 | Positive regulation of tryptophan metabolic process | 6/29142 | 0.0% | 2/227 | 0.9% | 1.61E-02 |
| GO:0046777 | Protein autophosphorylation | 300/29142 | 1.0% | 8/227 | 3.5% | 1.61E-02 |
| GO:0071822 | Protein complex subunit organization | 670/29142 | 2.3% | 13/227 | 5.7% | 1.61E-02 |
| GO:0072598 | Protein localization to chloroplast | 94/29142 | 0.3% | 4/227 | 1.8% | 6.46E-03 |
| GO:0018298 | Protein-chromophore linkage | 46/29142 | 0.2% | 4/227 | 1.8% | 4.84E-03 |
| GO:0010304 | PSII associated light-harvesting complex II catabolic process | 22/29142 | 0.1% | 3/227 | 1.3% | 5.66E-04 |
| GO:0072521 | Purine-containing compound metabolic process | 992/29142 | 3.4% | 18/227 | 7.9% | 4.25E-02 |
| GO:0019362 | Pyridine nucleotide metabolic process | 489/29142 | 1.7% | 15/227 | 6.6% | 4.22E-02 |
| GO:0072524 | Pyridine-containing compound metabolic process | 501/29142 | 1.7% | 15/227 | 6.6% | 1.55E-03 |
| GO:0072528 | Pyrimidine-containing compound biosynthetic process | 149/29142 | 0.5% | 7/227 | 3.1% | 1.68E-02 |
| GO:0006090 | Pyruvate metabolic process | 521/29142 | 1.8% | 23/227 | 10.1% | 2.53E-05 |
| GO:0072593 | Reactive oxygen species metabolic process | 383/29142 | 1.3% | 10/227 | 4.4% | 2.22E-02 |
| GO:0010928 | Regulation of auxin mediated signaling pathway | 54/29142 | 0.2% | 5/227 | 2.2% | 3.72E-04 |
| GO:0065008 | Regulation of biological quality | 1631/29142 | 5.6% | 28/227 | 12.3% | 1.23E-02 |
| GO:0032535 | Regulation of cellular component size | 170/29142 | 0.6% | 7/227 | 3.1% | 1.95E-02 |
| GO:0035303 | Regulation of dephosphorylation | 101/29142 | 0.3% | 7/227 | 3.1% | 2.45E-04 |
| GO:0043269 | Regulation of ion transport | 198/29142 | 0.7% | 7/227 | 3.1% | 4.13E-02 |
| GO:0032879 | Regulation of localization | 292/29142 | 1.0% | 8/227 | 3.5% | 3.15E-02 |
| GO:0035304 | Regulation of protein dephosphorylation | 98/29142 | 0.3% | 7/227 | 3.1% | 7.79E-05 |
| GO:0031399 | Regulation of protein modification process | 310/29142 | 1.1% | 7/227 | 3.1% | 3.67E-02 |
| GO:0010155 | Regulation of proton transport | 65/29142 | 0.2% | 6/227 | 2.6% | 1.71E-02 |
| GO:0090357 | Regulation of tryptophan metabolic process | 7/29142 | 0.0% | 2/227 | 0.9% | 1.72E-02 |
| GO:0009628 | Response to abiotic stimulus | 3515/29142 | 12.1% | 56/227 | 24.7% | 2.58E-02 |
| GO:0009637 | Response to blue light | 170/29142 | 0.6% | 14/227 | 6.2% | 5.41E-03 |
| GO:0051707 | Response to other organism | 1739/29142 | 6.0% | 30/227 | 13.2% | 1.08E-02 |
| GO:0009314 | Response to radiation | 1300/29142 | 4.5% | 39/227 | 17.2% | 2.97E-05 |
| GO:0010114 | Response to red light | 111/29142 | 0.4% | 7/227 | 3.1% | 1.82E-02 |
| GO:0050896 | Response to stimulus | 8706/29142 | 29.9% | 103/227 | 45.4% | 1.80E-03 |
| GO:1902347 | Response to strigolactone | 7/29142 | 0.0% | 2/227 | 0.9% | 2.71E-02 |
| GO:0010266 | Response to vitamin B1 | 5/29142 | 0.0% | 2/227 | 0.9% | 1.24E-02 |
| GO:0048511 | Rhythmic process | 204/29142 | 0.7% | 10/227 | 4.4% | 4.19E-04 |
| GO:0019693 | Ribose phosphate metabolic process | 915/29142 | 3.1% | 19/227 | 8.4% | 3.81E-02 |
| GO:0019843 | rRNA binding | 154/29142 | 0.5% | 8/227 | 3.5% | 1.23E-04 |
| GO:0050278 | Sedoheptulose-bisphosphatase activity | 2/29142 | 0.0% | 1/227 | 0.4% | 3.41E-02 |
| GO:0009070 | Serine family amino acid biosynthetic process | 138/29142 | 0.5% | 8/227 | 3.5% | 7.02E-05 |
| GO:0009071 | Serine family amino acid catabolic process | 27/29142 | 0.1% | 4/227 | 1.8% | 6.36E-04 |
| GO:0016143 | S-glycoside metabolic process | 202/29142 | 0.7% | 9/227 | 4.0% | 4.35E-03 |
| GO:0010016 | Shoot system morphogenesis | 450/29142 | 1.5% | 14/227 | 6.2% | 2.95E-03 |
| GO:0004871 | Signal transducer activity | 381/29142 | 1.3% | 9/227 | 4.0% | 3.81E-02 |
| GO:0044711 | Single-organism biosynthetic process | 3675/29142 | 12.6% | 57/227 | 25.1% | 6.36E-04 |
| GO:0044723 | Single-organism carbohydrate metabolic process | 1686/29142 | 5.8% | 31/227 | 13.7% | 2.07E-02 |
| GO:1902578 | Single-organism localization | 2319/29142 | 8.0% | 35/227 | 15.4% | 3.85E-02 |
| GO:0044802 | Single-organism membrane organization | 509/29142 | 1.7% | 15/227 | 6.6% | 4.45E-03 |
| GO:0044699 | Single-organism process | 14545/29142 | 49.9% | 147/227 | 64.8% | 2.23E-02 |
| GO:0044765 | Single-organism transport | 2274/29142 | 7.8% | 35/227 | 15.4% | 8.29E-03 |
| GO:0019252 | Starch biosynthetic process | 120/29142 | 0.4% | 13/227 | 5.7% | 3.81E-07 |
| GO:0000096 | Sulfur amino acid metabolic process | 299/29142 | 1.0% | 8/227 | 3.5% | 3.53E-02 |
| GO:0044272 | Sulfur compound biosynthetic process | 439/29142 | 1.5% | 18/227 | 7.9% | 5.81E-06 |
| GO:0006790 | Sulfur compound metabolic process | 895/29142 | 3.1% | 19/227 | 8.4% | 5.49E-03 |
| GO:0006949 | Syncytium formation | 20/29142 | 0.1% | 3/227 | 1.3% | 1.29E-02 |
| GO:0046906 | Tetrapyrrole binding | 393/29142 | 1.3% | 9/227 | 4.0% | 2.57E-02 |
| GO:0033014 | Tetrapyrrole biosynthetic process | 159/29142 | 0.5% | 17/227 | 7.5% | 1.29E-10 |
| GO:0033013 | Tetrapyrrole metabolic process | 255/29142 | 0.9% | 17/227 | 7.5% | 2.25E-08 |
| GO:0009229 | Thiamine diphosphate biosynthetic process | 8/29142 | 0.0% | 2/227 | 0.9% | 3.26E-02 |
| GO:0042357 | Thiamine diphosphate metabolic process | 8/29142 | 0.0% | 2/227 | 0.9% | 1.88E-02 |
| GO:0042724 | Thiamine-containing compound biosynthetic process | 17/29142 | 0.1% | 5/227 | 2.2% | 4.41E-05 |
| GO:0042723 | Thiamine-containing compound metabolic process | 20/29142 | 0.1% | 5/227 | 2.2% | 3.43E-05 |
| GO:0052837 | Thiazole biosynthetic process | 7/29142 | 0.0% | 2/227 | 0.9% | 2.58E-02 |
| GO:0052838 | Thiazole metabolic process | 7/29142 | 0.0% | 2/227 | 0.9% | 1.91E-02 |
| GO:0009579 | Thylakoid | 773/29142 | 2.7% | 49/227 | 21.6% | 2.44E-26 |
| GO:0044436 | Thylakoid part | 548/29142 | 1.9% | 39/227 | 17.2% | 1.22E-22 |
| GO:0008762 | UDP-N-acetylmuramate dehydrogenase activity | 13/29142 | 0.0% | 2/227 | 0.9% | 2.56E-02 |
| GO:0006636 | Unsaturated fatty acid biosynthetic process | 42/29142 | 0.1% | 5/227 | 2.2% | 2.07E-02 |
| GO:0033559 | Unsaturated fatty acid metabolic process | 51/29142 | 0.2% | 5/227 | 2.2% | 1.61E-02 |
| GO:0009110 | Vitamin biosynthetic process | 166/29142 | 0.6% | 6/227 | 2.6% | 4.74E-02 |
| GO:0006766 | Vitamin metabolic process | 323/29142 | 1.1% | 10/227 | 4.4% | 4.35E-02 |
| GO:0015250 | Water channel activity | 33/29142 | 0.1% | 5/227 | 2.2% | 7.86E-03 |
| GO:0030104 | Water homeostasis | 31/29142 | 0.1% | 4/227 | 1.8% | 3.10E-02 |
| GO:0005372 | Water transmembrane transporter activity | 33/29142 | 0.1% | 5/227 | 2.2% | 8.42E-04 |

## ABA-down

Table S3.3. Enriched GO categories among the gene products downregulated in ABA treatment. FDR, False discovery rate by Benjamini-Hochberg multitest correction

| GO term | Description | Background |  | Condition |  | FDR |
| --- | --- | --- | --- | --- | --- | --- |
| GO:0004020 | Adenylylsulfate kinase activity | 5/29142 | 0.0% | 3/274 | 1.1% | 1.75E-04 |
| GO:0009820 | Alkaloid metabolic process | 77/29142 | 0.3% | 4/274 | 1.5% | 4.18E-02 |
| GO:0043447 | Alkane biosynthetic process | 7/29142 | 0.0% | 2/274 | 0.7% | 4.68E-02 |
| GO:0043450 | Alkene biosynthetic process | 83/29142 | 0.3% | 5/274 | 1.8% | 4.34E-02 |
| GO:0009072 | Aromatic amino acid family metabolic process | 351/29142 | 1.2% | 11/274 | 4.0% | 7.71E-03 |
| GO:0016887 | ATPase activity | 1017/29142 | 3.5% | 15/274 | 5.5% | 3.26E-02 |
| GO:0043492 | ATPase activity, coupled to movement of substances | 441/29142 | 1.5% | 12/274 | 4.4% | 2.57E-02 |
| GO:0010315 | Auxin efflux | 35/29142 | 0.1% | 4/274 | 1.5% | 6.14E-03 |
| GO:0008150 | Biological_process | 22261/29142 | 76.4% | 236/274 | 86.1% | 2.47E-03 |
| GO:0070588 | Calcium ion transmembrane transport | 53/29142 | 0.2% | 6/274 | 2.2% | 5.21E-03 |
| GO:0005388 | Calcium-transporting ATPase activity | 51/29142 | 0.2% | 6/274 | 2.2% | 5.59E-03 |
| GO:0046394 | Carboxylic acid biosynthetic process | 1292/29142 | 4.4% | 32/274 | 11.7% | 2.89E-03 |
| GO:0043169 | Cation binding | 4446/29142 | 15.3% | 66/274 | 24.1% | 3.67E-03 |
| GO:0043449 | Cellular alkene metabolic process | 83/29142 | 0.3% | 5/274 | 1.8% | 2.34E-02 |
| GO:0044255 | Cellular lipid metabolic process | 1580/29142 | 5.4% | 28/274 | 10.2% | 1.69E-02 |
| GO:0051213 | Dioxygenase activity | 152/29142 | 0.5% | 10/274 | 3.6% | 2.81E-02 |
| GO:0003677 | DNA binding | 2467/29142 | 8.5% | 28/274 | 10.2% | 5.21E-03 |
| GO:1902221 | Erythrose 4-phosphate/phosphoenolpyruvate family amino acid metabolic process | 145/29142 | 0.5% | 7/274 | 2.6% | 1.09E-02 |
| GO:0045229 | External encapsulating structure organization | 662/29142 | 2.3% | 11/274 | 4.0% | 4.37E-03 |
| GO:0051552 | Flavone metabolic process | 22/29142 | 0.1% | 3/274 | 1.1% | 2.81E-02 |
| GO:0007276 | Gamete generation | 136/29142 | 0.5% | 6/274 | 2.2% | 1.95E-02 |
| GO:0009755 | Hormone-mediated signaling pathway | 1121/29142 | 3.8% | 26/274 | 9.5% | 4.14E-02 |
| GO:0070814 | Hydrogen sulfide biosynthetic process | 3/29142 | 0.0% | 2/274 | 0.7% | 2.13E-02 |
| GO:0070813 | Hydrogen sulfide metabolic process | 3/29142 | 0.0% | 2/274 | 0.7% | 1.95E-02 |
| GO:0016820 | Hydrolase activity, acting on acid anhydrides, catalyzing transmembrane movement of substances | 443/29142 | 1.5% | 12/274 | 4.4% | 4.53E-03 |
| GO:0005506 | Iron ion binding | 378/29142 | 1.3% | 15/274 | 5.5% | 4.21E-03 |
| GO:0009694 | Jasmonic acid metabolic process | 126/29142 | 0.4% | 14/274 | 5.1% | 5.42E-05 |
| GO:0008610 | Lipid biosynthetic process | 1068/29142 | 3.7% | 24/274 | 8.8% | 1.76E-02 |
| GO:0006629 | Lipid metabolic process | 1870/29142 | 6.4% | 31/274 | 11.3% | 2.86E-02 |
| GO:0048232 | Male gamete generation | 42/29142 | 0.1% | 6/274 | 2.2% | 1.81E-02 |
| GO:0004478 | Methionine adenosyltransferase activity | 6/29142 | 0.0% | 3/274 | 1.1% | 1.95E-02 |
| GO:0072330 | Monocarboxylic acid biosynthetic process | 701/29142 | 2.4% | 25/274 | 9.1% | 1.01E-02 |
| GO:0032787 | Monocarboxylic acid metabolic process | 1547/29142 | 5.3% | 34/274 | 12.4% | 1.05E-02 |
| GO:0051704 | Multi-organism process | 2367/29142 | 8.1% | 52/274 | 19.0% | 1.07E-04 |
| GO:1900366 | Negative regulation of defense response to insect | 4/29142 | 0.0% | 3/274 | 1.1% | 2.17E-03 |
| GO:1900674 | Olefin biosynthetic process | 148/29142 | 0.5% | 7/274 | 2.6% | 2.86E-02 |
| GO:1900673 | Olefin metabolic process | 167/29142 | 0.6% | 7/274 | 2.6% | 1.95E-02 |
| GO:0006730 | One-carbon metabolic process | 78/29142 | 0.3% | 5/274 | 1.8% | 1.95E-02 |
| GO:0016053 | Organic acid biosynthetic process | 1294/29142 | 4.4% | 32/274 | 11.7% | 3.85E-04 |
| GO:0006082 | Organic acid metabolic process | 3935/29142 | 13.5% | 55/274 | 20.1% | 9.15E-03 |
| GO:0015849 | Organic acid transport | 328/29142 | 1.1% | 8/274 | 2.9% | 3.79E-02 |
| GO:1901576 | Organic substance biosynthetic process | 8227/29142 | 28.2% | 93/274 | 33.9% | 2.57E-02 |
| GO:0055114 | Oxidation-reduction process | 2170/29142 | 7.4% | 48/274 | 17.5% | 1.10E-03 |
| GO:0016491 | Oxidoreductase activity | 2036/29142 | 7.0% | 45/274 | 16.4% | 1.72E-04 |
| GO:0016705 | Oxidoreductase activity, acting on paired donors, with incorporation or reduction of molecular oxygen | 395/29142 | 1.4% | 19/274 | 6.9% | 1.07E-02 |
| GO:0016701 | Oxidoreductase activity, acting on single donors with incorporation of molecular oxygen | 70/29142 | 0.2% | 7/274 | 2.6% | 1.81E-02 |
| GO:0031408 | Oxylipin biosynthetic process | 52/29142 | 0.2% | 7/274 | 2.6% | 3.75E-02 |
| GO:0031407 | Oxylipin metabolic process | 53/29142 | 0.2% | 7/274 | 2.6% | 1.45E-02 |
| GO:0019336 | Phenol-containing compound catabolic process | 6/29142 | 0.0% | 2/274 | 0.7% | 4.14E-02 |
| GO:0009699 | Phenylpropanoid biosynthetic process | 178/29142 | 0.6% | 9/274 | 3.3% | 1.04E-02 |
| GO:0046271 | Phenylpropanoid catabolic process | 6/29142 | 0.0% | 3/274 | 1.1% | 2.47E-03 |
| GO:0009698 | Phenylpropanoid metabolic process | 219/29142 | 0.8% | 10/274 | 3.6% | 5.08E-03 |
| GO:0048235 | Pollen sperm cell differentiation | 19/29142 | 0.1% | 3/274 | 1.1% | 1.80E-02 |
| GO:0072657 | Protein localization to membrane | 304/29142 | 1.0% | 5/274 | 1.8% | 3.91E-02 |
| GO:2000068 | Regulation of defense response to insect | 7/29142 | 0.0% | 3/274 | 1.1% | 1.05E-02 |
| GO:0010468 | Regulation of gene expression | 3554/29142 | 12.2% | 31/274 | 11.3% | 5.78E-03 |
| GO:0010439 | Regulation of glucosinolate biosynthetic process | 7/29142 | 0.0% | 3/274 | 1.1% | 2.25E-03 |
| GO:0051252 | Regulation of RNA metabolic process | 3031/29142 | 10.4% | 33/274 | 12.0% | 1.75E-04 |
| GO:0006355 | Regulation of transcription, DNA-templated | 2868/29142 | 9.8% | 31/274 | 11.3% | 6.76E-03 |
| GO:0000975 | Regulatory region DNA binding | 296/29142 | 1.0% | 12/274 | 4.4% | 2.47E-03 |
| GO:0001067 | Regulatory region nucleic acid binding | 296/29142 | 1.0% | 12/274 | 4.4% | 1.39E-04 |
| GO:0045730 | Respiratory burst | 71/29142 | 0.2% | 5/274 | 1.8% | 3.79E-02 |
| GO:0010200 | Response to chitin | 380/29142 | 1.3% | 23/274 | 8.4% | 1.64E-03 |
| GO:0009719 | Response to endogenous stimulus | 2534/29142 | 8.7% | 67/274 | 24.5% | 1.71E-03 |
| GO:0009620 | Response to fungus | 603/29142 | 2.1% | 25/274 | 9.1% | 2.73E-02 |
| GO:0009753 | Response to jasmonic acid | 480/29142 | 1.6% | 24/274 | 8.8% | 5.57E-03 |
| GO:0080167 | Response to karrikin | 194/29142 | 0.7% | 16/274 | 5.8% | 1.75E-04 |
| GO:0009612 | Response to mechanical stimulus | 61/29142 | 0.2% | 8/274 | 2.9% | 2.25E-03 |
| GO:1901698 | Response to nitrogen compound | 744/29142 | 2.6% | 25/274 | 9.1% | 4.82E-02 |
| GO:0010243 | Response to organonitrogen compound | 431/29142 | 1.5% | 24/274 | 8.8% | 7.27E-04 |
| GO:1901700 | Response to oxygen-containing compound | 2755/29142 | 9.5% | 68/274 | 24.8% | 1.80E-02 |
| GO:0050896 | Response to stimulus | 8706/29142 | 29.9% | 148/274 | 54.0% | 2.67E-10 |
| GO:0006950 | Response to stress | 5087/29142 | 17.5% | 107/274 | 39.1% | 1.04E-02 |
| GO:0009611 | Response to wounding | 460/29142 | 1.6% | 36/274 | 13.1% | 5.61E-10 |
| GO:0032774 | RNA biosynthetic process | 3070/29142 | 10.5% | 31/274 | 11.3% | 4.04E-02 |
| GO:0006556 | S-adenosylmethionine biosynthetic process | 6/29142 | 0.0% | 3/274 | 1.1% | 2.21E-03 |
| GO:0046500 | S-adenosylmethionine metabolic process | 22/29142 | 0.1% | 3/274 | 1.1% | 2.29E-02 |
| GO:0046244 | Salicylic acid catabolic process | 2/29142 | 0.0% | 2/274 | 0.7% | 5.21E-03 |
| GO:0016528 | Sarcoplasm | 51/29142 | 0.2% | 6/274 | 2.2% | 7.65E-04 |
| GO:0016529 | Sarcoplasmic reticulum | 51/29142 | 0.2% | 6/274 | 2.2% | 1.64E-03 |
| GO:0019748 | Secondary metabolic process | 707/29142 | 2.4% | 23/274 | 8.4% | 1.64E-03 |
| GO:0044550 | Secondary metabolite biosynthetic process | 452/29142 | 1.6% | 18/274 | 6.6% | 1.01E-02 |
| GO:0043565 | Sequence-specific DNA binding | 566/29142 | 1.9% | 18/274 | 6.6% | 4.73E-04 |
| GO:0019953 | Sexual reproduction | 224/29142 | 0.8% | 7/274 | 2.6% | 3.79E-02 |
| GO:0007165 | Signal transduction | 2643/29142 | 9.1% | 40/274 | 14.6% | 1.51E-02 |
| GO:0044711 | Single-organism biosynthetic process | 3675/29142 | 12.6% | 59/274 | 21.5% | 1.81E-02 |
| GO:0044763 | Single-organism cellular process | 11381/29142 | 39.1% | 126/274 | 46.0% | 2.56E-02 |
| GO:0044710 | Single-organism metabolic process | 8440/29142 | 29.0% | 106/274 | 38.7% | 2.25E-03 |
| GO:0044283 | Small molecule biosynthetic process | 1807/29142 | 6.2% | 34/274 | 12.4% | 4.79E-02 |
| GO:0000103 | Sulfate assimilation | 36/29142 | 0.1% | 4/274 | 1.5% | 4.14E-02 |
| GO:0046906 | Tetrapyrrole binding | 393/29142 | 1.3% | 12/274 | 4.4% | 5.57E-03 |
| GO:0005667 | Transcription factor complex | 1592/29142 | 5.5% | 26/274 | 9.5% | 2.81E-02 |
| GO:0006351 | Transcription, DNA-templated | 3067/29142 | 10.5% | 31/274 | 11.3% | 1.40E-03 |
| GO:0016765 | Transferase activity, transferring alkyl or aryl (other than methyl) groups | 174/29142 | 0.6% | 7/274 | 2.6% | 1.78E-02 |
| GO:0010025 | Wax biosynthetic process | 40/29142 | 0.1% | 4/274 | 1.5% | 2.56E-02 |
| GO:0010166 | Wax metabolic process | 40/29142 | 0.1% | 4/274 | 1.5% | 1.70E-02 |

## Pyr-up

Table S3.4. Enriched GO categories among the gene products upregulated in Pyr treatment. FDR, False discovery rate by Benjamini-Hochberg multitest correction.

| GO term | Description | Background |  | Condition |  | FDR |
| --- | --- | --- | --- | --- | --- | --- |
| GO:0042446 | Hormone biosynthetic process | 210/29142 | 0.7% | 4/31 | 12.9% | 9.33E-04 |
| GO:0042445 | Hormone metabolic process | 294/29142 | 1.0% | 4/31 | 12.9% | 1.21E-02 |
| GO:0045735 | Nutrient reservoir activity | 53/29142 | 0.2% | 4/31 | 12.9% | 2.06E-04 |
| GO:0006082 | Organic acid metabolic process | 3935/29142 | 13.5% | 9/31 | 29.0% | 4.99E-02 |
| GO:0019825 | Oxygen binding | 166/29142 | 0.6% | 3/31 | 9.7% | 2.96E-02 |
| GO:0009269 | Response to desiccation | 37/29142 | 0.1% | 4/31 | 12.9% | 8.41E-03 |
| GO:0050896 | Response to stimulus | 8706/29142 | 29.9% | 19/31 | 61.3% | 3.29E-02 |
| GO:0044710 | Single-organism metabolic process | 8440/29142 | 29.0% | 15/31 | 48.4% | 1.21E-02 |
| GO:0046906 | Tetrapyrrole binding | 393/29142 | 1.3% | 5/31 | 16.1% | 6.93E-04 |

## Pyr-down

Table S3.6. Enriched GO categories among the gene products downregulated in Pyr treatment. FDR, False discovery rate by Benjamini-Hochberg multitest correction.

| GO term | Description | Background |  | Condition |  | FDR |
| --- | --- | --- | --- | --- | --- | --- |
| GO:0044255 | Cellular lipid metabolic process | 1580/29142 | 5.4% | 4/15 | 26.7% | 3.54E-02 |
| GO:0002213 | Defense response to insect | 35/29142 | 0.1% | 2/15 | 13.3% | 3.00E-02 |
| GO:0010311 | Lateral root formation | 56/29142 | 0.2% | 2/15 | 13.3% | 4.80E-02 |
| GO:1900366 | Negative regulation of defense response to insect | 4/29142 | 0.0% | 2/15 | 13.3% | 1.09E-02 |
| GO:0043901 | Negative regulation of multi-organism process | 16/29142 | 0.1% | 2/15 | 13.3% | 9.00E-03 |
| GO:0002832 | Negative regulation of response to biotic stimulus | 16/29142 | 0.1% | 2/15 | 13.3% | 1.09E-02 |
| GO:0032102 | Negative regulation of response to external stimulus | 16/29142 | 0.1% | 2/15 | 13.3% | 9.00E-03 |
| GO:0016053 | Organic acid biosynthetic process | 1294/29142 | 4.4% | 5/15 | 33.3% | 9.00E-03 |
| GO:0016701 | Oxidoreductase activity, acting on single donors with incorporation of molecular oxygen | 70/29142 | 0.2% | 3/15 | 20.0% | 1.83E-02 |
| GO:2000068 | Regulation of defense response to insect | 7/29142 | 0.0% | 2/15 | 13.3% | 9.00E-03 |
| GO:0043900 | Regulation of multi-organism process | 196/29142 | 0.7% | 2/15 | 13.3% | 4.95E-02 |

## ABA_Pyr-up

Table S3.7. Enriched GO categories among the gene products upregulated in ABA+Pyr treatment. FDR, False discovery rate by Benjamini-Hochberg multitest correction.

| GO term | Description | Background |  | Condition |  | FDR |
| --- | --- | --- | --- | --- | --- | --- |
| GO:0090440 | Abscisic acid transporter activity | 4/29142 | 0.0% | 2/392 | 0.5% | 1.22E-02 |
| GO:0019144 | ADP-sugar diphosphatase activity | 4/29142 | 0.0% | 2/392 | 0.5% | 1.09E-02 |
| GO:0015665 | Alcohol transmembrane transporter activity | 33/29142 | 0.1% | 4/392 | 1.0% | 3.10E-02 |
| GO:0016832 | Aldehyde-lyase activity | 34/29142 | 0.1% | 7/392 | 1.8% | 1.29E-04 |
| GO:1901607 | Alpha-amino acid biosynthetic process | 567/29142 | 1.9% | 16/392 | 4.1% | 1.05E-02 |
| GO:0006026 | Aminoglycan catabolic process | 66/29142 | 0.2% | 5/392 | 1.3% | 2.02E-02 |
| GO:0048046 | Apoplast | 659/29142 | 2.3% | 33/392 | 8.4% | 3.40E-05 |
| GO:0019438 | Aromatic compound biosynthetic process | 4274/29142 | 14.7% | 79/392 | 20.2% | 2.36E-03 |
| GO:0015416 | ATPase-coupled organic phosphonate transmembrane transporter activity | 3/29142 | 0.0% | 2/392 | 0.5% | 1.70E-02 |
| GO:0009341 | Beta-galactosidase complex | 33/29142 | 0.1% | 4/392 | 1.0% | 4.48E-03 |
| GO:0016051 | Carbohydrate biosynthetic process | 1038/29142 | 3.6% | 31/392 | 7.9% | 2.01E-03 |
| GO:1901135 | Carbohydrate derivative metabolic process | 1944/29142 | 6.7% | 50/392 | 12.8% | 5.87E-05 |
| GO:0005975 | Carbohydrate metabolic process | 2109/29142 | 7.2% | 49/392 | 12.5% | 8.37E-04 |
| GO:0019203 | Carbohydrate phosphatase activity | 49/29142 | 0.2% | 3/392 | 0.8% | 2.97E-02 |
| GO:0015977 | Carbon fixation | 72/29142 | 0.2% | 7/392 | 1.8% | 1.31E-03 |
| GO:0015976 | Carbon utilization | 179/29142 | 0.6% | 13/392 | 3.3% | 1.53E-04 |
| GO:0052689 | Carboxylic ester hydrolase activity | 313/29142 | 1.1% | 9/392 | 2.3% | 4.38E-02 |
| GO:0009986 | Cell surface | 35/29142 | 0.1% | 5/392 | 1.3% | 2.36E-03 |
| GO:0006081 | Cellular aldehyde metabolic process | 404/29142 | 1.4% | 22/392 | 5.6% | 3.92E-06 |
| GO:0008652 | Cellular amino acid biosynthetic process | 598/29142 | 2.1% | 19/392 | 4.8% | 3.24E-02 |
| GO:0044262 | Cellular carbohydrate metabolic process | 1041/29142 | 3.6% | 26/392 | 6.6% | 2.02E-02 |
| GO:0044085 | Cellular component biogenesis | 2291/29142 | 7.9% | 50/392 | 12.8% | 1.03E-02 |
| GO:0019725 | Cellular homeostasis | 456/29142 | 1.6% | 16/392 | 4.1% | 1.38E-02 |
| GO:0044271 | Cellular nitrogen compound biosynthetic process | 5248/29142 | 18.0% | 90/392 | 23.0% | 2.36E-03 |
| GO:0043623 | Cellular protein complex assembly | 527/29142 | 1.8% | 19/392 | 4.8% | 1.63E-03 |
| GO:0071214 | Cellular response to abiotic stimulus | 281/29142 | 1.0% | 16/392 | 4.1% | 4.98E-04 |
| GO:0071483 | Cellular response to blue light | 46/29142 | 0.2% | 10/392 | 2.6% | 3.52E-02 |
| GO:0016168 | Chlorophyll binding | 42/29142 | 0.1% | 12/392 | 3.1% | 1.59E-07 |
| GO:0015995 | Chlorophyll biosynthetic process | 126/29142 | 0.4% | 24/392 | 6.1% | 1.09E-17 |
| GO:0009941 | Chloroplast envelope | 1087/29142 | 3.7% | 69/392 | 17.6% | 2.01E-02 |
| GO:0044434 | Chloroplast part | 2121/29142 | 7.3% | 106/392 | 27.0% | 6.56E-14 |
| GO:0030093 | Chloroplast photosystem I | 4/29142 | 0.0% | 3/392 | 0.8% | 1.75E-03 |
| GO:0009902 | Chloroplast relocation | 70/29142 | 0.2% | 12/392 | 3.1% | 1.38E-03 |
| GO:0009534 | Chloroplast thylakoid | 609/29142 | 2.1% | 57/392 | 14.5% | 4.46E-16 |
| GO:0009535 | Chloroplast thylakoid membrane | 448/29142 | 1.5% | 45/392 | 11.5% | 1.32E-05 |
| GO:0098807 | Chloroplast thylakoid membrane protein complex | 34/29142 | 0.1% | 8/392 | 2.0% | 9.77E-04 |
| GO:0048037 | Cofactor binding | 696/29142 | 2.4% | 17/392 | 4.3% | 4.09E-02 |
| GO:0051188 | Cofactor biosynthetic process | 514/29142 | 1.8% | 30/392 | 7.7% | 5.19E-09 |
| GO:0051186 | Cofactor metabolic process | 1152/29142 | 4.0% | 50/392 | 12.8% | 1.09E-11 |
| GO:0019344 | Cysteine biosynthetic process | 124/29142 | 0.4% | 11/392 | 2.8% | 4.95E-02 |
| GO:0006534 | Cysteine metabolic process | 182/29142 | 0.6% | 13/392 | 3.3% | 5.06E-06 |
| GO:0016805 | Dipeptidase activity | 4/29142 | 0.0% | 2/392 | 0.5% | 2.62E-02 |
| GO:0009055 | Electron carrier activity | 237/29142 | 0.8% | 9/392 | 2.3% | 4.09E-02 |
| GO:0022900 | Electron transport chain | 196/29142 | 0.7% | 14/392 | 3.6% | 9.92E-05 |
| GO:0009368 | Endopeptidase Clp complex | 8/29142 | 0.0% | 2/392 | 0.5% | 4.60E-02 |
| GO:1990066 | Energy quenching | 13/29142 | 0.0% | 5/392 | 1.3% | 2.53E-03 |
| GO:0031975 | Envelope | 1782/29142 | 6.1% | 73/392 | 18.6% | 7.59E-15 |
| GO:0051656 | Establishment of organelle localization | 111/29142 | 0.4% | 12/392 | 3.1% | 1.34E-05 |
| GO:0051667 | Establishment of plastid localization | 70/29142 | 0.2% | 12/392 | 3.1% | 1.06E-10 |
| GO:0072596 | Establishment of protein localization to chloroplast | 92/29142 | 0.3% | 4/392 | 1.0% | 7.91E-03 |
| GO:0090150 | Establishment of protein localization to membrane | 304/29142 | 1.0% | 6/392 | 1.5% | 3.04E-02 |
| GO:0030312 | External encapsulating structure | 987/29142 | 3.4% | 27/392 | 6.9% | 9.80E-03 |
| GO:0005576 | Extracellular region | 2206/29142 | 7.6% | 53/392 | 13.5% | 1.48E-03 |
| GO:0010181 | FMN binding | 93/29142 | 0.3% | 6/392 | 1.5% | 5.74E-03 |
| GO:0006000 | Fructose metabolic process | 112/29142 | 0.4% | 10/392 | 2.6% | 8.52E-03 |
| GO:0015928 | Fucosidase activity | 5/29142 | 0.0% | 2/392 | 0.5% | 4.72E-02 |
| GO:0006091 | Generation of precursor metabolites and energy | 977/29142 | 3.4% | 51/392 | 13.0% | 4.49E-15 |
| GO:0045550 | Geranylgeranyl reductase activity | 2/29142 | 0.0% | 2/392 | 0.5% | 5.25E-03 |
| GO:0051156 | Glucose 6-phosphate metabolic process | 190/29142 | 0.7% | 15/392 | 3.8% | 1.45E-05 |
| GO:0019682 | Glyceraldehyde-3-phosphate metabolic process | 263/29142 | 0.9% | 21/392 | 5.4% | 4.82E-08 |
| GO:0015168 | Glycerol transmembrane transporter activity | 25/29142 | 0.1% | 4/392 | 1.0% | 7.29E-03 |
| GO:0015793 | Glycerol transport | 25/29142 | 0.1% | 4/392 | 1.0% | 2.63E-03 |
| GO:0006546 | Glycine catabolic process | 23/29142 | 0.1% | 5/392 | 1.3% | 6.73E-03 |
| GO:0019758 | Glycosinolate biosynthetic process | 150/29142 | 0.5% | 11/392 | 2.8% | 6.14E-03 |
| GO:0019757 | Glycosinolate metabolic process | 202/29142 | 0.7% | 11/392 | 2.8% | 8.72E-03 |
| GO:1901659 | Glycosyl compound biosynthetic process | 360/29142 | 1.2% | 20/392 | 5.1% | 2.73E-04 |
| GO:1901657 | Glycosyl compound metabolic process | 1010/29142 | 3.5% | 35/392 | 8.9% | 2.48E-04 |
| GO:0018130 | Heterocycle biosynthetic process | 4181/29142 | 14.3% | 78/392 | 19.9% | 1.63E-03 |
| GO:0009757 | Hexose mediated signaling | 9/29142 | 0.0% | 2/392 | 0.5% | 3.11E-02 |
| GO:0042744 | Hydrogen peroxide catabolic process | 92/29142 | 0.3% | 5/392 | 1.3% | 2.52E-02 |
| GO:0006818 | Hydrogen transport | 335/29142 | 1.1% | 17/392 | 4.3% | 1.75E-02 |
| GO:0008553 | Hydrogen-exporting ATPase activity, phosphorylative mechanism | 14/29142 | 0.0% | 3/392 | 0.8% | 2.01E-02 |
| GO:0042802 | Identical protein binding | 423/29142 | 1.5% | 14/392 | 3.6% | 4.23E-03 |
| GO:0006020 | Inositol metabolic process | 51/29142 | 0.2% | 7/392 | 1.8% | 3.10E-04 |
| GO:0005779 | Integral component of peroxisomal membrane | 12/29142 | 0.0% | 3/392 | 0.8% | 3.74E-03 |
| GO:0043231 | Intracellular membrane-bounded organelle | 19003/29142 | 65.2% | 283/392 | 72.2% | 3.83E-02 |
| GO:0044446 | Intracellular organelle part | 7469/29142 | 25.6% | 139/392 | 35.5% | 5.81E-04 |
| GO:0030522 | Intracellular receptor signaling pathway | 39/29142 | 0.1% | 9/392 | 2.3% | 4.61E-07 |
| GO:0031231 | Intrinsic component of peroxisomal membrane | 12/29142 | 0.0% | 3/392 | 0.8% | 3.51E-03 |
| GO:0009240 | Isopentenyl diphosphate biosynthetic process | 114/29142 | 0.4% | 11/392 | 2.8% | 4.40E-04 |
| GO:0019288 | Isopentenyl diphosphate biosynthetic process, methylerythritol 4-phosphate pathway | 105/29142 | 0.4% | 11/392 | 2.8% | 2.41E-02 |
| GO:0046490 | Isopentenyl diphosphate metabolic process | 114/29142 | 0.4% | 11/392 | 2.8% | 6.44E-05 |
| GO:0008299 | Isoprenoid biosynthetic process | 360/29142 | 1.2% | 15/392 | 3.8% | 2.33E-03 |
| GO:0006720 | Isoprenoid metabolic process | 438/29142 | 1.5% | 15/392 | 3.8% | 9.09E-03 |
| GO:0009965 | Leaf morphogenesis | 239/29142 | 0.8% | 11/392 | 2.8% | 9.77E-03 |
| GO:0051002 | Ligase activity, forming nitrogen-metal bonds | 9/29142 | 0.0% | 5/392 | 1.3% | 4.52E-07 |
| GO:0030076 | Light-harvesting complex | 36/29142 | 0.1% | 7/392 | 1.8% | 2.54E-05 |
| GO:0008289 | Lipid binding | 418/29142 | 1.4% | 12/392 | 3.1% | 4.33E-02 |
| GO:0010876 | Lipid localization | 266/29142 | 0.9% | 9/392 | 2.3% | 5.28E-03 |
| GO:0009106 | Lipoate metabolic process | 23/29142 | 0.1% | 5/392 | 1.3% | 5.12E-04 |
| GO:0010007 | Magnesium chelatase complex | 10/29142 | 0.0% | 6/392 | 1.5% | 7.50E-06 |
| GO:0046406 | Magnesium protoporphyrin IX methyltransferase activity | 4/29142 | 0.0% | 2/392 | 0.5% | 1.03E-02 |
| GO:0006013 | Mannose metabolic process | 127/29142 | 0.4% | 10/392 | 2.6% | 1.96E-02 |
| GO:0016020 | Membrane | 11376/29142 | 39.0% | 188/392 | 48.0% | 1.09E-02 |
| GO:0098796 | Membrane protein complex | 846/29142 | 2.9% | 28/392 | 7.1% | 2.43E-03 |
| GO:0051540 | Metal cluster binding | 216/29142 | 0.7% | 9/392 | 2.3% | 1.42E-02 |
| GO:0090307 | Mitotic spindle assembly | 2/29142 | 0.0% | 1/392 | 0.3% | 3.99E-02 |
| GO:0060089 | Molecular transducer activity | 208/29142 | 0.7% | 10/392 | 2.6% | 7.01E-03 |
| GO:0015672 | Monovalent inorganic cation transport | 491/29142 | 1.7% | 20/392 | 5.1% | 1.21E-02 |
| GO:0010598 | NAD(P)H dehydrogenase complex (plastoquinone) | 7/29142 | 0.0% | 3/392 | 0.8% | 1.98E-03 |
| GO:0006739 | NADP metabolic process | 206/29142 | 0.7% | 18/392 | 4.6% | 3.11E-03 |
| GO:0006740 | NADPH regeneration | 4/29142 | 0.0% | 3/392 | 0.8% | 1.42E-03 |
| GO:0034660 | ncRNA metabolic process | 655/29142 | 2.2% | 17/392 | 4.3% | 1.49E-02 |
| GO:0034470 | ncRNA processing | 466/29142 | 1.6% | 17/392 | 4.3% | 7.50E-06 |
| GO:0010360 | Negative regulation of anion channel activity | 11/29142 | 0.0% | 4/392 | 1.0% | 3.78E-02 |
| GO:1903960 | Negative regulation of anion transmembrane transport | 11/29142 | 0.0% | 4/392 | 1.0% | 1.18E-03 |
| GO:1903792 | Negative regulation of anion transport | 11/29142 | 0.0% | 4/392 | 1.0% | 3.10E-04 |
| GO:0034766 | Negative regulation of ion transmembrane transport | 11/29142 | 0.0% | 4/392 | 1.0% | 1.63E-03 |
| GO:0032413 | Negative regulation of ion transmembrane transporter activity | 11/29142 | 0.0% | 4/392 | 1.0% | 2.31E-02 |
| GO:0043271 | Negative regulation of ion transport | 19/29142 | 0.1% | 4/392 | 1.0% | 8.06E-03 |
| GO:0034763 | Negative regulation of transmembrane transport | 11/29142 | 0.0% | 4/392 | 1.0% | 7.36E-04 |
| GO:0051051 | Negative regulation of transport | 19/29142 | 0.1% | 4/392 | 1.0% | 2.36E-03 |
| GO:0032410 | Negative regulation of transporter activity | 11/29142 | 0.0% | 4/392 | 1.0% | 2.36E-03 |
| GO:0016151 | Nickel cation binding | 3/29142 | 0.0% | 2/392 | 0.5% | 3.64E-03 |
| GO:0055086 | Nucleobase-containing small molecule metabolic process | 1434/29142 | 4.9% | 37/392 | 9.4% | 1.47E-03 |
| GO:0009163 | Nucleoside biosynthetic process | 181/29142 | 0.6% | 9/392 | 2.3% | 4.93E-02 |
| GO:0009116 | Nucleoside metabolic process | 791/29142 | 2.7% | 26/392 | 6.6% | 2.02E-02 |
| GO:0006753 | Nucleoside phosphate metabolic process | 1197/29142 | 4.1% | 32/392 | 8.2% | 7.43E-03 |
| GO:0046939 | Nucleotide phosphorylation | 370/29142 | 1.3% | 14/392 | 3.6% | 2.97E-02 |
| GO:0031967 | Organelle envelope | 1782/29142 | 6.1% | 73/392 | 18.6% | 4.07E-14 |
| GO:0051640 | Organelle localization | 207/29142 | 0.7% | 12/392 | 3.1% | 3.48E-05 |
| GO:0044422 | Organelle part | 7478/29142 | 25.7% | 140/392 | 35.7% | 6.81E-04 |
| GO:0031984 | Organelle subcompartment | 1270/29142 | 4.4% | 60/392 | 15.3% | 5.79E-14 |
| GO:1901362 | Organic cyclic compound biosynthetic process | 4571/29142 | 15.7% | 82/392 | 20.9% | 2.36E-03 |
| GO:0015850 | Organic hydroxy compound transport | 50/29142 | 0.2% | 6/392 | 1.5% | 4.81E-03 |
| GO:0015604 | Organic phosphonate transmembrane transporter activity | 3/29142 | 0.0% | 2/392 | 0.5% | 1.36E-02 |
| GO:0015716 | Organic phosphonate transport | 3/29142 | 0.0% | 2/392 | 0.5% | 1.22E-02 |
| GO:1901566 | Organonitrogen compound biosynthetic process | 2727/29142 | 9.4% | 65/392 | 16.6% | 6.32E-05 |
| GO:1901564 | Organonitrogen compound metabolic process | 5468/29142 | 18.8% | 103/392 | 26.3% | 1.73E-04 |
| GO:0019637 | Organophosphate metabolic process | 1781/29142 | 6.1% | 42/392 | 10.7% | 2.00E-03 |
| GO:0018131 | Oxazole or thiazole biosynthetic process | 7/29142 | 0.0% | 3/392 | 0.8% | 3.69E-03 |
| GO:0046484 | Oxazole or thiazole metabolic process | 7/29142 | 0.0% | 3/392 | 0.8% | 2.36E-03 |
| GO:0052880 | Oxidoreductase activity, acting on diphenols and related substances as donors, with copper protein as acceptor | 4/29142 | 0.0% | 2/392 | 0.5% | 4.60E-02 |
| GO:0016703 | Oxidoreductase activity, acting on single donors with incorporation of molecular oxygen, incorporation of one atom of oxygen (internal monooxygenases or internal mixed function oxidases) | 5/29142 | 0.0% | 2/392 | 0.5% | 1.96E-02 |
| GO:0006098 | Pentose-phosphate shunt | 188/29142 | 0.6% | 15/392 | 3.8% | 9.63E-03 |
| GO:0016559 | Peroxisome fission | 22/29142 | 0.1% | 3/392 | 0.8% | 5.28E-03 |
| GO:0046471 | Phosphatidylglycerol metabolic process | 32/29142 | 0.1% | 3/392 | 0.8% | 4.95E-02 |
| GO:0008654 | Phospholipid biosynthetic process | 308/29142 | 1.1% | 12/392 | 3.1% | 1.14E-02 |
| GO:0008974 | Phosphoribulokinase activity | 4/29142 | 0.0% | 2/392 | 0.5% | 5.74E-03 |
| GO:0016775 | Phosphotransferase activity, nitrogenous group as acceptor | 36/29142 | 0.1% | 3/392 | 0.8% | 2.84E-02 |
| GO:0009881 | Photoreceptor activity | 28/29142 | 0.1% | 8/392 | 2.0% | 1.45E-04 |
| GO:0015979 | Photosynthesis | 379/29142 | 1.3% | 57/392 | 14.5% | 1.59E-40 |
| GO:0019685 | Photosynthesis, dark reaction | 35/29142 | 0.1% | 8/392 | 2.0% | 6.30E-03 |
| GO:0009765 | Photosynthesis, light harvesting | 52/29142 | 0.2% | 9/392 | 2.3% | 1.14E-02 |
| GO:0019684 | Photosynthesis, light reaction | 243/29142 | 0.8% | 36/392 | 9.2% | 3.04E-06 |
| GO:0034357 | Photosynthetic membrane | 503/29142 | 1.7% | 49/392 | 12.5% | 3.51E-21 |
| GO:0009521 | Photosystem | 95/29142 | 0.3% | 21/392 | 5.4% | 3.57E-09 |
| GO:0010207 | Photosystem II assembly | 65/29142 | 0.2% | 11/392 | 2.8% | 1.52E-02 |
| GO:0009638 | Phototropism | 35/29142 | 0.1% | 8/392 | 2.0% | 2.06E-02 |
| GO:0031409 | Pigment binding | 20/29142 | 0.1% | 6/392 | 1.5% | 5.55E-06 |
| GO:0046148 | Pigment biosynthetic process | 273/29142 | 0.9% | 25/392 | 6.4% | 5.04E-03 |
| GO:0042440 | Pigment metabolic process | 373/29142 | 1.3% | 25/392 | 6.4% | 8.97E-08 |
| GO:1905392 | Plant organ morphogenesis | 774/29142 | 2.7% | 17/392 | 4.3% | 3.01E-02 |
| GO:0009536 | Plastid | 5694/29142 | 19.5% | 153/392 | 39.0% | 3.23E-17 |
| GO:0009526 | Plastid envelope | 1115/29142 | 3.8% | 71/392 | 18.1% | 4.31E-06 |
| GO:0051644 | Plastid localization | 70/29142 | 0.2% | 12/392 | 3.1% | 4.70E-05 |
| GO:0009657 | Plastid organization | 427/29142 | 1.5% | 24/392 | 6.1% | 6.81E-10 |
| GO:0044435 | Plastid part | 2165/29142 | 7.4% | 108/392 | 27.6% | 2.72E-32 |
| GO:0031976 | Plastid thylakoid | 616/29142 | 2.1% | 57/392 | 14.5% | 2.58E-17 |
| GO:0055035 | Plastid thylakoid membrane | 455/29142 | 1.6% | 45/392 | 11.5% | 3.32E-05 |
| GO:0032544 | Plastid translation | 18/29142 | 0.1% | 3/392 | 0.8% | 4.01E-02 |
| GO:0010287 | Plastoglobule | 92/29142 | 0.3% | 23/392 | 5.9% | 2.08E-09 |
| GO:0000271 | Polysaccharide biosynthetic process | 508/29142 | 1.7% | 15/392 | 3.8% | 2.85E-02 |
| GO:0006779 | Porphyrin-containing compound biosynthetic process | 156/29142 | 0.5% | 24/392 | 6.1% | 3.07E-08 |
| GO:0006778 | Porphyrin-containing compound metabolic process | 255/29142 | 0.9% | 24/392 | 6.1% | 8.37E-04 |
| GO:0051094 | Positive regulation of developmental process | 145/29142 | 0.5% | 7/392 | 1.8% | 1.08E-02 |
| GO:0009911 | Positive regulation of flower development | 58/29142 | 0.2% | 5/392 | 1.3% | 1.22E-02 |
| GO:0010628 | Positive regulation of gene expression | 473/29142 | 1.6% | 14/392 | 3.6% | 1.09E-02 |
| GO:0010557 | Positive regulation of macromolecule biosynthetic process | 471/29142 | 1.6% | 14/392 | 3.6% | 1.10E-02 |
| GO:0051240 | Positive regulation of multicellular organismal process | 120/29142 | 0.4% | 7/392 | 1.8% | 3.65E-03 |
| GO:1903508 | Positive regulation of nucleic acid-templated transcription | 425/29142 | 1.5% | 14/392 | 3.6% | 1.21E-02 |
| GO:0045935 | Positive regulation of nucleobase-containing compound metabolic process | 460/29142 | 1.6% | 14/392 | 3.6% | 3.04E-02 |
| GO:0048582 | Positive regulation of post-embryonic development | 95/29142 | 0.3% | 7/392 | 1.8% | 1.32E-03 |
| GO:2000243 | Positive regulation of reproductive process | 71/29142 | 0.2% | 5/392 | 1.3% | 1.06E-02 |
| GO:1902680 | Positive regulation of RNA biosynthetic process | 425/29142 | 1.5% | 14/392 | 3.6% | 1.04E-02 |
| GO:0051254 | Positive regulation of RNA metabolic process | 434/29142 | 1.5% | 14/392 | 3.6% | 5.74E-03 |
| GO:0045893 | Positive regulation of transcription, DNA-templated | 424/29142 | 1.5% | 14/392 | 3.6% | 1.09E-02 |
| GO:1905182 | Positive regulation of urease activity | 2/29142 | 0.0% | 2/392 | 0.5% | 5.90E-03 |
| GO:0046777 | Protein autophosphorylation | 300/29142 | 1.0% | 12/392 | 3.1% | 1.47E-03 |
| GO:0006461 | Protein complex assembly | 616/29142 | 2.1% | 19/392 | 4.8% | 2.34E-02 |
| GO:0031503 | Protein complex localization | 17/29142 | 0.1% | 2/392 | 0.5% | 4.93E-02 |
| GO:0071822 | Protein complex subunit organization | 670/29142 | 2.3% | 19/392 | 4.8% | 6.47E-04 |
| GO:0004673 | Protein histidine kinase activity | 36/29142 | 0.1% | 3/392 | 0.8% | 2.46E-02 |
| GO:0072598 | Protein localization to chloroplast | 94/29142 | 0.3% | 4/392 | 1.0% | 6.49E-03 |
| GO:0018298 | Protein-chromophore linkage | 46/29142 | 0.2% | 8/392 | 2.0% | 3.04E-06 |
| GO:0010304 | PSII associated light-harvesting complex II catabolic process | 22/29142 | 0.1% | 4/392 | 1.0% | 8.37E-04 |
| GO:0072521 | Purine-containing compound metabolic process | 992/29142 | 3.4% | 26/392 | 6.6% | 1.66E-02 |
| GO:0019362 | Pyridine nucleotide metabolic process | 489/29142 | 1.7% | 22/392 | 5.6% | 8.16E-03 |
| GO:0072524 | Pyridine-containing compound metabolic process | 501/29142 | 1.7% | 22/392 | 5.6% | 7.51E-05 |
| GO:0072528 | Pyrimidine-containing compound biosynthetic process | 149/29142 | 0.5% | 9/392 | 2.3% | 9.21E-03 |
| GO:0006090 | Pyruvate metabolic process | 521/29142 | 1.8% | 24/392 | 6.1% | 1.03E-05 |
| GO:0010361 | Regulation of anion channel activity by blue light | 9/29142 | 0.0% | 4/392 | 1.0% | 1.73E-02 |
| GO:0032535 | Regulation of cellular component size | 170/29142 | 0.6% | 8/392 | 2.0% | 3.78E-02 |
| GO:0035303 | Regulation of dephosphorylation | 101/29142 | 0.3% | 10/392 | 2.6% | 6.01E-05 |
| GO:0043467 | Regulation of generation of precursor metabolites and energy | 47/29142 | 0.2% | 5/392 | 1.3% | 2.01E-02 |
| GO:0019220 | Regulation of phosphate metabolic process | 264/29142 | 0.9% | 11/392 | 2.8% | 2.63E-02 |
| GO:0051174 | Regulation of phosphorus metabolic process | 264/29142 | 0.9% | 11/392 | 2.8% | 1.14E-02 |
| GO:0010109 | Regulation of photosynthesis | 60/29142 | 0.2% | 6/392 | 1.5% | 2.22E-02 |
| GO:0035304 | Regulation of protein dephosphorylation | 98/29142 | 0.3% | 10/392 | 2.6% | 3.87E-06 |
| GO:0031399 | Regulation of protein modification process | 310/29142 | 1.1% | 11/392 | 2.8% | 5.18E-03 |
| GO:0031647 | Regulation of protein stability | 18/29142 | 0.1% | 3/392 | 0.8% | 4.87E-02 |
| GO:0010155 | Regulation of proton transport | 65/29142 | 0.2% | 10/392 | 2.6% | 3.83E-04 |
| GO:2001141 | Regulation of RNA biosynthetic process | 2869/29142 | 9.8% | 42/392 | 10.7% | 2.24E-02 |
| GO:0010119 | Regulation of stomatal movement | 126/29142 | 0.4% | 7/392 | 1.8% | 2.02E-02 |
| GO:0006355 | Regulation of transcription, DNA-templated | 2868/29142 | 9.8% | 42/392 | 10.7% | 1.89E-03 |
| GO:1905181 | Regulation of urease activity | 2/29142 | 0.0% | 2/392 | 0.5% | 3.74E-03 |
| GO:0009628 | Response to abiotic stimulus | 3515/29142 | 12.1% | 83/392 | 21.2% | 3.40E-03 |
| GO:0009637 | Response to blue light | 170/29142 | 0.6% | 22/392 | 5.6% | 2.09E-05 |
| GO:0010218 | Response to far red light | 107/29142 | 0.4% | 11/392 | 2.8% | 8.00E-03 |
| GO:0009314 | Response to radiation | 1300/29142 | 4.5% | 54/392 | 13.8% | 7.79E-06 |
| GO:0010114 | Response to red light | 111/29142 | 0.4% | 10/392 | 2.6% | 3.76E-02 |
| GO:0050896 | Response to stimulus | 8706/29142 | 29.9% | 151/392 | 38.5% | 1.20E-02 |
| GO:0010266 | Response to vitamin B1 | 5/29142 | 0.0% | 2/392 | 0.5% | 2.24E-02 |
| GO:0019693 | Ribose phosphate metabolic process | 915/29142 | 3.1% | 27/392 | 6.9% | 1.17E-02 |
| GO:0019843 | rRNA binding | 154/29142 | 0.5% | 6/392 | 1.5% | 1.72E-02 |
| GO:0016072 | rRNA metabolic process | 300/29142 | 1.0% | 15/392 | 3.8% | 5.17E-03 |
| GO:0006364 | rRNA processing | 294/29142 | 1.0% | 15/392 | 3.8% | 1.09E-02 |
| GO:0019748 | Secondary metabolic process | 707/29142 | 2.4% | 21/392 | 5.4% | 4.34E-02 |
| GO:0009070 | Serine family amino acid biosynthetic process | 138/29142 | 0.5% | 12/392 | 3.1% | 4.38E-06 |
| GO:0009071 | Serine family amino acid catabolic process | 27/29142 | 0.1% | 5/392 | 1.3% | 7.09E-04 |
| GO:0070009 | Serine-type aminopeptidase activity | 6/29142 | 0.0% | 2/392 | 0.5% | 2.06E-02 |
| GO:0016143 | S-glycoside metabolic process | 202/29142 | 0.7% | 11/392 | 2.8% | 3.74E-03 |
| GO:0010016 | Shoot system morphogenesis | 450/29142 | 1.5% | 14/392 | 3.6% | 1.38E-02 |
| GO:0004871 | Signal transducer activity | 381/29142 | 1.3% | 17/392 | 4.3% | 4.34E-04 |
| GO:0044711 | Single-organism biosynthetic process | 3675/29142 | 12.6% | 75/392 | 19.1% | 6.90E-03 |
| GO:1902578 | Single-organism localization | 2319/29142 | 8.0% | 55/392 | 14.0% | 5.43E-03 |
| GO:0044765 | Single-organism transport | 2274/29142 | 7.8% | 55/392 | 14.0% | 3.86E-04 |
| GO:0019252 | Starch biosynthetic process | 120/29142 | 0.4% | 9/392 | 2.3% | 3.65E-03 |
| GO:0046524 | Sucrose-phosphate synthase activity | 12/29142 | 0.0% | 2/392 | 0.5% | 3.04E-02 |
| GO:0000096 | Sulfur amino acid metabolic process | 299/29142 | 1.0% | 14/392 | 3.6% | 3.37E-03 |
| GO:0044272 | Sulfur compound biosynthetic process | 439/29142 | 1.5% | 25/392 | 6.4% | 5.47E-08 |
| GO:0006790 | Sulfur compound metabolic process | 895/29142 | 3.1% | 31/392 | 7.9% | 5.53E-05 |
| GO:0046906 | Tetrapyrrole binding | 393/29142 | 1.3% | 17/392 | 4.3% | 1.13E-04 |
| GO:0033014 | Tetrapyrrole biosynthetic process | 159/29142 | 0.5% | 24/392 | 6.1% | 4.09E-15 |
| GO:0033013 | Tetrapyrrole metabolic process | 255/29142 | 0.9% | 24/392 | 6.1% | 1.62E-11 |
| GO:0042357 | Thiamine diphosphate metabolic process | 8/29142 | 0.0% | 2/392 | 0.5% | 4.23E-02 |
| GO:0042724 | Thiamine-containing compound biosynthetic process | 17/29142 | 0.1% | 6/392 | 1.5% | 1.75E-05 |
| GO:0042723 | Thiamine-containing compound metabolic process | 20/29142 | 0.1% | 6/392 | 1.5% | 9.29E-06 |
| GO:0052837 | Thiazole biosynthetic process | 7/29142 | 0.0% | 3/392 | 0.8% | 2.71E-03 |
| GO:0052838 | Thiazole metabolic process | 7/29142 | 0.0% | 3/392 | 0.8% | 2.01E-03 |
| GO:0009579 | Thylakoid | 773/29142 | 2.7% | 70/392 | 17.9% | 6.93E-34 |
| GO:0044436 | Thylakoid part | 548/29142 | 1.9% | 53/392 | 13.5% | 1.75E-26 |
| GO:0009606 | Tropism | 224/29142 | 0.8% | 12/392 | 3.1% | 2.31E-02 |
| GO:0006636 | Unsaturated fatty acid biosynthetic process | 42/29142 | 0.1% | 7/392 | 1.8% | 3.48E-05 |
| GO:0033559 | Unsaturated fatty acid metabolic process | 51/29142 | 0.2% | 7/392 | 1.8% | 4.44E-04 |
| GO:0009110 | Vitamin biosynthetic process | 166/29142 | 0.6% | 9/392 | 2.3% | 8.16E-03 |
| GO:0006766 | Vitamin metabolic process | 323/29142 | 1.1% | 15/392 | 3.8% | 3.65E-03 |
| GO:0015250 | Water channel activity | 33/29142 | 0.1% | 5/392 | 1.3% | 7.91E-03 |
| GO:0005372 | Water transmembrane transporter activity | 33/29142 | 0.1% | 5/392 | 1.3% | 5.17E-03 |

## ABA_Pyr-down

Table S3.7. Enriched GO categories among the gene products downregulated in Pyr treatment. FDR, False discovery rate by Benjamini-Hochberg multitest correction

| GO term | Description | Background |  | Condition |  | FDR |
| --- | --- | --- | --- | --- | --- | --- |
| GO:0016629 | 12-Oxophytodienoate reductase activity | 4/29142 | 0.0% | 3/882 | 0.3% | 1.72E-02 |
| GO:0042218 | 1-Aminocyclopropane-1-carboxylate biosynthetic process | 5/29142 | 0.0% | 3/882 | 0.3% | 2.37E-02 |
| GO:0018871 | 1-Aminocyclopropane-1-carboxylate metabolic process | 6/29142 | 0.0% | 3/882 | 0.3% | 2.34E-02 |
| GO:0009838 | Abscission | 40/29142 | 0.1% | 9/882 | 1.0% | 9.83E-05 |
| GO:0030554 | Adenyl nucleotide binding | 3638/29142 | 12.5% | 99/882 | 11.2% | 3.61E-02 |
| GO:0032559 | Adenyl ribonucleotide binding | 3622/29142 | 12.4% | 99/882 | 11.2% | 2.87E-02 |
| GO:0004020 | Adenylylsulfate kinase activity | 5/29142 | 0.0% | 3/882 | 0.3% | 8.65E-03 |
| GO:0043450 | Alkene biosynthetic process | 83/29142 | 0.3% | 13/882 | 1.5% | 1.26E-04 |
| GO:0031225 | Anchored component of membrane | 186/29142 | 0.6% | 17/882 | 1.9% | 4.42E-02 |
| GO:0046658 | Anchored component of plasma membrane | 108/29142 | 0.4% | 12/882 | 1.4% | 2.56E-02 |
| GO:0009901 | Anther dehiscence | 19/29142 | 0.1% | 8/882 | 0.9% | 3.13E-03 |
| GO:0048653 | Anther development | 62/29142 | 0.2% | 9/882 | 1.0% | 2.26E-03 |
| GO:0009074 | Aromatic amino acid family catabolic process | 63/29142 | 0.2% | 7/882 | 0.8% | 8.82E-03 |
| GO:0019438 | Aromatic compound biosynthetic process | 4274/29142 | 14.7% | 166/882 | 18.8% | 1.03E-04 |
| GO:0005524 | ATP binding | 3463/29142 | 11.9% | 98/882 | 11.1% | 7.92E-03 |
| GO:0016887 | ATPase activity | 1017/29142 | 3.5% | 27/882 | 3.1% | 3.54E-04 |
| GO:0043492 | ATPase activity, coupled to movement of substances | 441/29142 | 1.5% | 20/882 | 2.3% | 4.54E-04 |
| GO:0015662 | ATPase activity, coupled to transmembrane movement of ions, phosphorylative mechanism | 126/29142 | 0.4% | 9/882 | 1.0% | 2.19E-02 |
| GO:0060919 | Auxin influx | 5/29142 | 0.0% | 3/882 | 0.3% | 6.18E-03 |
| GO:0065007 | Biological regulation | 8148/29142 | 28.0% | 321/882 | 36.4% | 2.26E-03 |
| GO:0008150 | Biological_process | 22261/29142 | 76.4% | 740/882 | 83.9% | 2.02E-06 |
| GO:0070588 | Calcium ion transmembrane transport | 53/29142 | 0.2% | 7/882 | 0.8% | 1.87E-02 |
| GO:0005516 | Calmodulin binding | 364/29142 | 1.2% | 24/882 | 2.7% | 1.40E-02 |
| GO:0007154 | Cell communication | 3064/29142 | 10.5% | 148/882 | 16.8% | 3.54E-04 |
| GO:0008219 | Cell death | 535/29142 | 1.8% | 34/882 | 3.9% | 1.62E-02 |
| GO:0071944 | Cell periphery | 5923/29142 | 20.3% | 239/882 | 27.1% | 3.30E-06 |
| GO:0044277 | Cell wall disassembly | 11/29142 | 0.0% | 4/882 | 0.5% | 2.34E-02 |
| GO:0016998 | Cell wall macromolecule catabolic process | 42/29142 | 0.1% | 6/882 | 0.7% | 2.54E-02 |
| GO:0071554 | Cell wall organization or biogenesis | 935/29142 | 3.2% | 49/882 | 5.6% | 6.25E-03 |
| GO:0043449 | Cellular alkene metabolic process | 83/29142 | 0.3% | 13/882 | 1.5% | 1.10E-04 |
| GO:0044262 | Cellular carbohydrate metabolic process | 1041/29142 | 3.6% | 53/882 | 6.0% | 8.95E-03 |
| GO:0034754 | Cellular hormone metabolic process | 149/29142 | 0.5% | 12/882 | 1.4% | 4.17E-02 |
| GO:0044264 | Cellular polysaccharide metabolic process | 864/29142 | 3.0% | 44/882 | 5.0% | 5.44E-03 |
| GO:0006464 | Cellular protein modification process | 4004/29142 | 13.7% | 113/882 | 12.8% | 3.47E-05 |
| GO:0080169 | Cellular response to boron-containing substance deprivation | 2/29142 | 0.0% | 2/882 | 0.2% | 3.31E-02 |
| GO:0036294 | Cellular response to decreased oxygen levels | 31/29142 | 0.1% | 8/882 | 0.9% | 4.17E-02 |
| GO:0071456 | Cellular response to hypoxia | 30/29142 | 0.1% | 8/882 | 0.9% | 1.51E-03 |
| GO:0071453 | Cellular response to oxygen levels | 31/29142 | 0.1% | 8/882 | 0.9% | 8.95E-03 |
| GO:1902170 | Cellular response to reactive nitrogen species | 27/29142 | 0.1% | 3/882 | 0.3% | 4.66E-02 |
| GO:0051716 | Cellular response to stimulus | 4150/29142 | 14.2% | 190/882 | 21.5% | 1.81E-05 |
| GO:0008061 | Chitin binding | 24/29142 | 0.1% | 6/882 | 0.7% | 1.10E-03 |
| GO:0006030 | Chitin metabolic process | 23/29142 | 0.1% | 5/882 | 0.6% | 4.65E-02 |
| GO:0006952 | Defense response | 2010/29142 | 6.9% | 154/882 | 17.5% | 7.22E-03 |
| GO:0009900 | Dehiscence | 23/29142 | 0.1% | 8/882 | 0.9% | 3.90E-06 |
| GO:0005984 | Disaccharide metabolic process | 537/29142 | 1.8% | 35/882 | 4.0% | 9.46E-03 |
| GO:0016102 | Diterpenoid biosynthetic process | 53/29142 | 0.2% | 4/882 | 0.5% | 3.10E-02 |
| GO:0016103 | Diterpenoid catabolic process | 6/29142 | 0.0% | 3/882 | 0.3% | 4.75E-02 |
| GO:0072505 | Divalent inorganic anion homeostasis | 17/29142 | 0.1% | 4/882 | 0.5% | 3.00E-02 |
| GO:0003677 | DNA binding | 2467/29142 | 8.5% | 98/882 | 11.1% | 2.25E-10 |
| GO:0004857 | Enzyme inhibitor activity | 124/29142 | 0.4% | 11/882 | 1.2% | 1.65E-02 |
| GO:0090150 | Establishment of protein localization to membrane | 304/29142 | 1.0% | 19/882 | 2.2% | 1.13E-04 |
| GO:0030312 | External encapsulating structure | 987/29142 | 3.4% | 45/882 | 5.1% | 4.42E-02 |
| GO:0045229 | External encapsulating structure organization | 662/29142 | 2.3% | 37/882 | 4.2% | 3.36E-07 |
| GO:0005576 | Extracellular region | 2206/29142 | 7.6% | 105/882 | 11.9% | 2.28E-04 |
| GO:0010227 | Floral organ abscission | 21/29142 | 0.1% | 4/882 | 0.5% | 2.83E-02 |
| GO:0045487 | Gibberellin catabolic process | 6/29142 | 0.0% | 3/882 | 0.3% | 1.42E-02 |
| GO:0006749 | Glutathione metabolic process | 126/29142 | 0.4% | 9/882 | 1.0% | 3.51E-02 |
| GO:0019757 | Glycosinolate metabolic process | 202/29142 | 0.7% | 16/882 | 1.8% | 3.05E-02 |
| GO:0018130 | Heterocycle biosynthetic process | 4181/29142 | 14.3% | 157/882 | 17.8% | 4.54E-04 |
| GO:0009755 | Hormone-mediated signaling pathway | 1121/29142 | 3.8% | 75/882 | 8.5% | 3.61E-02 |
| GO:0016798 | Hydrolase activity, acting on glycosyl bonds | 538/29142 | 1.8% | 27/882 | 3.1% | 1.27E-02 |
| GO:0002252 | Immune effector process | 233/29142 | 0.8% | 28/882 | 3.2% | 4.25E-02 |
| GO:0002376 | Immune system process | 1025/29142 | 3.5% | 76/882 | 8.6% | 9.47E-09 |
| GO:0043231 | Intracellular membrane-bounded organelle | 19003/29142 | 65.2% | 529/882 | 60.0% | 2.94E-04 |
| GO:0031224 | Intrinsic component of membrane | 5677/29142 | 19.5% | 247/882 | 28.0% | 4.50E-05 |
| GO:0006811 | Ion transport | 1764/29142 | 6.1% | 80/882 | 9.1% | 1.40E-02 |
| GO:0005506 | Iron ion binding | 378/29142 | 1.3% | 34/882 | 3.9% | 1.00E-05 |
| GO:0009695 | Jasmonic acid biosynthetic process | 102/29142 | 0.4% | 21/882 | 2.4% | 4.58E-05 |
| GO:0009694 | Jasmonic acid metabolic process | 126/29142 | 0.4% | 25/882 | 2.8% | 7.43E-10 |
| GO:0016301 | Kinase activity | 2213/29142 | 7.6% | 72/882 | 8.2% | 2.41E-02 |
| GO:0010311 | Lateral root formation | 56/29142 | 0.2% | 7/882 | 0.8% | 2.34E-02 |
| GO:0048232 | Male gamete generation | 42/29142 | 0.1% | 8/882 | 0.9% | 9.09E-03 |
| GO:0016020 | Membrane | 11376/29142 | 39.0% | 396/882 | 44.9% | 1.29E-02 |
| GO:0044425 | Membrane part | 6514/29142 | 22.4% | 257/882 | 29.1% | 1.62E-04 |
| GO:0043227 | Membrane-bounded organelle | 19172/29142 | 65.8% | 532/882 | 60.3% | 8.64E-05 |
| GO:0046873 | Metal ion transmembrane transporter activity | 315/29142 | 1.1% | 16/882 | 1.8% | 1.13E-02 |
| GO:0004478 | Methionine adenosyltransferase activity | 6/29142 | 0.0% | 3/882 | 0.3% | 4.07E-02 |
| GO:0006555 | Methionine metabolic process | 124/29142 | 0.4% | 11/882 | 1.2% | 2.84E-02 |
| GO:0007064 | Mitotic sister chromatid cohesion | 27/29142 | 0.1% | 4/882 | 0.5% | 9.28E-03 |
| GO:0072330 | Monocarboxylic acid biosynthetic process | 701/29142 | 2.4% | 47/882 | 5.3% | 2.77E-04 |
| GO:0004497 | Monooxygenase activity | 270/29142 | 0.9% | 25/882 | 2.8% | 2.31E-02 |
| GO:0055083 | Monovalent inorganic anion homeostasis | 18/29142 | 0.1% | 4/882 | 0.5% | 3.57E-02 |
| GO:0032504 | Multicellular organism reproduction | 345/29142 | 1.2% | 21/882 | 2.4% | 2.50E-02 |
| GO:0048609 | Multicellular organismal reproductive process | 325/29142 | 1.1% | 21/882 | 2.4% | 7.32E-04 |
| GO:0051704 | Multi-organism process | 2367/29142 | 8.1% | 147/882 | 16.7% | 2.45E-11 |
| GO:0051245 | Negative regulation of cellular defense response | 4/29142 | 0.0% | 3/882 | 0.3% | 1.72E-02 |
| GO:1900366 | Negative regulation of defense response to insect | 4/29142 | 0.0% | 3/882 | 0.3% | 1.80E-02 |
| GO:0001071 | Nucleic acid binding transcription factor activity | 1538/29142 | 5.3% | 90/882 | 10.2% | 4.01E-07 |
| GO:0034654 | Nucleobase-containing compound biosynthetic process | 3585/29142 | 12.3% | 132/882 | 15.0% | 4.50E-05 |
| GO:1900674 | Olefin biosynthetic process | 148/29142 | 0.5% | 18/882 | 2.0% | 9.83E-05 |
| GO:1900673 | Olefin metabolic process | 167/29142 | 0.6% | 18/882 | 2.0% | 2.33E-04 |
| GO:0009311 | Oligosaccharide metabolic process | 580/29142 | 2.0% | 35/882 | 4.0% | 9.46E-03 |
| GO:0006730 | One-carbon metabolic process | 78/29142 | 0.3% | 12/882 | 1.4% | 2.50E-04 |
| GO:0016053 | Organic acid biosynthetic process | 1294/29142 | 4.4% | 65/882 | 7.4% | 1.67E-02 |
| GO:0006082 | Organic acid metabolic process | 3935/29142 | 13.5% | 161/882 | 18.3% | 2.13E-03 |
| GO:0015849 | Organic acid transport | 328/29142 | 1.1% | 21/882 | 2.4% | 1.72E-02 |
| GO:0015711 | Organic anion transport | 481/29142 | 1.7% | 29/882 | 3.3% | 1.81E-02 |
| GO:1901362 | Organic cyclic compound biosynthetic process | 4571/29142 | 15.7% | 175/882 | 19.8% | 1.75E-04 |
| GO:0016491 | Oxidoreductase activity | 2036/29142 | 7.0% | 102/882 | 11.6% | 9.67E-05 |
| GO:0016705 | Oxidoreductase activity, acting on paired donors, with incorporation or reduction of molecular oxygen | 395/29142 | 1.4% | 34/882 | 3.9% | 1.13E-02 |
| GO:0016701 | Oxidoreductase activity, acting on single donors with incorporation of molecular oxygen | 70/29142 | 0.2% | 12/882 | 1.4% | 3.79E-03 |
| GO:0019825 | Oxygen binding | 166/29142 | 0.6% | 16/882 | 1.8% | 1.64E-03 |
| GO:0031408 | Oxylipin biosynthetic process | 52/29142 | 0.2% | 12/882 | 1.4% | 1.87E-04 |
| GO:0031407 | Oxylipin metabolic process | 53/29142 | 0.2% | 13/882 | 1.5% | 4.63E-07 |
| GO:0061134 | Peptidase regulator activity | 41/29142 | 0.1% | 6/882 | 0.7% | 3.46E-02 |
| GO:0009699 | Phenylpropanoid biosynthetic process | 178/29142 | 0.6% | 17/882 | 1.9% | 1.65E-02 |
| GO:0046271 | Phenylpropanoid catabolic process | 6/29142 | 0.0% | 3/882 | 0.3% | 1.72E-02 |
| GO:0009698 | Phenylpropanoid metabolic process | 219/29142 | 0.8% | 20/882 | 2.3% | 2.22E-03 |
| GO:0009395 | Phospholipid catabolic process | 130/29142 | 0.4% | 9/882 | 1.0% | 3.82E-02 |
| GO:0016773 | Phosphotransferase activity, alcohol group as acceptor | 1902/29142 | 6.5% | 67/882 | 7.6% | 8.70E-03 |
| GO:0090558 | Plant epidermis development | 611/29142 | 2.1% | 16/882 | 1.8% | 4.50E-02 |
| GO:0005886 | Plasma membrane | 5199/29142 | 17.8% | 211/882 | 23.9% | 1.47E-04 |
| GO:0009555 | Pollen development | 387/29142 | 1.3% | 26/882 | 2.9% | 2.41E-02 |
| GO:0048235 | Pollen sperm cell differentiation | 19/29142 | 0.1% | 4/882 | 0.5% | 2.84E-02 |
| GO:0033037 | Polysaccharide localization | 101/29142 | 0.3% | 10/882 | 1.1% | 6.74E-03 |
| GO:0005976 | Polysaccharide metabolic process | 968/29142 | 3.3% | 52/882 | 5.9% | 2.84E-04 |
| GO:0010628 | Positive regulation of gene expression | 473/29142 | 1.6% | 24/882 | 2.7% | 2.18E-02 |
| GO:0010101 | Post-embryonic root morphogenesis | 80/29142 | 0.3% | 9/882 | 1.0% | 2.44E-02 |
| GO:0043234 | Protein complex | 3742/29142 | 12.8% | 108/882 | 12.2% | 1.96E-03 |
| GO:0072657 | Protein localization to membrane | 304/29142 | 1.0% | 19/882 | 2.2% | 1.90E-05 |
| GO:0036211 | Protein modification process | 4004/29142 | 13.7% | 113/882 | 12.8% | 4.91E-05 |
| GO:0051259 | Protein oligomerization | 50/29142 | 0.2% | 5/882 | 0.6% | 1.29E-02 |
| GO:0006468 | Protein phosphorylation | 1763/29142 | 6.0% | 64/882 | 7.3% | 1.62E-02 |
| GO:0006612 | Protein targeting to membrane | 280/29142 | 1.0% | 19/882 | 2.2% | 1.10E-04 |
| GO:0050789 | Regulation of biological process | 7383/29142 | 25.3% | 296/882 | 33.6% | 1.58E-03 |
| GO:0031326 | Regulation of cellular biosynthetic process | 3472/29142 | 11.9% | 137/882 | 15.5% | 2.24E-02 |
| GO:2000112 | Regulation of cellular macromolecule biosynthetic process | 3380/29142 | 11.6% | 128/882 | 14.5% | 2.20E-04 |
| GO:0050794 | Regulation of cellular process | 6653/29142 | 22.8% | 268/882 | 30.4% | 3.79E-06 |
| GO:0010468 | Regulation of gene expression | 3554/29142 | 12.2% | 134/882 | 15.2% | 2.45E-11 |
| GO:0010439 | Regulation of glucosinolate biosynthetic process | 7/29142 | 0.0% | 3/882 | 0.3% | 3.09E-02 |
| GO:0002682 | Regulation of immune system process | 405/29142 | 1.4% | 30/882 | 3.4% | 2.08E-02 |
| GO:0010556 | Regulation of macromolecule biosynthetic process | 3388/29142 | 11.6% | 128/882 | 14.5% | 4.42E-03 |
| GO:0060255 | Regulation of macromolecule metabolic process | 4037/29142 | 13.9% | 145/882 | 16.4% | 3.69E-02 |
| GO:1903506 | Regulation of nucleic acid-templated transcription | 2869/29142 | 9.8% | 124/882 | 14.1% | 3.13E-02 |
| GO:0019219 | Regulation of nucleobase-containing compound metabolic process | 3130/29142 | 10.7% | 127/882 | 14.4% | 3.95E-07 |
| GO:0080090 | Regulation of primary metabolic process | 4011/29142 | 13.8% | 147/882 | 16.7% | 4.65E-02 |
| GO:0030162 | Regulation of proteolysis | 60/29142 | 0.2% | 5/882 | 0.6% | 3.57E-02 |
| GO:0048583 | Regulation of response to stimulus | 1052/29142 | 3.6% | 64/882 | 7.3% | 3.63E-02 |
| GO:2001141 | Regulation of RNA biosynthetic process | 2869/29142 | 9.8% | 124/882 | 14.1% | 2.96E-03 |
| GO:0051252 | Regulation of RNA metabolic process | 3031/29142 | 10.4% | 126/882 | 14.3% | 1.17E-09 |
| GO:1900376 | Regulation of secondary metabolite biosynthetic process | 29/29142 | 0.1% | 6/882 | 0.7% | 2.66E-02 |
| GO:0006355 | Regulation of transcription, DNA-templated | 2868/29142 | 9.8% | 124/882 | 14.1% | 2.50E-04 |
| GO:0000975 | Regulatory region DNA binding | 296/29142 | 1.0% | 28/882 | 3.2% | 2.28E-04 |
| GO:0001067 | Regulatory region nucleic acid binding | 296/29142 | 1.0% | 28/882 | 3.2% | 8.32E-08 |
| GO:0045730 | Respiratory burst | 71/29142 | 0.2% | 16/882 | 1.8% | 1.20E-06 |
| GO:0002679 | Respiratory burst involved in defense response | 71/29142 | 0.2% | 16/882 | 1.8% | 1.82E-03 |
| GO:0001101 | Response to acid chemical | 2025/29142 | 6.9% | 148/882 | 16.8% | 6.91E-03 |
| GO:0009607 | Response to biotic stimulus | 1761/29142 | 6.0% | 127/882 | 14.4% | 2.20E-04 |
| GO:0042221 | Response to chemical | 4679/29142 | 16.1% | 276/882 | 31.3% | 1.35E-03 |
| GO:0010200 | Response to chitin | 380/29142 | 1.3% | 67/882 | 7.6% | 2.68E-10 |
| GO:0034285 | Response to disaccharide | 173/29142 | 0.6% | 11/882 | 1.2% | 3.80E-02 |
| GO:0009719 | Response to endogenous stimulus | 2534/29142 | 8.7% | 193/882 | 21.9% | 1.54E-09 |
| GO:0009723 | Response to ethylene | 403/29142 | 1.4% | 41/882 | 4.6% | 3.63E-02 |
| GO:0043207 | Response to external biotic stimulus | 1739/29142 | 6.0% | 127/882 | 14.4% | 7.02E-03 |
| GO:0009605 | Response to external stimulus | 2457/29142 | 8.4% | 154/882 | 17.5% | 1.61E-02 |
| GO:0009620 | Response to fungus | 603/29142 | 2.1% | 69/882 | 7.8% | 1.13E-04 |
| GO:0042542 | Response to hydrogen peroxide | 141/29142 | 0.5% | 19/882 | 2.2% | 8.82E-03 |
| GO:0009753 | Response to jasmonic acid | 480/29142 | 1.6% | 58/882 | 6.6% | 3.19E-04 |
| GO:0080167 | Response to karrikin | 194/29142 | 0.7% | 24/882 | 2.7% | 2.77E-03 |
| GO:0009642 | Response to light intensity | 275/29142 | 0.9% | 24/882 | 2.7% | 1.13E-02 |
| GO:1901698 | Response to nitrogen compound | 744/29142 | 2.6% | 80/882 | 9.1% | 1.26E-06 |
| GO:0010033 | Response to organic substance | 3194/29142 | 11.0% | 216/882 | 24.5% | 3.14E-03 |
| GO:0010243 | Response to organonitrogen compound | 431/29142 | 1.5% | 68/882 | 7.7% | 4.37E-10 |
| GO:0051707 | Response to other organism | 1739/29142 | 6.0% | 127/882 | 14.4% | 2.57E-03 |
| GO:0006979 | Response to oxidative stress | 731/29142 | 2.5% | 71/882 | 8.0% | 1.29E-03 |
| GO:1901700 | Response to oxygen-containing compound | 2755/29142 | 9.5% | 208/882 | 23.6% | 3.85E-07 |
| GO:0050896 | Response to stimulus | 8706/29142 | 29.9% | 438/882 | 49.7% | 8.12E-26 |
| GO:0006950 | Response to stress | 5087/29142 | 17.5% | 314/882 | 35.6% | 3.85E-07 |
| GO:0009611 | Response to wounding | 460/29142 | 1.6% | 82/882 | 9.3% | 3.60E-17 |
| GO:0032774 | RNA biosynthetic process | 3070/29142 | 10.5% | 125/882 | 14.2% | 8.39E-11 |
| GO:0006556 | S-adenosylmethionine biosynthetic process | 6/29142 | 0.0% | 3/882 | 0.3% | 1.57E-02 |
| GO:0046500 | S-adenosylmethionine metabolic process | 22/29142 | 0.1% | 5/882 | 0.6% | 1.23E-02 |
| GO:0046244 | Salicylic acid catabolic process | 2/29142 | 0.0% | 2/882 | 0.2% | 2.87E-02 |
| GO:0016528 | Sarcoplasm | 51/29142 | 0.2% | 6/882 | 0.7% | 3.42E-02 |
| GO:0016529 | Sarcoplasmic reticulum | 51/29142 | 0.2% | 6/882 | 0.7% | 4.80E-02 |
| GO:0019748 | Secondary metabolic process | 707/29142 | 2.4% | 65/882 | 7.4% | 7.40E-09 |
| GO:0044550 | Secondary metabolite biosynthetic process | 452/29142 | 1.6% | 42/882 | 4.8% | 4.50E-05 |
| GO:0090487 | Secondary metabolite catabolic process | 147/29142 | 0.5% | 15/882 | 1.7% | 4.64E-02 |
| GO:0043565 | Sequence-specific DNA binding | 566/29142 | 1.9% | 45/882 | 5.1% | 2.22E-05 |
| GO:0016143 | S-glycoside metabolic process | 202/29142 | 0.7% | 16/882 | 1.8% | 3.61E-02 |
| GO:0007165 | Signal transduction | 2643/29142 | 9.1% | 136/882 | 15.4% | 8.08E-08 |
| GO:0023052 | Signaling | 2667/29142 | 9.2% | 136/882 | 15.4% | 1.77E-05 |
| GO:0044700 | Single organism signaling | 2665/29142 | 9.1% | 136/882 | 15.4% | 1.54E-04 |
| GO:0044763 | Single-organism cellular process | 11381/29142 | 39.1% | 404/882 | 45.8% | 2.22E-02 |
| GO:0044710 | Single-organism metabolic process | 8440/29142 | 29.0% | 331/882 | 37.5% | 1.75E-04 |
| GO:0080086 | Stamen filament development | 12/29142 | 0.0% | 4/882 | 0.5% | 7.15E-03 |
| GO:0006790 | Sulfur compound metabolic process | 895/29142 | 3.1% | 46/882 | 5.2% | 1.62E-02 |
| GO:0016115 | Terpenoid catabolic process | 16/29142 | 0.1% | 4/882 | 0.5% | 8.23E-04 |
| GO:0046906 | Tetrapyrrole binding | 393/29142 | 1.3% | 31/882 | 3.5% | 3.95E-05 |
| GO:0009407 | Toxin catabolic process | 147/29142 | 0.5% | 15/882 | 1.7% | 1.49E-03 |
| GO:0009404 | Toxin metabolic process | 177/29142 | 0.6% | 19/882 | 2.2% | 1.94E-04 |
| GO:0005667 | Transcription factor complex | 1592/29142 | 5.5% | 92/882 | 10.4% | 3.75E-09 |
| GO:0006351 | Transcription, DNA-templated | 3067/29142 | 10.5% | 125/882 | 14.2% | 1.31E-10 |
| GO:0072506 | Trivalent inorganic anion homeostasis | 17/29142 | 0.1% | 4/882 | 0.5% | 3.00E-02 |

# Venn GO enrichment

Built from above data using Gennt University Venn utility (accesible in http://bioinformatics.psb.ugent.be/webtools/Venn/)

## Input files:

Table S3.8. Summary of enriched GO annotations

| **List names** | **number of elements** | **number of unique elements** |
| --- | --- | --- |
| ABA-down | 96 | 96 |
| ABA-up | 234 | 234 |
| ABA_Pyr-down | 194 | 194 |
| ABA_Pyr-up | 249 | 249 |
| Pyr-down | 11 | 11 |
| Pyr-up | 9 | 9 |
| **Overall number of unique elements** | | **518** |

## Text results:

Table S3.9. Summary of enriched GO annotations intersections between genes sets up/down-regulated by per treatment.

| **Names** | **total** | **Elements** |
| --- | --- | --- |
| ABA-down ABA-up ABA_Pyr-down ABA_Pyr-up Pyr-up | 2 | GO:0046906 GO:0050896 |
| ABA-down ABA-up Pyr-down | 1 | GO:0044255 |
| ABA-down ABA-up ABA_Pyr-up | 1 | GO:0044711 |
| ABA-down ABA-up ABA_Pyr-down | 1 | GO:0008150 |
| ABA-up ABA_Pyr-down ABA_Pyr-up | 8 | GO:0016020 GO:0016143 GO:0044262 GO:0006790 GO:0010628 GO:0005576 GO:0030312 GO:0019757 |
| ABA-down ABA_Pyr-down Pyr-up | 2 | GO:0044710 GO:0006082 |
| ABA-down ABA_Pyr-down Pyr-down | 3 | GO:1900366 GO:0016701 GO:0016053 |
| ABA-down ABA_Pyr-down ABA_Pyr-up | 2 | GO:0006355 GO:0019748 |
| ABA-down ABA-up | 6 | GO:0006629 GO:0009072 GO:0008610 GO:1901576 GO:0010315 GO:0032787 |
| ABA-up ABA_Pyr-up | 156 | GO:0006020 GO:0009534 GO:0031409 GO:0019144 GO:0051002 GO:0098807 GO:0006091 GO:1901607 GO:0019693 GO:0016559 GO:0031231 GO:0019684 GO:0005975 GO:0019758 GO:0035304 GO:0010598 GO:0010557 GO:0033014 GO:0033559 GO:0051644 GO:0051156 GO:1902680 GO:0042744 GO:0009070 GO:0016168 GO:0009579 GO:0044436 GO:0015850 GO:0042724 GO:0010207 GO:0072524 GO:0019288 GO:0046777 GO:0015977 GO:0019344 GO:0034470 GO:0009526 GO:0019685 GO:1905392 GO:0098796 GO:0006546 GO:0006090 GO:0009536 GO:0042440 GO:0044435 GO:0048037 GO:0044271 GO:1901659 GO:0034660 GO:0031984 GO:0009116 GO:0044272 GO:0042802 GO:0044765 GO:0045893 GO:0009535 GO:0031399 GO:0042357 GO:1902578 GO:0051254 GO:0006779 GO:0051656 GO:0019843 GO:0008974 GO:0010016 GO:0009240 GO:0006739 GO:0009110 GO:0046406 GO:0009941 GO:0004871 GO:0008299 GO:0035303 GO:0009314 GO:0042723 GO:0015979 GO:0072596 GO:0009341 GO:0006636 GO:0051186 GO:0009071 GO:0031975 GO:0033013 GO:0018131 GO:0015168 GO:0016051 GO:0000271 GO:0006778 GO:0019682 GO:0010287 GO:0045935 GO:0009902 GO:0032544 GO:0018298 GO:0032535 GO:0030522 GO:0005779 GO:0009881 GO:0015665 GO:0006000 GO:0051640 GO:0010007 GO:0046490 GO:0019252 GO:0044446 GO:1903508 GO:0019637 GO:0016832 GO:0051188 GO:0044434 GO:0008654 GO:0044422 GO:0000096 GO:0010266 GO:0006534 GO:0051667 GO:0015995 GO:0009521 GO:0072598 GO:0031976 GO:0009965 GO:1901566 GO:0010114 GO:0072528 GO:0043623 GO:0048046 GO:0019362 GO:0005372 GO:0009657 GO:0009106 GO:0072521 GO:0006081 GO:0052837 GO:0015250 GO:0009637 GO:0009628 GO:0015793 GO:0010304 GO:1901135 GO:0006720 GO:0034357 GO:1901564 GO:0046148 GO:0006026 GO:0009055 GO:0071822 GO:0031967 GO:0010155 GO:1901657 GO:0052838 GO:0046484 GO:0006766 GO:0055035 GO:0055086 GO:0030076 GO:0015976 |
| ABA-up ABA_Pyr-down | 4 | GO:0044264 GO:0006952 GO:0051707 GO:0005976 |
| ABA-down Pyr-down | 1 | GO:2000068 |
| ABA-down ABA_Pyr-down | 56 | GO:0080167 GO:0003677 GO:0016705 GO:0031407 GO:0010439 GO:0015849 GO:0009699 GO:0031408 GO:0009611 GO:0007165 GO:0046500 GO:0043450 GO:0006351 GO:0048232 GO:1900673 GO:0043492 GO:0009719 GO:1900674 GO:0048235 GO:0016529 GO:0045229 GO:0043449 GO:0010468 GO:0043565 GO:0016491 GO:0016887 GO:0009698 GO:0051252 GO:0016528 GO:0044763 GO:0051704 GO:0010243 GO:1901698 GO:1901700 GO:0009694 GO:0070588 GO:0006950 GO:0005667 GO:0044550 GO:0045730 GO:0072330 GO:0000975 GO:0001067 GO:0009755 GO:0046271 GO:0009620 GO:0005506 GO:0072657 GO:0010200 GO:0032774 GO:0009753 GO:0004478 GO:0004020 GO:0006556 GO:0006730 GO:0046244 |
| ABA_Pyr-down Pyr-up | 1 | GO:0019825 |
| ABA_Pyr-down Pyr-down | 1 | GO:0010311 |
| ABA_Pyr-down ABA_Pyr-up | 6 | GO:0018130 GO:0043231 GO:0090150 GO:2001141 GO:1901362 GO:0019438 |
| ABA-up | 55 | GO:0098542 GO:0006949 GO:0042886 GO:0030104 GO:0050660 GO:0009653 GO:0006644 GO:0051173 GO:0045764 GO:0044723 GO:0004645 GO:0003999 GO:0000023 GO:0072593 GO:0044802 GO:0009073 GO:0071840 GO:0043269 GO:0050278 GO:0090407 GO:0016829 GO:0000254 GO:0061024 GO:0031328 GO:0044444 GO:0080064 GO:1902347 GO:0010928 GO:0009250 GO:0008762 GO:0005737 GO:0009891 GO:0034637 GO:0044439 GO:0009668 GO:0031903 GO:1901618 GO:0031325 GO:0044699 GO:0009893 GO:0033692 GO:0005575 GO:0065008 GO:0042044 GO:0032879 GO:0090358 GO:0010604 GO:1901615 GO:0009229 GO:0044438 GO:0033240 GO:0090357 GO:0015925 GO:0016556 GO:0048511 |
| ABA-down | 21 | GO:0016765 GO:0055114 GO:0009820 GO:0010025 GO:0009612 GO:0019953 GO:0070814 GO:0051213 GO:0000103 GO:0043169 GO:0007276 GO:0005388 GO:0070813 GO:0044283 GO:0046394 GO:0043447 GO:0019336 GO:0016820 GO:0051552 GO:0010166 GO:1902221 |
| Pyr-up | 4 | GO:0045735 GO:0042446 GO:0009269 GO:0042445 |
| Pyr-down | 5 | GO:0043901 GO:0032102 GO:0043900 GO:0002832 GO:0002213 |
| ABA_Pyr-up | 74 | GO:0070009 GO:0010119 GO:1905182 GO:0034766 GO:0010360 GO:0032413 GO:0016151 GO:0045550 GO:0006740 GO:0009606 GO:0032410 GO:0006818 GO:1903960 GO:0009163 GO:0019203 GO:0016703 GO:0048582 GO:0022900 GO:0009638 GO:0009765 GO:0046524 GO:0010361 GO:0015672 GO:0052880 GO:0004673 GO:0019725 GO:0019220 GO:0071483 GO:1905181 GO:0015416 GO:2000243 GO:0044085 GO:0006098 GO:0051540 GO:0051051 GO:0071214 GO:0010218 GO:0090307 GO:0016775 GO:0034763 GO:0009368 GO:0010876 GO:0031647 GO:0015928 GO:0016805 GO:0043467 GO:0030093 GO:0016072 GO:0008289 GO:1903792 GO:0060089 GO:0009986 GO:0051240 GO:0006364 GO:0008553 GO:0051174 GO:0009911 GO:0051094 GO:0006013 GO:0010181 GO:0006461 GO:0008652 GO:0052689 GO:0046939 GO:0006753 GO:0009757 GO:0031503 GO:1990066 GO:0046471 GO:0015716 GO:0010109 GO:0043271 GO:0015604 GO:0090440 |
| ABA_Pyr-down | 108 | GO:0044700 GO:0009607 GO:0072505 GO:0080090 GO:0034754 GO:2000112 GO:0072506 GO:0016301 GO:0009407 GO:0044277 GO:0009074 GO:0002376 GO:0010033 GO:0007064 GO:0005984 GO:0009395 GO:0006612 GO:0043227 GO:0006030 GO:0008061 GO:0071944 GO:0060255 GO:0004857 GO:0006468 GO:0009311 GO:0071453 GO:0048653 GO:0090487 GO:0061134 GO:0006555 GO:0032559 GO:0009695 GO:0016103 GO:0005516 GO:1903506 GO:0006464 GO:0050794 GO:0051245 GO:0034654 GO:0001101 GO:0018871 GO:0007154 GO:0006979 GO:0009723 GO:1900376 GO:0002252 GO:0019219 GO:0033037 GO:0043207 GO:0044425 GO:0031225 GO:1902170 GO:0065007 GO:0004497 GO:0051716 GO:0005886 GO:0048583 GO:0048609 GO:0016629 GO:0042218 GO:0009404 GO:0055083 GO:0016998 GO:0042542 GO:0071554 GO:0030554 GO:0009642 GO:0009901 GO:0031326 GO:0016102 GO:0023052 GO:0036211 GO:0001071 GO:0090558 GO:0008219 GO:0016773 GO:0016115 GO:0009605 GO:0005524 GO:0009900 GO:0002682 GO:0043234 GO:0031224 GO:0051259 GO:0046873 GO:0034285 GO:0071456 GO:0030162 GO:0046658 GO:0015662 GO:0006811 GO:0016798 GO:0010227 GO:0006749 GO:0009555 GO:0010101 GO:0042221 GO:0050789 GO:0060919 GO:0080086 GO:0009838 GO:0010556 GO:0032504 GO:0015711 GO:0080169 GO:0045487 GO:0002679 GO:0036294 |
