# Supplementary material for: ABA signalling manipulation suppresses senescence of a leafy vegetable stored at room temperature
Source: Plant Biotechnol J. 2017 Aug 16;16(2):530–44. doi: 10.1111/pbi.12793 (PMC5787841; doi:10.1111/pbi.12793)
Supplement: Supplementary file 5 — Table S4 List of DE transcripts annotated with the GO terms ‘GO:0043207 Response to external biotic stimulus’; ‘GO:0000302 ‘Response to reactive oxygen species’; and ‘GO:0016209 Antioxidant activity’. [file PBI-16-530-s005.docx]

## S4 DE transcripts upregulated or downregulated per each treatment and annotated “GO:0043207 Response to external biotic stimulus”; “GO:0000302 Response to reactive oxygen species”; and “GO:0016209 Antioxidant activity”.

## S4.1-6 DE transcripts upregulated or downregulated per each treatment and annotated “GO:0043207 Response to external biotic stimulus”.

### ABA-up

Table S4.1. Upregulated gene products after 8 days of ABA treatment and annotated “GO:0043207 response to external biotic stimulus”. Treat, treatment and change sign. Gene name, as annotated by Blast2GO after best blastx in nr Viridiplantae database. Best Swiss-Prot hit (Uniprot manually annotated and reviewed protein database) as *Arabidopsis thaliana* locus name or Uniprot “Entry|Entry name” if best curated hit is from another species. ABA, Pyr and ABA+Pyr log2 fold change to control condition.

| Treat | Isoform id | Gene name | Best Swiss-Prot hit | ABA | Pyr | ABA+Pyr |
| --- | --- | --- | --- | --- | --- | --- |
| ABA-up | TCONS_00019084 | phosphoribulokinase precursor | at1g32060 | 2.0 | 0.0 | 1.8 |
| ABA-up | TCONS_00011573 | maternal effect embryo arrest 14 | at2g15890 | 1.8 | 0.1 | 2.0 |
| ABA-up | TCONS_00005992 | ATP-dependent protease La domain-containing | at1g75460 | 1.7 | 0.8 | 1.8 |
| ABA-up | TCONS_00019083 | phosphoribulokinase precursor | at1g32060 | 1.7 | 0.4 | 1.8 |
| ABA-up | TCONS_00009167 | NADH dehydrogenase-like complex N | at5g58260 | 1.6 | 0.2 | 1.7 |
| ABA-up | TCONS_00015354 | phosphomethylpyrimidine synthase | at2g29630 | 1.6 | 0.0 | 1.7 |
| ABA-up | TCONS_00020592 | AT3g14310 MLN21 9 | at3g14310 | 1.5 | 0.8 | 1.6 |
| ABA-up | TCONS_00015353 | phosphomethylpyrimidine synthase | at2g29630 | 1.5 | -0.3 | 1.6 |
| ABA-up | TCONS_00034452 | cytochrome b6-f complex iron-sulfur subunit | at4g03280 | 1.4 | 0.4 | 1.1 |
| ABA-up | TCONS_00031881 | chloroplast sedoheptulose-1,7-bisphosphatase | at3g55800 | 1.4 | 0.4 | 1.3 |
| ABA-up | TCONS_00013461 | chaperonin 60 subunit beta 1 | p21241\|rubb_brana | 1.3 | -0.0 | 1.0 |
| ABA-up | TCONS_00005582 | thiazole biosynthetic enzyme | at5g54770 | 1.3 | -0.0 | 1.2 |
| ABA-up | TCONS_00005581 | thiamin biosynthetic enzyme | at5g54770 | 1.2 | -0.2 | 1.0 |
| ABA-up | TCONS_00001932 | crt homolog 1-like | at4g24460 | 1.2 | 0.4 | 1.4 |
| ABA-up | TCONS_00007060 | alpha- glucan phosphorylase l chloroplastic amyloplastic-like | at3g29320 | 1.2 | 0.5 | 1.5 |
| ABA-up | TCONS_00005631 | plastid ribosomal | at4g17560 | 1.2 | 0.3 | 1.7 |
| ABA-up | TCONS_00018113 | cryptochrome partial | at1g04400 | 1.1 | 0.1 | 1.2 |
| ABA-up | TCONS_00009801 | ribulose bisphosphate carboxylase oxygenase activase chloroplastic-like | at2g39730 | 1.0 | 0.3 | 1.0 |
| ABA-up | TCONS_00005170 | glycosyl hydrolase family 3 | at5g20950 | 1.0 | 0.4 | 1.1 |
| ABA-up | TCONS_00005681 | zinc finger CONSTANS-LIKE 6 | at1g68520 | 1.0 | 0.2 | 1.5 |
| ABA-up | TCONS_00032396 | peptidyl-prolyl cis-trans isomerase CYP20-3 | at3g62030 | 1.0 | 0.2 | 1.6 |
| ABA-up | TCONS_00001950 | rna-binding protein cp31 | at4g24770 | 0.9 | 0.3 | 0.9 |
| ABA-up | TCONS_00003659 | 2-cys peroxiredoxin bas1 | at3g11630 | 0.9 | 0.5 | 1.4 |
| ABA-up | TCONS_00007061 | alpha- glucan phosphorylase l chloroplastic amyloplastic-like | at3g29320 | 0.9 | 0.6 | 1.6 |
| ABA-up | TCONS_00003290 | AP2 domain containing | at3g16770 | 0.8 | 0.1 | 1.0 |
| ABA-up | TCONS_00032645 | acetone-cyanohydrin lyase | at2g23600 | 0.8 | 0.4 | 1.2 |
| ABA-up | TCONS_00009632 | peroxidase ATP2a | at2g37130 | 0.7 | 0.3 | 1.3 |
| ABA-up | TCONS_00017007 | peroxidase ATP2a | at2g37130 | 0.7 | 0.0 | 1.4 |
| ABA-up | TCONS_00032646 | acetone-cyanohydrin lyase | at2g23620 | 0.6 | 0.3 | 1.1 |
| ABA-up | TCONS_00027257 | tonoplast intrinsic | at3g26520 | 0.2 | 0.4 | 1.6 |

### ABA-down

Table S4.2. Downregulated gene products after 8 days of ABA treatment and annotated “GO:0043207 response to external biotic stimulus”. Treat, treatment and change sign. Gene name, as annotated by Blast2GO after best blastx in nr Viridiplantae database. Best Swiss-Prot hit (Uniprot manually annotated and reviewed protein database) as *Arabidopsis thaliana* locus name or Uniprot “Entry|Entry name” if best curated hit is from another species. ABA, Pyr and ABA+Pyr log2 fold change to control condition.

| Treat | Isoform id | Gene name | Best Swiss-Prot hit | ABA | Pyr | ABA+Pyr |
| --- | --- | --- | --- | --- | --- | --- |
| ABA-down | TCONS_00003691 | leucoanthocyanidin dioxygenase | at3g11180 | -4.8 | -0.6 | -2.5 |
| ABA-down | TCONS_00003693 | leucoanthocyanidin dioxygenase | at3g11180 | -3.5 | -0.6 | -2.5 |
| ABA-down | TCONS_00000827 | ethylene-responsive transcription factor erf109-like | at4g34410 | -3.3 | -0.6 | -1.6 |
| ABA-down | TCONS_00033664 | glutaredoxin ATGRXS13 | at1g03850 | -3.3 | -0.8 | -1.5 |
| ABA-down | TCONS_00010154 | jasmonic acid-amido synthetase JAR1 | at2g46370 | -3.2 | -0.5 | -4.2 |
| ABA-down | TCONS_00010155 | jasmonic acid-amido synthetase JAR1 | at2g46370 | -3.1 | -0.4 | -5.0 |
| ABA-down | TCONS_00033004 | lipoxygenase 3 | at1g17420 | -3.0 | -1.6 | -2.5 |
| ABA-down | TCONS_00001643 | serine threonine kinase | at4g21390 | -2.9 | -0.6 | -2.5 |
| ABA-down | TCONS_00018728 | AC012189 5EST gb | at1g15010 | -2.9 | -0.8 | -1.9 |
| ABA-down | TCONS_00004528 | 2-oxoglutarate and fe -dependent oxygenase superfamily protein | at5g05600 | -2.5 | -0.2 | -1.9 |
| ABA-down | TCONS_00031019 | lipoxygenase | at1g17420 | -2.5 | -0.3 | -1.3 |
| ABA-down | TCONS_00004527 | 2-oxoglutarate and fe -dependent oxygenase superfamily protein | at5g05600 | -2.3 | -0.3 | -1.8 |
| ABA-down | TCONS_00037492 | NAC domain-containing 102 | at5g08790 | -2.2 | -0.3 | -1.2 |
| ABA-down | TCONS_00011900 | cytochrome P450 | at5g36220 | -2.1 | -0.5 | -2.1 |
| ABA-down | TCONS_00019704 | lipoxygenase 5 | at3g22400 | -2.1 | -0.9 | -1.4 |
| ABA-down | TCONS_00003400 | ethylene-responsive transcription factor erf109-like | at4g34410 | -2.0 | -0.9 | -1.8 |
| ABA-down | TCONS_00019706 | lipoxygenase | at3g22400 | -2.0 | -1.6 | -1.3 |
| ABA-down | TCONS_00019705 | lipoxygenase | at3g22400 | -2.0 | -1.5 | -1.3 |
| ABA-down | TCONS_00036542 | leucine-rich repeat receptor kinase | at5g20480 | -1.9 | -0.5 | -2.1 |
| ABA-down | TCONS_00011614 | heat shock 70 | at5g02500 | -1.9 | -0.4 | -1.8 |
| ABA-down | TCONS_00012827 | phosphatase 2C | at2g30020 | -1.8 | -0.4 | -1.3 |
| ABA-down | TCONS_00013979 | VQ motif-containing | at2g41180 | -1.8 | -0.2 | -1.3 |
| ABA-down | TCONS_00016009 | unnamed protein product | -- | -1.7 | -0.2 | -1.6 |
| ABA-down | TCONS_00005506 | heat shock 81-2 | at5g56030 | -1.7 | -0.3 | -1.4 |
| ABA-down | TCONS_00000487 | 3-deoxy-D-arabino-heptulosonate 7-phosphate synthase 1 | at4g39980 | -1.6 | -0.0 | -1.0 |
| ABA-down | TCONS_00005471 | calcium-transporting ATPase 8 | at4g29900 | -1.5 | -0.4 | -1.7 |
| ABA-down | TCONS_00005470 | calcium-transporting ATPase 8 | at5g57110 | -1.5 | -0.6 | -1.5 |
| ABA-down | TCONS_00015689 | disease resistance | at2g34930 | -1.5 | -0.3 | -1.2 |
| ABA-down | TCONS_00006869 | cytosolic sulfotransferase 12-like | at2g03760 | -1.5 | -0.4 | -1.1 |
| ABA-down | TCONS_00032018 | autoinhibited calcium ATPase | at3g57330 | -1.4 | -0.2 | -1.1 |
| ABA-down | TCONS_00006314 | glutathione s-transferase | at2g02930 | -1.4 | 0.1 | -0.9 |
| ABA-down | TCONS_00002549 | heat shock 70 | at5g02500 | -1.3 | -0.3 | -1.1 |
| ABA-down | TCONS_00002785 | atp sulfurylase | at4g14680 | -1.3 | -0.2 | -1.1 |
| ABA-down | TCONS_00014663 | nodulin family | at2g39210 | -1.2 | -0.5 | -1.5 |
| ABA-down | TCONS_00017135 | nodulin family | at2g39210 | -1.2 | 0.2 | -1.2 |
| ABA-down | TCONS_00005466 | calcium-transporting ATPase 8 | -- | -1.1 | -1.2 | -1.7 |
| ABA-down | TCONS_00007679 | 12-oxophytodienoate reductase 3-like | at2g06050 | -1.1 | -0.5 | -1.3 |
| ABA-down | TCONS_00005214 | CCR4-associated factor 1 | at5g22250 | -1.1 | -0.2 | -1.1 |
| ABA-down | TCONS_00013151 | WRKY transcription factor 11 | at4g31550 | -1.1 | -0.4 | -1.0 |
| ABA-down | TCONS_00023656 | 4-cumarate-COA-ligase | at1g51680 | -1.1 | -0.5 | -0.9 |
| ABA-down | TCONS_00011495 | 12-oxophytodienoate reductase 3 | at2g06050 | -1.0 | -0.1 | -1.0 |
| ABA-down | TCONS_00031895 | zinc finger CCCH domain-containing 29 | at3g55980 | -0.8 | -0.2 | -1.0 |
| ABA-down | TCONS_00027963 | macrophage migration inhibitory factor homolog | at3g51660 | -0.7 | -0.0 | -1.2 |

### Pyr-up

Table S4.3. Upregulated gene products after 8 days of Pyr treatment and annotated “GO:0043207 response to external biotic stimulus”. Treat, treatment and change sign. Gene name, as annotated by Blast2GO after best blastx in nr Viridiplantae database. Best Swiss-Prot hit (Uniprot manually annotated and reviewed protein database) as Arabidopsis thaliana locus name or Uniprot “Entry|Entry name” if best curated hit is from another species. ABA, Pyr and ABA+Pyr log2 fold change to control condition.

| Treat | Isoform id | Gene name | Best Swiss-Prot hit | ABA | Pyr | ABA+Pyr |
| --- | --- | --- | --- | --- | --- | --- |
| Pyr-up | TCONS_00012660 | cytochrome P450 [Arabidopsis thaliana] | o81345\|c79b1_sinal | -1.6 | 2.1 | -0.6 |
| Pyr-up | TCONS_00031664 | glucosyl transferase | at3g53160 | -2.7 | 1.5 | -0.6 |
| Pyr-up | TCONS_00037766 | anthranilate synthase component I-1 | at5g05730 | -1.6 | 1.4 | -0.5 |
| Pyr-up | TCONS_00032012 | beta-1,3-glucanase 2 | at3g57260 | -2.8 | 1.4 | -0.6 |
| Pyr-up | TCONS_00034007 | peroxidase | at5g06730 | -0.2 | 1.2 | 0.1 |

### Pyr-down

Table S4.4. Downregulated gene products after 8 days of Pyr treatment and annotated “GO:0043207 response to external biotic stimulus”. Treat, treatment and change sign. Gene name, as annotated by Blast2GO after best blastx in nr Viridiplantae database. Best Swiss-Prot hit (Uniprot manually annotated and reviewed protein database) as Arabidopsis thaliana locus name or Uniprot “Entry|Entry name” if best curated hit is from another species. ABA, Pyr and ABA+Pyr log2 fold change to control condition.

| Treat | Isoform id | Gene name | Best Swiss-Prot hit | ABA | Pyr | ABA+Pyr |
| --- | --- | --- | --- | --- | --- | --- |
| Pyr-down | TCONS_00019706 | lipoxygenase | at3g22400 | -2.0 | -1.6 | -1.3 |
| Pyr-down | TCONS_00033004 | lipoxygenase 3 | at1g17420 | -3.0 | -1.6 | -2.5 |
| Pyr-down | TCONS_00019705 | lipoxygenase | at3g22400 | -2.0 | -1.5 | -1.3 |

### ABA+Pyr-up

Table S4.5. Upregulated gene products after 8 days of ABA+Pyr treatment and annotated “GO:0043207 response to external biotic stimulus”. Treat, treatment and change sign. Gene name, as annotated by Blast2GO after best blastx in nr Viridiplantae database. Best Swiss-Prot hit (Uniprot manually annotated and reviewed protein database) as Arabidopsis thaliana locus name or Uniprot “Entry|Entry name” if best curated hit is from another species. ABA, Pyr and ABA+Pyr log2 fold change to control condition.

| Treat | Isoform id | Gene name | Best Swiss-Prot hit | ABA | Pyr | ABA+Pyr |
| --- | --- | --- | --- | --- | --- | --- |
| ABA_Pyr-up | TCONS_00011573 | maternal effect embryo arrest 14 | at2g15890 | 1.8 | 0.1 | 2.0 |
| ABA_Pyr-up | TCONS_00005992 | ATP-dependent protease La domain-containing | at1g75460 | 1.7 | 0.8 | 1.8 |
| ABA_Pyr-up | TCONS_00019083 | phosphoribulokinase precursor | at1g32060 | 1.7 | 0.4 | 1.8 |
| ABA_Pyr-up | TCONS_00019084 | phosphoribulokinase precursor | at1g32060 | 2.0 | 0.0 | 1.8 |
| ABA_Pyr-up | TCONS_00009167 | NADH dehydrogenase-like complex N | at5g58260 | 1.6 | 0.2 | 1.7 |
| ABA_Pyr-up | TCONS_00015354 | phosphomethylpyrimidine synthase | at2g29630 | 1.6 | 0.0 | 1.7 |
| ABA_Pyr-up | TCONS_00020592 | AT3g14310 MLN21 9 | at3g14310 | 1.5 | 0.8 | 1.6 |
| ABA_Pyr-up | TCONS_00015353 | phosphomethylpyrimidine synthase | at2g29630 | 1.5 | -0.3 | 1.6 |
| ABA_Pyr-up | TCONS_00006617 | protein | -- | 2.0 | 0.3 | 1.5 |
| ABA_Pyr-up | TCONS_00031881 | chloroplast sedoheptulose-1,7-bisphosphatase | at3g55800 | 1.4 | 0.4 | 1.3 |
| ABA_Pyr-up | TCONS_00009754 | plasma membrane intrinsic 2E | at2g39010 | 1.3 | 1.1 | 1.2 |
| ABA_Pyr-up | TCONS_00018113 | cryptochrome partial | at1g04400 | 1.1 | 0.1 | 1.2 |
| ABA_Pyr-up | TCONS_00005582 | thiazole biosynthetic enzyme | at5g54770 | 1.3 | -0.0 | 1.2 |
| ABA_Pyr-up | TCONS_00022475 | nitrate transporter -like | at1g69850 | 1.7 | 0.7 | 1.1 |
| ABA_Pyr-up | TCONS_00034452 | cytochrome b6-f complex iron-sulfur subunit | at4g03280 | 1.4 | 0.4 | 1.1 |
| ABA_Pyr-up | TCONS_00005170 | glycosyl hydrolase family 3 | at5g20950 | 1.0 | 0.4 | 1.1 |
| ABA_Pyr-up | TCONS_00034297 | AT4g01050 F2N1 31 | at4g01050 | 1.4 | 0.3 | 1.0 |
| ABA_Pyr-up | TCONS_00009801 | ribulose bisphosphate carboxylase oxygenase activase chloroplastic-like | at2g39730 | 1.0 | 0.3 | 1.0 |
| ABA_Pyr-up | TCONS_00005581 | thiamin biosynthetic enzyme | at5g54770 | 1.2 | -0.2 | 1.0 |
| ABA_Pyr-up | TCONS_00013461 | chaperonin 60 subunit beta 1 | p21241\|rubb_brana | 1.3 | -0.0 | 1.0 |
| ABA_Pyr-up | TCONS_00004401 | golden2-like transcription factor | at5g44190 | 1.6 | 1.0 | 0.9 |
| ABA_Pyr-up | TCONS_00034451 | cytochrome b6-f complex iron-sulfur subunit | at4g03280 | 1.3 | 0.3 | 0.9 |
| ABA_Pyr-up | TCONS_00001950 | rna-binding protein cp31 | at4g24770 | 0.9 | 0.3 | 0.9 |
| ABA_Pyr-up | TCONS_00006766 | pcp17c2 | -- | 1.1 | 1.0 | 0.8 |
| ABA_Pyr-up | TCONS_00004692 | peroxisomal nad-malate dehydrogenase 2 | q9xfw3\|mdhg2_brana | 1.2 | 0.1 | 0.8 |
| ABA_Pyr-up | TCONS_00022476 | probable peptide nitrate transporter | at1g69850 | 1.0 | 0.1 | 0.7 |
| ABA_Pyr-up | TCONS_00022422 | probable peptide nitrate transporter | at3g43790 | 1.4 | 0.5 | 0.6 |
| ABA_Pyr-up | TCONS_00022423 | probable peptide nitrate transporter | at3g43790 | 1.1 | 0.4 | 0.5 |
| ABA_Pyr-up | TCONS_00010491 | legume lectin | at3g16530 | 1.3 | 0.4 | 0.0 |
| ABA_Pyr-up | TCONS_00012723 | PHLOEM 2-LIKE A1 | at4g19840 | 1.3 | 0.2 | 0.0 |

### ABA+Pyr-down

Table S4.6. Downregulated gene products after 8 days of ABA+Pyr treatment and annotated “GO:0043207 response to external biotic stimulus”. Treat, treatment and change sign. Gene name, as annotated by Blast2GO after best blastx in nr Viridiplantae database. Best Swiss-Prot hit (Uniprot manually annotated and reviewed protein database) as Arabidopsis thaliana locus name or Uniprot “Entry|Entry name” if best curated hit is from another species. ABA, Pyr and ABA+Pyr log2 fold change to control condition.

| Treat | Isoform id | Gene name | Best Swiss-Prot hit | ABA | Pyr | ABA+Pyr |
| --- | --- | --- | --- | --- | --- | --- |
| ABA_Pyr-down | TCONS_00010155 | jasmonic acid-amido synthetase JAR1 | at2g46370 | -3.1 | -0.4 | -5.0 |
| ABA_Pyr-down | TCONS_00010154 | jasmonic acid-amido synthetase JAR1 | at2g46370 | -3.2 | -0.5 | -4.2 |
| ABA_Pyr-down | TCONS_00003691 | leucoanthocyanidin dioxygenase | at3g11180 | -4.8 | -0.6 | -2.5 |
| ABA_Pyr-down | TCONS_00033004 | lipoxygenase 3 | at1g17420 | -3.0 | -1.6 | -2.5 |
| ABA_Pyr-down | TCONS_00001643 | serine threonine kinase | at4g21390 | -2.9 | -0.6 | -2.5 |
| ABA_Pyr-down | TCONS_00018335 | phosphatase 2C | at1g07160 | -Inf | -2.6 | -2.4 |
| ABA_Pyr-down | TCONS_00011900 | cytochrome P450 | at5g36220 | -2.1 | -0.5 | -2.1 |
| ABA_Pyr-down | TCONS_00036542 | leucine-rich repeat receptor kinase | at5g20480 | -1.9 | -0.5 | -2.1 |
| ABA_Pyr-down | TCONS_00033871 | allene oxide cyclase | at3g25780 | -2.9 | -0.7 | -1.9 |
| ABA_Pyr-down | TCONS_00018728 | AC012189 5EST gb | at1g15010 | -2.9 | -0.8 | -1.9 |
| ABA_Pyr-down | TCONS_00004528 | 2-oxoglutarate and fe -dependent oxygenase superfamily protein | at5g05600 | -2.5 | -0.2 | -1.9 |
| ABA_Pyr-down | TCONS_00004527 | 2-oxoglutarate and fe -dependent oxygenase superfamily protein | at5g05600 | -2.3 | -0.3 | -1.8 |
| ABA_Pyr-down | TCONS_00003400 | ethylene-responsive transcription factor erf109-like | at4g34410 | -2.0 | -0.9 | -1.8 |
| ABA_Pyr-down | TCONS_00011614 | heat shock 70 | at5g02500 | -1.9 | -0.4 | -1.8 |
| ABA_Pyr-down | TCONS_00005471 | calcium-transporting ATPase 8 | at4g29900 | -1.5 | -0.4 | -1.7 |
| ABA_Pyr-down | TCONS_00000827 | ethylene-responsive transcription factor erf109-like | at4g34410 | -3.3 | -0.6 | -1.6 |
| ABA_Pyr-down | TCONS_00016009 | unnamed protein product | -- | -1.7 | -0.2 | -1.6 |
| ABA_Pyr-down | TCONS_00033664 | glutaredoxin ATGRXS13 | at1g03850 | -3.3 | -0.8 | -1.5 |
| ABA_Pyr-down | TCONS_00005470 | calcium-transporting ATPase 8 | at5g57110 | -1.5 | -0.6 | -1.5 |
| ABA_Pyr-down | TCONS_00014663 | nodulin family | at2g39210 | -1.2 | -0.5 | -1.5 |
| ABA_Pyr-down | TCONS_00019704 | lipoxygenase 5 | at3g22400 | -2.1 | -0.9 | -1.4 |
| ABA_Pyr-down | TCONS_00005506 | heat shock 81-2 | at5g56030 | -1.7 | -0.3 | -1.4 |
| ABA_Pyr-down | TCONS_00031019 | lipoxygenase | at1g17420 | -2.5 | -0.3 | -1.3 |
| ABA_Pyr-down | TCONS_00019705 | lipoxygenase | at3g22400 | -2.0 | -1.5 | -1.3 |
| ABA_Pyr-down | TCONS_00019706 | lipoxygenase | at3g22400 | -2.0 | -1.6 | -1.3 |
| ABA_Pyr-down | TCONS_00012827 | phosphatase 2C | at2g30020 | -1.8 | -0.4 | -1.3 |
| ABA_Pyr-down | TCONS_00013979 | VQ motif-containing | at2g41180 | -1.8 | -0.2 | -1.3 |
| ABA_Pyr-down | TCONS_00007679 | 12-oxophytodienoate reductase 3-like | at2g06050 | -1.1 | -0.5 | -1.3 |
| ABA_Pyr-down | TCONS_00037492 | NAC domain-containing 102 | at5g08790 | -2.2 | -0.3 | -1.2 |
| ABA_Pyr-down | TCONS_00017154 | PBS1-like 1 kinase | at2g39660 | -2.1 | -0.9 | -1.2 |
| ABA_Pyr-down | TCONS_00007721 | ABC transporter | at3g47780 | -1.9 | 0.0 | -1.2 |
| ABA_Pyr-down | TCONS_00015689 | disease resistance | at2g34930 | -1.5 | -0.3 | -1.2 |
| ABA_Pyr-down | TCONS_00017135 | nodulin family | at2g39210 | -1.2 | 0.2 | -1.2 |
| ABA_Pyr-down | TCONS_00009713 | WRKY transcription factor 33 | at2g38470 | -2.2 | -0.3 | -1.1 |
| ABA_Pyr-down | TCONS_00011523 | AGD2-like defense response 1 | at2g13810 | -2.2 | 0.6 | -1.1 |
| ABA_Pyr-down | TCONS_00010910 | 70 kDa heat shock | at3g12580 | -1.8 | -0.4 | -1.1 |
| ABA_Pyr-down | TCONS_00031016 | lipoxygenase | at1g17420 | -1.8 | -0.7 | -1.1 |
| ABA_Pyr-down | TCONS_00006869 | cytosolic sulfotransferase 12-like | at2g03760 | -1.5 | -0.4 | -1.1 |
| ABA_Pyr-down | TCONS_00032018 | autoinhibited calcium ATPase | at3g57330 | -1.4 | -0.2 | -1.1 |
| ABA_Pyr-down | TCONS_00002549 | heat shock 70 | at5g02500 | -1.3 | -0.3 | -1.1 |
| ABA_Pyr-down | TCONS_00002785 | atp sulfurylase | at4g14680 | -1.3 | -0.2 | -1.1 |
| ABA_Pyr-down | TCONS_00005214 | CCR4-associated factor 1 | at5g22250 | -1.1 | -0.2 | -1.1 |
| ABA_Pyr-down | TCONS_00000989 | calmodulin-binding protein | at4g33050 | -3.4 | 0.3 | -1.0 |
| ABA_Pyr-down | TCONS_00036563 | blue copper binding | at5g20230 | -2.6 | -0.2 | -1.0 |
| ABA_Pyr-down | TCONS_00009797 | PBS1-like 1 kinase | at2g39660 | -2.4 | 0.1 | -1.0 |
| ABA_Pyr-down | TCONS_00036564 | blue copper binding | at5g20230 | -2.4 | -0.3 | -1.0 |
| ABA_Pyr-down | TCONS_00007319 | Cam-binding 60-like G | at5g26920 | -2.0 | -0.4 | -1.0 |
| ABA_Pyr-down | TCONS_00004031 | ATAF2 | at5g08790 | -1.8 | -0.0 | -1.0 |
| ABA_Pyr-down | TCONS_00003593 | protein fyd | at3g12570 | -1.8 | -0.3 | -1.0 |
| ABA_Pyr-down | TCONS_00000487 | 3-deoxy-D-arabino-heptulosonate 7-phosphate synthase 1 | at4g39980 | -1.6 | -0.0 | -1.0 |
| ABA_Pyr-down | TCONS_00019011 | subtilase family | at1g32940 | -1.4 | 0.2 | -1.0 |
| ABA_Pyr-down | TCONS_00030017 | cytochrome P450 monooxygenase | at4g31500 | -1.3 | -0.0 | -1.0 |
| ABA_Pyr-down | TCONS_00013151 | WRKY transcription factor 11 | at4g31550 | -1.1 | -0.4 | -1.0 |
| ABA_Pyr-down | TCONS_00011495 | 12-oxophytodienoate reductase 3 | at2g06050 | -1.0 | -0.1 | -1.0 |
| ABA_Pyr-down | TCONS_00005316 | zinc finger | at5g59820 | -2.3 | -0.5 | -0.9 |
| ABA_Pyr-down | TCONS_00007273 | ring finger | at5g27420 | -2.2 | 0.1 | -0.9 |
| ABA_Pyr-down | TCONS_00037700 | partial | at5g06320 | -2.1 | -0.3 | -0.9 |
| ABA_Pyr-down | TCONS_00002996 | vq motif-containing protein | at3g22160 | -1.7 | -0.1 | -0.9 |
| ABA_Pyr-down | TCONS_00008075 | elicitor-activated gene 3 | at4g37990 | -1.7 | -0.1 | -0.9 |
| ABA_Pyr-down | TCONS_00001408 | ethylene responsive element binding factor 2 | at4g17500 | -1.7 | 0.0 | -0.9 |
| ABA_Pyr-down | TCONS_00019646 | UDP-glucosyl transferase 74b1 | at1g24100 | -1.5 | -0.1 | -0.9 |
| ABA_Pyr-down | TCONS_00017697 | syntaxin 121 family | at3g11820 | -1.5 | -0.2 | -0.9 |
| ABA_Pyr-down | TCONS_00000706 | serine threonine-protein kinase cx32 | at4g35600 | -1.5 | -0.4 | -0.9 |
| ABA_Pyr-down | TCONS_00006314 | glutathione s-transferase | at2g02930 | -1.4 | 0.1 | -0.9 |
| ABA_Pyr-down | TCONS_00000163 | serine threonine kinase | at1g61610 | -1.1 | -0.5 | -0.9 |
| ABA_Pyr-down | TCONS_00023656 | 4-cumarate-COA-ligase | at1g51680 | -1.1 | -0.5 | -0.9 |
| ABA_Pyr-down | TCONS_00005814 | probable ribose-5-phosphate isomerase-like | at1g71100 | -0.8 | -0.4 | -0.9 |
| ABA_Pyr-down | TCONS_00009747 | protease inhibitor | -- | -2.5 | -0.4 | -0.8 |
| ABA_Pyr-down | TCONS_00005873 | lipoxygenase 3 | at1g72520 | -2.5 | 0.3 | -0.8 |
| ABA_Pyr-down | TCONS_00017130 | MLO 2 | at2g39200 | -2.2 | 0.1 | -0.8 |
| ABA_Pyr-down | TCONS_00009855 | zinc finger CCCH domain-containing 29 | at2g40140 | -1.9 | -0.3 | -0.8 |
| ABA_Pyr-down | TCONS_00027235 | cytochrome P450 | at3g26170 | -1.7 | 0.1 | -0.8 |
| ABA_Pyr-down | TCONS_00012338 | anthranilate synthase beta subunit | at1g25220 | -1.5 | 0.3 | -0.8 |
| ABA_Pyr-down | TCONS_00005891 | TIR-NBS class of disease resistance | at1g72890 | -1.5 | -0.1 | -0.8 |
| ABA_Pyr-down | TCONS_00022039 | sulfotransferase 16 | at1g74100 | -1.4 | 0.6 | -0.8 |
| ABA_Pyr-down | TCONS_00014147 | trypsin inhibitor 1 | p26780\|iti2_sinal | -1.3 | -0.1 | -0.8 |
| ABA_Pyr-down | TCONS_00014786 | syntaxin synt4 | at3g52400 | -1.1 | -0.0 | -0.8 |
| ABA_Pyr-down | TCONS_00000679 | thaumatin-like protein 1-like | at4g36010 | -1.0 | -0.0 | -0.8 |
| ABA_Pyr-down | TCONS_00016931 | yellow-leaf-specific gene 9 | at2g35980 | -3.1 | 0.5 | -0.7 |
| ABA_Pyr-down | TCONS_00032639 | TET8 ARATH ame: Full=Tetraspanin-8 | at2g23810 | -2.0 | -0.1 | -0.7 |
| ABA_Pyr-down | TCONS_00009770 | Nitrate and chloride transporter | at2g39210 | -1.8 | 0.2 | -0.7 |
| ABA_Pyr-down | TCONS_00031722 | L-type lectin-domain containing receptor kinase | at3g53810 | -1.5 | -0.3 | -0.7 |
| ABA_Pyr-down | TCONS_00008441 | NAC domain-containing 102 | at5g08790 | -1.5 | -0.2 | -0.7 |
| ABA_Pyr-down | TCONS_00002948 | calcium-transporting ATPase plasma membrane-type-like | at3g22910 | -1.5 | -0.5 | -0.7 |
| ABA_Pyr-down | TCONS_00022797 | Argonaute family | at1g31280 | -1.5 | -0.3 | -0.7 |
| ABA_Pyr-down | TCONS_00023687 | serine threonine kinase | at4g23190 | -1.3 | -0.2 | -0.7 |
| ABA_Pyr-down | TCONS_00028998 | WRKY DNA-binding 18 | at4g31800 | -1.3 | -0.3 | -0.7 |
| ABA_Pyr-down | TCONS_00014532 | transcription factor AS1 | at2g37630 | -1.0 | -0.3 | -0.7 |
| ABA_Pyr-down | TCONS_00032012 | beta-1,3-glucanase 2 | at3g57260 | -2.8 | 1.4 | -0.6 |
| ABA_Pyr-down | TCONS_00006660 | ethylene responsive element binding factor 2 | at5g47220 | -1.9 | -0.2 | -0.6 |
| ABA_Pyr-down | TCONS_00008315 | partial | at5g06320 | -1.7 | -0.2 | -0.6 |
| ABA_Pyr-down | TCONS_00027674 | cytochrome P450 | -- | -1.6 | 0.3 | -0.6 |
| ABA_Pyr-down | TCONS_00007985 | basic endochitinase | q09023\|chi2_brana | -1.5 | -0.3 | -0.6 |
| ABA_Pyr-down | TCONS_00002721 | glucose-6-phosphate phosphate translocator chloroplastic-like | at1g61800 | -1.5 | -0.0 | -0.6 |
| ABA_Pyr-down | TCONS_00018063 | glutaredoxin ATGRXS13 | at1g03850 | -1.4 | -0.0 | -0.6 |
| ABA_Pyr-down | TCONS_00031424 | UDP-glucuronate 4-epimerase 6-like | at3g23820 | -1.4 | -0.0 | -0.6 |
| ABA_Pyr-down | TCONS_00020741 | basic endochitinase | q09023\|chi2_brana | -1.1 | 0.6 | -0.6 |
| ABA_Pyr-down | TCONS_00006376 | allene oxide synthase | at5g42650 | -1.1 | 0.1 | -0.6 |
| ABA_Pyr-down | TCONS_00005948 | desulfoglucosinolate sulfotransferase | at1g74100 | -1.1 | 0.2 | -0.6 |
| ABA_Pyr-down | TCONS_00011983 | mitogen-activated kinase 3 | at3g45640 | -0.9 | -0.1 | -0.6 |
| ABA_Pyr-down | TCONS_00028344 | abscisic acid 8 -hydroxylase 1 | at4g19230 | -0.9 | -0.2 | -0.6 |
| ABA_Pyr-down | TCONS_00006377 | l-type lectin-domain containing receptor kinase -like | at2g37710 | -0.9 | -0.0 | -0.6 |
| ABA_Pyr-down | TCONS_00023943 | concanavalin A-like lectin kinase | at3g59700 | -2.3 | 0.2 | -0.5 |
| ABA_Pyr-down | TCONS_00004017 | myb domain 78 | at3g06490 | -1.8 | -0.0 | -0.5 |
| ABA_Pyr-down | TCONS_00037766 | anthranilate synthase component I-1 | at5g05730 | -1.6 | 1.4 | -0.5 |
| ABA_Pyr-down | TCONS_00010103 | pectinesterase 2-like | at2g45220 | -1.1 | -0.0 | -0.5 |
| ABA_Pyr-down | TCONS_00026860 | WRKY DNA-binding 28 | at5g46350 | -1.1 | -0.2 | -0.5 |
| ABA_Pyr-down | TCONS_00016122 | serine threonine kinase | at4g23180 | -2.2 | 0.6 | -0.4 |
| ABA_Pyr-down | TCONS_00006712 | by genscan and genefinder | at2g01300 | -2.1 | -0.0 | -0.4 |
| ABA_Pyr-down | TCONS_00026859 | WRKY DNA-binding 28 | at5g46350 | -1.1 | 0.1 | -0.4 |
| ABA_Pyr-down | TCONS_00033111 | L-type lectin-domain containing receptor kinase -like | at1g15530 | -1.0 | 0.0 | -0.4 |
| ABA_Pyr-down | TCONS_00034470 | callose synthase 12-like | at4g03550 | -1.0 | -0.3 | -0.4 |
| ABA_Pyr-down | TCONS_00010262 | patatin group A-3-like | at2g26560 | -2.2 | 0.8 | -0.3 |
| ABA_Pyr-down | TCONS_00014106 | pirin -like | at2g43120 | -1.7 | -0.0 | -0.3 |
| ABA_Pyr-down | TCONS_00009838 | nematode resistance | at2g40000 | -1.0 | -0.1 | -0.3 |
| ABA_Pyr-down | TCONS_00010263 | phospholipase A 2A | at2g26560 | -2.2 | 0.6 | -0.2 |
| ABA_Pyr-down | TCONS_00005458 | cytochrome P450 monooxygenase | at5g57220 | -1.4 | 0.6 | -0.2 |
| ABA_Pyr-down | TCONS_00027234 | cytochrome P450 | at3g26160 | -1.3 | -0.3 | -0.2 |
| ABA_Pyr-down | TCONS_00004111 | dehydroascorbate reductase | at1g75270 | -1.2 | 1.0 | -0.2 |
| ABA_Pyr-down | TCONS_00011799 | 2-nitropropane dioxygenase | at5g64250 | -1.0 | 0.1 | -0.2 |
| ABA_Pyr-down | TCONS_00011738 | ethylene-responsive transcription factor ABR1-like | at5g64750 | -2.3 | 0.1 | -0.1 |
| ABA_Pyr-down | TCONS_00018783 | pleiotropic drug resistance 1-like | at1g15520 | -2.1 | 0.2 | -0.1 |
| ABA_Pyr-down | TCONS_00027505 | proline dehydrogenase | at3g30775 | -1.8 | 0.0 | -0.1 |
| ABA_Pyr-down | TCONS_00021118 | myb domain 78 | at3g06490 | -1.4 | 0.0 | -0.1 |
| ABA_Pyr-down | TCONS_00031667 | nodulin glutamate-ammonia ligase | at3g53180 | -1.2 | -0.0 | -0.1 |
| ABA_Pyr-down | TCONS_00007080 | proline dehydrogenase | at3g30775 | -2.1 | -0.1 | 0.0 |
| ABA_Pyr-down | TCONS_00035202 | WRKY transcription factor 8 | -- | -Inf | 1.8 | 0.5 |

## S4.7-12 DE transcripts upregulated or downregulated per each treatment and annotated “GO:0000302 Response to reactive oxygen species”.

### ABA-up

Table S4.7. Upregulated gene products after 8 days of ABA treatment and annotated “GO:0000302 Response to reactive oxygen species”. Treat, treatment and change sign. Gene name, as annotated by Blast2GO after best blastx in nr Viridiplantae database. Best Swiss-Prot hit (Uniprot manually annotated and reviewed protein database) as *Arabidopsis thaliana* locus name or Uniprot “Entry|Entry name” if best curated hit is from another species. ABA, Pyr and ABA+Pyr log2 fold change to control condition.

| Treat | Isoform id | Gene name | Best Swiss-Prot hit | ABA | Pyr | ABA+Pyr |
| --- | --- | --- | --- | --- | --- | --- |
| No DE isoforms |  |  |  |  |  |  |

### ABA-down

Table S4.8. Downregulated gene products after 8 days of ABA treatment and annotated “GO:0000302 Response to reactive oxygen species”. Treat, treatment and change sign. Gene name, as annotated by Blast2GO after best blastx in nr Viridiplantae database. Best Swiss-Prot hit (Uniprot manually annotated and reviewed protein database) as *Arabidopsis thaliana* locus name or Uniprot “Entry|Entry name” if best curated hit is from another species. ABA, Pyr and ABA+Pyr log2 fold change to control condition.

| Treat | Isoform id | Gene name | Best Swiss-Prot hit | ABA | Pyr | ABA+Pyr |
| --- | --- | --- | --- | --- | --- | --- |
| ABA-down | TCONS_00027759 | probable WRKY transcription factor 41-like | at5g24110 | -3.1 | -0.4 | -3.2 |
| ABA-down | TCONS_00007679 | 12-oxophytodienoate reductase 3-like | at2g06050 | -1.1 | -0.5 | -1.3 |
| ABA-down | TCONS_00020925 | dehydration-responsive element-binding 2A | at3g11020 | -3.3 | -0.6 | -1.2 |
| ABA-down | TCONS_00028326 | heat stress transcription factor A-4a-like | at4g18880 | -2.5 | -0.3 | -1.2 |
| ABA-down | TCONS_00022652 | probable calcium-binding CML23-like | at5g37770 | -1.1 | -0.4 | -1.1 |
| ABA-down | TCONS_00025476 | mitogen-activated kinase kinase kinase 19 | at5g67080 | -2.2 | -0.3 | -1.0 |
| ABA-down | TCONS_00011495 | 12-oxophytodienoate reductase 3 | at2g06050 | -1.0 | -0.1 | -1.0 |

### Pyr-up

Table S4.9. Upregulated gene products after 8 days of Pyr treatment and annotated “GO:0000302 Response to reactive oxygen species”. Treat, treatment and change sign. Gene name, as annotated by Blast2GO after best blastx in nr Viridiplantae database. Best Swiss-Prot hit (Uniprot manually annotated and reviewed protein database) as Arabidopsis thaliana locus name or Uniprot “Entry|Entry name” if best curated hit is from another species. ABA, Pyr and ABA+Pyr log2 fold change to control condition.

| Treat | Isoform id | Gene name | Best Swiss-Prot hit | ABA | Pyr | ABA+Pyr |
| --- | --- | --- | --- | --- | --- | --- |
| No DE isoforms |  |  |  |  |  |  |

### Pyr-down

Table S4.10. Downregulated gene products after 8 days of Pyr treatment and annotated “GO:0000302 Response to reactive oxygen species”. Treat, treatment and change sign. Gene name, as annotated by Blast2GO after best blastx in nr Viridiplantae database. Best Swiss-Prot hit (Uniprot manually annotated and reviewed protein database) as Arabidopsis thaliana locus name or Uniprot “Entry|Entry name” if best curated hit is from another species. ABA, Pyr and ABA+Pyr log2 fold change to control condition.

| Treat | Isoform id | Gene name | Best Swiss-Prot hit | ABA | Pyr | ABA+Pyr |
| --- | --- | --- | --- | --- | --- | --- |
| No DE isoforms |  |  |  |  |  |  |

### ABA+Pyr-up

Table S4.11. Upregulated gene products after 8 days of ABA+Pyr treatment and annotated “GO:0000302 Response to reactive oxygen species”. Treat, treatment and change sign. Gene name, as annotated by Blast2GO after best blastx in nr Viridiplantae database. Best Swiss-Prot hit (Uniprot manually annotated and reviewed protein database) as Arabidopsis thaliana locus name or Uniprot “Entry|Entry name” if best curated hit is from another species. ABA, Pyr and ABA+Pyr log2 fold change to control condition.

| Treat | Isoform id | Gene name | Best Swiss-Prot hit | ABA | Pyr | ABA+Pyr |
| --- | --- | --- | --- | --- | --- | --- |
| ABA_Pyr-up | TCONS_00032575 | glutathione peroxidase partial | at4g31870 | 1.4 | 0.8 | 0.7 |
| ABA_Pyr-up | TCONS_00005365 | kinase family | at5g58950 | 1.0 | 0.5 | 0.8 |

### ABA+Pyr-down

Table S4.12. Downregulated gene products after 8 days of ABA+Pyr treatment and annotated “GO:0000302 Response to reactive oxygen species”. Treat, treatment and change sign. Gene name, as annotated by Blast2GO after best blastx in nr Viridiplantae database. Best Swiss-Prot hit (Uniprot manually annotated and reviewed protein database) as Arabidopsis thaliana locus name or Uniprot “Entry|Entry name” if best curated hit is from another species. ABA, Pyr and ABA+Pyr log2 fold change to control condition.

| Treat | Isoform id | Gene name | Best Swiss-Prot hit | ABA | Pyr | ABA+Pyr |
| --- | --- | --- | --- | --- | --- | --- |
| ABA_Pyr-down | TCONS_00035202 | WRKY transcription factor 8 | -- | -Inf | 1.8 | 0.5 |
| ABA_Pyr-down | TCONS_00020925 | dehydration-responsive element-binding 2A | at3g11020 | -3.3 | -0.6 | -1.2 |
| ABA_Pyr-down | TCONS_00027759 | probable WRKY transcription factor 41-like | at5g24110 | -3.1 | -0.4 | -3.2 |
| ABA_Pyr-down | TCONS_00028326 | heat stress transcription factor A-4a-like | at4g18880 | -2.5 | -0.3 | -1.2 |
| ABA_Pyr-down | TCONS_00005873 | lipoxygenase 3 | at1g72520 | -2.5 | 0.3 | -0.8 |
| ABA_Pyr-down | TCONS_00000531 | BTB and TAZ domain 4 | at4g37610 | -2.4 | 0.0 | -0.4 |
| ABA_Pyr-down | TCONS_00037786 | dehydration-responsive element-binding 2A | at5g05410 | -2.2 | -0.2 | -0.0 |
| ABA_Pyr-down | TCONS_00025476 | mitogen-activated kinase kinase kinase 19 | at5g67080 | -2.2 | -0.3 | -1.0 |
| ABA_Pyr-down | TCONS_00018783 | pleiotropic drug resistance 1-like | at1g15520 | -2.1 | 0.2 | -0.1 |
| ABA_Pyr-down | TCONS_00004514 | dehydration-responsive element-binding protein 2a | at5g05410 | -2.0 | -0.1 | -0.3 |
| ABA_Pyr-down | TCONS_00001996 | heat shock protein | at4g25200 | -1.9 | -0.3 | -0.0 |
| ABA_Pyr-down | TCONS_00010910 | 70 kDa heat shock | at3g12580 | -1.8 | -0.4 | -1.1 |
| ABA_Pyr-down | TCONS_00003593 | protein fyd | at3g12570 | -1.8 | -0.3 | -1.0 |
| ABA_Pyr-down | TCONS_00010237 | heat stress transcription factor A-2 | at2g26150 | -1.8 | -0.0 | -0.1 |
| ABA_Pyr-down | TCONS_00029220 | BTB POZ and TAZ domain-containing 4-like | at4g37610 | -1.8 | -0.3 | -0.3 |
| ABA_Pyr-down | TCONS_00022346 | transcription factor FER-LIKE IRON DEFICIENCY-INDUCED TRANSCRIPTION FACTOR-like | at2g28160 | -1.6 | -0.6 | -0.8 |
| ABA_Pyr-down | TCONS_00023257 | BTB and TAZ domain 1 | at5g63160 | -1.5 | -0.1 | -0.1 |
| ABA_Pyr-down | TCONS_00010236 | heat shock transcription factor A2 | at2g26150 | -1.5 | -0.4 | -0.2 |
| ABA_Pyr-down | TCONS_00033826 | AAA-ATPase 1 | at3g28580 | -1.5 | -0.1 | -0.7 |
| ABA_Pyr-down | TCONS_00000577 | heat shock transcription factor HSF4 | at4g36990 | -1.2 | -0.2 | -0.7 |
| ABA_Pyr-down | TCONS_00004111 | dehydroascorbate reductase | at1g75270 | -1.2 | 1.0 | -0.2 |
| ABA_Pyr-down | TCONS_00015146 | purple acid phosphatase 17 | at3g17790 | -1.2 | -0.3 | -0.4 |
| ABA_Pyr-down | TCONS_00022022 | heat shock 101 | at1g74300 | -1.2 | -0.1 | 0.1 |
| ABA_Pyr-down | TCONS_00026859 | WRKY DNA-binding 28 | at5g46350 | -1.1 | 0.1 | -0.4 |
| ABA_Pyr-down | TCONS_00007679 | 12-oxophytodienoate reductase 3-like | at2g06050 | -1.1 | -0.5 | -1.3 |
| ABA_Pyr-down | TCONS_00026860 | WRKY DNA-binding 28 | at5g46350 | -1.1 | -0.2 | -0.5 |
| ABA_Pyr-down | TCONS_00022652 | probable calcium-binding CML23-like | at5g37770 | -1.1 | -0.4 | -1.1 |
| ABA_Pyr-down | TCONS_00033585 | L-ascorbate peroxidase 1 | at1g07890 | -1.0 | -0.0 | -0.8 |
| ABA_Pyr-down | TCONS_00006903 | cytochrome P450 | at3g26200 | -1.0 | 0.2 | -0.3 |
| ABA_Pyr-down | TCONS_00011495 | 12-oxophytodienoate reductase 3 | at2g06050 | -1.0 | -0.1 | -1.0 |

## S4.13-18 DE transcripts upregulated or downregulated per each treatment and annotated “GO:0016209 Antioxidant activity”.

### ABA-up

Table S4.13. Upregulated gene products after 8 days of ABA treatment and annotated “GO:0016209 Antioxidant activity”. Treat, treatment and change sign. Gene name, as annotated by Blast2GO after best blastx in nr Viridiplantae database. Best Swiss-Prot hit (Uniprot manually annotated and reviewed protein database) as *Arabidopsis thaliana* locus name or Uniprot “Entry|Entry name” if best curated hit is from another species. ABA, Pyr and ABA+Pyr log2 fold change to control condition.

| Treat | Isoform id | Gene name | Best Swiss-Prot hit | ABA | Pyr | ABA+Pyr |
| --- | --- | --- | --- | --- | --- | --- |
| ABA-up | TCONS_00009632 | peroxidase ATP2a | at2g37130 | 0.7 | 0.3 | 1.3 |
| ABA-up | TCONS_00003659 | 2-cys peroxiredoxin bas1 | at3g11630 | 0.9 | 0.5 | 1.4 |
| ABA-up | TCONS_00017007 | peroxidase ATP2a | at2g37130 | 0.7 | 0.0 | 1.4 |
| ABA-up | TCONS_00001702 | peroxidase | at4g21960 | 1.6 | 0.4 | 1.8 |

### ABA-down

Table S4.14. Downregulated gene products after 8 days of ABA treatment and annotated “GO:0016209 Antioxidant activity”. Treat, treatment and change sign. Gene name, as annotated by Blast2GO after best blastx in nr Viridiplantae database. Best Swiss-Prot hit (Uniprot manually annotated and reviewed protein database) as *Arabidopsis thaliana* locus name or Uniprot “Entry|Entry name” if best curated hit is from another species. ABA, Pyr and ABA+Pyr log2 fold change to control condition.

| Treat | Isoform id | Gene name | Best Swiss-Prot hit | ABA | Pyr | ABA+Pyr |
| --- | --- | --- | --- | --- | --- | --- |
| ABA-down | TCONS_00031932 | manganese superoxide dismutase | at3g56350 | -2.5 | -0.3 | -1.4 |

### Pyr-up

Table S4.15. Upregulated gene products after 8 days of Pyr treatment and annotated “GO:0016209 Antioxidant activity”. Treat, treatment and change sign. Gene name, as annotated by Blast2GO after best blastx in nr Viridiplantae database. Best Swiss-Prot hit (Uniprot manually annotated and reviewed protein database) as Arabidopsis thaliana locus name or Uniprot “Entry|Entry name” if best curated hit is from another species. ABA, Pyr and ABA+Pyr log2 fold change to control condition.

| Treat | Isoform id | Gene name | Best Swiss-Prot hit | ABA | Pyr | ABA+Pyr |
| --- | --- | --- | --- | --- | --- | --- |
| Pyr-up | TCONS_00034007 | peroxidase | at5g06730 | -0.2 | 1.2 | 0.1 |

### Pyr-down

Table S16. Downregulated gene products after 8 days of Pyr treatment and annotated “GO:0016209 Antioxidant activity”. Treat, treatment and change sign. Gene name, as annotated by Blast2GO after best blastx in nr Viridiplantae database. Best Swiss-Prot hit (Uniprot manually annotated and reviewed protein database) as Arabidopsis thaliana locus name or Uniprot “Entry|Entry name” if best curated hit is from another species. ABA, Pyr and ABA+Pyr log2 fold change to control condition.

| Treat | Isoform id | Gene name | Best Swiss-Prot hit | ABA | Pyr | ABA+Pyr |
| --- | --- | --- | --- | --- | --- | --- |
| No DE isoforms |  |  |  |  |  |  |

### ABA+Pyr-up

Table S4.17. Upregulated gene products after 8 days of ABA+Pyr treatment and annotated “GO:0016209 Antioxidant activity”. Treat, treatment and change sign. Gene name, as annotated by Blast2GO after best blastx in nr Viridiplantae database. Best Swiss-Prot hit (Uniprot manually annotated and reviewed protein database) as Arabidopsis thaliana locus name or Uniprot “Entry|Entry name” if best curated hit is from another species. ABA, Pyr and ABA+Pyr log2 fold change to control condition.

| Treat | Isoform id | Gene name | Best Swiss-Prot hit | ABA | Pyr | ABA+Pyr |
| --- | --- | --- | --- | --- | --- | --- |
| ABA_Pyr-up | TCONS_00019736 | peroxidase 30 | at3g21770 | 1.8 | 0.3 | 0.2 |
| ABA_Pyr-up | TCONS_00001702 | peroxidase | at4g21960 | 1.6 | 0.4 | 1.8 |
| ABA_Pyr-up | TCONS_00032575 | glutathione peroxidase partial | at4g31870 | 1.4 | 0.8 | 0.7 |
| ABA_Pyr-up | TCONS_00022086 | alpha dioxygenase | at1g73680 | 0.9 | 0.4 | 0.8 |

### ABA+Pyr-down

Table S4.18. Downregulated gene products after 8 days of ABA+Pyr treatment and annotated “GO:0016209 Antioxidant activity”. Treat, treatment and change sign. Gene name, as annotated by Blast2GO after best blastx in nr Viridiplantae database. Best Swiss-Prot hit (Uniprot manually annotated and reviewed protein database) as Arabidopsis thaliana locus name or Uniprot “Entry|Entry name” if best curated hit is from another species. ABA, Pyr and ABA+Pyr log2 fold change to control condition.

| Treat | Isoform id | Gene name | Best Swiss-Prot hit | ABA | Pyr | ABA+Pyr |
| --- | --- | --- | --- | --- | --- | --- |
| ABA_Pyr-down | TCONS_00031932 | manganese superoxide dismutase | at3g56350 | -2.5 | -0.3 | -1.4 |
| ABA_Pyr-down | TCONS_00033802 | glutathione peroxidase | at2g48150 | -2.2 | -0.4 | -0.6 |
| ABA_Pyr-down | TCONS_00004111 | dehydroascorbate reductase | at1g75270 | -1.2 | 1.0 | -0.2 |
| ABA_Pyr-down | TCONS_00037110 | major facilitator | at5g14130 | -1.2 | -0.2 | -0.5 |
| ABA_Pyr-down | TCONS_00015146 | purple acid phosphatase 17 | at3g17790 | -1.2 | -0.3 | -0.4 |
| ABA_Pyr-down | TCONS_00009517 | glutathione peroxidase | at2g31570 | -1.1 | -0.1 | -0.7 |
| ABA_Pyr-down | TCONS_00033585 | L-ascorbate peroxidase 1 | at1g07890 | -1.0 | -0.0 | -0.8 |
| ABA_Pyr-down | TCONS_00031932 | manganese superoxide dismutase | at3g56350 | -2.5 | -0.3 | -1.4 |
| ABA_Pyr-down | TCONS_00033802 | glutathione peroxidase | at2g48150 | -2.2 | -0.4 | -0.6 |
| ABA_Pyr-down | TCONS_00004111 | dehydroascorbate reductase | at1g75270 | -1.2 | 1.0 | -0.2 |
| ABA_Pyr-down | TCONS_00037110 | major facilitator | at5g14130 | -1.2 | -0.2 | -0.5 |
| ABA_Pyr-down | TCONS_00015146 | purple acid phosphatase 17 | at3g17790 | -1.2 | -0.3 | -0.4 |
| ABA_Pyr-down | TCONS_00009517 | glutathione peroxidase | at2g31570 | -1.1 | -0.1 | -0.7 |
| ABA_Pyr-down | TCONS_00033585 | L-ascorbate peroxidase 1 | at1g07890 | -1.0 | -0.0 | -0.8 |
